# Monocyte infiltration induces CNS arginine catabolism to fuel neuroinflammation

**DOI:** 10.1038/s41590-026-02516-4

**Published:** 2026-05-18

**Authors:** Martina Kerndl, Laszlo Musiejovsky, Andrea Komljenovic, Hon Shing Lam, Andrea Vogel, Tobias Bausbacher, Christian J. Riedl, Roko Sango, Lenka Matejovicova, Anja Dobrijevic, Laura Oberbichler, Melanie Hofmann, Markus Kieler, Lucia Quemada Garrido, Lara Veronika Perko Budja, Julia Stefanie Brunner, James Lucas Cairns, Paul Cheng, Kerstin Kitt, Christine Isaguirre, Thomas Köcher, Kristaps Klavins, Thomas Rattei, Simon Hametner, Stephan Blüml, Carsten Hopf, Ryan D. Sheldon, Omar Sharif, Gernot Schabbauer

**Affiliations:** 1 Institute of Vascular Biology and Thrombosis Research, Center for Physiology and Pharmacology, Medical University of Vienna, Vienna, Austria; 2 Christian Doppler Laboratory for Arginine Metabolism in Rheumatoid Arthritis and Multiple Sclerosis, Vienna, Austria; 3 Christian Doppler Laboratory for Immunometabolism and Systems Biology of Obesity-Related Diseases (InSpiReD), Vienna, Austria; 4 Center for Mass Spectrometry and Optical Spectroscopy (CeMOS), Technische Hochschule Mannheim, Mannheim, Germany; 5 Mannheim Center for Translational Neuroscience (MCTN), Medical Faculty Mannheim, Heidelberg University, Mannheim, Germany; 6 Divsion of Neuropathology and Neurochemistry, Department of Neurology, Medical University of Vienna, Vienna, Austria; 7 Comprehensive Center for Clinical Neurosciences and Mental Health, Medical University of Vienna, Vienna, Austria; 8 Center for Pathobiochemistry and Genetics, Institute of Medical Genetics, Medical University of Vienna, Vienna, Austria; 9 Center for Microbiology and Environmental Systems Science, University of Vienna, Vienna, Austria; 10 Doctoral School in Microbiology and Environmental Science, University of Vienna, Vienna, Austria; 11 SFB Immunometabolism, Medical University of Vienna, Vienna, Austria; 12 Medical Faculty, Heidelberg University, Heidelberg, Germany; 13 Bio Cancer Treatment International, Hong Kong, China; 14 Department for Cancer Immunology and Immune Modulation, Boehringer Ingelheim Pharma, Biberach, Germany; 15 Mass Spectrometry Core, Van Andel Institute, Grand Rapids, MI USA; 16 Vienna Biocenter Core Facilities, Vienna, Austria; 17 Institute of Biomaterials and Bioengineering, Faculty of Natural Sciences and Technology, Riga Technical University, Riga, Latvia; 18 Division of Rheumatology, Department of Internal Medicine III, Medical University of Vienna, Vienna, Austria; 19 Present Address: LoopLab Bio, Vienna, Austria; 20 Present Address: Institute of Experimental Immunology, University of Zürich, Zurich, Switzerland; 21 Present Address: Cell Biology Program, Memorial Sloan Kettering Cancer Center, New York, NY USA

**Keywords:** Neuroimmunology, Autoimmunity, Inflammation, Animal disease models

## Abstract

Inflammatory responses are associated with recruitment of monocyte-derived cells (Mdcs) into tissues. Although tissue-specific Mdc reprogramming is well established, how Mdc infiltration alters tissue metabolism remains unclear. Here, using a mouse neuroinflammation model coupled with genetic fate mapping, metabolomics and metabolite imaging, we identify that central nervous system (CNS) Mdc infiltration is associated with substantial metabolic changes and assign disease-linked metabolites therein. In particular, we found that increased arginine catabolism driven by lesion-associated arginase 1 (*Arg1*)-expressing Mdcs promoted oxidative damage, lipid accumulation and Mdc dysfunction. Genetic ARG1 deficiency within Mdcs during neuroinflammation increased extracellular arginine and was associated with rewiring of the CNS metabolic landscape, including attenuated disease-linked metabolites. This was accompanied by enhanced Mdc-driven anti-inflammation, regulatory T cell expansion and improved disease outcome. Opposing effects were observed following dietary arginine deficiency. Together, our work highlights key roles for Mdcs in CNS metabolism and reveals the pleiotropic beneficial effects of arginine in neuroinflammation.

## Abstract (web summary)

Kerndl and colleagues identify that arginase 1-expressing monocyte-derived cells mediate arginine catabolism and detrimental effects during experimental autoimmune encephalitis-triggered neuroinflammation.

## Main

In the central nervous system (CNS), at homeostasis, choroid plexus macrophages at interfaces between blood and cerebrospinal fluid (CSF) and macrophages within the dura mater are CNS-associated macrophage (CAM) populations replenished by monocytes^1,2^. Mouse patrolling Ly6C^lo^CCR2^−^CX3CR^hi^ monocytes play homeostatic functions, whereas classical inflammatory Ly6C^hi^CCR2^+^CX3CR1^lo^ monocytes are recruited to sites of inflammation, where they differentiate into dendritic cells (moDCs) and macrophages (moMACs), with CCR2 being important for monocyte migration from bone marrow to tissues^3^. CAMs express a different transcriptional signature than resident microglia, and although during neuroinflammation several distinct monocyte, moDC and moMAC populations transiently emerge through the blood–brain barrier, triggering autoimmune neuroinflammation, these monocyte-derived cells (Mdcs) do not contribute to the microglial pool^4–8^. The transcriptome and effector functions of Mdcs in neuroinflammatory disease is instructed by proinflammatory cytokines, including interferon-γ (IFNγ) and granulocyte macrophage colony-stimulating factor (GM-CSF), with subsets of moMACs and moDCs simultaneously expressing key urea cycle components including arginase 1 (*Arg1*) and nitric oxide synthase 2 (*Nos2*)^5,9^. Although this suggests a complex CNS Mdc evolution, other work indicates a unidirectional path comprising initial infiltration of *Nos2*-expressing Mdcs at peak disease that are slowly replaced by *Arg1*-expressing cells as neuroinflammatory disease resolves^10^. Aside from cytokines, metabolic fine-tuning is key for Mdc tissue adaptation, particularly under shifting nutrient availability^11^. Herein, CNS-associated *Nos2*-expressing Mdcs use glycolysis, whereas *Arg1*-expressing Mdcs use oxidative phosphorylation to generate ATP^10^, metabolic signatures established to promote pro- or anti-inflammation, respectively^11^. Further, many studies have used metabolomic approaches to identify biomarkers in blood, CSF and urine associated with progression of neuroinflammatory diseases, including multiple sclerosis (MS) and its mouse animal model experimental autoimmune encephalitis (EAE)^12–14^. Recently, metabolomics of isolated microglia revealed their critical role in promoting neuroinflammation^15^. Despite this progress, how Mdc influx directly influences CNS metabolism per se and the implications herein for inflammation remain poorly understood.

Here, we leveraged the transient nature of CNS myeloid cell influx during EAE to examine which metabolic aspects of this demyelinating neuroinflammatory disease are driven or suppressed by Mdcs and identified pivotal roles for arginine catabolism by ARG1^+^ Mdcs in neuroinflammation.

## Results

### CNS metabolic shifts in neuroinflammation coincide with monocyte influx

**Fig. 1:**
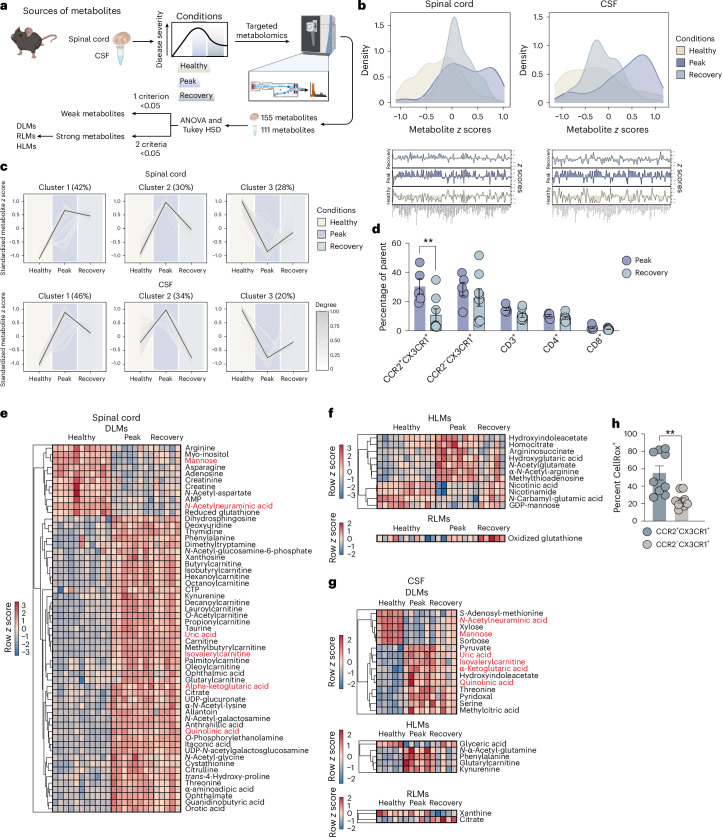
Neuroinflammation is associated with metabolic changes related to neurotoxicity, energy metabolism and redox homeostasis. **a**, Experimental scheme for identifying metabolites associated with EAE phases. Schematic created in BioRender; Kieler, M. https://biorender.com/2z6ava8 (2026); HSD, honestly significant difference. **b**, Top: density plot showing *z*-score distributions (*x* axis) of measured metabolites in the indicated groups. Bottom: bar plots representing *z* scores of each metabolite. **c**, Metabolite cluster plots showing trajectories of standardized metabolite *z* scores (*y* axis) over EAE disease phase (*x* axis) for each measured metabolite over biological replicate (gray lines). Shades of gray depict cluster membership degree. The black line depicts weighted average therein; spinal cord, *n* = 11 (healthy), *n* = 8 (peak disease) and *n* = 5 (recovery) animals; CSF, *n* = 5 animals for all experimental groups. **d**, Percentages of depicted spinal cord immune cell populations at peak disease (*n* = 5) and during recovery (*n* = 8); **P = 0.0012. **e**–**g**, Heat maps displaying degree of changes in spinal cord (**e**,**f**) or CSF (**g**) metabolite abundance among the different experimental groups. The heat map legend represents row *z* scores. DLMs are significant between healthy animals and animals at peak disease and between healthy animals and animals during recovery. RLMs are significant between animals at peak disease and during recovery and between healthy animals and animals during recovery. HLMs are significant between healthy animals and animals at peak disease and between animals at peak disease and animals during recovery. Matching metabolites in the spinal cord and CSF are highlighted in red. **h**, Percentage of CellRox^+^ cells among CCR2^+^CX3CR1^+^ or CCR2^−^CX3CR1^+^ cells during EAE; *n* = 9 animals; **P = 0.002. Results are shown as mean ± s.e.m. (**d**,**h**). Data were analyzed by a one-way analysis of variance (ANOVA) followed by a Tukey’s multiple-comparisons post-test (**e**–**g**) or two-tailed unpaired *t*-test (**d**,**h**). Cells were pregated on single viable cells and then CD45^hi^Ly6G^−^CD3^−^ for myeloid cells (**d**,**h**) or CD45^+^ for T cells (**d**). CTP, cytidine triphosphate. Source data

**Extended Data Fig. 1:**
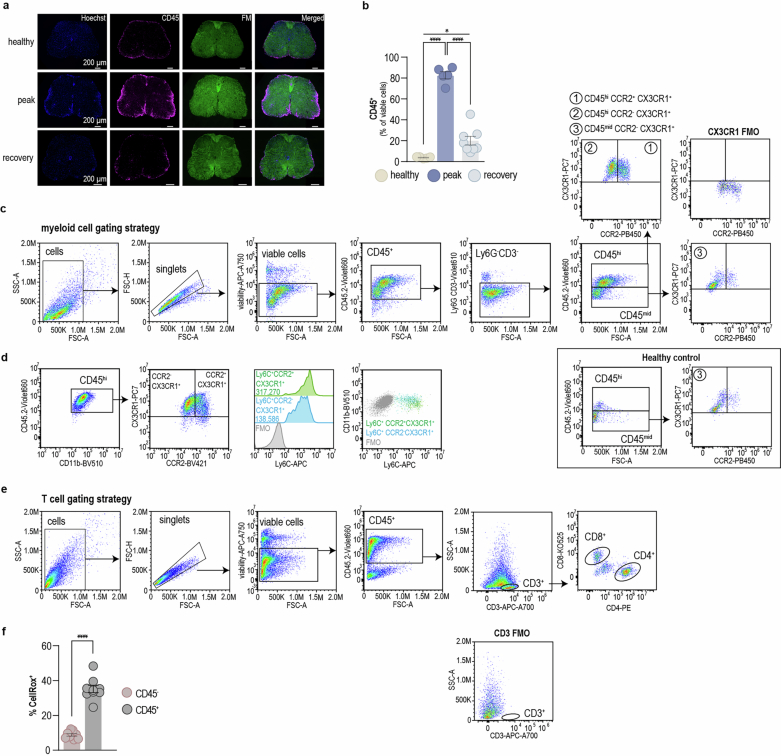
Gating strategies for immune cell populations and their characterization during neuroinflammation. **a**, Representative immunofluorescence depicting CD45^+^ cells and demyelination (fluoromyelin, FM) in spinal cord sections of C57BL/6J animals at the indicated disease-phases. Cy5, CD45-AF647; DAPI, Hoechst; FITC, fluoromyelin. Scale bar represents 200 μm. **b**, Percentages of viable spinal cord CD45^+^ cells in C57BL/6J animals. n = 5 (healthy, peak), n = 8 (recovery) animals. ANOVA ****P < 0.0001. *P = 0.0186, ****P < 0.0001. **c**, Gating strategy for myeloid cells. CD3^−^ cells gated on CD45^hi^ to exclude CD45^mid^ microglia, then on CCR2 and CX3CR1. A healthy control is included to demonstrate the fidelity of the populations. **d**, Gating strategy, representative plots depicting Ly6C expression in the indicated populations at peak-disease. The number in the histogram represents MFI of Ly6C within the indicated population. Cells gated on CD3^−^CD11b^+^CD45^hi^. **e**, Gating strategy for T cells. Cells gated on CD45^+^CD3^+^ then CD4^+^ or CD8^+^. **f**, CellRox flow cytometry assay depicting percentage of CellRox^+^ in CD45^+^ or CD45^−^ cells during EAE. n = 9 per group. ****P < 0.0001. Results are mean ± SEM (**b,f**). Statistical analysis performed with one-way ANOVA followed by Tukey multiple-comparisons post-test (**b**) or two-tailed unpaired t-test (**f**). Cells pre-gated on single, viable, Ly6G^−^ (**b,c**,**d**,**e**,**f**). Source data

**Extended Data Fig. 2:**
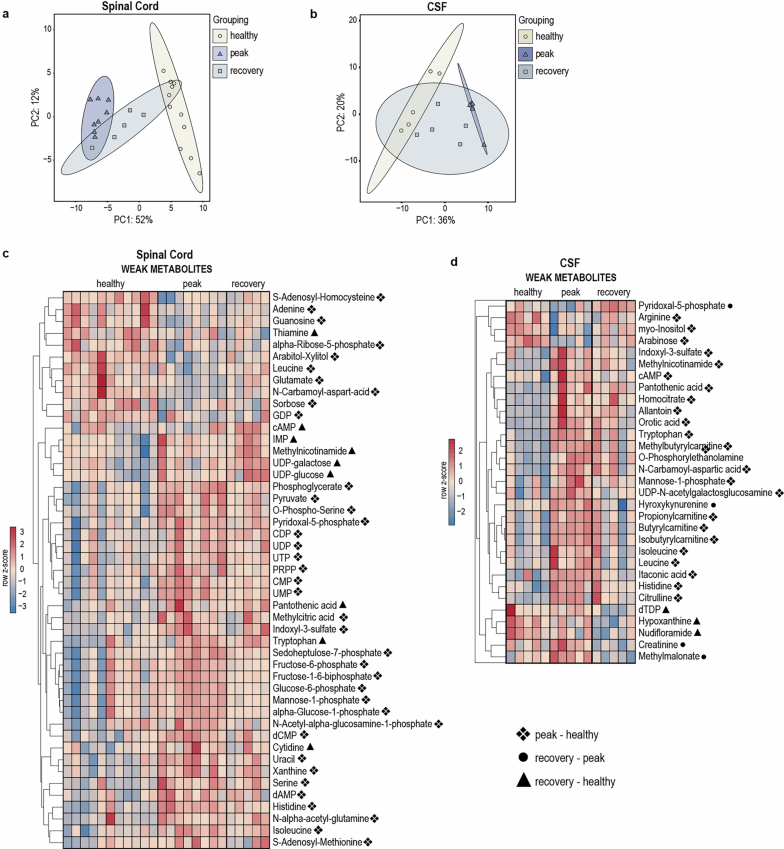
PCAs and heatmaps of CNS metabolites during neuroinflammation. **a**, Principal component analysis (PCA) of spinal cord metabolites in EAE. n = 11 (healthy), n = 8 (peak), n = 5 (recovery) animals. **b**, PCA of cerebrospinal fluid (CSF) metabolites in EAE. n = 5 animals for all experimental groups. **c/d**, Heatmap depicting significantly changed weak spinal cord (**c**) or CSF (**d**) metabolites. Heatmap legend represents row z-scores. Significance between groups is indicated with different symbols. Statistical analysis was performed with one-way ANOVA followed by Tukey multiple-comparisons post-test (**c/d**). Source data

To identify EAE-triggered metabolites and understand their relationship with myeloid cell influx, we immunized mice with myelin oligodendrocyte glycoprotein peptide 33–55 (MOG_35–55_) in complete Freund’s adjuvant, collected spinal cord tissue and CSF at peak disease or during recovery, performed targeted metabolomics and subsequently categorized metabolites at homeostasis and during different EAE phases (Fig. 1a and Supplementary Tables 1–4). Unimmunized animals were healthy controls. Substantial CD45^+^ cell influx, coinciding with demyelination, occurring at peak disease was attenuated at recovery (Extended Data Fig. 1a,b). Principal component analysis identified distinct metabolite separation between healthy animals and animals at peak disease, while recovered animals exhibited a scattered in-between profile, notably in the CSF (Extended Data Fig. 2a,b). Accordingly, spinal cord metabolites in healthy animals and recovered animals exhibited bimodal and unimodal distributions, respectively, peak disease metabolites in both tissues displayed the widest *z*-score spreads, and CSF metabolite distributions were more variable than those in the spinal cord (Fig. 1b and Extended Data Fig. 2a,b). Supervised clustering of metabolite trajectories over different EAE phases identified three patterns. Relative to healthy animals, at peak disease, most CNS metabolites increased and slightly (cluster 1) or strongly decreased (cluster 2) at recovery. We identified a cluster (cluster 3) of attenuated CNS metabolites in severely ill animals compared to in healthy controls (Fig. 1c and Supplementary Table 5), implying metabolite consumption or degradation at peak disease. Given the temporal changes in spinal cord CD45^+^ cells that were barely detectable in healthy animals (Extended Data Fig. 1a,b), we next investigated which immune cells were predominant during the distinct EAE phases and possibly linked to metabolite accumulation or consumption reflected in the cluster analysis. Examining infiltrating myeloid cell contributions, we determined percentages of CCR2^+^CX3CR1^+^/CCR2^−^CX3CR1^+^ cells, excluding CD45^mid^Ly6G^+^CD3^+^ cells (microglia, neutrophils and T cells) from the analysis (Extended Data Fig. 1c). CX3CR1^+^CCR2^+^ Mdcs were elevated at peak disease versus during recovery (Fig. 1d). We noted concurrent CCR2 and Ly6C downregulation therein (Extended Data Fig. 1c,d), suggesting that Mdcs were derived from circulating Ly6C^hi^CX3CR1^lo^ monocytes downregulating CCR2 expression after CNS entry^3,16^. There were no differences in T cells (CD4^+^ and CD8^+^) between disease phases (Fig. 1d and Extended Data Fig. 1e).

Having established Mdc frequency shifts between EAE phases, we examined which metabolites are specifically related to disease, recovery or homeostasis and identified 57 spinal cord and 15 CSF disease-linked metabolites (DLMs), up or down, relative to healthy animals (Fig. 1a,e–g). Neurotoxic quinolinic acid and α-aminoadipic acid, metabolites produced by CAMs and activated microglia or glial cells, respectively, and reported to induce oxidative stress, lipid peroxidation and oligodendrocyte and neuronal death, were augmented^17–20^. This, along with decreased reduced glutathione, an endogenous antioxidant, indicated substantial oxidative stress at peak disease^21^ (Fig. 1e). We observed decreased adenosine, adenosine monophosphate and neuroprotective creatine^22,23^, suggesting impaired energy metabolism and mitochondrial dysfunction in diseased spinal cord. Multiple carnitines were elevated, suggesting that spinal cord energy metabolism imbalances may be compensated by ATP production through carnitine-aided transport and oxidation of long-chain fatty acids^24^. Several DLMs, including *N*-acetylneuraminic acid, mannose, isovalerylcarnitine, α-ketoglutaric acid, quinolinic acid and uric acid, were observed in both the spinal cord and CSF (Fig. 1e,g). Enhanced CSF uric acid during EAE is consistent with previous work^25^.

We detected 11 and 5 homeostasis-linked metabolites (HLMs) whose trajectories at recovery resembled the healthy spinal cord and CSF, respectively, with no overlap between tissues (Fig. 1f,g). Although the DLM reduced glutathione was unchanged between peak disease and recovery, indicating that resolving oxidative stress is important during EAE, metabolites protective against oxidative stress (nicotinamide and nicotinic acid)^26^ and decreased at peak disease were elevated to homeostatic healthy levels at recovery (Fig. 1e,f). Recovery-linked metabolites (RLMs) were defined as specifically exhibiting significant changes in recovered animals versus under both other conditions. We only detected the inactive oxidized form of glutathione^21^ (Fig. 1f), further implying attenuated spinal cord oxidative stress at recovery. In line with metabolomics and previous work^8^, CellRox analysis demonstrated substantial oxidative stress at peak disease within CD45^+^ versus CD45^−^ cells (Extended Data Fig. 1f). Compared to CD45^hi^CCR2^−^CX3CR1^+^ cells, CD45^hi^CCR2^+^CX3CR1^+^ cells displayed more CellRox, suggesting that monocyte infiltration promotes neurotoxicity (Fig. 1h).

These findings suggest that monocyte infiltration, an established EAE severity driver^6,7^, is associated with substantial alterations of the CNS metabolic landscape linked to neurotoxicity, perturbations in energy metabolism and redox homeostasis. Accordingly, several metabolites linked to glutathione-mediated protection against oxidative stress (*S*-adenosyl-methionine, *S*-adenosyl-homocysteine and methylnicotinamide)^27,28^ and glycolysis (glucose-6-phosphate, fructose-6-phosphate and pyruvate)^10^ did not pass the double threshold required for classification as strong metabolites and were classified as weak metabolites. Notably, compared to healthy animals, most were elevated in both tissues at peak disease and reminiscent of DLMs (Extended Data Fig. 2c,d).

### Arginine catabolism is accompanied by ARG1^+^iNOS^−^ Mdc infiltration

**Fig. 2:**
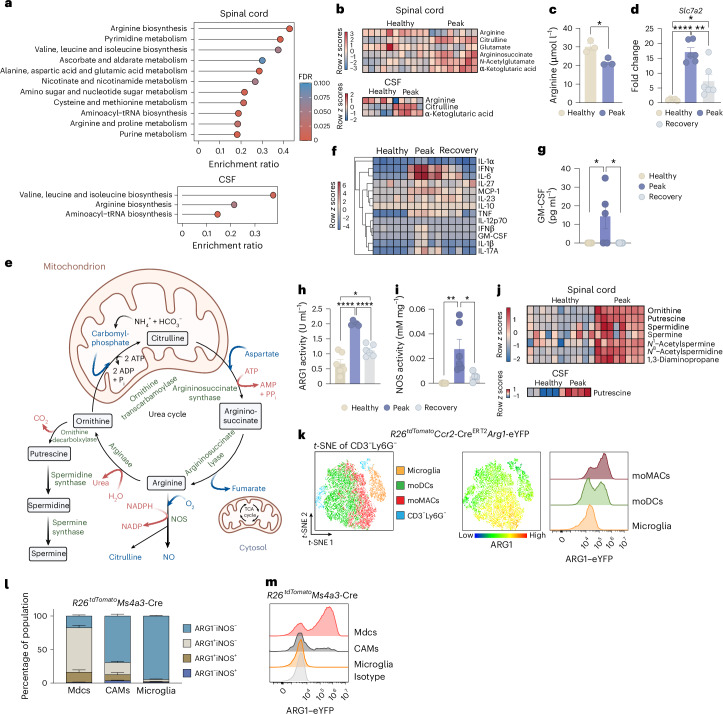
ARG1^+^ Mdcs perturb CNS arginine metabolism at peak neuroinflammation. **a**, Metabolite set enrichment analysis of significantly changed spinal cord and CSF metabolites between healthy animals and animals at peak disease. The enrichment ratio represents the hit number within a pathway divided by pathway size; spinal cord, *n* = 11 (healthy) and *n* = 8 (peak disease) animals; CSF, *n* = 5 (healthy) and *n* = 5 (peak disease) animals; FDR, false discovery rate. **b**, Metabolite heat maps of the arginine biosynthesis pathway in **a**. **c**, CSF arginine measured by ELISA; *n* = 3 animals per group; *P = 0.018. **d**, Spinal cord *Slc7a2* mRNA levels; *n* = 5 (healthy) and 6 (peak/recovery) animals; ANOVA ****P < 0.001; *P = 0.0403; **P = 0.0019; ****P < 0.0001. **e**, Urea cycle schematic; enzymes are in green. Schematic created in BioRender; Kieler, M. https://biorender.com/b42nb9u (2026); P_i_, inorganic phosphate; PP_i_, pyrophosphate; TCA, tricarboxylic acid. **f**, CSF cytokines in indicated C57BL/6J animals. Rows depict *z* scores; *n* = 5 per group. **g**, Quantification of CSF GM-CSF in **f**; *n* = 5 animals per group; ANOVA *P = 0.0315; *P = 0.0384. **h**, Spinal cord ARG1 activity; *n* = 6 animals per group; ANOVA ****P < 0.0001; *P = 0.019; ****P < 0.0001. **i**, Spinal cord NOS activity; *n* = 5 (healthy), *n* = 6 (peak) and *n* = 4 (recovery) animals; ANOVA **P = 0.0088; *P = 0.0479; **P = 0.0099. **j**, Heat maps of polyamine biosynthesis metabolites in identical animals as those in **a**. All metabolites except spermine significantly changed (*P* < 0.01). **k**, Plots depicting ARG1 in myeloid cell populations at peak disease in *R26*^*tdTomato*^*Ccr2*-Cre^ER^^T2^*Arg1*-eYFP animals. Populations were overlaid using the gating depicted in Extended Data Fig. 4a. **l**, Percentage of ARG1^−^/ARG1^+^ and iNOS^−^/iNOS^+^ cells in Mdcs (MS4A3–tdTomato^+^CX3CR1^+^), CAMs/cDC2s (MS4A3–tdTomato^−^CX3CR1^+^P2RY12^−^) and microglia (MS4A3–tdTomato^−^CX3CR1^+^P2RY12^+^) in *R26*^*tdTomato*^*Ms4a3*-Cre animals at peak disease; *n* = 5. **m**, Representative histograms depicting ARG1 expression in myeloid cell populations at peak disease in *R26*^*tdTomato*^*Ms4a3*-Cre animals using the gating depicted in Extended Data Fig. 4f. Results are shown as mean ± s.e.m. (**c**,**d**,**g**–**i**,**l**). Data in **h** are representative results from two independent experiments. Data were analyzed by one-way ANOVA followed by a Dunnett’s test (**g**), Tukey’s multiple-comparisons post-test (**d**,**f**,**h**,**i**) or two-tailed unpaired *t*-test (**c**) or Student’s *t*-test (**b**,**j**). Cells were pregated on single viable CD45^+^CD11b^+^Ly6G^−^CD3^−^ (**l**). Source data

Metabolic pathway enrichment analysis of significant spinal cord metabolites at peak disease versus in healthy animals (including both strong and weak metabolites) demonstrated enrichment of metabolites related to arginine and branched-chain amino acid biosynthesis, as well as nucleotide and nicotinate/nicotinamide metabolism (Fig. 2a). Enrichment in arginine biosynthesis was present in both tissues (Fig. 2a). Examination of individual spinal cord metabolites within the arginine biosynthesis pathway revealed decreased arginine and increased citrulline and argininosuccinate (Fig. 2b). Decreased extracellular arginine in mouse CSF at peak disease was verified by enzyme-linked immunosorbent assay (ELISA) and is in agreement with rats with EAE^29^ (Fig. 2c); further, it was associated with upregulation of the myeloid-expressed arginine transporter *Slc7a2* (ref. ^30^; Fig. 2d).

**Extended Data Fig. 3:**
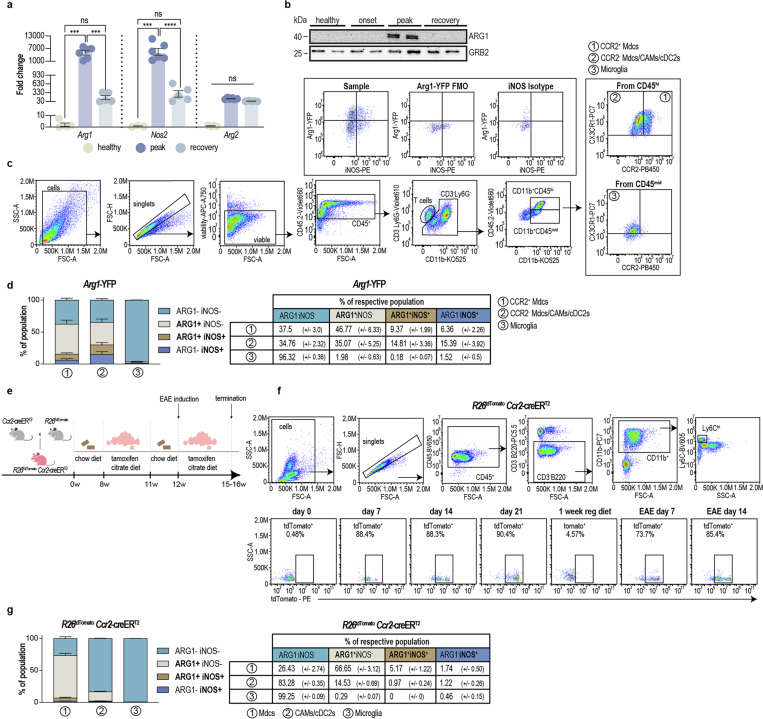
Experimental setup, gating strategy and characterization of ARG1/iNOS kinetics during neuroinflammation in Arg1-eYFP and R26tdTomato Ccr2-creERT2 animals. **a**, Spinal cord (SC) *Arg1, Arg2, Nos2* mRNA levels. n = 5 (healthy/recovery) or 6 (peak) animals. ***P = 0.0001, ***P = 0.0002, ***P = 0.0002, ****P < 0.0001. **b**, SC ARG1 protein levels at the indicated disease phases. n = 2 animals per condition. Clinical scores are onset: 0; peak: 3.5, 3.75; recovery: 1.75, 1.5 (with a previous score ≥ 3 for both animals). **c**, Gating strategy for ARG1 and iNOS expression for (**d**). Myeloid cells were gated as either CD45^hi^CCR2^+^CX3CR1^+^ for CCR2^+^ Mdcs (population 1), CD45^hi^CCR2^−^CX3CR1^+^ for CCR2^−^ Mdcs/CAMs/cDC2s (population 2) or CD45^mid^CCR2^−^CX3CR1^+^ for microglia (population 3). For ARG1 and iNOS percentages within each population in SC of *Arg1*-eYFP animals, cells then gated for ARG1-YFP or iNOS, FMOs are depicted. **d**, Percentage ARG1 and iNOS expression in CCR2^+^ Mdcs, CCR2^−^ Mdcs/CAMs/cDC2s and microglia of *Arg1*-eYFP animals at the peak-disease identified in (**c**). n = 5 animals. **e**, Genetic makeup and experimental scheme for tamoxifen feeding of *R26*^tdTomato^ *Ccr2*-creER^T2^ animals. Graphics created in BioRender; Kieler, M. https://biorender.com/zgorj77 (2026). **f**, Representative flow cytometry plots depicting percent tdTomato^+^ cells in Ly6C^hi^ peripheral blood monocytes post tamoxifen feeding and in EAE. **g**, Percentage ARG1 and iNOS expression in Mdcs (CD45^hi^CCR2-tdTomato^+^CX3CR1^+^), CAMs/cDC2s (CD45^hi^CCR2-tdTomato^−^CX3CR1^+^) and microglia (CD45^mid^CCR2-tdTomato^−^CX3CR1^+^) of *R26*^tdTomato^ *Ccr2*-creER^T2^ animals at peak-disease. n = 4 animals. Results are mean ± SEM (**a,d,g**). Statistical analysis performed with two-way ANOVA followed by Tukey multiple-comparisons post-test (**a**). Cells pre-gated on single, viable, Ly6G^−^CD3^−^CD11b^+^ (**c**,**d**,**g**). Source data

This finding coincided with dramatic transcriptional increases of spinal cord urea cycle components, notably the genes encoding the arginine-catabolizing enzymes ARG1 and inducible NOS (iNOS, refs. ^10,31^ Fig. 2e and Extended Data Fig. 3a). Robust *Arg1* versus *Arg2* expression is consistent with previous studies^32^. Western blotting verified ARG1 EAE kinetics, additionally demonstrating none at disease onset (Extended Data Fig. 3b). *Arg1* and *Nos2* expression during EAE paralleled CSF cytokines linked to monocyte influx and proinflammation (MCP-1 and TNF), as well as Mdc effector function (IFNγ and GM-CSF^9,33^; Fig. 2f,g). Further, ARG1 and NOS activity were elevated in the spinal cord at peak disease, and although postrecovery activity of both decreased, ARG1 activity remained modestly increased compared to in healthy mice (Fig. 2h,i). This finding, together with citrulline accumulation at peak EAE, which is derived from iNOS-mediated arginine catabolism (Fig. 2b,e), suggests that ARG1- or iNOS-expressing Mdcs contribute to arginine consumption at peak disease. Subsequent examination of polyamines downstream of ARG1 revealed that ornithine, putrescine and spermidine were elevated in the spinal cord, indicating a predominant role for ARG1. Further, consistent with rats with EAE, putrescine was elevated in the CSF (Fig. 2j and Supplementary Table 6)^29^.

Examining ARG1 and iNOS kinetics within Mdcs at peak disease, we next used animals expressing enhanced yellow fluorescent protein (eYFP) under the control of the *Arg1* promoter (*Arg1*-eYFP animals) and stained for iNOS within spinal cord-infiltrating CD11b^+^CD45^hi^CCR2^+^CX3CR1^+^ cells (Extended Data Fig. 3c). Roughly half of infiltrating Mdcs were selectively ARG1^+^ (ARG1^+^iNOS^−^), whereas only 6–10% were selectively iNOS^+^ (ARG1^−^iNOS^+^) or ARG1^+^iNOS^+^. Further, although CD11b^+^CD45^mid^CCR2^−^CX3CR1^+^ microglia exhibited minimal ARG1, CD11b^+^CD45^hi^CCR2^−^CX3CR1^+^ cells were also largely ARG1^+^iNOS^−^ (Extended Data Fig. 3d). This latter population likely represents a mixture of Mdcs that have lost CCR2, type 2 conventional DCs (cDC2s) or CAMs.

Knowing that CCR2 is downregulated on Ly6C^hi^ monocytes after CNS entry^3,16^, we next used fate mapping to detect ARG1 and iNOS on Mdcs even if they no longer express *Ccr2*. We generated *R26*^*tdTomato*^*Ccr2*-Ere^ER^^T2^ animals that after tamoxifen treatment irreversibly label CCR2^+^ cells, including Ly6C^hi^ monocytes and their progeny^9^, fed them a tamoxifen diet for 3 weeks, rested them for 1 week to alleviate long-term tamoxifen use and examined labeling efficiency. Consistent with rapid blood monocyte turnover^34^, tdTomato expression was lost (Extended Data Fig. 3e,f). Subsequently, while inducing EAE and concomitantly reintroducing a tamoxifen diet, we next determined ARG1 and iNOS levels within spinal cord-infiltrating Mdcs. Concordant with *Arg1*-eYFP animals at peak disease, most infiltrating CD45^hi^CCR2–tdTomato^+^CX3CR1^+^ Mdcs were selectively ARG1^+^ (ARG1^+^iNOS^−^; Extended Data Fig. 3g). Because CAM populations exhibit mixed ontogeny^2^, this experimental approach additionally allowed for the identification of a minor population of long-lived or non-monocyte-derived CD45^hi^CCR2–tdTomato^−^CX3CR1^+^ cells, which could represent CAMs or cDC2s. Although this population displayed 15% ARG1^+^iNOS^−^ positivity, ARG1 was largely absent on CD45^mid^CCR2–tdTomato^−^CX3CR1^+^ microglia in this experimental setup (Extended Data Fig. 3g).

**Extended Data Fig. 4:**
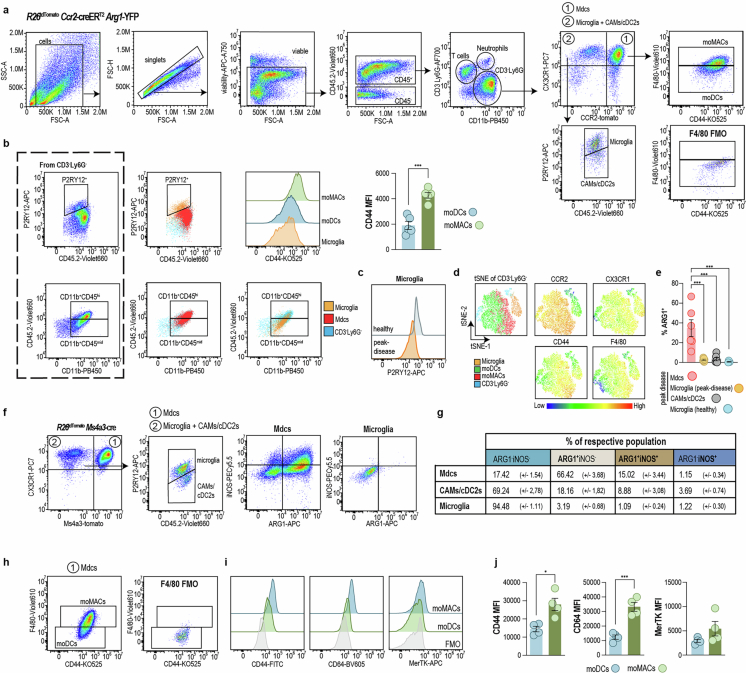
Experimental setup, gating strategy and characterization of ARG1/iNOS kinetics during neuroinflammation in *R26*^tdTomato^*Ccr2*-creER^T2^*Arg1*-eYFP and *R26*^tdTomato^ Ms4a3-cre animals. **a**, Gating strategy for myeloid cells. Cells are gated on CCR2-tdTomato and CX3CR1 to identify microglia (CCR2-tdTomato^−^CX3CR1^+^P2RY12^+^), CAMs/cDC2s (CCR2-tdTomato^−^CX3CR1^+^P2RY12^−^), Mdcs (CCR2-tdTomato^+^CX3CR1^+^). Within Mdcs, moMACs and moDCs were identified as F4/80^+^CD44^hi^ and F4/80^−^CD44^+^ cells respectively. **b**, Proof of myeloid cell subsets identified in (**a**). Microglia are P2RY12^+^CD11b^+^CD45^mid^. Mdcs are CD11b^+^CD45^hi^ and F4/80^−^ moDCs and F4/80^hi^ moMACs are CD44^+^ and CD44^hi^ respectively. n = 5 animals. ***P = 0.0003. **c**, Histograms depicting P2RY12 downregulation in microglia of animals at peak-disease versus health. Cells gated on CCR2-tdTomato^−^CX3CR1^+^. **d**, t-SNE plots as proof for myeloid cell subsets identified in (**a**), generated from CD45^+^CD3^−^Ly6G^−^ cells and manually gated microglia (orange) and moDCs (green) and moMACs (red) were overlaid using the gating strategy shown in (**a**). Parent plot is identical to Fig. 2k and shown for comparison. **e**, Percentage ARG1-expression in SC myeloid subsets of *R26*^tdTomato^*Ccr2*-creER^T2^ *Arg1*-eYFP animals at peak-disease (n = 6) and health (n = 4). ANOVA ***P = 0.0001. ***P = 0.0009 (Mdcs vs microglia (health), 0.0005 (Mdcs vs microglia (peak-disease), 0.0006 (Mdcs vs CAMs/cDC2s). Cells were gated as in (**a**). **f**, Gating strategy for myeloid cells in *R26*^tdTomato^ Ms4a3-cre animals. Cells gated using MS4A3-tdTomato and CX3CR1 to identify microglia (MS4A3-tdTomato^−^CX3CR1^+^P2RY12^+^), CAMs/cDC2s (MS4A3-tdTomato^−^CX3CR1^+^P2RY12^−^), Mdcs (MS4A3-tdTomato^+^CX3CR1^+^). **g**, Quantification ARG1 and iNOS expression within Mdcs and microglia identified in (**f**) in spinal cords of *R26*^tdTomato^ Ms4a3-cre animals. n = 5 animals. **h**, Mdcs, moMACs and moDCs identified as F4/80^+^CD44^hi^ and F4/80^−^CD44^+^ cells respectively from (**f**). **i/j**, Representative histograms and quantification depicting CD44, CD64 and MerTK expression on moMACs and moDCs. n = 4 animals. *P = 0.011, ***P = 0.0008. Results are mean ± SEM (**e,g,j**). Statistical analysis performed with one-way ANOVA followed by Tukey multiple-comparisons post-test (**e**) or two-tailed unpaired t-test (**b,j**). Cells pre-gated as single, viable, CD45^+^Ly6G^−^CD3^−^CD11b^+^ (**a,f**). Source data

Further characterizing infiltrating ARG1^+^ Mdcs, we next crossed *Arg1*-eYFP with *R26*^*tdTomato*^*Ccr2*-Cre^ER^^T2^ animals, generating *R26*^*tdTomato*^*Ccr2*-Cre^ER^^T2^*Arg1*-eYFP animals. This together with an expanded myeloid cell flow cytometry panel revealed infiltrating CD11b^+^CD45^hi^CCR2–tdTomato^+^CX3CR1^+^ Mdcs composed of both F4/80^+^CD44^hi^ moMACs and F4/80^−^CD44^+^ moDCs^5,9^, also corroborating that CD11b^+^CD45^mid^CCR2–tdTomato^−^CX3CR1^+^ microglia expressed the microglial marker P2RY12 and were CD11b^+^CD45^mid^CD44^lo^^4,5,9^ (Extended Data Fig. 4a,b). Consistent with P2RY12 downregulation in EAE^4^ relative to microglia from healthy animals, CD11b^+^CD45^mid^CCR2–tdTomato^−^CX3CR1^+^ microglia at peak disease expressed lower P2RY12 (Extended Data Fig. 4c). Unsupervised *t*-distributed stochastic neighbor embedding (*t*-SNE)-guided analysis confirmed extensive ARG1 within CD44^+^CCR2–tdTomato^+^ Mdcs with manual gating, demonstrating the highest levels in moMACs (Fig. 2k). Interestingly, consistent with the postulated stepwise differentiation of CNS-infiltrating monocytes into moDCs and subsequently ARG1-expressing moMACs^9^, as monocytes differentiated into Mdcs at peak disease, they concomitantly acquired ARG1, CD44 and F4/80, with CD44 and ARG1 being higher in moMACs than in moDCs (Fig. 2k and Extended Data Fig. 4d). Importantly, compared to CD11b^+^CD45^+^CCR2–tdTomato^−^CX3CR1^+^P2RY12^−^ CAMs/cDC2s and CD11b^+^CD45^+^CCR2–tdTomato^−^CX3CR1^+^P2RY12^+^ microglia, Mdcs exhibited the most ARG1 (Extended Data Fig. 4e).

To alleviate tamoxifen use in *R26*^*tdTomato*^*Ccr2*-Cre^ER^^T2^*Arg1*-eYFP animals, we next used *R26*^*tdTomato*^*Ms4a3*-Cre animals that efficiently fate map progeny of granulocyte–monocyte progenitors^35^. This confirmed that CD11b^+^CD45^+^MS4A3–tdTomato^+^CX3CR1^+^ Mdcs were mainly ARG1^+^iNOS^−^ at peak disease and that microglia exhibited marginal ARG1, with similar percentages obtained to *R26*^*tdTomato*^*Ccr2*-Cre^ER^^T2^ animals (Fig. 2l,m and Extended Data Figs. 3g and 4f,g). F4/80^+^ moMACs exhibited elevated CD44 and CD64 levels^9^ compared to F4/80^−^ moDCs (Extended Data Fig. 4h–j), demonstrating fidelity of moMAC and moDC populations. We noted comparable ARG1 expression in non-monocyte-derived CD11b^+^CD45^+^MS4A3–tdTomato^−^CX3CR1^+^P2RY12^−^ cells that could comprise embryonic CAMs or cDC2s to percentages obtained in *R26*^*tdTomato*^*Ccr2*-Cre^ER^^T2^ animals (Fig. 2l,m and Extended Data Figs. 3g and 4f,g). Together, arginine catabolism at peak disease within the CNS is accompanied by prominent ARG1^+^iNOS^−^ Mdc infiltration.

### Arginine catabolism localizes in lesions with ARG1^+^ myeloid cells

**Fig. 3:**
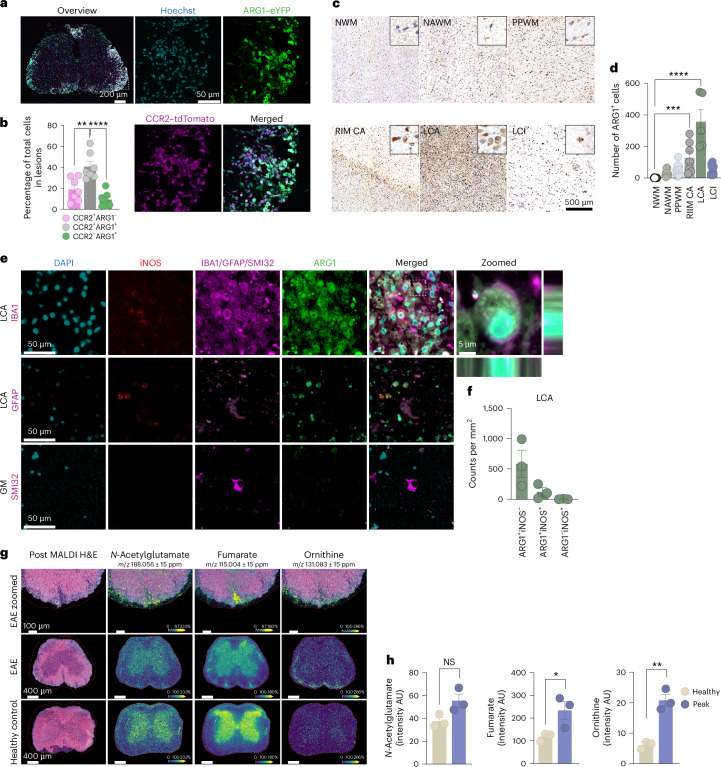
Human MS and mouse EAE lesions are characterized by ARG1^+^ myeloid cells and arginine catabolism. **a**, Representative immunofluorescence images of spinal cord sections of *R26*^*tdTomato*^*Ccr2*-Cre^ER^^T2^*Arg1*-eYFP animals at peak disease. Magnification of the lesion center (×40) is shown. **b**, Quantification of **a**; *n* = 7 sections; ANOVA ****P < 0.0001; **P = 0.003; ****P < 0.0001. **c**, Representative immunohistochemical ARG1 staining of brain tissue from individuals with MS and healthy control brain tissue; NWM, normal white matter; NAWM, normal-appearing white matter (MS myelinated WM); PPWM, periplaque white matter; RIM CA, rim of the active center; LCA, lesion center active; LCI, lesion center inactive. **d**, Quantification of **c**; *n* = 8 (normal white matter), *n* = 13 (normal-appearing white matter), *n* = 18 (periplaque white matter), *n* = 13 (lesion center inactive), *n* = 7 (rim of the active center), *n* = 5 (lesion center active) individuals with MS; ANOVA ****P < 0.0001; ***P = 0.0004; ****P < 0.0001. **e**, Representative immunofluorescence images from human MS sections; GM, gray matter. IBA1 indicates myeloid cells, GFAP indicates astrocytes, and SMI32 indicates neurons. Depicted are *z* stacks of the indicated ARG1^+^ cell. **f**, Quantification of ARG1 and iNOS single- and double-positive cells from human MS active lesion centers from **e**; *n* = 3 individuals with MS; ANOVA *P = 0.0492. **g**, Representative hematoxylin and eosin (H&E) staining and spatial distributions of indicated metabolites in spinal cords of healthy animals and animals at peak disease as determined using MSI. **h**, Quantification of **g**; *n* = 3 per experimental group; *P = 0.0478; **P = 0.0022; NS, not significant; AU, arbitrary units. Results are shown as mean ± s.e.m. (**b**,**d**,**f**,**h**). Data were analyzed by one-way ANOVA followed by a Dunnett’s test (**d**), Tukey’s multiple-comparisons post-test (**b**,**f**) or two-tailed unpaired *t*-test (**h**). Source data

**Extended Data Fig. 5:**
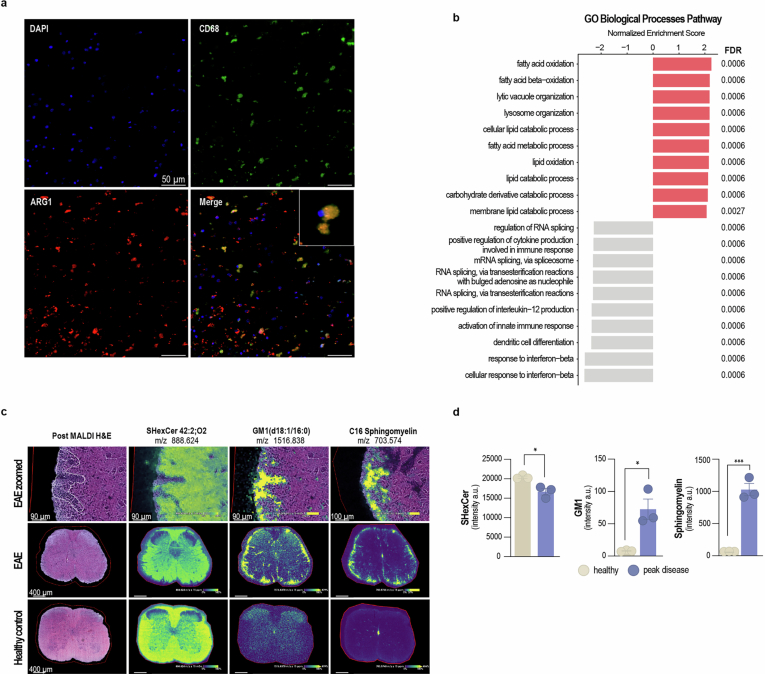
ARG1 is expressed on CD68^+^ myeloid cells in human MS lesions and engage in lipid metabolism. **a**, Immunofluorescence image of ARG1 and CD68 double staining from one typical human MS RIM CA lesion section. **b**, Top 10 up-/ downregulated GO biological pathways from GSEA of differentially expressed genes shown in Fig. 4a and their normalized enrichment scores. FDR ≤ 0.01 and is displayed on the right. **c/d**, Representative H&E staining and spatial distributions of indicated lipids in spinal cords of healthy and peak-diseased animals as determined using (MALDI)-MS imaging (MSI). Scale bar in zoomed pictures (upper panel) represents 90–100 μm and in middle/lower panels represents 400 μm. n = 3. *P = 0.0145 (GM1), 0.0146 (SHexCer), ***P = 0.0005 (sphingomyelin). Results are mean ± SEM (**d**). Statistical analysis performed with two-tailed unpaired t-test (**d**). Source data

We next examined the spatial localization of ARG1^+^ Mdcs and observed that most CCR2–tdTomato^+^ cells within *R26*^*tdTomato*^*Ccr2*-Cre^ER^^T2^*Arg1*-eYFP animals were ARG1^+^ and localized within EAE lesions (Fig. 3a). Although a small portion of ARG1^+^CCR2–tdTomato^−^ cells were present, most were ARG1^+^CCR2–tdTomato^+^ Mdcs (Fig. 3b). Translating these findings to humans, we performed ARG1 immunohistochemistry on postmortem brain autopsy samples from healthy control individuals and individuals with different clinical courses of MS (Supplementary Table 7). Individuals with MS included those exhibiting active lesions, chronic active lesions and inactive lesions^36,37^. Although little ARG1 was present in the white matter of healthy control individuals, abundance increased in individuals with MS, with the normal-appearing white matter exhibiting some ARG1^+^ cells. These cells were slightly elevated in the periplaque white matter and significantly augmented at the rim of the active center and at the active lesion center. The center of inactive and chronic active lesions was hypocellular, exhibiting sparse ARG1 (Fig. 3c,d). We noted that within the rim of chronic active lesions, ARG1 was found in CD68^+^ cells (Extended Data Fig. 5a). Immunofluorescence demonstrated that, analogous to mouse EAE, most myeloid cells in human MS lesions were ARG1^+^iNOS^−^, with *z* stacks revealing pronounced cytoplasmic localization. Further, although glial fibrillary acidic protein^+^ (GFAP^+^) astrocytes were found in active lesion centers^38^, they did not express ARG1, nor did SMI32^+^ neurons^39^ in the gray matter (Fig. 3e,f). We conclude that conserved ARG1 expression occurs in lesion-associated myeloid cells in human MS and mouse EAE.

Hypothesizing that the presence of ARG1^+^ cells would coincide with its downstream metabolites, we next used matrix-assisted laser desorption/ionization–mass spectrometry imaging (MALDI–MSI) to spatially resolve the distribution of several urea cycle metabolites^40,41^. This revealed that *m*/*z* features assigned to fumarate, *N*-acetylglutamate and ornithine were localized within lesions at peak disease, with quantification demonstrating significantly more ornithine and fumarate (Fig. 3g,h). Thus, ARG1^+^ cells are associated with arginine catabolism in lesions.

### Lipid-laden ARG1^+^ Mdcs regulate redox homeostasis via arginine

**Fig. 4:**
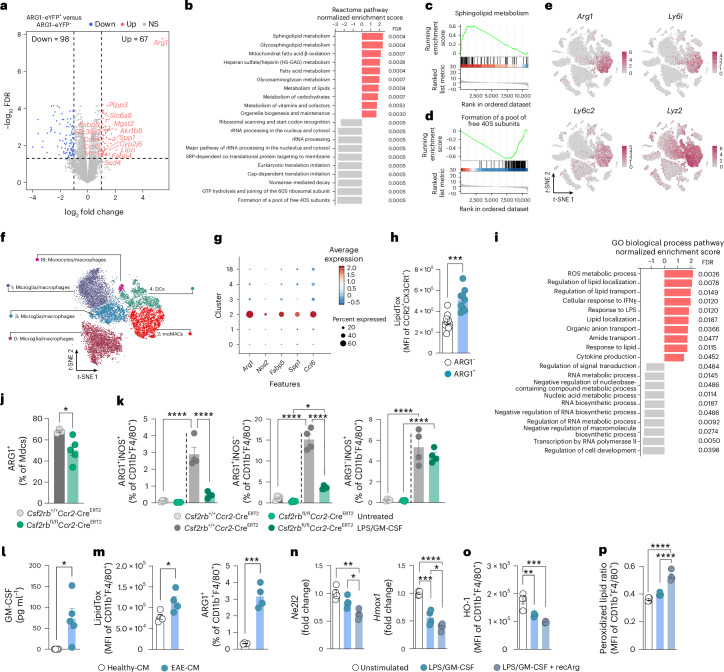
ARG1^+^ Mdcs exhibit a transcriptome reflecting their lipid-rich environment, modulating redox homeostasis via arginine. **a**, Transcriptional differences between ARG1^+^YFP^+^/ARG1^−^YFP^−^ cells at peak disease. Cells were sorted on single viable Ly6G^−^CD3^−^CD45^+^CX3CR1^+^ cells; *n* = 5 animals. **b**, Top ten upregulated/downregulated Reactome pathways from GSEA of differentially expressed genes in **a**; FDR ≤ 0.01. **c**,**d**, GSEA from **a** for sphingolipid metabolism (**c**) or formation of a pool of free 40S subunits (**d**). **e**, *t*-SNE plot showing *Arg1*, *Ly61*, *Ly6c2* and *Lyz2* during EAE in scRNA-seq data set GSE130119. **f**, Myeloid clusters from **e** (*n* = 14,224 cells). **g**, Dot plot showing expression of selected genes for cell subsets in **f**. **h**, LipidTox median fluorescence intensity (MFI) of ARG1^+^ versus ARG1^−^ CX3CR1^+^CCR2^+^ Mdcs from the spinal cord of *Arg1*-eYFP animals at peak disease; *n* = 9 animals; ***P = 0.002. **i**, Ten selected significantly upregulated/downregulated GO biological processes from GSEA of differentially expressed genes between *Slc7a2*^*hi*^ and all other myeloid cells as in Extended Data Fig. 6g; FDR ≤ 0.05. **j**, Spinal cord ARG1 expression within CX3CR1^+^CCR2^+^ Mdcs of *Csf2rb*^+/+^*Ccr2*-Cre^ER^^T2^ (*n* = 3) and *Csf2rb*^*fl/fl*^*Ccr2*-Cre^ER^^T2^ (*n* = 5) animals at peak disease; *P = 0.0417. **k**, Percentage of ARG1/iNOS in LPS/GM-CSF-treated BMDMs of *Csf2rb*^+/+^*Ccr2*-Cre^ER^^T2^ (*n* = 4) and *Csf2rb*^*fl/fl*^*Ccr2*-Cre^ER^^T2^ (*n* = 4) animals after 48 h; ANOVA ****P < 0.0001; *P = 0.0178; ****P < 0.0001. **l**, GM-CSF in healthy-CM or EAE-CM; *n* = 5 samples; *P = 0.0158. **m**, LipidTox MFI and ARG1 frequency of treated BMDMs; *n* = 4 samples; *P = 0.0409; ***P = 0.002. **n**, *Ne2l2* and *Hmox1* in treated BMDMs; *n* = 4 animals per group; ANOVA **P = 0.0015 (*Ne2l2*); ****P < 0.0001 (*Hmox1*); *P = 0.0321 (LPS/GM-CSF versus LPS/GM-CSF + recArg (*Ne2l2*)) or *P = 0.0448 (LPS/GM-CSF versus LPS/GM-CSF (*Hmox1*)); **P = 0.0011 (untreated versus LPS/GM-CSF + recArg (*Ne2l2*)); ***P = 0.0001 (untreated versus LPS/GM-CSF (*Hmox1*)); ****P < 0.0001. **o**, HO-1 MFI in treated BMDMs; *n* = 4 per condition; ANOVA ***P = 0.0002; **P = 0.0024; ***P = 0.0001. **p**, Lipid peroxidation of BMDMs using C11-BODIPY; *n* = 4 per condition; ANOVA ****P < 0.0001; ****P < 0.0001. Results are shown as mean ± s.e.m. (**h**,**j**–**p**). Representative results from two (**l**–**o**) or three independent experiments (**p**) are shown. Data were analyzed by one-way ANOVA followed by a Tukey’s multiple-comparisons post-test (**k**,**m**–**p**) or two-tailed unpaired *t*-test (**h**,**j**,**l**,**m**). BMDMs were pregated on single viable CD11b^+^F4/80^+^ cells (**k**,**m**,**o**,**p**) or Mdcs on Ly6G^−^CD3^−^CD11b^+^CD45^hi^ cells (**h** and **j**). Source data

To examine how ARG1 contributes to the transcription of lesion-associated Mdcs, we next performed bulk RNA sequencing (RNA-seq) on sorted viable Ly6G^−^CD3^−^CD45^+^CX3CR1^+^YFP^+^ versus viable Ly6G^−^CD3^−^CD45^+^CX3CR1^+^YFP^−^ cells from *Arg1*-eYFP animals with EAE at peak disease. Although this experimental setup does not precisely differentiate between CAMs and Mdcs, we identified 67 and 98 up- and downregulated genes, respectively (Fig. 4a). Although no M2 markers of polarization were enriched in ARG1^+^ cells except for *Mrc1*, genes more than twofold upregulated included those involved in fatty acid metabolism (*Fabp4*, *Fabp5* and *Scd4*)^42^, lipid catabolism and uptake of bioactive lipids (*Lipn, Plpp3* and *Cyp2j6*; Fig. 4a). Further, genes expressed by CAMs and microglia implicated in demyelination (*Spp1* and *Ccl6*) were elevated in ARG1^+^ cells^43^. Gene set enrichment analysis (GSEA) based on the Reactome database revealed enrichment in metabolism of diverse lipids ranging from sphingolipids and glycosphingolipids to fatty acids (Fig. 4b,c). GSEA based on Gene Ontology (GO; biological processes) ratified engagement of ARG1^+^ cells in lipid metabolic processes such as fatty acid oxidation and catabolism (Extended Data Fig. 5b). Downregulated gene sets in ARG1^+^ cells were associated with translation and 40S ribosomes (Fig. 4b,d), possibly reflecting localized impacts of extracellular arginine withdrawal within the CNS by ARG1^+^ Mdcs (Fig. 2) and its reported impacts on global translation through ribosome pausing^44^.

**Extended Data Fig. 6:**
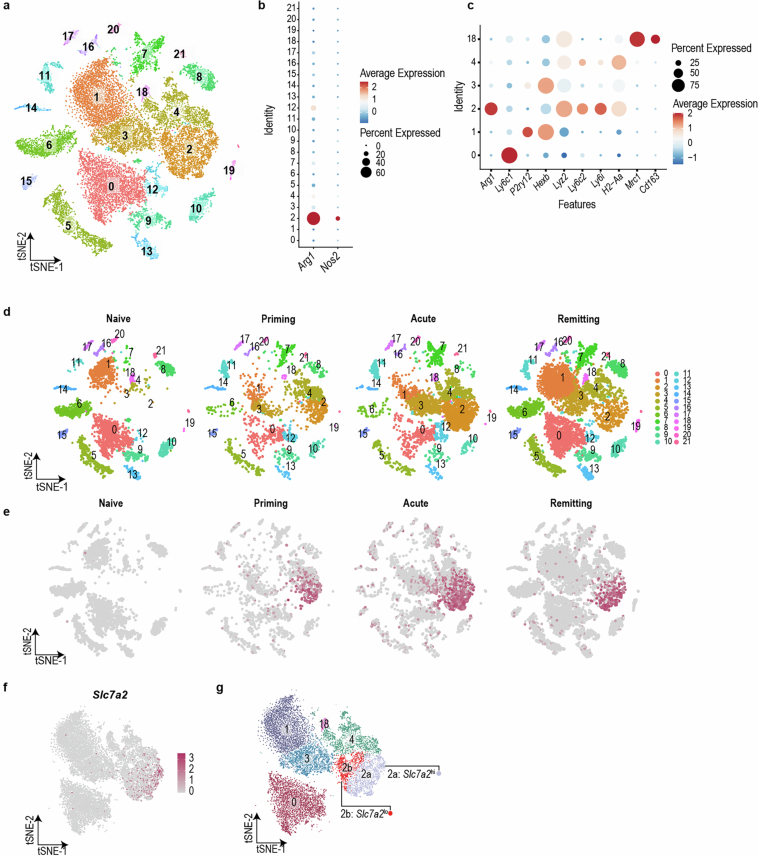
t-SNE plots and subclustering of disease associated populations in EAE. **a**, Unsupervised clustering t-distributed stochastic neighbor embedding (t-SNE) plot showing spinal cord cellular composition during EAE among all CNS cells subjected to scRNA-seq (24,275 cells) from data set GSE130119. Coordinates, metadata and annotations were kept the same as the original publication. 0, MG/MΦ; 1, MG/MΦ; 2, moMACs; 3, MG/MΦ; 4, Dendritic cells; 5, Ependymal cells; 6, Oligodendrocytes; 7, T cells; 8, Astrocytes; 9, Pericytes; 10, Astrocytes; 11, Neutrophils; 12, Endothelial cells; 13, Sm. Muscle cells; 14, Astrocytes; 15, Endothelial cells; 16, Neurons; 17, Epithelial cells; 18, Monocytes/MΦ; 19, Epithelial cells; 20, Stromal cells; 21, Neurons. MG, microglia; MΦ, macrophages. **b**, Dot plot showing *Arg1* and *Nos2* expression in all clusters from (**a**). **c**, Dot plot showing expression of selected markers in myeloid cell clusters from (**a**). **d**, t-SNE plot showing spinal cord cellular composition during the different EAE phases subjected to scRNA-seq (naïve: 4185 cells; priming: 2746 cells; acute: 6087 cells; recovery/remitting; 9214 cells) from data set GSE130119. Coordinates, metadata and annotations were kept the same as the original publication. **e**, t-SNE plot showing *Arg1*-expression during the different EAE phases in (**d**). **f**, t-SNE plot illustrating myeloid clusters from (**a**) depicting *Slc7a2* expression (n = 14,224 cells). **g**, t-SNE plot as in (**f**) with sub-clustering of *Slc7a2*^hi^ and *Slc7a2*^lo^ cells. Source data

To gain insights into ARG1^+^ Mdcs at a single-cell level, we next used a publicly available single-cell RNA-seq (scRNA-seq) data set encompassing animals at various EAE stages, which identified multiple disease-associated cell populations, including five myeloid clusters^45^ (Extended Data Fig. 6a). This revealed predominant and specific *Arg1* in a cluster (cluster 2) rich in typical monocyte and macrophage markers, including *Ly6i*, *Ly6c2* and *Lyz2*(refs. ^4,5^; Fig. 4e and Extended Data Fig. 6b). Specific analysis of myeloid cell clusters revealed that clusters 1 and 3 expressed typical microglial markers *P2ry12* and *Hexb*, whereas cluster 18 expressed CAM-identifying genes, *Mrc1* and *Cd163*(refs. ^1,4^; Fig. 4f and Extended Data Fig. 6c). Stratification into the different EAE phases demonstrated robust *Arg1* at peak disease (Extended Data Fig. 6d,e). Consistent with bulk RNA-seq, the *Arg1*^+^ cluster strongly expressed lipid- and demyelination-associated genes versus other myeloid clusters (Fig. 4a,f,g). Accordingly, MALDI–MSI demonstrated dynamic white matter lipid remodeling during peak disease. Herein, myelin-derived lipids, including ganglioside GM1 (d18:1/16:0) and sphingomyelin (d18:1/16:0), accumulated, whereas very long-chain sulfatides species (SHexCer 42:2;O2), important for myelin compaction, stabilization and function, decreased specifically within lesions^46,47^ (Extended Data Fig. 5c,d). Further, LipidTox flow cytometry demonstrated that ARG1^+^ Mdcs were more lipid laden (Fig. 4h). Thus, the transcriptome of ARG1^+^ Mdcs during peak EAE reflects their response to diverse myelin-derived lipids accumulating during demyelination^46,47^. These data further suggest that ARG1^+^ Mdcs are reminiscent of neurodegeneration-promoting lipid droplet-accumulating microglia (LDAM)^48^.

**Extended Data Fig. 7:**
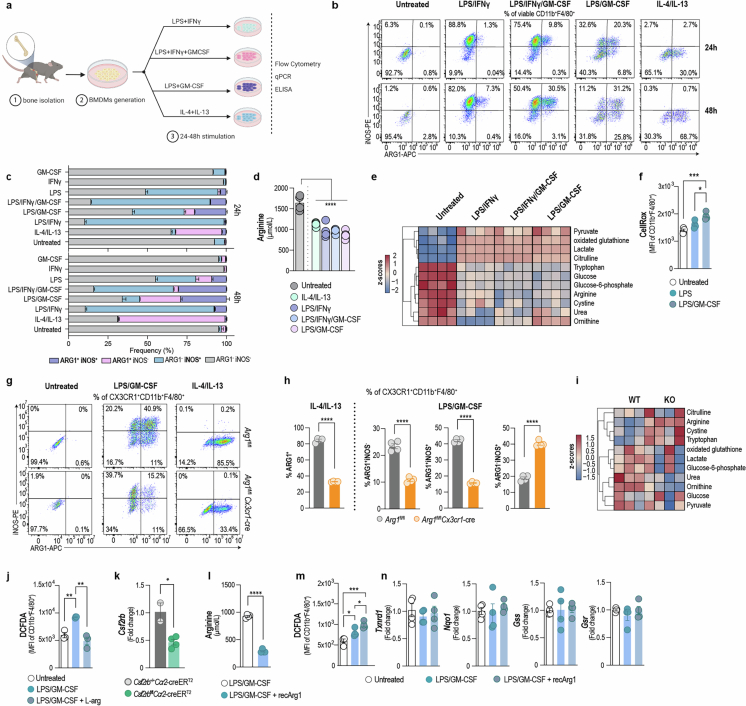
GM-CSF promotes ARG1 mediated arginine catabolism, enhancing oxidative stress. **a**, Scheme examining how GM-CSF and IFN-γ impacts macrophage ARG1/iNOS post LPS. Schematic created in BioRender; Kieler, M. https://biorender.com/2yw4ydi (2026). **b/c**, Representative flow cytometry plots and quantification depicting percentage ARG1/iNOS in treated-BMDMs of C57BL/6J animals. n = 4 samples per condition. Statistical matrix in Supplementary Table 8. **d**, Arginine levels in SN of the indicated conditions. n = 4 (untreated), 5 (treatments) per condition. ANOVA ****P < 0.0001. ****P < 0.0001. **e**, Extracellular metabolites of treated-BMDMs from C57BL/6J animals for 48 h. Rows depict z-scores with significant metabolites (FDR < 0.05). n = 4 samples per condition. **f**, CellRox MFI of treated-BMDMs. n = 4 samples per condition. ANOVA ***P = 0.0005. *P = 0.0119, ***P = 0.0004. **g/h**, Representative flow cytometry plots and quantification depicting ARG1/iNOS in treated-BMDMs of *Arg1*^fl/fl^ (n = 4) and *Arg1*^fl/fl^ *Cx3cr1*-cre (n = 4) animals after 48 h. ****P < 0.0001. **i**, Extracellular metabolites of LPS/GM-CSF treated-BMDMs from *Arg1*^fl/fl^ (WT, n = 4) and *Arg1*^fl/fl^ *Cx3cr1*-cre (KO, n = 3) after 48 h. Rows depict z-scores of identical metabolites indicated in (**e**). **j**, Dichlorodihydrofluorescein Diacetate (DCFDA) MFI of treated-BMDMs. L-arg, L-arginine. n = 3 samples per condition. ANOVA **P = 0.0019. **P = 0.0075 (untreated vs LPS/GM-CSF) and 0.0019 (LPS/GM-CSF vs LPS/GM-CSF + L-arg). **k**, *Csf2rb* within CX3CR1^+^CCR2^+^ Mdcs from spinal cords of *Csf2rb*^+/+^ *Ccr2*-creER^T2^ (n = 2) and *Csf2rb*^fl/fl^ *Ccr2*-creER^T2^ (n = 4) animals at peak-disease. *P = 0.0128. Cells sorted for single viable CD45^+^, then Ly6G^−^CD3^−^CD11b^+^Ly6C^+^CX3CR1^+^. **l**, Arginine in SN of treated-BMDMs. n = 4 samples per condition. ****P < 0.0001. **m**, DCFDA MFI of treated-BMDMs. recArg1, recombinant Arginase 1. n = 4 samples per condition. ANOVA ***P = 0.0007. *P = 0.0331 (untreated vs LG) = 0.0349 (LG vs LG + recArg), ***P = 0.0005. **n**, *Txnrd1, Nqo1, Gss* and *Gsr* levels of treated-BMDMs. n = 4 samples per condition. Results are mean ± SEM (**b,d,f,h,j,k,l,m,n**). Representative results from 2 (**b,c,g,h,m,n**) or 3 (**j**) independent experiments. Statistical analysis performed with two-way ANOVA followed by Tukey multiple-comparisons post-test (**d**), two-tailed unpaired t-test (**k,l**) or Benjamini-Hochberg (**e,i**). Cells were pre-gated on single viable CD11b^+^F4/80^+^ (**b,c,f,j,m**) or CX3CR1^+^CD11b^+^F4/80^+^ (**g,h**). Source data

Knowing that *Slc7a2* is upregulated at peak disease (Fig. 2d), we examined if this arginine transporter^30^ is coexpressed by *Arg1*^+^ Mdcs and observed some expression within the moMACs cluster (Extended Data Fig. 6f). To uncover how environmental arginine availability impacts myeloid cellular responses, we next subclustered *Slc7a2*^*hi*^-expressing moMACs and performed GSEA versus all other myeloid clusters (Extended Data Fig. 6g). Consistent with the notion that *Arg1*^+^ moMACs resemble LDAM, characterized by lipid droplet formation, high reactive oxygen species (ROS) production and proinflammation triggered by the endotoxin lipopolysaccharide (LPS)^48^, *Slc7a2*^*hi*^ and *Arg1*-coexpressing moMACs exhibited enrichment in biological processes associated with lipids, ROS and responses to LPS (Fig. 4i). As EAE immunopathology is promoted by GM-CSF, which positively impacts *Arg1* expression^9,49^, and responses to IFNγ were enriched in *Slc7a2*^*hi*^ and *Arg1*-coexpressing moMACs (Fig. 4i), we next examined how GM-CSF impacts macrophage ARG1/iNOS expression by treating bone marrow-derived macrophages (BMDMs) with LPS and boosting inflammatory responses with either IFNγ and/or GM-CSF. Additionally, we used interleukin-4/interleukin-13 (IL-4/IL-13), a well-established ARG1 inducer^31^ (Extended Data Fig. 7a). Although LPS/IFNγ or IL-4/IL-13 led to either iNOS or ARG1 production, and GM-CSF or IFNγ alone had no impact, simultaneous LPS/IFNγ/GM-CSF stimulation resulted in a shift from iNOS^+^ARG1^−^ to iNOS^+^ARG1^+^ expression. This effect was prominent in the absence of IFNγ, with LPS/GM-CSF-treated cells exhibiting a clear temporal shift from an iNOS^+^ARG1^+^ to an iNOS^−^ARG1^+^ signature (Extended Data Fig. 7b,c). Metabolic characterization of the aforementioned conditions in the presence of LPS demonstrated extracellular arginine consumption, which was verified using ELISA (Extended Data Fig. 7d,e). Notably, LPS/GM-CSF-treated cells that selectively exhibited high ARG1 catabolized arginine via ARG1 as demonstrated by their exclusive extracellular ornithine and urea accumulation (Extended Data Fig. 7e). Simultaneously, relative to LPS alone, LPS/GM-CSF caused enhanced ROS generation (Extended Data Fig. 7f).

Examining contributions of ARG1-instigated arginine catabolism therein, we next generated *Arg1*^*fl/fl*^*Cx3cr1*-Cre animals by crossing *Arg1*^*fl/fl*^ mice with animals constitutively expressing Cre recombinase under the control of the *Cx3cr1* promoter, generating BMDMs from both genotypes. Compared to controls, *Arg1* was efficiently decreased in *Arg1*^*fl/fl*^*Cx3cr1*-Cre IL-4/IL-13- and LPS/GM-CSF-stimulated macrophages, the latter of which was notably associated with extracellular arginine accumulation and impairments in ARG1-generated urea and ornithine (Extended Data Fig. 7g–i and Supplementary Fig. 1). Although after LPS/GM-CSF arginine abundance remained high within ARG1-deficient macrophages, citrulline levels were unchanged relative to controls, suggesting that arginine catabolism via iNOS insignificantly contributes to their increased ROS state (Extended Data Fig. 7i). Instead, indicative that ARG1-mediated extracellular arginine consumption heightens oxidative stress, and, consistent with the antioxidant effects of L-arginine^50,51^, LPS/GM-CSF-induced ROS was attenuated by L-arginine supplementation (Extended Data Fig. 7j). Together, these data assign a role for ARG1-mediated arginine catabolism downstream of the GM-CSF receptor to the ability of GM-CSF to promote ROS production within CNS-associated Mdcs^9,49^. To strengthen a contribution of GM-CSF to ARG1-mediated arginine catabolism in Mdcs during EAE, we next used *Csf2rb*^*fl/fl*^*Ccr2*-Cre^ER^^T2^ animals^49^. Sorted Mdcs from these animals exhibited attenuated *Csf2rb* expression, confirming efficient recombination in the *Csf2rb* locus after tamoxifen treatment, with flow cytometry demonstrating significant, albeit modest, ARG1 reduction (Fig. 4j and Extended Data Fig. 7k). Further, BMDMs isolated from *Csf2rb*^*fl/fl*^*Ccr2*-Cre^ER^^T2^ animals and administered 4-hydroxytamoxifen to delete *Csf2rb* displayed attenuated ARG1^+^ signatures after LPS/GM-CSF treatment relative to control animals (Fig. 4k).

**Extended Data Fig. 8:**
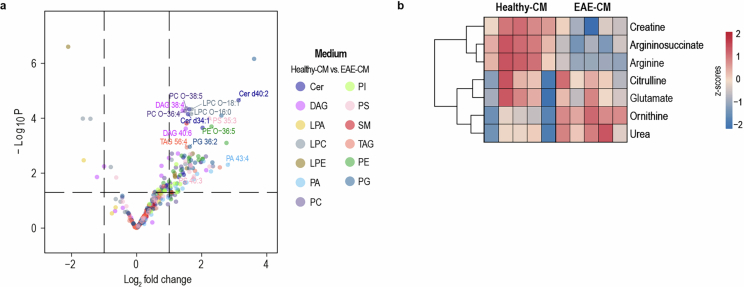
Characterization of EAE conditioned media. **a**, Volcano plot showing lipidomic differences between conditioned media generated from spinal cord of healthy (Healthy-CM) or animals suffering from EAE at peak-disease (EAE-CM). n = 5 animals per condition. LPA, lysophosphatidic acid; PE, phosphatidylethanolamine; PA, phosphatidic acid; PC, phosphatidylcholine; SM, sphingomyelin; PI, phosphatidylinositol; PG, phosphatidylglycerol; PS, phosphatidylserine; LPC, lysophosphatidylcholine; LPE, lysophosphatidylethanolamine; DAG, diacylglycerol; Cer, ceramide; TAG, triacylglycerol. **b**, Differences in arginine related metabolite composition between conditioned media. Heatmap depicts row z-scores of metabolites. n = 5 animals per condition. All metabolites except for citrulline and glutamate are significantly changed (P < 0.05). Statistical analysis performed with Welch Two Sample t-test. Source data

Mimicking the LDAM phenotype in EAE and analogous to others examining detrimental effects of LDAM-conditioned medium on neurons^52^, we generated medium conditioned with the spinal cords of healthy animals (healthy-CM) or animals at peak EAE (EAE-CM). Lipidomics demonstrated that compared to healthy-CM, EAE-CM contained significantly more lipids, including long-chain ceramides (Cer d40:2, Cer d34:1) and various phospholipids like phosphatidylcholines and phosphatidylethanolamines (Extended Data Fig. 8a). Although in this setup we could not detect the polyamines, analogous to the spinal cord, EAE-CM contained less arginine and creatine and more ornithine. We also noted augmented urea and GM-CSF (Figs. 2 and 4l and Extended Data Fig. 8b). Having established this, we next stimulated macrophages with either conditioned medium. EAE-CM increased macrophage lipid load and induced ARG1 (Fig. 4m).

As arginine levels in supernatants were only decreased by one-third after LPS/GM-CSF-induced arginase catabolism (Extended Data Fig. 7d), we next depleted extracellular arginine using recombinant ARG1 (recARG1)^53^ and examined oxidative stress. Extracellular arginine was robustly depleted, and LPS/GM-CSF-mediated oxidative stress was further elevated (Extended Data Fig. 7l,m). To understand the mechanisms therein, we measured the expression of several antioxidant genes, including those associated with the glutathione pathway (*Gss* and *Gsr*), heme oxygenase 1 (HO-1; encoded by *Hmox1*) and the well-established central antioxidant regular NRF2 (encoded by *Ne2l2*)^21,54^. Extracellular arginine depletion in the context of LPS/GM-CSF significantly reduced *Ne2l2* and *Hmox1* expression, with attenuated HO-1 levels verified by flow cytometry, and was associated with elevated lipid peroxidation (Fig. 4n–p and Extended Data Fig. 7n). Together, these observations suggest that at peak disease, lipid-laden ARG1^+^ Mdcs within demyelinating lesions may deprive the local microenvironment of arginine, thereby instigating oxidative stress and lipid peroxidation.

### ARG1^+^ Mdcs skew spinal cord metabolism worsening EAE

**Fig. 5:**
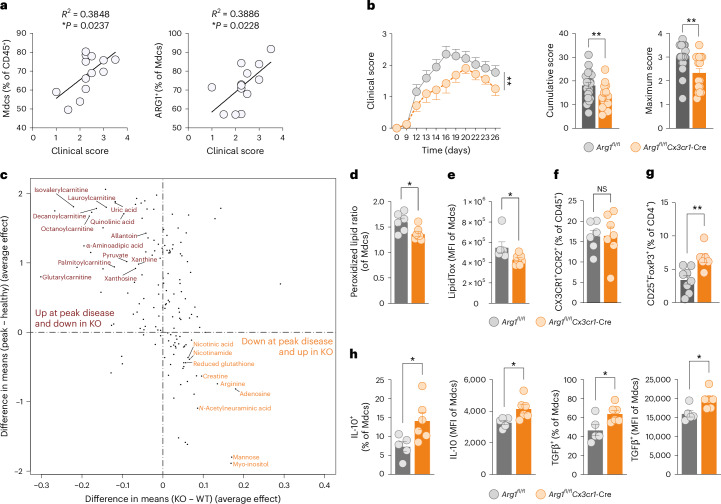
ARG1^+^ Mdcs augment disease severity, altering the spinal cord metabolic and anti-inflammatory environment in neuroinflammation. **a**, Two-tailed simple linear regression of spinal cord Mdc amounts within the CD45^+^ pool and ARG1 expression within Mdcs with clinical score; *n* = 13. **b**, Clinical course and cumulative and maximum score of *Arg1*^*fl/fl*^ (*n* = 21) and *Arg1*^*fl/fl*^*Cx3cr1*-Cre (*n* = 16) animals subjected to EAE; **P = 0.007, cumulative score; **P = 0.0024, maximum score; **P = 0.0068, area under the curve (AUC) of clinical course. **c**, Scatter plot comparing changes in mean metabolite abundance between healthy animals (*n* = 11) and animals at peak disease (*n* = 8) plotted against changes in mean metabolite abundance between *Arg1*^*fl/fl*^ (*n* = 4) and *Arg1*^*fl/fl*^*Cx3cr1*-Cre (*n* = 5) animals at peak disease in the spinal cord; KO, knockout; WT, wild type. **d**, Lipid peroxidation levels within Mdcs from the spinal cord of *Arg1*^*fl/fl*^ (*n* = 6) and *Arg1*^*fl/fl*^*Cx3cr1*-Cre (*n* = 7) animals at peak disease using C11-BODIPY; *P = 0.0208. **e**, LipidTox MFI of spinal cord Mdcs of *Arg1*^*fl/fl*^ (*n* = 6) and *Arg1*^*fl/fl*^*Cx3cr1*-Cre (*n* = 7) animals at peak disease; *P = 0.0468. **f**, Percentages of spinal cord Mdcs from CD45^+^ cells of *Arg1*^*fl/fl*^ (*n* = 6) and *Arg1*^*fl/fl*^*Cx3cr1*-Cre (*n* = 7) animals at peak disease. **g**, Percentage of CD25^+^FoxP3^+^ T_reg_ cells from CD4^+^ T cells of *Arg1*^*fl/fl*^ (*n* = 8) and *Arg1*^*fl/fl*^*Cx3cr1*-Cre (*n* = 6) animals at peak disease; **P = 0.0059. **h**, Frequency and MFI of IL-10/TGFβ of spinal cord Mdcs of *Arg1*^*fl/fl*^ (*n* = 5) and *Arg1*^*fl/fl*^*Cx3cr1*-Cre (*n* = 6) animals at peak disease; from left to right: *P = 0.0339, *P = 0.0253, *P = 0.0313 and *P = 0.0239. Results are shown as mean ± s.e.m. (**b**,**d**,**e**–**h**). Results were pooled from two (**g**) or three (**b**) independent experiments. Data were analyzed by two-tailed unpaired *t*-tests (**b**,**d**–**h**). Cells were pregated on single viable Ly6G^−^CD3^−^CD11b^+^CD45^hi^ cells (**f**) and then CX3CR1^+^CCR2^+^ cells (**a**,**d**,**e**) or CX3CR1^+^Ly6C^+^ cells (**h**) for Mdcs or CD45^+^CD3^+^CD4^+^ for T cells (**g**). Source data

**Extended Data Fig. 9:**
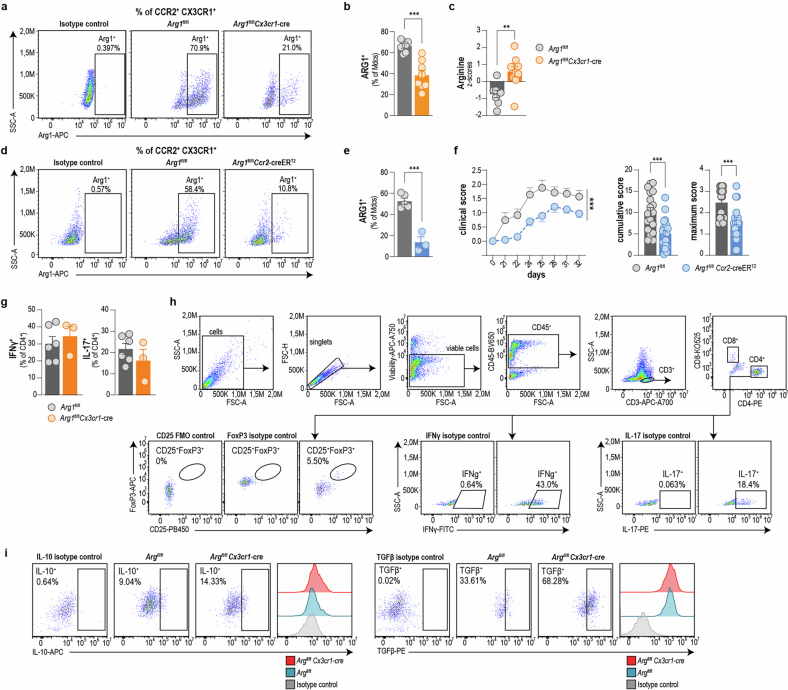
Gating strategies, *Arg1* knockout efficiency and quantification of T cell subsets in *Arg1*^fl/fl^*Cx3cr1*-cre animals and *Arg1*^fl/fl^*Ccr2*-creER^T2^ animals during EAE. **a/b**, Representative flow cytometry plots and quantification depicting percentage ARG1-expression in spinal cord CCR2^+^CX3CR1^+^ Mdcs of *Arg1*^fl/fl^ (n = 7) and *Arg1*^fl/fl^ *Cx3cr1*-cre (n = 8) animals at peak-disease. ***P = 0.002. **c**, CSF Arginine levels of *Arg1*^fl/fl^ (n = 8) and *Arg1*^fl/fl^ *Cx3cr1*-cre (n = 10) animals at peak-disease presented as z-scores from targeted metabolomics analysis. **P = 0.0031 **d/e**, Representative flow cytometry plots and quantification depicting percentage ARG1-expression in spinal cord CCR2^+^CX3CR1^+^ Mdcs of *Arg1*^fl/fl^ (n = 5) and *Arg1*^fl/fl^ *Ccr2*-creER^T2^ (n = 3) animals at peak-disease. ***P = 0.0004. **f**, Clinical course, cumulative and maximum score of *Arg1*^fl/f^ (n = 18) and *Arg1*^fl/fl^ *Ccr2*-creER^T2^ (n = 19) animals subjected to EAE. ***P = 0.0007 (AUC), ***P = 0.0006, ***P = 0.0007. **g**, Percentage IFN-γ^+^ and IL-17^+^ cells within the CD4^+^ T cell pool of *Arg1*^fl/fl^ (n = 6) and *Arg1*^fl/fl^ *Cx3cr1*-cre (n = 3) animals at peak-disease. **h**, Gating strategy for CD4^+^ T cell IL-17^+^, IFN-γ^+^ or CD25^+^FoxP3^+^ subsets. **i**, Plots demonstrating specificity of IL-10 and TGFβ antibodies used for staining on CX3CR1^+^Ly6C^+^ Mdcs. Sample is depicted in red (*Arg1*^fl/fl^ *Cx3cr1*-cre), blue (*Arg1*^fl/fl^) and isotype control in grey. Results are mean ± SEM (**b,c,e,f,g**). Representative results shown from 2 (**d,e**), 3 (**a,b**) or pooled from 3 (**f**) independent experiments. Statistical analysis performed with two-tailed unpaired t-test (**b,c,e,f,g**). Cells were pre-gated on single viable Ly6G^−^CD3^−^CD45^hi^ for Mdcs (**a,b,d,e,i**) or CD45^+^CD3^+^CD4^+^ for T cells (**g**). Source data

Thus far, our data indicate that infiltrating ARG1^+^ lipid-laden Mdcs are associated with arginine consumption and resemble neurologically detrimental LDAMs. Hypothesizing that ARG1^+^ Mdcs are detrimental, we next correlated ARG1 expression within the Mdc pool with clinical score. Consistent with monocyte infiltration being adverse during EAE^6,7^, positive correlations were detected between Mdc amounts within the CD45^+^ immune cell pool and clinical score. ARG1^+^ Mdcs were associated with worsened outcome (Fig. 5a), implying that ARG1 acquisition therein promotes disease pathology. To prove that myeloid ARG1 was detrimental during neuroinflammation, we next subjected *Arg1*^*fl/fl*^*Cx3cr1*-Cre mice to EAE. Flow cytometry demonstrated that a twofold reduction in ARG1 within CCR2^+^CX3CR1^+^ Mdcs was associated with relative increases in CSF arginine (Extended Data Fig. 9a–c), indicative of decreased ARG1-mediated arginine catabolism in *Arg1*^*fl/fl*^*Cx3cr1*-Cre animals. As hypothesized, relative to *Arg1*^*fl/fl*^ littermate controls, overall disease was ameliorated (Fig. 5b). To ascertain that these effects were specific for Mdcs and not CAMs, cDC2s or microglia, we crossed *Arg1*^*fl/fl*^ mice with animals expressing the tamoxifen-inducible monocyte-specific Cre recombinase (*Ccr2*-Cre^ER^^T2^)^49^, generating *Arg1*^*fl/fl*^*Ccr2*-Cre^ER^^T2^ animals, and induced EAE in the presence of tamoxifen. *Arg1* was robustly deleted within CCR2^+^CX3CR1^+^ Mdcs, and this experimental setup confirmed that ARG1-deficient Mdcs were associated with dampened disease (Extended Data Fig. 9d–f). Together, these complementary genetic approaches illustrate that *Arg1*-expressing Mdcs promote worsened EAE outcome.

We next examined how myeloid ARG1 deficiency impacts the spinal cord metabolic landscape and the trajectory of previously identified DLMs and HLMs relative to health (Fig. 1e,f). Targeted metabolomics indicated that the average metabolic effect of ARG1 deficiency was associated with increased arginine and a trajectory reversal of several DLMs, with many of those up or down at peak disease relative to health displaying opposing patterns in *Arg1*^*fl/fl*^*Cx3cr1*-Cre animals (Figs. 1e and 5c). Therein, neurotoxic quinolinic and α-aminoadipic acid, DLMs associated with oxidative stress, lipid peroxidation and parenchymal cell death^17,18^, were, on average, elevated at peak disease and decreased in ARG1-deficient animals (Figs. 1e and 5c). Other DLMs, in particular those associated with purine catabolism and salvage, including uric acid and its upstream and downstream metabolites xanthosine and allantoin, respectively^55^, displayed a similar trajectory (Fig. 5c), suggesting that ARG1-deficient animals display altered cerebral bioenergetics and energy metabolism. Several medium- to long-chain carnitines generated from amino acid metabolism (octanoyl-, decanoyl-, lauroyl- and palmitoylcarnitine), previously described to impair mitochondrial oxidative phosphorylation^56^ and promote inflammation, were lower in ARG1-deficient animals (Fig. 5c and Extended Data Fig. 2c). This finding, together with higher levels of neuroprotective reduced glutathione^21^ in ARG1-deficient animals, indicated that their improved outcome was associated with attenuated oxidative stress (Fig. 5c). Given this and that lipid-laden ARG1^+^ Mdcs at peak disease are engaged in redox homeostasis, likely via impacting extracellular arginine levels (Fig. 4), we next examined lipid peroxidation and lipid ladenness within both genotypes of CCR2^+^CX3CR1^+^ Mdcs, hypothesizing attenuated lipid peroxidation within ARG1-deficient Mdcs. Indeed, lipid peroxidation and load were significantly decreased in ARG1-deficient Mdcs (Fig. 5d,e). These effects were independent of differences in Mdc spinal cord frequencies between genotypes, as Mdc percentages among the CD45^+^ pool were similar (Fig. 5f).

Several neuroprotective metabolites associated with energy metabolism and regulatory T (T_reg_) cell expansion that were, on average, down at peak disease were up in *Arg1*^*fl/fl*^*Cx3cr1*-Cre animals. These included creatine, mannose and adenosine^23,57,58^ (Fig. 5c). Examining helper T cell subsets at peak disease revealed no differences in type 1 helper T cells and IL-17-producing helper T cells, whereas T_reg_ cells were increased in *Arg1*^*fl/fl*^*Cx3cr1*-Cre animals (Fig. 5g and Extended Data Fig. 9g,h). Increased T_reg_ cells were associated with elevated levels of T_reg_ cell-promoting cytokines IL-10 and transforming growth factor-β (TGFβ) within the Mdc pool of *Arg1*^*fl/fl*^*Cx3cr1*-Cre animals (Fig. 5h and Extended Data Fig. 9i). Together, these findings suggest that myeloid ARG1 deficiency is associated with both an altered metabolic landscape and cytokine profiles within the Mdc pool, resulting in modified T_reg_ cell frequencies. Although this may reflect the better outcome of *Arg1*^*fl/fl*^*Cx3cr1*-Cre animals, it suggests contributions for CNS-localized arginine to dampened disease.

### Arginine scarcity negatively impacts neuroinflammation

**Fig. 6:**
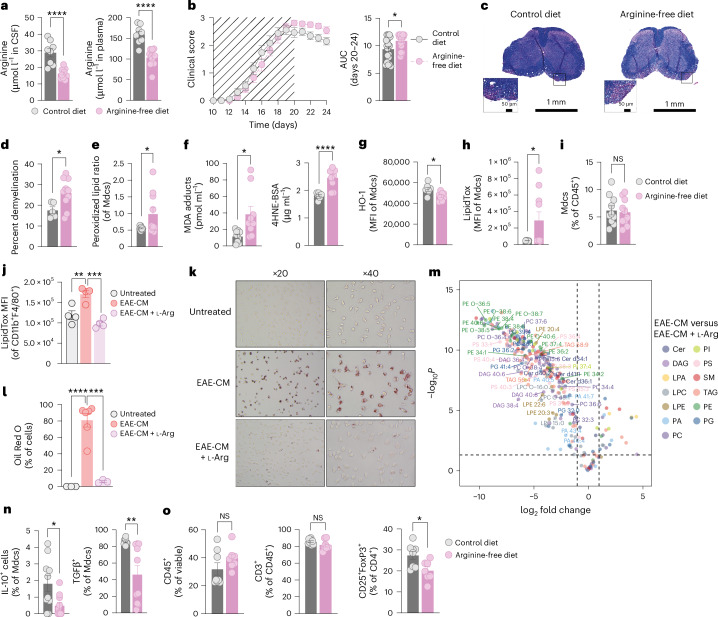
Arginine depletion in EAE promotes Mdc dysfunction and pleiotropic negative neuroinflammatory effects. **a**, CSF/plasma arginine of animals on a control (*n* = 8) or arginine-free (*n* = 11) diet; ****P < 0.0001. **b**, Clinical course of animals on a control (*n* = 19) or arginine-free (*n* = 19) diet subjected to EAE. AUC performed between days 20 and 24 is shown; *P = 0.0146. **c**,**d**, Representative Luxol fast blue staining (**c**) and quantification (**d**) of animals fed a control (*n* = 5) or arginine-free (*n* = 10) diet; *P = 0.0322. **e**, Lipid peroxidation of spinal cord Mdcs using C11-BODIPY of animals fed a control (*n* = 10) or arginine-free (*n* = 10) diet at peak disease; *P = 0.0434. **f**, Malondialdehyde (MDA) adduct and 4-hydroxynonenal-bovine serum albumin (4-HNE-BSA) from the spinal cord; *n* = 9 animals per diet; *P = 0.017; ****P < 0.0001. **g**, HO-1 MFI of spinal cord Mdcs of animals fed a control (*n* = 7) or arginine-free (*n* = 8) diet at peak disease; *P = 0.0222. **h**, LipidTox MFI from spinal cord Mdcs of animals fed a control (*n* = 10) or arginine-free (*n* = 10) diet at peak disease; *P = 0.0320. **i**, Frequency of spinal cord Mdcs from a CD45^+^ cell pool of animals fed a control (*n* = 10) or arginine-free (*n* = 10) diet at peak disease. **j**, LipidTox MFI of treated BMDMs;*n* = 4 samples per condition; ANOVA ***P = 0.0008; **P = 0.0033; ***P = 0.001. **k**,**l**, Representative Oil Red O staining (**k**) and quantification (**l**) of treated BMDMs; *n* = 3 (untreated/EAE-CM + L-arginine) and 6 (EAE-CM); ANOVA ****P < 0.0001; ***P = 0.0002 and ****P < 0.0001. **m**, Lipidomics differences between treated BMDMs; *n* = 4 samples per condition; Cer, ceramide; DAG, diacylglycerol; LPA, lysophosphatidic acid; LPC, lysophosphatidylcholine; LPE, lysophosphatidylethanolamine; PA, phosphatidic acid; PC, phosphatidylcholine; PE, phosphatidylethanolamine; PG, phosphatidylglycerol; PI, phosphatidylinositol; PS, phosphatidylserine; SM, sphingomyelin; TAG, triacylglycerol. **n**, Percentage of IL-10^+^ and TGFβ^+^ cells from spinal cord Mdcs of animals fed a control (*n* = 10) or arginine-free (*n* = 10) diet at peak disease; *P = 0.0194; **P = 0.0021. **o**, Frequency of CD45^+^ cells, T cells and T_reg_ cells from animals fed a control (*n* = 8) or arginine-free (*n* = 7) diet at peak disease; *P = 0.0163. Results are shown as mean ± s.e.m. (**a**,**b**,**d**–**j**,**l**,**n**,**o**). Representative results were pooled from two (**e**,**h**,**n**) independent experiments. Data were analyzed by one-way ANOVA followed by a Tukey’s multiple-comparisons post-test (**j**,**l**) or two-tailed unpaired *t*-test (**a**,**b**,**d**–**i**,**n**,**o**). Cells were pregated on single viable CD11b^+^F4/80^+^ cells for BMDMs (**j**) or Ly6G^−^CD3^−^CD11b^+^CD45^hi^ cells (**i**) and then CX3CR1^+^CCR2^+^ cells for Mdcs (**a**,**d**,**e**,**n**) or CD45^+^CD3^+^CD4^+^ cells for T cells (**o**). Source data

**Extended Data Fig. 10:**
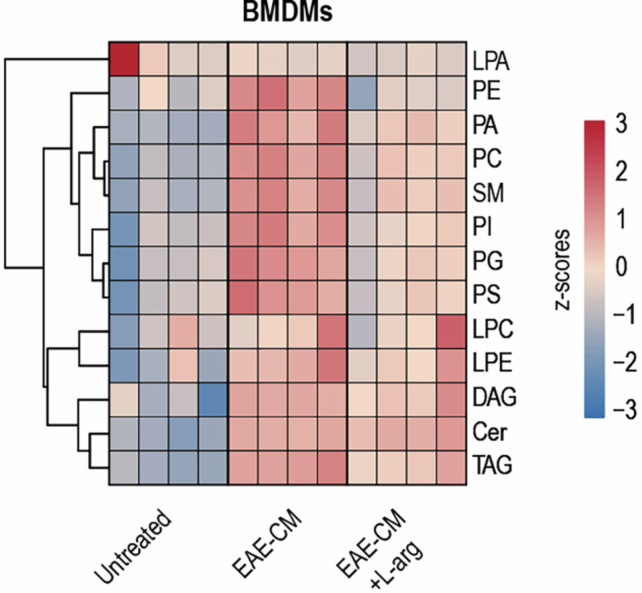
Overall abundance of lipid classes in macrophages stimulated with EAE-CM in arginine presence or absence. Heatmap depicting abundance of lipid species in BMDMs treated as indicated. EAE-CM, EAE-conditioned medium; L-arg, L-arginine. n = 4 samples per condition. Heatmap legend represents row z-scores. Significantly changed lipid species adj P < 0.05 are PE, PA, PC, SM, PI, PG, PS, TAG LPA, lysophosphatidic acid; PE, phosphatidylethanolamine; PA, phosphatidic acid; PC, phosphatidylcholine; SM, sphingomyelin; PI, phosphatidylinositol; PG, phosphatidylglycerol; PS, phosphatidylserine; LPC, lysophosphatidylcholine; LPE, lysophosphatidylethanolamine; DAG, diacylglycerol; Cer, ceramide; TAG, triacylglycerol. Statistical analysis performed with one-way ANOVA followed by Tukey HSD post-test. Source data

Testing this, we next fed animals an arginine-sufficient or arginine-deficient diet, with the arginine-free diet significantly decreasing plasma and CSF arginine and subsequently inducing EAE (Fig. 6a). Consistent with hypothesized beneficial effects of arginine, these animals exhibited mildly worsened clinical outcome, with Luxol fast blue staining indicating significantly more demyelination at the disease end point (Fig. 6b–d). Mdcs of mice fed an arginine-free diet exhibited heightened lipid peroxidation (Fig. 6e), suggesting that extracellular arginine scarcity may contribute to oxidative damage and Mdc dysfunction. Lipid peroxidation within Mdcs was accompanied by enhanced 4-hydroxynonenal and malondialdehyde spinal cord adducts and was simultaneously associated with decreased HO-1 within Mdcs (Fig. 6f,g). These Mdcs were more lipid laden (Fig. 6h), suggesting that local microenvironmental arginine availability in EAE directly modulates lipid droplet anabolism or catabolism of lesion-associated Mdcs. These effects were not due to increased spinal cord Mdc frequencies as animals under both dietary regimens exhibited similar amounts (Fig. 6i). Knowing that EAE-CM increase macrophage lipid load (Fig. 4m) and that ARG1^+^ cells engage in lipid metabolism (Fig. 4b), we next examined how L-arginine impacts macrophage lipid ladenness. LipidTox flow cytometry and Oil Red O staining revealed that macrophage lipid ladenness induced by EAE-CM was attenuated by arginine (Fig. 6j–l), suggesting that extracellular arginine availability positively impacts lipid metabolism. The overall abundances of several EAE-CM-induced lipid classes (phosphatidylcholine, phosphatidylethanolamine, sphingomyelin, phosphatidic acid and phosphatidylglycerol) were attenuated (Extended Data Fig. 10), further strengthening the effects of L-arginine on macrophage lipid ladenness. Although we noted no effect on overall ceramide levels, long-chain ceramides (Cer d40:2, Cer d34:1) specifically enriched in EAE-CM and several ethanolamine plasmalogens (PE O−36:5, PE O−38:7, PE O−40:6) that are important for myelin compaction and stability^59^ were reduced by L-arginine (Fig. 6m). Together, we propose that an arginine-depleted environment in EAE may promote neuroinflammation by enhancing Mdc dysfunction, a hypothesis supported by reduced IL-10 and TGFβ levels within the Mdc pool of animals fed an arginine-free diet (Fig. 6n).

Noting the reciprocal Mdc cytokine profiles of *Arg1*^*fl/fl*^*Cx3cr1*-Cre animals at peak disease that also exhibited elevated T_reg_ cells (Fig. 5g,h), we next examined how arginine availability influences T_reg_ cells. Although animals fed both diets displayed similar immune cell infiltration in the spinal cord and identical T cell frequencies within the CD45^+^ immune cell pool therein, at the disease end point, T_reg_ cells were decreased in animals fed an arginine-free diet (Fig. 6o).

## Discussion

Here, we found that following EAE-driven neuroinflammation, the CNS undergoes profound metabolic alterations. We identified comprehensive sets of HLMs, DLMs and RLMs, providing compelling evidence that Mdcs alter homeostatic CNS metabolism. Notably, we could assign arginine catabolism as an Mdc-driven, ARG1-dependent metabolic disturbance, harboring disease-promoting properties.

CNS lipid dyshomeostasis is a hallmark of several neurodegenerative diseases, including MS, and is implicated in disease pathogenesis. In particular, in demyelinating diseases like MS, lesion-associated Mdcs need to efficiently process myelin debris, enabling CNS repair^60^. Sustained uptake and aberrant myelin-derived lipid accumulation together with oxidative stress is linked to defective myeloid lipid handling, mitochondrial injury, hypoxia and increased inflammation^37,61^. ARG1-deficient Mdcs from *Arg1*^*fl/fl*^*Cx3cr1*-Cre animals at peak disease exhibited lower lipid load and peroxidation, while conversely arginine scarcity augmented macrophage lipid load and peroxidation and simultaneously decreased *Nrf2* and HO-1 expression. Macrophages in active MS lesions and oligodendrocytes at lesion edges stain positive for nuclear NRF2 (ref. ^62^). Similarly, CD68^+^HO-1^+^ myeloid cells are elevated in EAE lesions^63^. This may reflect a cell defense mechanism against the profound oxidative damage and mitochondrial injury characteristic of MS and EAE lesions, which gives rise to a state of hypoxia associated with energy deficiency^37^. Accordingly, NRF2- and HO-1-deficient animals exhibit worsened EAE severity^64,65^. We demonstrated that subsets of *Arg1*- and *Slc7a2*-coexpressing Mdcs were enriched in gene sets related to lipid responses, localization and transport and that, although extracellular arginine availability negatively impacted lipid levels, arginine scarcity augmented macrophage lipid load. Together, our findings suggest that limiting extracellular arginine availability via ARG1 for cellular import through SLC7A2 dysregulates lipid metabolism, promoting mitochondrial dysfunction and inflammation. Accordingly, ARG1^+^ Mdcs are reminiscent of LDAM^48^ and detrimental in neuroinflammation.

In line with previous work demonstrating predominant ARG1 on Mdcs versus microglia^66^, ARG1^+^ Mdcs represented the main ARG1-expressing population at peak disease. Genetic fate mapping also revealed a minor population of CD11b^+^CD45^+^MS4A3–tdTomato^−^CX3CR1^+^P2RY12^−^ cells expressing ARG1, which we designated non-monocyte-derived CAMs or cDC2s. Additionally, as infiltrating monocytes differentiated into moMACs and moDCs, they upregulated ARG1 expression, with the highest expression in moMACs. Our observations support that ARG1 expression is an integral part of the niche-specific, GM-CSF- and IFNγ-driven gradual differentiation from infiltrating monocytes to disease-promoting, phagocytic moMACs that may have moDCs as an intermediate state^9^. Accordingly, in the context of LPS, IFNγ treatment exclusively led to iNOS^+^ macrophages and simultaneous GM-CSF and IFNγ treatment led to ARG1^+^iNOS^+^ macrophages, whereas GM-CSF treatment alone mainly induced ARG1^+^iNOS^−^ macrophages that engaged in arginine catabolism. Mdcs from *Csf2rb*^*fl/fl*^*Ccr2*-Cre^ER^^T2^ animals treated with tamoxifen also exhibited reduced ARG1 expression. Here, tamoxifen was administered at disease onset as *Csfr2b* expression on CCR2^+^Ly6C^hi^ cells is critical for EAE development^49^. Of note, tamoxifen itself ameliorates EAE^67^, which could explain differences in clinical score between *Arg1*^*fl/fl*^*Cx3cr1*-Cre and *Arg1*^*fl/fl*^*Ccr2*-Cre^ER^^T2^ animals.

Both *Arg1*^*fl/fl*^*Ccr2*-Cre^ER^^T2^ and *Arg1*^*fl/fl*^*Cx3cr1*-Cre animals exhibited better outcome, which is in agreement with milder EAE in animals subjected to chemical ARG1 inhibition^68^. Beneficial effects of *Arg1* deletion in innate immune cells associated with augmented IL-10 and improved outcome also occur during allergic lung inflammation^69,70^. Nonetheless, we acknowledge the limitations of our experimental setup using *Arg1*^*fl/fl*^*Cx3cr1*-Cre animals, which aside from broad targeting activity also include confounding effects of *Cx3cr1* haploinsufficiency that in turn can influence myeloid cell activation and worsen EAE severity^71,72^. Although we cannot conclusively rule this out, *Arg1*^*fl/fl*^*Cx3cr1*-Cre animals exhibited better clinical outcome, and ARG1-expressing CAMs or cDC2s represented a minor population in the inflamed CNS, suggesting that their phenotype is unlikely due to off-target Cre activity or partial CX3CR1 loss.

Importantly, our findings differ from work demonstrating prevalence of iNOS-expressing Mdcs at peak neuroinflammation, challenging the general concept that ARG1 is a signature enzyme associated with resolving inflammation^9,10^. Rather, ARG1^+^ Mdcs exhibited signatures instructed by lesion-associated pathophysiological cues, where they engaged in arginine catabolism. In support of this, several polyamines downstream of ARG1 were significantly elevated in EAE. However, metabolic flux experiments are needed to fully elucidate the contributions of ARG1 to its downstream metabolites in EAE. Although controversy exists regarding ARG1 and iNOS expression in human myeloid cells^73^, ARG1 is present in peripheral blood mononuclear cells of individuals with MS^74^. Despite the small number of individuals with MS analyzed, we expand on these findings by revealing that ARG1-expressing myeloid cells are found in human MS lesions. We disclose that mice with EAE fed an arginine-free diet display lower IL-10, TGFβ and T_reg_ cell levels, highlighting the crucial role for arginine catabolism downstream of ARG1 in promoting neuroinflammation. Although arginine influences T cell survival and proliferation^75,76^, in neuroinflammation, arginine exerted unremarkable influences on T cell frequencies. Instead, arginine scarcity dampens Mdc-driven IL-10 and TGFβ production, setting the stage for reduced T_reg_ cell abundance, thereby conferring worsened EAE outcome^77^, although we cannot rule out direct effects of arginine on T_reg_ cell abundance^78^. Our findings agree with neuroprotective functions of arginine in individuals with cerebral ischemia/reperfusion injury^79^.

Together, our data suggest a detrimental role of infiltrating ARG1-expressing Mdcs that exhibit increased lipid load, oxidative stress and peroxidation. ARG1^+^ Mdcs drive arginine catabolism and attenuate neuroprotective metabolites and IL-10 and TGFβ levels. This causes increased spinal cord oxidative stress, energy metabolism deficits and neuroinflammation. Although the perturbations used here indicate that arginine availability impacts Mdc lipid ladenness, they remain associative. Whether arginine availability influences lipid uptake, synthesis, catabolism or efflux still remains to be elucidated. Further, it remains to be determined if similar HLMs, DLMs and RLMs are found in relapsing–remitting MS models and the contributions of Mdcs and arginine therein.

## Methods

### Human samples and neuropathological assessment

All procedures done on human postmortem tissues in this study were approved by the Ethics Committee of Medical University of Vienna (votes: 1636/19 and 1067/2024). Tissue samples were acquired from the tissue bank of the Division for Neuropathology and Neurochemistry at the Medical University of Vienna. MS was diagnosed either during the individual’s life or via autopsy and neuropathological examination. The mean age was 52 ± 15 years (*n* = 5 men and *n* = 13 women). Disease clinical course was classified as acute (*n* = 3), relapsing and remitting (*n* = 5), secondary progressive (*n* = 4) and primary progressive (*n* = 2). In some cases, the definite clinical course was not available; these cases were designated as unknown (*n* = 4). Control individuals showed an absence of major pathological alterations; however, minor vascular pathology in the form of small vessel disease was frequently detected. Only white matter sections without pronounced pathology were selected. The mean age for control individuals was 63 ± 15 years (*n* = 4 men and *n* = 3 women). Participant characteristics are summarized in Supplementary Table 7.

### Experimental animals

Mice were bred and housed in pathogen-free facilities at the Medical University of Vienna and were maintained on a 12-h light/12-h dark cycle at 21–23 °C and 45–65% humidity. C57BL/6J mice were obtained from the Animal Core Facility of the Medical University of Vienna or were purchased from Janvier (SC-C57J-M). *Csf2rb*^*fl/fl*^*Ccr2*-Cre^ER^^T2(−mKate2)^ mice^49^ were kindly provided by B. Becher (Institute of Experimental Immunology, University of Zürich, Switzerland) and were further crossed to mice expressing a CAG-*loxP*-STOP-*loxP*-tdTomato cassette in the *Rosa26* locus (B6;129S6-Gt(*Rosa*)26Sor^tm14(CAG−tdTomato)Hze/J^, The Jackson Laboratory, stock 007908, *R26*^*tdTomato*^), which were provided by C. Österreicher (Center for Physiology and Pharmacology, Medical University of Vienna, Austria)^80^, to generate *R26*^*tdTomato*^*Ccr2*-Cre^ER^^T2^ fate-mapping animals. *R26*^*tdTomato*^*Ms4a3*-Cre mice were generated by crossing *R26*^*tdTomato*^ mice with *Ms4a3*-Cre mice (C57BL/6J-Ms4a3^*em2(cre)Fgnx*^/J, The Jackson Laboratory, 036382). *Arg1*-eYFP reporter mice (B6.129S4-*Arg1*^tm1.1Lky^/J, The Jackson Laboratory, 0015857) were crossed with *R26*^*tdTomato*^*Ccr2*-Cre^ER^^T2^ mice to generate *R26*^*tdTomato*^*Ccr2*-Cre^ER^^T2^*Arg1*-eYFP animals. *Arg1*^*fl/fl*^ mice (C57BL/6-*Arg1*^*tm1Pmu*^/J, The Jackson Laboratory, 008817) were a kind gift of P. J. Murray (Immunoregulation Research Group, Max Planck Institute of Biochemistry, Germany)^81^ and were crossed with *Cx3cr1*-Cre mice (B6J.B6N(Cg)-*Cx3cr1*^*tm1.1(cre)Jung*^/J, The Jackson Laboratory, 025524) or *Ccr2*-Cre^ER^^T2^ mice to generate *Arg1*^*fl/fl*^*Cx3cr1*-Cre or *Arg1*^*fl/fl*^*Ccr2*-Cre^ER^^T2^ mice, respectively. The presence of respective transgenes was confirmed by PCR analysis of DNA from ear biopsies of genetically modified animals using the following primer pairs (Microsynth): *Csf2rb*: 5’-GAGAGAGGGTCCTTTTGGTC-3’ (forward) and 5’-CCTCCCCTCTTCTGTATCTTC-3’ (reverse); *Ccr2*-Cre^ERT2^-mKATE: 5’-CTCTACTTCATCGCATTCCTTGC-3’ (forward) and 5’-GGTTGATGAAGGTTTTGCTGC-3’ (reverse) or 5’-AGAAAGTGAGCCCTCTGTATGG-3’ (forward) and 5’-TTGGCATTTCCTGGTGAGC-3’ (reverse); *Cx3cr1*-Cre: 5’-TCGCGATTATCTTCTATATCTTCAG-3’ (forward) and 5’-GCTCGACCAGTTTAGTTACCC-3’ (reverse); *Ms4a3*-Cre: 5’-AGAGAAATCATCAGGGCAGAAAT-3’ (common forward), 5’-GAAAGGGGAACAAGCGAAGAT-3’ (wild-type reverse) and 5’-TTGGCGAGAGGGGAAAGAC-3’ (mutant reverse); tdTomato-wild-type: 5’-AAGGGAGCTGCAGTGGAGTA-3’ (forward) and 5’-CCGAAAATCTGTGGGAAGTC-3’ (reverse); tdTomato-mutant: 5’-CTGTTCCTGTACGGCATGG-3’ (forward) and 5’-GGCATTAAAGCAGCGTATCC-3’ (reverse); *Arg1*: 5’-TGCGAGTTCATGACTAAGGTT-3’ (forward) and 5’-AAAGCTCAGGTGAATCGG-3’ (reverse); *Arg1*-YFP_wild-type: 5’-AGAGCAAGCACCCCGTTTCTTCTC-3’ (forward) and 5’-GCTGTGATGCCCCAGATGGTTTTC-3’ (reverse); *Arg1*-YFP_mutant: 5’-TGAGCAAAGACCCCAACGAGAAGC-3’ (forward) and 5’-GCTGTGATGCCCCAGATGGTTTTC-3’ (reverse). For all experiments, male animals (8–12 weeks old) were used with the exception of BMDM generation and experiments involving *Csf2rb*^*fl/fl*^*Ccr2*-Cre^ERT2^ mice, where both sexes were included. All animal experiments were performed in strict accordance with regulations of the relevant animal welfare acts and protocols approved by the respective regulatory bodies (Austrian Ministry of Sciences, 2022-0.474.463 and 2025-1.040.322).

### EAE induction

Active EAE was induced by subcutaneous immunization with 75 μg of MOG_35–55_ (Charité) emulsified in complete Freund’s adjuvant (1:2 dilution, 150 μl per mouse), which refers to incomplete Freund’s adjuvant (Merck, F5506-10X10ML) enriched with 10 mg ml^−1^ *Mycobacterium tuberculosis* (Difco/BD Pharmingen, H37Ra). Pertussis toxin from *Bordetella pertussis* (List/Quadratech, 181 or Hooke Laboratories, BT-0105) was administered intraperitoneally at days 0 and 2 after immunization (200 ng per mouse for pertussis toxin from List and 150 ng per mouse for pertussis toxin from Hooke Laboratories at days 0 and 2). For tamoxifen-inducible Cre strains, with the exception of *Csf2rb*^*fl/fl*^*Ccr2*-Cre^ER^^T2^ mice, animals were fed a tamoxifen diet (Tam400/CreER, tamoxifen citrate 400 ppm, ENVIGO, TD.55125IC) for 3 weeks, followed by 1 week of a regular chow diet (ssniff, V1536-000). Thereafter, EAE was induced, and animals were re-fed a tamoxifen diet throughout the course of EAE. For *Csf2rb*^*fl/fl*^*Ccr2*-Cre^ER^^T2^ and *Csf2rb*^+/+^*Ccr2*-Cre^ER^^T2^ littermate control animals, tamoxifen (5 mg per mouse) was administered as previously described^49^. Briefly, tamoxifen was dissolved in corn oil and administered by oral gavage (250 μl per mouse at 20 mg ml^−1^) at the first sign of clinical symptoms. Tamoxifen was administered two times (24–48 h apart), and animals were collected 3–4 days after the first dose (scores of 1.5–2). To assess the effects of arginine in EAE, animals were fed a control (Research Diets, A10021B) or arginine-free (Research Diets, A10036) diet 14 or 21 days before EAE, and animals were maintained on their respective dietary regimens throughout EAE. For arginine-free diet studies involving clinical outcome, only animals developing peak disease (score 3) were included.

Clinical signs of EAE were assessed using the following disease scores: no clinical signs (0), partial tail weakness (0.5), full paralysis of the tail (1), changes in gait or inability to climb (1.5), paralysis of one hind limb (2), paralysis of one hind limb and restraints in second hind limb (2.5), total paralysis of the hind limbs (3), hind limb paralysis and restraints in one fore limb (3.5), tetraparese (4) and moribund animal/death (5). EAE experiments were terminated at the following stages of disease development: the ‘onset’ stage is defined as the first time point at which immunized animals lose weight (7–10%) compared to the previous day, ‘peak disease’ is defined as the first day the animals display a clinical score of 3–4 (with peak in scores), and ‘recovery’ is defined as a distinct drop in score from a previous peak score of 3–4 to 1–2. The course of disease was monitored and evaluated via clinical scores. Additionally, the cumulative scores (summation of all daily clinical scores per animal over time), maximum scores (the highest clinical score reached per animal) and AUC are reported. Animals exhibiting extreme weight loss due to tamoxifen diet (>20% after 2 weeks) or those that did not develop disease (score 0) were excluded from the study.

### Spinal cord cell isolation

Mice were killed, and spinal cords were extracted, cut into small pieces and digested using either a tissue dissociation kit (Miltenyi Biotec, tissue dissociation kit 1 for inflamed neural tissue, 130-110-201) according to the manufacturer’s instructions or 3 ml of digestion medium (DMEM supplemented with 1 mg ml^−1^ papain (Sigma-Aldrich, P4762), 0.03 mg ml^−1^ DNase I (Roche, 11284932001) and 0.5 mg ml^−1^ collagenase/dispase (Sigma-Aldrich, 10269638001)). In experiments where digestion medium was used, mechanical and enzymatic dissociation was performed with a GentleMACS Octo Dissociator with Heaters (Miltenyi Biotec) and a spinal cord-specific program (37C_ABDK_01). Digestion was stopped by the addition of 5 ml of neutralization medium (DMEM supplemented with 10% fetal bovine serum). Tissue homogenate was filtered through a prewet, 70-μm cell strainer and postwashing and centrifuged at 250*g* for 5 min at 21 °C. The supernatant was discarded, and isolated cells were separated from debris/myelin using a 70%/37%/30% layered Percoll gradient (Sigma-Aldrich, P1644) with centrifugation at 300*g* for 40 min at 18 °C without braking. Three milliliters of the immune cell-containing 70%/37% interphase was collected, washed with 9 ml of 1× HBSS (Gibco, 14175-05) and pelleted by centrifugation at 500*g* for 7 min at 4 °C. Debris removal in experiments involving the Miltenyi Biotec tissue dissociation kit was performed with Debris Removal Solution (Miltenyi Biotec, 130-109-398). After cell isolation with either protocol, cell pellets were resuspended in 1 ml of flow buffer (PBS supplemented with 1% fetal calf serum (FCS)). Cells were counted and again pelleted by centrifugation at 500*g* for 7 min at 4 °C. Finally, cells were resuspended with an appropriate volume of flow buffer and further prepared for flow cytometry or sorting by FACS.

### CSF isolation

CSF was isolated from the cisterna magna as previously described^82^. Briefly, animals were anesthetized with ketamine (20% Ketasol, 100 mg ml^−1^, Livisto) and xylazine (10% Xylasol, 20 mg ml^−1^, Livisto) diluted in PBS. An incision from the top of the skull to the dorsal thorax was made, and musculature from the skull base to the first vertebrae was removed, exposing the meninges overlying the cisterna magna. The tissue above the cisterna magna was removed, and the surrounding area was cleaned with a cotton swab to eliminate residual blood or other interstitial fluid. The arachnoid membrane above the cisterna magna was then punctured with a microneedle. This resulted in the release of CSF, which is under positive pressure. CSF was pulse centrifuged and stored at −80 °C until subsequent downstream analyses.

### BMDM culture and stimulations

For preparation of bone marrow cell suspensions, cells were flushed from mouse femurs and tibiae with sterile PBS (Gibco, 14190-094). Cell suspensions were filtered through 70-μm cell strainers (Falcon, 352350), and two times the volume of erythrocyte lysis buffer (0.15 M NH_4_Cl, 10 mM KHCO_3_, 0.1 mM Na_2_EDTA, pH 7.2–7.4) was added for 5 min. Cells were pelleted and resuspended in RPMI-1640 (Gibco, 61870-044) supplemented with 10% FCS (Sigma-Aldrich, F7524-500ML), 100 U ml^−1^ penicillin/streptomycin (Lonza, LON17-745E and Capricorn, AAS-B) and 30 ng ml^−1^ M-CSF (R&D Systems, 416-ML-050). Cells were then transferred to 10-cm culture dishes (Sarstedt, 82.1473.001) at a concentration of 0.5 million cells per ml. After 3 days of cultivation, fresh RPMI-1640 supplemented as described above was added (1/10 of the total volume). Three days later, cells were washed once with prewarmed PBS and detached using prewarmed CellStripper (Corning, 15313661). Cells were replated at a concentration of 1 million cells per ml in 12- or 24-well plates (Costar, 3737) and stimulated with 100 ng ml^−1^ LPS (Invivogen, tlrl-3pelps), 20 ng ml^−1^ IFNγ (Miltenyi Biotec, 130-105-774), 20 ng ml^−1^ GM-CSF (R&D Systems, 415-ML-050), 20 ng ml^−1^ IL-4 (Miltenyi Biotec, 130-097-761) and 20 ng ml^−1^ IL-13 (Miltenyi Biotec, 130-094-070). Supernatants from treated cells were centrifuged for 5 min at 4 °C and 500*g*, immediately snap-frozen in liquid nitrogen and stored at −80 °C until subsequent downstream analyses. For experiments where extracellular arginine was depleted, 300 ng ml^−1^ recARG1 (Bio Cancer, PEG-BCT-100, rhArgIpeg5000) or RPMI medium without lysine and arginine (Thermo Scientific, 88365, substituted with 0.21857923 mM L-lysine hydrochloride and for control medium also with 1.1494253 mM arginine) was used and was present throughout the entire stimulation. For experiments with BMDMs from *Csf2rb*^*fl/fl*^*Ccr2*-Cre^ER^^T2^ and *Csf2rb*^+/+^*Ccr2*-Cre^ER^^T2^ control mice, 4-hydroxytamoxifen was given at a final concentration of 2 μM in full medium for the duration of the differentiation.

### Flow cytometry and cell sorting

For analysis of spinal cord cell populations, stainings were performed in PBS using fixable viability dye (Invitrogen, 65-0865-14, 1:2,000, 10 min at room temperature) as well as the following antibodies: CD45.2-BV650 (clone 104, Biolegend, 109836, 1:100), Ly6G-BV605 (clone 1A8, Biolegend, 127639, 1:100), Ly6G-AF700 (clone 1A8, Biolegend, 127622, 1:100), CD3-BV605 (clone 145-2C11, Biolegend, 100351, 1:100), Ly6C-BV605 (clone HK14, Biolegend, 128035, 1:100), Ly6C-BV510 (clone HK14, Biolegend, 128033, 1:100), CD11b-BV510 (clone M1/70, Biolegend, 101245, 1:100), CD11b-PB (clone M1/70.15, Invitrogen, RM2828, 1:100), CD11b-BV605 (clone M1/70, Biolegend, 101237, 1:100), CX3CR1-PeCy7 (clone SA011F11, Biolegend, 149015, 1:100), CCR2-BV421 (clone SA203G11, Biolegend, 150605, 1:100), CCR2-FITC (clone SA203G11, Biolegend, 150607, 1:100), F4/80-BV510 (clone BM8, Biolegend, 123135, 1:100), F4/80-BV605 (clone BM8, Biolegend, 123133, 1:100), F4/80-FITC (clone BM8, Biolegend, 123108, 1:100), CD11c-PE-Cy5.5 (clone N418, Invitrogen, 35-0114-82, 1:100), P2RY12-APC (clone S16007D, Biolegend, 848006, 1:100), P2RY12-AF488 (clone S16007D, Biolegend, 848016, 1:100), MerTK-APC (clone 2B10C42, Biolegend, 151507, 1:100), CD44-FITC (clone IM7, Invitrogen, 11-0441-82, 1:100), CD44-eF506 (clone IM7, Invitrogen, 69-0441-82, 1:100), CD64-BV605 (clone X54-5/7.1, Biolegend, 139323, 1:100), CD3-AF700 (clone 17A2, Biolegend, 100216, 1:100), CD3-rF710 (clone 17A2, Tonbo, 80-0032-U100, 1:80), CD8-BV510 (clone 53-6.7, Biolegend, 100751, 1:80), CD4-APC (clone REA604, Miltenyi Biotec, 130-116-487, 1:80), CD4-PE (clone REA604, Miltenyi Biotec, 130-116-509, 1:80) and CD25-BV421 (clone PC61, Biolegend, 102033, 1:80). Surface staining was performed for 20 min at 21 °C. Intracellular staining was performed using a Fixation and Permeabilization Buffer Set (eBioscience, 88-8824-00), True-Phos Buffer Set (Biolegend, 425401) or Foxp3/Transcription Factor Staining Buffer Set (Invitrogen, 00-5523-00) according to the manufacturer’s instructions and included antibodies specific to ARG1-PE (clone A1exF5, Invitrogen, 12-3697-80, 1:50), ARG1-APC (clone A1exF5, Invitrogen, 17-3697-82, 1:50), iNOS-PE (clone CXNFT, Invitrogen, 12-5920-82, 1:100), iNOS-PE-Cy5.5 (clone CXNFT, Invitrogen, 12-5920-80, 1:50), IL-10-APC (clone JES5-16E3, Invitrogen, 17-7101-82, 1:50), TGFβ1-PE (clone TW7-16B4, Biolegend, 141403, 1:50), HO-1-AF647 (clone EPR18161-128, Abcam, ab237268, 1:50), IL-17-PE (clone REA660, Miltenyi Biotec, 130-112-009, 1:50), IFNγ-FITC (clone REA638, Miltenyi Biotec, 130-117-780, 1:50) and FoxP3-APC (clone 3G3, Tonbo, 20-5773, 1:100) or isotype control rat IgG2a κ-APC (clone eBR2a, Invitrogen, 17-4321-81, 1:50), rat IgG2a κ-PE (clone eBR2a, Invitrogen, 12-4321-80, 1:50), rat IgG2a κ-PE (clone eBR2a, Invitrogen, 12-4321-41, 1:100), rat IgG2a κ-PE-Cy5.5 (clone eBR2a, Invitrogen, 35-4321-82, 1:50), rat IgG2b κ-APC (clone eB149/10H5, Invitrogen, 17-4031-81, 1:50), mouse IgG1 κ-PE (clone P3.6.2.8.1, Invitrogen, 12-4714-81, 1:50), rabbit anti-goat IgG-AF647 (Invitrogen, A21446, 1:50), REA control antibody-APC (clone REA293, Miltenyi Biotec, 130-113-446, 1:80), REA control antibody-FITC (clone REA293, Miltenyi Biotec, 130-113-449, 1:50), REA control antibody-PE (clone REA293, Miltenyi Biotec, 130-118-347, 1:50) and rat IgG1 κ-APC (clone P3.6.2.8.1, eBioscience, 17-4714-41, 1:100). Intracellular staining was performed overnight at 4 °C. For cell sorting of ARG1^+^ and ARG1^−^ cells, isolated cells from the spinal cord were filtered through a 40-μm cell strainer and sorted as viable CD3^−^Ly6G^−^CD45^+^CX3CR1^+^ARG1^+^/CD3^−^Ly6G^−^CD45^+^CX3CR1^+^ARG1^−^ cells. For analysis of *Csf2rb* expression within Mdcs from *Csf2rb*^*fl/fl*^*Ccr2*-Cre^ER^^T2^ and *Csf2rb*^+/+^*Ccr2*-Cre^ER^^T2^ control mice by quantitative real-time PCR with reverse transcription, isolated cells from the spinal cord were filtered through a 40-μm cell strainer and sorted as viable, CD45^+^CD3^−^Ly6G^−^CD11b^+^Ly6C^+^CX3CR1^+^ cells directly into Eppendorf tubes containing RLT Plus buffer.

For analysis of tdTomato expression within Ly6C^hi^ blood monocytes by flow cytometry, EDTA-treated blood was stained with antibodies and fixed with 2% paraformaldehyde for 15 min. The cell suspension was then subjected to red blood cell lysis and resuspended in PBS + 1% FCS before proceeding to flow cytometric analysis. The following antibodies were used: Ly6G-AF-700 (clone 1A8, Biolegend, 127622, 1:200), Ly6C-BV605 (clone HK14, Biolegend, 128035, 1:80), CD11b-PE-Cy7 (clone M1/70, eBioscience, 25-0112-82, 1:400), B220-PE-Cy5 (clone RA3-6B2, Tonbo, 55-0452-U100, 1:100) and CD3-PerCP-Cy5.5 (clone 145-2C11, Tonbo, 65-0031-U025, 1:100).

For analysis of BMDMs by flow cytometry, cells were detached using CellStripper (Corning, 15313661). Staining of detached cells was performed in PBS using fixable viability dye (Invitrogen, 65-0865-14) as well as the following antibodies: F4/80-BV605 (clone BM8, Biolegend, 123133, 1:80), F4/80-BV421 (clone BM8, Biolegend, 123137, 1:80), CX3CR1-PeCy7 (clone SA011F11, Biolegend, 149015, 1:100) and CD11b-BV510 (clone M1/70, Biolegend, 101245, 1:80). Intracellular staining was performed using a Fixation and Permeabilization Buffer Set (eBioscience, 88-8824-00), according to the manufacturer’s instructions, and included antibodies specific to ARG1-APC (clone A1exF5, Invitrogen, 17-3697-82, 1:100) and iNOS-PE (clone CXNFT, Invitrogen, 12-5920-82, 1:100) or isotype control rat IgG2a κ-APC (clone eBR2a, Invitrogen, 17-4321-81, 1:100) and rat IgG2a κ-PE (clone eBR2a, Invitrogen, 12-4321-41, 1:100).

For analysis of lipid content using LipidTox (Invitrogen, H34477, 1:150 in PBS), cells were stained for 25 min at 37 °C. Levels of lipid peroxidation were measured using C11-BODIPY (Invitrogen, D3861, 1:5,000 in PBS, 30 min at 37 °C) and were determined by dividing the mean fluorescence intensity of the FITC channel (oxidized lipids) by the sum of the mean fluorescence intensity of the FITC and PE channels (reduced lipids, together the total amount of lipids within a cell), which results in the ratio of oxidized/total lipids. For detection of cellular oxidative stress, CellRox (Invitrogen, C10422 or C10444, 1:2,000 in PBS, 30 min at 37 °C) was used.

Compensation was performed on single-stained samples of cells. All stained samples were acquired on a CytoFLEX S cytometer (Beckman Coulter), and results were analyzed using CytExpert (v2.5) and FlowJo software (v10.8.1). Cell sorting was performed on a FACSAria Fusion flow cytometer (BD Biosciences).

### Lesion characterization and ARG1 immunohistochemistry of human samples

MS lesions were assessed on 2-μm cut sections using Luxol fast blue and CD68 immunohistochemistry staining as previously described^83,84^. White matter MS lesions were identified by marked myelin loss as determined by Luxol fast blue staining and categorized following previously established criteria^36,37^. Briefly, active lesions were identified by an accumulation of CD68^+^ macrophages and microglia, which frequently contain Luxol fast blue products. Chronic active lesions showed a demyelinated lesion center and an accumulation of CD68^+^ myeloid cells, occasionally containing myelin degradation products, at the lesion rim. Inactive lesions were identified by a hypocellular and demyelinated lesion center, showing no pronounced infiltration of CD68^+^ cells to the center or the rim. Remyelinated lesions were not examined in this study. Lesions were subdivided into separate zones. The lesion center is surrounded by the lesion rim, a distinct border, where myelin increases toward the level of the normal-appearing white matter. The periplaque white matter is a 1-cm-wide zone that transitions into the normal-appearing white matter. ARG1 levels within the aforementioned lesions were assessed by immunohistochemistry (polyclonal ARG1, Invitrogen, PA5-85267, sodium citrate buffer, 1:100) as previously described^83,84^.

### Immunofluorescence staining of human samples and image acquisition

For immunofluorescent co-staining of ARG1 and CD68 in human MS samples, a modified protocol for double labeling was used to enhance the ARG1 signal. After incubation, the first primary antibody was developed by a biotinylated tyramine precipitate and subsequently inactivated by a second heat steaming step with sodium citrate buffer^83,84^. Briefly, after dewaxing, the slides were steamed at 80 °C using sodium citrate buffer at pH 6.0 for 40 min. Endogenous peroxidase was then blocked using Peroxidase Blocking Solution from Dako (SDS151) for 10 min. After 10 min of 10% FCS exposure, slides were incubated with primary anti-ARG1 (polyclonal, Invitrogen, PA5-85267, 1:100) overnight at 4 °C. The next day, a biotinylated secondary antibody (K675, Dako, SDS391) was applied for 30 min. Following this step, the slides were incubated with streptavidin-linked horseradish peroxidase (K675, Dako, SDS315) for 30 min, and the catalyzed signal amplification kit by Dako (K1500) was applied for 20 min. Afterward, the slides were steamed in sodium citrate buffer at 80 °C for 30 min and incubated with anti-CD68 (clone KP1, Dako, M0814, 1:500) overnight at 4 °C. Labeling was accomplished by exposure to streptavidin-linked Cy3 (Jackson ImmunoResearch, 016-160-084, 1:1,000) and secondary Alexa Fluor 488-linked anti-mouse IgG (polyclonal, Jackson ImmunoResearch, 115-545-166, 1:800) for 1 h at 21 °C. Nuclei were stained with DAPI (Thermo Fisher Scientific, D1306, 300 nM), and the slides were coverslipped using an aqueous mounting medium (Polysciences, 18606). Images were acquired with an Olympus BX63 microscope and exported as TIFF files. Figure composition and processing was performed with ImageJ^85^ and GIMP (https://www.gimp.org).

For immunofluorescent co-stainings of ARG1 and iNOS with markers for myeloid cells (IBA1), astrocytes (GFAP) and neurons (SMI32), formaldehyde-fixed paraffin-embedded 2-μm-thick tissue slides were deparaffinized in xylene for 20 min. Slides were rehydrated, and endogenous peroxidase activity was blocked using 0.9% hydrogen peroxide in methanol. Heat-induced epitope retrieval was performed in citrate buffer (GV80511, Agilent Dako) using a heater (PT200, Agilent Dako). Aldehyde-induced autofluorescence was blocked by 1% (wt/vol) sodium borohydride in TBS twice for 2 min each. Nonspecific protein binding was blocked by applying 10% normal goat serum (16210-064, Gibco) in PBS for 30 min. Primary antibodies from distinct host species (anti-NOS2, 1:5,000, rabbit, PA3-030A, Invitrogen; anti-ARG1, 1:50, mouse, PA5-85267, Invitrogen) were applied overnight in a commercially available antibody diluent (S2022, Agilent Dako) at 4 °C. Fluorophore-labeled secondary antibodies (Cy3 anti-rabbit, 1:1,000, goat, 711-485-152, Jackson ImmunoResearch; AF488 anti-mouse, 1:800, goat, 115-545-166, Jackson ImmunoResearch) were applied for 90 min in Dako antibody diluent at 21 °C. The third primary antibody (anti-IBA1, 1:300, rabbit, 019-19741, FujiFilm Wako; anti-GFAP, 1:1,000, rabbit, Z0334, Agilent Dako; anti-neurofilament H, clone SMI32, 1:400, mouse, 801701, Sternberger Monoclonals) was then linked to a fluorophore using species-specific CoraLite Plus 647 labeled FlexAble kits (KFA503 and KFA523, Proteintech), according to the producer’s recommendations, and applied overnight at 4 °C. Counterstaining of nuclei was performed using 1 μg ml^−1^ DAPI (D1306, Invitrogen) in TBS for 5 min. Slides were coverslipped using a water-based mounting medium (18606, Polysciences) and stored at 4 °C.

Images of fluorescence multilabelings were acquired on an Olympus BX63 microscope with a ×100 objective and Olympus Cellsense software. Rolling ball background subtraction and bleaching correction were performed in Fiji and ImageJ2 v2.16.0 (refs. ^85–87^). Horizontal reconstruction of the images was done on *z* stacks with 0.1-μm steps in ImageJ2. For quantification of IBA1, whole slides were imaged at ×10 and stitched together. Consecutively, 1 × 1 mm regions of interest were selected and exported. Cell detection was performed using the cyto3 model^88^ in the cellpose GUI^89^ on the DAPI and IBA1 channels. Cell labels were transformed to regions of interest in Fiji and imported to QuPath v0.5.1 (refs. ^90,91^). The individual channels were thresholded for each slide, and a built-in combination classifier was applied.

### scRNA-seq analysis

scRNA-seq data from CNS cells during EAE are available under accession number GSE130119, and the expression matrix, metadata and co-ordinates were kept as previously described^45^. Clustered data were visualized using *t*-SNE plots, and, of the 22 clusters originally identified^45^, myeloid cell-specific clustering (clusters 0–4 and 18) was performed with *Arg1* expression identified in cluster 2. Cluster 2 was further subclustered into *Slc7a2*^*hi*^ and *Slc7a2*^*lo*^, and differentially expressed genes between *Slc7a2*^*hi*^ and all other myeloid cells were determined using the Seurat R package v4.3.0. Subsequently, GSEA of the differentially expressed genes ranked according to their average log_2 _(fold change) values was performed, and normalized enrichment scores were determined for the most significant pathways (FDR < 0.05) related to GO biological process using the fgsea R package^92^.

### Targeted metabolomics of CSF and spinal cord tissue

Metabolite profiling was conducted at the Van Andel Institute Mass Spectrometry Core. Polar metabolites were extracted by the Bligh and Dyer method^93^ from CSF (40 μl of CSF per 1 ml final extraction volume) and spinal cord tissue (80 mg of tissue per 1 ml final extraction volume). Spinal cord tissue was mechanically dissociated in a bead mill homogenizer. The top aqueous layer was collected, dried in a rotary vacuum and resuspended in water (40 μl for CSF and 100 μl for the spinal cord). Metabolites were profiled using two orthogonal liquid chromatography methods on an Agilent 6470 triple-quadrupole mass spectrometer. First, central carbon metabolites, including amino acids, tricarboxylic acid cycle intermediates and other organic acids, nucleotides/nucleosides, sugar phosphates and others, were profiled using ion-paired chromatography in ESI-negative mode, as described previously^94^. Note, this ion-paired chromatography method is incompatible with positive-mode analysis and requires a dedicated liquid chromatography system. If ion-paired and standard methods are to be used on the same mass spectrometer, extensive source cleaning is required between methods. Next, additional metabolites of interest, including acyl-carnitines, kynurenine and related intermediates and *S*-adenosylmethionine/*S*-adenosylhomocysteine amenable to ESI-positive mode, were analyzed by reversed-phase chromatography (Cortecs T3, 2.1 × 150 mm, 1.6 μM, 186008500, Waters). For this method, mobile phase A was 0.1% formic acid (vol/vol), and mobile phase B was 90% acetonitrile with 0.1% formic acid (vol/vol). The gradient was as follows: 0–2.0 min, 100% A at 0.4 ml min^−1^; 2.0–7.1, ramp to 100% B at 0.4 ml min^−1^; 7.1–8.0 min, hold at 99% B. The column was then re-equilibrated in 100% A at 0.6 ml min^−1^ for 2 min. Full liquid chromatography–mass spectrometry parameters for both methods and raw peak-area data are available in Supplementary Table 1.

### Targeted metabolomics data analysis

Metabolomics data were log_2_ transformed and scaled by using the following *z*-score formula: *z* = (*x* − *μ*) / *σ*, where *x* is metabolite abundance for one sample, *μ* is mean abundance for all samples, and *σ* is the population standard deviation. Metabolite profiles across experimental groups (Fig. 1c) were identified by soft clustering using the Mfuzz R package v2.56.0 (ref. ^95^) under default parameters except that the fuzzifier was set to 1.3137 for spinal cord and 1.3099 for CSF. Significant metabolites (FDR < 0.15) between experimental groups in both the spinal cord and CSF were determined using two two-tailed *t*-tests or one-way ANOVA, and *P* values were adjusted for multiple hypothesis testing using the Benjamini and Hochberg method. Metabolite set enrichment analysis was performed using Metaboanalyst v5.0 (ref. ^96^) and an FDR of <0.10. Metabolites were classified as strong or weak metabolites based on *P* value results from the different experimental groups (healthy, peak and recovery) using a one-way ANOVA followed by a Tukey’s honestly significant difference post-test (95% confidence level). Those metabolites passing a double threshold (*P* < 0.05 in two types of comparisons between the experimental groups) were grouped as strong metabolites whereas those passing only one threshold (one comparison between the experimental groups) were grouped as weak metabolites. Strong metabolites were subsequently subcategorized into DLMs, RLMs or HLMs, with DLMs significant between health and peak disease and between health and recovery, RLMs significant between health and recovery and between peak disease and recovery and HLMs significant between health and peak disease and between peak disease and recovery. Clusters of significant data from comparisons were depicted as heat maps using hierarchical clustering with Euclidean distance. For the average effect analysis examining impacts of ARG1 deficiency in CX3CR1^+^ cells at peak disease, mean *z* scores from *Arg1*^*fl/fl*^*Cx3cr1*-Cre mice were subtracted from mean *z* scores from *Arg1*^*fl/fl*^ animals and plotted against the mean *z* scores of healthy animals subtracted from mean *z* scores of animals with peak disease.

### Statistical analysis

Data collection and analysis were not performed blind to the conditions of the experiments, and mice were grouped randomly per cage. Data distribution was assumed to be normal, but this was not formally tested. No statistical methods were used to predetermine sample size, but our sample sizes were similar to those generally used in EAE studies.

Statistical analyses were performed by using two-tailed *t*-tests, an ordinary one-way ANOVA followed by a Tukey’s or Dunnett’s post-test, simple linear regression or two-way ANOVA followed by a Sidak’s post-test with Prism 9.4.1 software (GraphPad), unless otherwise stated. *P* values of clinical course AUC were determined by two-tailed *t*-test. Statistical significance is indicated by *P < 0.05, **P < 0.01, ***P < 0.001 and ****P < 0.001. All error bars indicate ±s.e.m.

### Reporting summary

Further information on research design is available in the Nature Portfolio Reporting Summary linked to this article.

## Online content

Any methods, additional references, Nature Portfolio reporting summaries, source data, extended data, supplementary information, acknowledgements, peer review information; details of author contributions and competing interests; and statements of data and code availability are available at 10.1038/s41590-026-02516-4.

## Supplementary information

Supplementary Information Supplementary Fig. 1 and Supplementary Tables 1–8.

Reporting Summary

Peer Review file

Supplementary Data Supplementary Methods and Supplementary References.

## Source data

Source Data Fig. 1 Statistical source data.

Source Data Fig. 2 Statistical source data.

Source Data Fig. 3 Statistical source data.

Source Data Fig. 4 Statistical source data.

Source Data Fig. 5 Statistical source data.

Source Data Fig. 6 Statistical source data.

Source Data Extended Data Fig. 1 Statistical source data.

Source Data Extended Data Fig. 2 Statistical source data.

Source Data Extended Data Fig. 3 Statistical source data.

Source Data Extended Data Fig. 3 Unprocessed western blots.

Source Data Extended Data Fig. 4 Statistical source data.

Source Data Extended Data Fig. 5 Statistical source data.

Source Data Extended Data Fig. 6 Statistical source data.

Source Data Extended Data Fig. 7 Statistical source data.

Source Data Extended Data Fig. 8 Statistical source data.

Source Data Extended Data Fig. 9 Statistical source data.

Source Data Extended Data Fig. 10 Statistical source data.

## Data Availability

Bulk RNA-seq data generated in this study have been deposited in the Gene Expression Omnibus under accession number GSE231474. The results published here are in part based on analysis of publicly available scRNA-seq data deposited under Gene Expression Ominbus data set GSE130119. Source data are provided with this paper.

## Code availability

All code used in this study is available on GitHub at https://github.com/osharif-lab/EAE-Arg1.
