# Supplementary material for: Monocyte infiltration induces CNS arginine catabolism to fuel neuroinflammation
Source: Nat Immunol. 2026 May 18;27(7):1418–32. doi: 10.1038/s41590-026-02516-4 (PMC13310764; doi:10.1038/s41590-026-02516-4)
Supplement: Supplementary file 1 — Supplementary Fig. 1 and Supplementary Tables 1–8. [file 41590_2026_2516_MOESM1_ESM.pdf]

nature immunology

Supplementary information https://doi.org/10.1038/s41590-026-02516-4

Monocyte infiltration induces CNS arginine catabolism to fuel neuroinflammation

In the format provided by the authors and unedited

Supplementary Figure 1

a

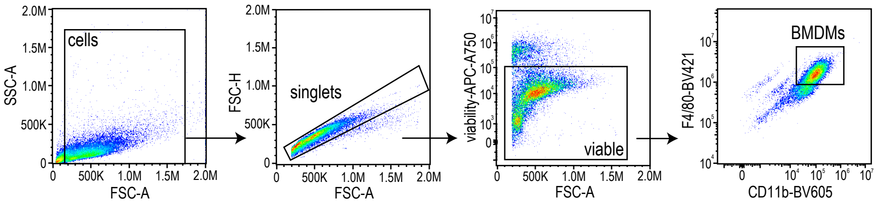

b

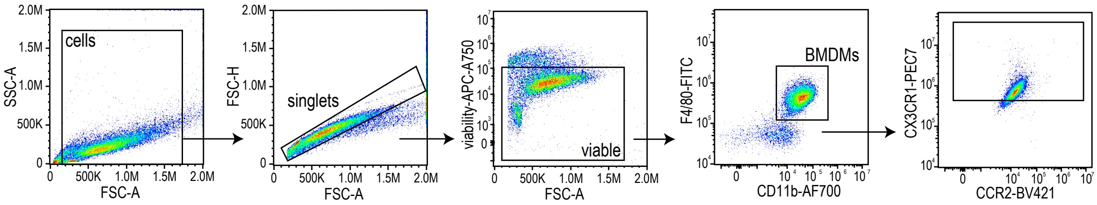

Supplementary Figure 1, Gating strategies for BMDMs

a, Gating strategy for BMDMs in **Extended Data Fig. 7b/c** **b**, Gating strategy for CX3CR1+ BMDMs in **Extended Data Fig. 7g/h** .

Cells were pre-gated on single viable CD11b+F4/80+ (**a, b**).

Supplementary Table 1. Transition list for metabolites measured with reversed phase and ion paired chromatography for targeted metabolomics.

RT, Retention Time

Reversed Phase Transition List								
**Name**	**RT**	**Ion Polarity**	**Transition**	**Collision Energy**	**Fragmentor**	**Transition**	**Collision Energy**	**Fragmentor**
1-methylnicotinamide_3106-60-3_C02918	0,905	Positive	137.0 -> 94.1	20	120	137.0 -> 92.0	20	120
S-Adenosyl-L-Methionine_485-80-3_C00019	0,906	Positive	399.2 -> 250.0	16	120	399.2 -> 136.0	32	120
L-carnitine_541-15-1_C00318	0,911	Positive	162.1 -> 60.2	20	120	162.1 -> 103.0	20	120
NicotinamideMononucleotide_1094-617_C00455	1,061	Positive	335.1 -> 123.0	12	95	335.1 -> 80.1	60	95
NicotinicAcid_59-67-6_C00253	1,263	Positive	124.0 -> 78.1	30	80	124.0 -> 80.1	20	80
Methionine_63-68-3_C00073	1,351	Positive	150.0 -> 56.0	20	60	150.0 -> 61.0	20	60
O-Acetylcarnitine_3040-38-8_C02571	1,404	Positive	204.1 -> 85.0	20	120	204.1 -> 145.0	10	120
QuinolinicAcid_89-00-9_C03722	1,454	Positive	168.0 -> 78.0	24	82	168.0 -> 124.0	8	82
Nicotinamide_98-92-0_C00153	1,555	Positive	123.1 -> 80.0	24	105	123.1 -> 53.0	36	105
OphthalmicAcid_495-27-2_C21016	1,589	Positive	290.1 -> 58.2	20	80	290.1 -> 215.0	10	80
Nudifloramide_701-44-0_C05842	1,887	Positive	153.1 -> 108.0	24	135	153.1 -> 78.0	36	135
3-Hyroxykynurenine_606-14-4_C03227	1,924	Positive	225.0 -> 110.0	16	92	225.0 -> 162.0	20	92
NN-Dimethyltryptamine_61-50-7_C08302	2,349	Positive	189.1 -> 189.1	0	82	189.1 -> 144.1	10	82

PicolinicAcid_98-98-6_C10164	2,450	Positive	124.0 -> 78.0	20	82	124.0 -> 106.0	8	82
Propionylcarnitine_17298-37-2_C03017	3,132	Positive	218.1 -> 159.0	10	120	218.1 -> 144.1	20	120
S-Adenosyl-L-Homocysteine_979-920_C00021	3,171	Positive	385.1 -> 136.0	20	115	385.1 -> 134.0	20	115
N-Methyl-4-Pyridone-3-Carboxamide_76949-3_C05843	3,373	Positive	153.1 -> 136.0	16	85	153.1 -> 92.0	28	85
N-methylNicotinamide_114-33-0_C05843	3,382	Positive	137.1 -> 53.2	20	120	137.1 -> 80.1	24	115
Glutarylcarnitine_102636-82-8_NA	3,522	Positive	276.1 -> 199.0	15	160	276.1 -> 216.9	15	160
Kynurenine_2922-83-0_C00328	4,103	Positive	209.0 -> 209.0	0	92	209.0 -> 146.0	20	92
ISTD-D8-Tryptophan_NA_NA	4,614	Positive	212.0 -> 195.0	10	50	212.0 -> 150.0	20	80
Methylmalonate_516-05-2_C02170	4,628	Positive	119.1 -> 73.1	10	80	119.1 -> 55.1	20	80
Butyrylcarnitine_1492-26-8_C02862	4,662	Positive	232.1 -> 57.1	45	120	232.1 -> 144.1	20	120
Isobutyrylcarnitine_25518-49-4_NA	4,670	Positive	232.1 -> 85.0	20	120	232.1 -> 173.0	10	120
NN-Dimethylserotonin_487-93-4_C08299	4,761	Positive	205.0 -> 205.0	0	80	205.0 -> 184.1	5	80
Tryptophan_73-22-3_C00078	4,761	Positive	205.0 -> 146.0	16	82	205.0 -> 118.0	32	82
ISTD-13C11-Tryptophan_202114-65-6_NA	4,769	Positive	216.0 -> 155.0	15	50	216.0 -> 169.0	15	50
3-hydroxyanthranillic acid_548-93-6_C00632	4,821	Positive	154.0 -> 108.0	20	114	154.0 -> 154.0	0	114
KynurenicAcid_492-27-3_C01717	4,900	Positive	190.0 -> 144.0	20	104	190.0 -> 116.0	36	104
NicotinuricAcid_583-08-4_C05380	4,925	Positive	181.1 -> 135.0	20	115	181.1 -> 79.1	48	115

Tryptamine_61-54-1_C00398	5,048	Positive	161.1 -> 91.1	40	60	161.1 -> 115.0	40	60
2-Methylbutyryl carnitine_31023-25-3_NA	5,075	Positive	246.0 -> 60.2	20	120	246.0 -> 144.1	20	120
Isovalerylcarnitine_31023-24-2_C20826	5,075	Positive	246.1 -> 187.0	15	80	246.1 -> 85.1	20	80
Serotonin_50-67-9_C00780	5,340	Positive	177.1 -> 115.0	40	82	177.1 -> 77.1	60	82
NicotinamideN-oxide_1986-81-8_NA	5,350	Positive	139.0 -> 78.0	36	139	139.0 -> 106.0	24	139
AnthranillicAcid_5326-47-6_NA	5,423	Positive	138.0 -> 92.0	20	80	138.0 -> 65.0	30	80
N-methylTryptamine_61-49-4_C06213	5,462	Positive	175.1 -> 132.0	12	80	175.1 -> 144.0	12	80
NicotinicAcidMononucleotide_321-028_C01185	5,481	Positive	337.1 -> 125.0	16	95	337.1 -> 97.0	20	95
N-methylserotonin_1134-01-6_C06212	5,493	Positive	191.0 -> 115.0	40	80	191.0 -> 160.0	20	80
Hexanoylcarnitine_22671-29-0_NA	5,530	Positive	260.2 -> 201.0	15	80	260.2 -> 99.1	20	120
Octanoylcarnitine_25243-95-2_C02838	6,216	Positive	288.1 -> 229.1	10	160	288.1 -> 144.1	20	160
Decanoylcarnitine_1492-27-9_C03299	6,756	Positive	316.2 -> 257.1	20	160	316.2 -> 155.2	20	160
LipoicAcid_1200-22-2_C16241	7,046	Negative	205.0 -> 127.0	15	150	205.0 -> 171.0	5	100
Lauroylcarnitine_25518-54-1_NA	7,147	Positive	344.3 -> 285.2	20	160	344.3 -> 183.0	20	200
Myristoylcarnitine_25597-07-3_NA	7,595	Positive	372.3 -> 313.2	20	200	372.3 -> 229.2	20	200
Palmitoylcarnitine_2364-67-2_C02990	7,929	Positive	400.2 -> 95.0	30	200	400.2 -> 341.2	20	160
Oleoylcarnitine_38677-66-6_NA	7,986	Positive	426.0 -> 85.0	30	100	426.0 -> 144.0	30	50

Stearoylcarnitine_25597-09-5_NA	8,288	Positive	428.3 -> 267.1	20	120	428.3 -> 369.2	20	120

Ion Paired Transition List

Name	RT	Ion Polarity	Transition	Collision Energy	Fragmentor	Transition	Collision Energy	Fragmentor
D-+-Galactosamine	1,096	Negative	238.1 -> 159.9	8	70	238.1 -> 100.0	6	70
Thiamine	1,12	Negative	264.1 -> 234.0	6	124			
Pyridoxamine	1,139	Negative	167.1 -> 121.0	22	102	167.1 -> 122.0	18	102
L-Histidine	1,148	Negative	154.1 -> 93.1	16	102	154.1 -> 137.0	12	102
L-asparagine	1,21	Negative	131.0 -> 113.0	4	86	131.0 -> 70.0	12	86
L-Carnitine	1,214	Negative	220.1 -> 146.0	4	66			
L-Cystathionine	1,214	Negative	221.1 -> 134.0	8	92	221.1 -> 120.0	9	92
L-Serine	1,22	Negative	104.0 -> 74.1	8	70			
S-2-Aminoethyl-L-cysteine	1,224	Negative	163.1 -> 76.1	10	88	163.1 -> 33.2	28	88
L-Glutamine	1,226	Negative	145.1 -> 127.0	7	94	145.1 -> 109.0	10	94
L-Cystine	1,239	Negative	239.0 -> 120.0	4	74	239.0 -> 74.1	20	74
trans-4-Hydroxy-L-proline	1,252	Negative	130.1 -> 128.0	12	100	130.1 -> 66.0	16	100
D-Xylose	1,259	Negative	149.1 -> 89.0	4	68	149.1 -> 71.0	4	68

L-Homoserine	1,261	Negative	118.0 -> 55.0	16	70	118.0 -> 72.1	10	70
L-Threonine	1,27	Negative	118.0 -> 74.1	9	72			
myo-Inositol	1,273	Negative	239.1 -> 179.0	0	72	179.0 -> 71.0	12	72
Taurine	1,278	Negative	124.0 -> 80.0	24	122			
D-Mannose	1,29	Negative	179.1 -> 89.0	4	66	179.1 -> 59.2	16	66
gamma-Aminobutyric acid	1,296	Negative	102.0 -> 84.1	8	64			
L-Arabitol	1,309	Negative	151.0 -> 89.0	12	110	151.0 -> 71.0	16	110
Xylitol	1,309	Negative	151.1 -> 89.0	8	96	151.1 -> 71.0	8	96
L-Citrulline	1,317	Negative	174.1 -> 131.0	8	88	174.1 -> 42.2	48	88
Cellobiose	1,322	Negative	341.1 -> 161.0	5	68			
D-Maltose	1,322	Negative	341.1 -> 161.0	5	72	341.1 -> 179.0	4	72
Melibiose	1,322	Negative	341.1 -> 179.0	4	76	341.1 -> 89.0	13	76
N-Acetyl D-galactosamine	1,333	Negative	220.1 -> 119.0	0	68			
Creatine	1,337	Negative	130.1 -> 88.1	8	66	130.1 -> 41.2	36	66
Trehalose	1,339	Negative	401.1 -> 341.0	10	98	341.0 -> 89.0	12	98
L-Proline	1,346	Negative	114.0 -> 68.1	12	94	114.0 -> 45.2	12	94

Allantoin	1,351	Negative	157.0 -> 97.0	12	76	157.0 -> 42.2	16	76
L-Canavanine	1,351	Negative	175.1 -> 74.0	10	74	175.1 -> 118.0	8	74
Cytosine	1,355	Negative	110.0 -> 67.1	8	58	110.0 -> 42.2	12	58
4-Guanidobutyric acid	1,362	Negative	144.1 -> 102.0	8	74	144.1 -> 41.2	32	74
Creatinine	1,363	Negative	112.0 -> 41.2	24	92	112.0 -> 68.1	16	92
L-Arginine	1,393	Negative	173.1 -> 131.0	11	90	173.1 -> 156.0	8	90
L-Homocysteine	1,422	Negative	134.0 -> 116.9	8	70			
L-Arabinose	1,437	Negative	149.0 -> 59.2	12	66	149.0 -> 89.1	4	66
L-Homocystine	1,441	Negative	267.0 -> 115.0	16	92	267.0 -> 132.0	8	92
2-Deoxy-D-ribose	1,485	Negative	193.1 -> 59.2	16	64	193.1 -> 133.0	0	64
L-Sorbose	1,536	Negative	179.1 -> 89.0	4	66	179.1 -> 71.0	14	66
Ribonic acid gamma lactone	1,539	Negative	147.0 -> 59.2	16	88	147.0 -> 99.0	8	88
D-Glucosamine 6-phosphate	1,543	Negative	258.0 -> 79.0	48	182	258.0 -> 97.0	16	182
Uracil	1,634	Negative	111.0 -> 42.2	14	86			
O-Phosphorylethanolamine	1,708	Negative	140.0 -> 79.0	14	122	140.0 -> 63.0	38	122
Cytidine	1,771	Negative	242.1 -> 109.0	8	104	242.1 -> 42.2	16	104

L-Methionine	1,775	Negative	148.0 -> 47.2	12	70	148.0 -> 100.0	6	70
Hypoxanthine	1,903	Negative	135.0 -> 92.0	17	104	135.0 -> 65.1	28	104
2-Deoxycytidine	1,956	Negative	286.1 -> 226.0	4	64	286.1 -> 93.0	20	64
Guanine	1,978	Negative	150.0 -> 133.0	12	116	150.0 -> 66.0	36	116
Xanthine	2,054	Negative	151.0 -> 108.0	16	104	151.0 -> 42.2	24	104
L-Isoleucine	2,099	Negative	130.0 -> 45.0	16	96			
L-Leucine	2,099	Negative	130.0 -> 45.0	16	96			
L-Tyrosine	2,213	Negative	180.0 -> 119.0	15	114	180.0 -> 93.0	12	114
Uridine	2,22	Negative	243.1 -> 200.0	6	128	243.1 -> 110.0	12	128
AICAR	2,312	Negative	257.1 -> 125.0	10	118	257.1 -> 42.2	44	118
3-Hydroxy-DL-kynurenine	2,339	Negative	223.1 -> 162.0	10	88	223.1 -> 205.9	4	88
Pyridoxal hydrochloride	2,399	Negative	166.1 -> 138.0	12	104	166.1 -> 108.0	18	104
Pyridoxine	2,425	Negative	168.1 -> 166.0	9	92	168.1 -> 122.1	17	92
2-Deoxyuridine	2,615	Negative	227.1 -> 184.0	6	116	227.1 -> 42.2	20	116
5-Methoxytryptamine	2,718	Negative	189.1 -> 174.0	12	92	189.1 -> 144.0	28	92
Adenine	2,763	Negative	134.0 -> 107.0	18	118	134.0 -> 92.1	20	118

Thymine	2,764	Negative	125.0 -> 42.2	14	86			
±-Mevalonolactone	2,894	Negative	189.1 -> 59.1	6	48			
Inosine	3,151	Negative	267.1 -> 135.0	22	144	267.1 -> 108.0	44	144
beta-Nicotinamide mononucleotide	3,401	Negative	333.0 -> 251.1	12	60	333.0 -> 135.1	36	60
2-Deoxyinosine	3,412	Negative	251.1 -> 135.0	20	124	251.1 -> 108.0	42	124
Guanosine	3,72	Negative	282.1 -> 150.0	17	152	282.1 -> 132.9	32	152
L-Kynurenine	3,747	Negative	207.1 -> 190.0	4	76	207.1 -> 144.0	20	76
2-Deoxyguanosine	4,006	Negative	266.1 -> 150.0	12	74	266.1 -> 108.0	36	74
DL-2-Aminoadipic acid	4,077	Negative	160.1 -> 142.0	9	96	160.1 -> 116.0	12	96
L-Phenylalanine	4,194	Negative	164.1 -> 147.0	9	100	164.1 -> 103.0	15	100
Argininosuccinic acid	4,222	Negative	289.0 -> 131.1	27	98	289.0 -> 88.0	36	98
L-Glutamic acid	4,287	Negative	146.0 -> 102.0	11	76	146.0 -> 128.0	8	76
L-Aspartic Acid	4,488	Negative	132.0 -> 88.0	10	76	132.0 -> 71.0	14	76
4-Hydroxy-L-glutamic acid	4,68	Negative	162.0 -> 144.0	6	72	162.0 -> 72.1	16	72
Glucoheptonic acid	4,986	Negative	225.1 -> 129.0	11	98	225.1 -> 75.1	18	98
S-5-Adenosyl-L-homocysteine	4,992	Negative	383.1 -> 134.0	24	130	383.1 -> 248.0	13	130
Thymidine	5,027	Negative	241.1 -> 42.2	16	122	241.1 -> 151.0	6	122

D-Gluconic acid	5,23	Negative	195.1 -> 75.2	18	104	195.1 -> 129.0	10	104
Galactonic acid	5,23	Negative	195.1 -> 75.1	18	94	195.1 -> 129.0	11	94
Uric acid	5,526	Negative	167.0 -> 124.0	13	128	167.0 -> 42.3	26	128
Glyceric acid	5,628	Negative	105.0 -> 75.1	8	70	105.0 -> 57.2	14	70
Glyoxylic acid	5,906	Negative	73.0 -> 45.2	6	48			
N-Acetylneuraminic acid	5,932	Negative	308.1 -> 87.0	14	94	308.1 -> 169.9	10	94
Quinic acid	6,063	Negative	191.1 -> 85.1	25	124	191.1 -> 93.1	24	124
O-Succinyl-L-homoserine	6,154	Negative	218.1 -> 117.0	7	68	218.1 -> 118.0	0	68
Adenosine	6,227	Negative	266.0 -> 107.0	44	122	266.0 -> 134.0	10	72
Shikimic acid	6,231	Negative	173.0 -> 111.0	7	132	173.0 -> 93.1	16	132
2-Deoxyadenosine	6,478	Negative	310.1 -> 250.0	4	74	250.0 -> 134.1	12	74
Oxamic acid	6,502	Negative	88.0 -> 44.3	6	44	88.0 -> 42.3	12	44
Chorismic acid	6,691	Negative	225.0 -> 189.0	6	68			
Succinic semialdehyde	6,964	Negative	101.0 -> 57.2	7	72			
Lactic acid	7,225	Negative	89.0 -> 43.3	10	48	89.0 -> 45.3	9	48
3-Dehydroshikimic acid	7,316	Negative	171.0 -> 127.0	8	86	171.0 -> 109.0	17	86
alpha-D(+)Mannose 1-phosphate	7,37	Negative	259.0 -> 97.0	14	100	259.0 -> 79.0	48	100

Creatine phosphate	7,424	Negative	210.0 -> 79.0	16	70	210.0 -> 96.9	4	70
L-Dihydroorotic acid	7,502	Negative	157.0 -> 113.0	5	116	157.0 -> 42.2	30	116
alpha-D-Glucose-1-phosphate	7,589	Negative	259.0 -> 79.0	28	126	259.0 -> 240.9	9	126
D-Glucose 6-phosphate	7,597	Negative	259.0 -> 97.0	14	102	259.0 -> 79.0	48	102
L-Tryptophan	7,761	Negative	203.1 -> 116.0	14	116	203.1 -> 74.1	14	116
D-Ribose 5-phosphate	7,784	Negative	229.0 -> 79.0	48	96	229.0 -> 97.0	10	96
D-Sedoheptulose-7-phosphate	7,891	Negative	289.0 -> 96.9	18	104	289.0 -> 79.0	48	104
Trehalose 6-phosphate	7,923	Negative	421.1 -> 241.0	27	176			
gamma-Glu-Cys	7,935	Negative	249.1 -> 128.0	8	94	249.1 -> 171.0	9	94
D-Fructose 6-phosphate	7,942	Negative	259.0 -> 97.0	14	102	259.0 -> 79.0	48	102
Xanthosine	8,093	Negative	283.1 -> 151.0	18	126	283.1 -> 108.0	40	126
D-Xylulose-5-phosphate	8,322	Negative	229.0 -> 79.0	36	86	229.0 -> 139.0	8	86
N-Acetyl-D-glucosamine 6-phosphate	8,672	Negative	300.0 -> 97.0	18	86	300.0 -> 79.0	48	86
Nicotinic acid mononucleotide	8,73	Negative	334.0 -> 289.9	4	70	334.0 -> 79.0	40	70
Orotic acid	8,921	Negative	155.0 -> 42.0	26	68	155.0 -> 40.0	44	68
2-Deoxy-D-glucose 6-phosphate	8,927	Negative	243.0 -> 79.1	45	96	243.0 -> 97.0	14	96

6-Hydroxynicotinic acid	9,048	Negative	138.0 -> 94.1	10	72	138.0 -> 42.3	27	72
N-Acetyl-alpha-D-glucosamine 1-phosphate	9,11	Negative	300.0 -> 97.0	16	132	300.0 -> 79.0	36	132
D-erythro-Dihydrosphingosine	9,118	Negative	300.3 -> 199.0	8	110	300.3 -> 282.0	6	110
4-Quinolinol	9,241	Negative	144.0 -> 116.0	28	160	144.0 -> 66.1	44	160
2-Deoxyribose 5-phosphate	9,247	Negative	213.0 -> 79.1	40	92	213.0 -> 96.9	12	92
beta-Nicotinamide adenine dinucleotide	9,452	Negative	662.1 -> 540.0	12	70	662.1 -> 327.9	36	70
2-Methyl-1-butanol	9,468	Negative	87.1 -> 43.3	5	38			
Pyruvic acid	9,468	Negative	87.0 -> 43.2	4	48			
Cytidine-5-monophosphate	9,512	Negative	322.0 -> 79.0	44	130	322.0 -> 97.0	22	130
o-Phospho-L-Serine	9,911	Negative	184.0 -> 79.0	36	132	184.0 -> 97.0	13	132
4-Aminobenzoic acid	9,921	Negative	136.0 -> 92.0	8	72			
2-Deoxycytidine 5-monophosphate	10,235	Negative	306.0 -> 194.9	14	118	306.0 -> 79.0	44	118
Glutathione (reduced)	10,235	Negative	306.1 -> 195.0	16	60	306.1 -> 110.0	20	60
Dihydroxyacetone phosphate	10,591	Negative	169.0 -> 97.0	6	128	169.0 -> 79.0	32	128
Uridine 5-monophosphate	10,594	Negative	323.0 -> 79.1	48	126	323.0 -> 97.0	24	126
2-3-Dihydroxyisovalerate	10,618	Negative	133.1 -> 75.1	10	88	133.1 -> 57.2	15	88

Inosine 5-monophosphate	10,904	Negative	347.0 -> 79.0	44	150	347.0 -> 97.0	22	150
5-Deoxy-5-(methylthio)adenosine	11,717	Negative	356.1 -> 296.0	4	70	356.1 -> 134.0	12	70
DL-Glyceraldehyde 3-phosphate	11,814	Negative	169.0 -> 79.0	28	130	169.0 -> 97.0	4	130
Nicotinic acid	11,816	Negative	122.0 -> 78.1	11	60	122.0 -> 51.2	32	60
3-Hydroxyanthranilic acid	11,891	Negative	152.0 -> 108.0	12	86	152.0 -> 107.0	23	86
2-Deoxyguanosine 5-monophosphate	11,97	Negative	346.0 -> 79.1	48	122	346.0 -> 97.0	36	122
Adenosine 5-monophosphate	11,97	Negative	346.0 -> 79.0	38	146	346.0 -> 97.0	24	146
D-pantothenic acid	11,997	Negative	218.0 -> 146.1	14	70	218.0 -> 88.0	10	70
4-Hydroxybenzoic acid	12,009	Negative	137.0 -> 93.0	14	88	137.0 -> 65.2	34	88
Guanosine 3,5-cyclic monophosphate	12,171	Negative	344.0 -> 150.0	24	104	344.0 -> 133.0	44	104
Prephenic acid	12,333	Negative	225.0 -> 101.0	9	84			
2-Ketobutyrate	12,396	Negative	101.0 -> 57.2	4	64			
Riboflavin	12,523	Negative	375.1 -> 255.0	14	170	375.1 -> 212.0	28	170
Vanillic acid	12,586	Negative	167.0 -> 123.0	9	82	167.0 -> 108.0	18	82
Maleic acid	12,648	Negative	115.0 -> 71.2	7	62	115.0 -> 27.3	15	62
Epicatechin	12,677	Negative	289.1 -> 245.0	11	128	289.1 -> 109.0	26	128

2-Deoxyadenosine 5-monophosphate	12,693	Negative	330.0 -> 79.0	48	128	330.0 -> 134.1	28	128
Succinic acid	12,842	Negative	117.0 -> 73.1	10	62	117.0 -> 99.0	8	62
Isopentenyl pyrophosphate	12,913	Negative	245.0 -> 209.0	4	52			
5-Hydroxy-3-indoleacetic acid	12,939	Negative	190.1 -> 144.0	21	74	190.1 -> 116.0	46	74
N-Formyl-L-Tyrosine	12,939	Negative	208.1 -> 164.0	7	122	208.1 -> 107.0	12	122
Malonic acid	13,002	Negative	103.0 -> 59.2	7	56	103.0 -> 41.3	32	56
N-Carbamoyl-DL-aspartic acid	13,378	Negative	175.0 -> 132.0	7	76	175.0 -> 88.1	19	76
Adenosine 3-5-cyclic monophosphate	13,385	Negative	328.0 -> 134.0	24	154	328.0 -> 79.0	48	154
L-Gluthathione (oxidized)	13,433	Negative	611.1 -> 306.1	24	152	611.1 -> 272.1	28	152
N-Carbamyl-L-glutamic acid	13,454	Negative	189.1 -> 102.1	20	78	189.1 -> 146.0	7	78
Citramalic acid	13,507	Negative	147.0 -> 87.0	14	76	147.0 -> 85.1	12	76
m-Hydroxybenzoic acid	13,508	Negative	137.0 -> 93.1	10	92	137.0 -> 65.2	28	92
L-Malic acid	13,601	Negative	133.0 -> 115.0	8	76	133.0 -> 71.1	14	76
4-Methyl-2-oxovaleric acid	13,677	Negative	129.1 -> 85.1	7	74			
Itaconic acid	13,677	Negative	129.0 -> 85.1	6	68	129.0 -> 41.3	12	68
L-Hydroxyglutaric acid	13,692	Negative	147.0 -> 128.9	7	74	147.0 -> 85.1	13	74

Mevalonic acid	13,692	Negative	147.1 -> 128.9	8	52	147.1 -> 103.1	11	52
2-2-Dimethyl Succinic acid	13,802	Negative	145.1 -> 101.1	11	88	145.1 -> 127.1	8	88
Adipic acid	13,802	Negative	145.1 -> 101.1	11	78	145.1 -> 83.1	12	78
N-acetyl aspartate	13,886	Negative	174.0 -> 88.0	16	60	174.0 -> 58.1	20	60
N-Acetylglutamic acid	13,978	Negative	188.1 -> 128.0	10	86	188.1 -> 102.0	16	86
Uridine 5'-diphosphogalactose	14,092	Negative	565.0 -> 322.9	24	180	565.0 -> 384.9	29	180
Uridine 5-diphosphoglucose	14,092	Negative	565.0 -> 322.9	24	152	565.0 -> 384.9	28	152
3-Hydroxyphenylacetic acid	14,139	Negative	151.0 -> 107.1	4	58	151.0 -> 65.2	26	58
3-Methylglutaric acid	14,182	Negative	145.1 -> 101.1	11	82	145.1 -> 41.3	52	82
alpha-Ketoglutaric acid	14,182	Negative	145.0 -> 101.0	5	70	145.0 -> 57.2	9	70
4-Hydroxyphenyl-pyruvic acid	14,256	Negative	179.0 -> 107.0	4	68			
Cytidine 5-diphosphate	14,287	Negative	402.0 -> 79.0	48	108	402.0 -> 158.9	24	108
Isopentyl acetate	14,293	Negative	129.1 -> 85.2	6	134	129.1 -> 41.2	12	134
Ketovaleric acid	14,505	Negative	115.0 -> 71.2	4	70			
3-phosphoglyceric acid	14,552	Negative	185.0 -> 79.0	30	80	185.0 -> 97.0	30	80
trans-trans Muconic acid	14,563	Negative	141.0 -> 53.3	8	64			

D-Ribulose 1,5-biphosphate	14,601	Negative	308.9 -> 97.0	22	80	308.9 -> 79.0	46	80
2-3-Dihydroxybenzoic acid	14,63	Negative	153.0 -> 109.0	15	96	153.0 -> 53.2	25	96
Mevalonic acid 5-phosphate	14,66	Negative	227.0 -> 79.0	40	88	227.0 -> 97.0	12	88
Uridine 5-diphosphate	14,684	Negative	403.0 -> 158.9	28	108	403.0 -> 79.0	48	108
Guanosine 5-diphosphate	14,709	Negative	442.0 -> 150.0	31	144	442.0 -> 158.9	20	144
2-Deoxycytidine 5-diphosphate	14,752	Negative	386.0 -> 97.0	20	106	386.0 -> 79.0	36	106
2-3-Pyridinedicarboxylic acid	14,798	Negative	166.0 -> 122.0	6	68	166.0 -> 78.1	13	68
Pyridoxal 5 phosphate	14,804	Negative	246.0 -> 79.0	30	70			
D-Fructose 1,6-biphosphate	14,871	Negative	338.9 -> 241.0	12	90	338.9 -> 97.0	22	90
trans-Aconitic acid	14,924	Negative	173.0 -> 129.0	4	58	173.0 -> 85.1	10	58
Citric acid	15,015	Negative	191.0 -> 111.0	10	78	191.0 -> 87.0	16	78
DL-Isocitric acid	15,015	Negative	191.0 -> 111.0	11	74	191.0 -> 173.0	6	74
2-4-Quinolinediol	15,102	Negative	160.0 -> 42.2	29	120	160.0 -> 117.9	19	120
Folinic acid	15,13	Negative	472.2 -> 343.0	20	160			
Phosphoenolpyruvic acid	15,186	Negative	167.0 -> 79.0	12	118			
Inosine 5-diphosphate	15,215	Negative	427.0 -> 158.9	28	90	427.0 -> 135.0	24	90

Homocitrate	15,218	Negative	205.0 -> 125.0	10	96	205.0 -> 145.0	10	96
2-Deoxyguanosine 5-diphosphate	15,224	Negative	426.0 -> 158.9	28	102	426.0 -> 79.1	48	102
Adenosine 5-diphosphate	15,224	Negative	426.0 -> 159.0	28	56	426.0 -> 328.0	16	56
4-Pyridoxic acid	15,227	Negative	182.0 -> 138.0	12	84	182.0 -> 108.1	20	84
cis-Aconitic acid	15,253	Negative	173.0 -> 129.0	4	62	173.0 -> 85.1	8	62
Thymidine 5-diphosphate	15,267	Negative	401.0 -> 79.0	44	114	401.0 -> 97.0	25	114
3-2-Hydroxyethylindole	15,431	Negative	160.1 -> 142.1	16	90	160.1 -> 130.1	14	90
2-Isopropylmalic acid	15,464	Negative	175.1 -> 113.0	13	92	175.1 -> 115.0	13	92
2-Deoxyadenosine 5-diphosphate	15,621	Negative	410.0 -> 79.1	44	158	410.0 -> 158.7	24	158
Indoline-2-carboxylate	16,208	Negative	162.1 -> 116.0	16	114	162.1 -> 118.0	11	114
Adenylosuccinic acid	16,314	Negative	462.0 -> 97.0	24	148	462.0 -> 134.1	48	148
Ketoisovaleric acid	16,487	Negative	115.0 -> 71.2	4	70			
3-Indoleacetic acid	16,528	Negative	174.1 -> 130.0	7	74	174.1 -> 128.0	19	74
o-Hydroxy hippuric acid	16,561	Negative	194.0 -> 150.0	11	86	194.0 -> 121.0	22	86
Lipoamide	16,721	Negative	204.1 -> 158.0	4	70	204.1 -> 64.1	20	70
Salicylic acid	16,74	Negative	137.0 -> 93.1	17	78	137.0 -> 65.2	33	78
UDP-Gluceronic Acid	16,751	Negative	579.0 -> 323.0	20	156	579.0 -> 158.8	40	156

2-Deoxyuridine 5-triphosphate	16,753	Negative	467.0 -> 158.8	36	144	467.0 -> 368.9	20	144
Deoxycytidine 5-triphosphate	16,778	Negative	466.0 -> 158.9	28	142	466.0 -> 367.9	20	142
Cytidine 5-triphosphate	16,828	Negative	482.0 -> 158.8	40	142	482.0 -> 79.0	40	142
Flavin adenine dinucleotide	16,927	Negative	784.1 -> 437.0	30	148	784.1 -> 346.0	35	148
Guanosine 5-triphosphate	16,937	Negative	522.3 -> 158.9	36	102	522.3 -> 424.0	20	102
PRPP	16,948	Negative	389.0 -> 291.0	12	60	389.0 -> 176.7	20	60
Uridine 5-triphosphate	16,988	Negative	483.0 -> 158.9	36	146	483.0 -> 385.0	20	146
Inosine 5-triphosphate	17,156	Negative	507.0 -> 159.0	40	124	507.0 -> 409.0	19	124
Adenosine 5-triphosphate	17,165	Negative	506.0 -> 159.0	38	122	506.0 -> 408.1	22	122
Deoxyguanosine 5-triphosphate	17,165	Negative	506.0 -> 158.8	32	150	506.0 -> 407.9	20	150
Deoxyadenosine 5-triphosphate	17,173	Negative	490.0 -> 391.9	24	148	490.0 -> 158.9	32	148
Deoxythymidine 5-triphosphate	17,208	Negative	481.0 -> 158.8	36	130	481.0 -> 383.1	20	130
Phenylpyruvic acid	17,278	Negative	163.0 -> 91.1	6	68			
Coenzyme A	17,812	Negative	766.0 -> 686.0	40	135	766.0 -> 419.0	40	135
Acetyl-Coenzyme A	17,996	Negative	808.0 -> 461.0	40	135	808.0 -> 728.0	40	135
Acetoacetyl-Coenzyme A	18,012	Negative	850.0 -> 686.0	40	135	850.0 -> 766.0	40	135

3-Hydroxybutyryl-Coenzyme A	18,128	Negative	852.0 -> 505.0	40	135				
Malonyl-Coenzyme A	18,145	Negative	852.0 -> 808.0	40	135	852.0 -> 728.0	40	135	
Methylmalonyl-Coenzyme A	18,203	Negative	866.0 -> 822.0	40	135	866.0 -> 742.0	40	135	
Succinyl-Coenzyme A	18,211	Negative	866.0 -> 519.0	40	135	866.0 -> 786.0	40	135	
Palmitoyl-Coenzyme A	18,32	Negative	1004.0 -> 924.0	40	135	1004.0 -> 657.0	40	135	
Proprionyl-Coenzyme A	18,534	Negative	822.0 -> 475.0	40	135	822.0 -> 742.0	40	135	
Butyryl-Coenzyme A	18,861	Negative	836.0 -> 756.0	40	135	836.0 -> 489.0	40	135	
Isovaleryl-Coenzyme A	19,002	Negative	850.0 -> 770.0	40	135	850.0 -> 503.0	40	135	
Taurocholic acid	20,659	Negative	514.0 -> 514.0	50	150				
Glycocholic acid	20,751	Negative	464.0 -> 74.0	40	215	464.0 -> 402.0	40	215	
Chenodeoxycholic acid	21,312	Negative	391.0 -> 373.0	36	190				
Glycodeoxycholic acid	21,427	Negative	448.0 -> 74.0	40	195	448.0 -> 386.0	40	195	

Supplementary Table 2. Reversed Phase and Ion Paired MS parameters for targeted metabolomics MS, Mass spectrometry

Reversed Phase MS parameters	
Mass Spectrometer	Agilent 6470
Ion Source	AJS ESI
Polarity	Value (+)
Gas Temp (°C)	200
Gas Flow (l/min)	13
Nebulizer (psi)	45
SheathGasHeater	325
SheathGasFlow	12
Capillary (V)	3000
VCharging	500
**Ion Paired MS parameters**	
Mass Spectrometer	Agilent 6470
Ion Source	AJS ESI
Polarity	Value (-)
Gas Temp (°C)	150
Gas Flow (l/min)	13
Nebulizer (psi)	45
SheathGasHeater	325
SheathGasFlow	12
Capillary (V)	2000
VCharging	500

Supplementary Table 3. Reversed phase LC parameters

LC, liquid chromatography

**Quaternary pump G7104A**	
Flow	0.400 mL/min
Use Solvent Types	Yes
Low Pressure Limit	0.00 bar
High Pressure Limit	1200.00 bar
Max. Flow Ramp Up	100.000 mL/min²
Primary Channel	Automatic
Max. Flow Ramp Down	100.000 mL/min²
Stroke	
Automatic Stroke Calculation	Yes
Mixer Selection	
Selected Mixer	Do not use Mixer
Blend Assist	
Enabled	No
Stop Time	
Stoptime Mode	Time set
Stoptime	11.00 min
Post Time	
Posttime Mode	Off

LC Solvents

Channel	Solvent Composition	Used
A	100% H_2O, w/ 0.1% Formic Acid	Yes
B	90:10 Acetonitrile:H_2O, 0.1% Formic Acid	Yes
C	100% H_2O	No
D	100% Isopropanol	No

LC gradient program

Time (min)	A (%)	B (%)	C (%)	D (%)	Flow (mL/min)	Pressure (bar)
2.00 min	100.00 %	0.00 %	0.00 %	0.00 %	--- mL/min	--- bar
7.10 min	1.00 %	99.00 %	0.00 %	0.00 %	--- mL/min	--- bar
8.00 min	1.00 %	99.00 %	0.00 %	0.00 %	0.400 mL/min	--- bar
8.10 min	100.00 %	0.00 %	0.00 %	0.00 %	0.600 mL/min	--- bar

10.00 min	100.00 %	0.00 %	0.00 %	0.00 %	0.600 mL/min	--- bar
10.10 min	100.00 %	0.00 %	0.00 %	0.00 %	0.400 mL/min	--- bar
11.00 min	100.00 %	0.00 %	0.00 %	0.00 %	--- mL/min	--- bar

**Column Compartment G7116B**	
Temperature Control Mode	Temperature Set
Temperature	50.0 °C
Enable Analysis Left Temperature	
Enable Analysis Left Temperature On	No
Right Temperature Control	
Right temperature Control Mode	Combined
Enable Analysis Right Temperature	
Enable Analysis Right Temperature On	No
Enforce column for run	
Enforce column for run enabled	No
Stop Time	
Stoptime Mode	As pump/injector
Post Time	
Posttime Mode	Off
Valve Position	Position 2 (Port 1 -> 2)
Ready when front door open	Yes
Position Switch After Run	Do not switch

Supplementary Table 4. Ion Paired LC parameters

LC, liquid chromatography

Quaternary pump G7104A	
Flow	0.250 mL/min
Use Solvent Types	Yes
Low Pressure Limit	0.00 bar
High Pressure Limit	1200.00 bar
Max. Flow Ramp Up	100.000 mL/min²
Primary Channel	Automatic
Max. Flow Ramp Down	100.000 mL/min²
Stroke	
Automatic Stroke Calculation	Yes
Mixer Selection	
Selected Mixer	Do not use Mixer
Blend Assist	
Enabled	No
Stop Time	
Stoptime Mode	Time set
Stoptime	40.00 min
Post Time	
Posttime Mode	Off

LC Solvents

Channel	Solvent Composition	Used
A	97:3 Aq:MeOH w/ 10mM TBA, 15mM Acetic Acid	Yes
B	100% Isopropanol	No
C	97:3 MeOH:H20 w/ 10mM Tributylamine, 15mM Acetic Acid	Yes
D	99% ACN	Yes

LC gradient program

Time (min)	A (%)	B (%)	C (%)	D (%)	Flow (mL/min)	Pressure limit (bar)
2.50 min	100.00 %	0.00 %	0.00 %	0.00 %	0.250 mL/min	1200.00 bar
7.50 min	80.00 %	0.00 %	20.00 %	0.00 %	0.250 mL/min	1200.00 bar
13.00 min	55.00 %	0.00 %	45.00 %	0.00 %	0.250 mL/min	1200.00 bar
20.00 min	1.00 %	0.00 %	99.00 %	0.00 %	0.250 mL/min	1200.00 bar

24.00 min	1.00 %	0.00 %	99.00 %	0.00 %	0.250 mL/min	1200.00 bar
24.05 min	1.00 %	0.00 %	0.00 %	99.00 %	0.250 mL/min	1200.00 bar
27.00 min	1.00 %	0.00 %	0.00 %	99.00 %	0.250 mL/min	1200.00 bar
27.50 min	--- %	--- %	0.00 %	99.00 %	0.800 mL/min	1200.00 bar
31.35 min	--- %	--- %	0.00 %	99.00 %	0.800 mL/min	1200.00 bar
31.50 min	1.00 %	0.00 %	0.00 %	99.00 %	0.600 mL/min	1200.00 bar
32.25 min	100.00 %	0.00 %	0.00 %	0.00 %	0.400 mL/min	1200.00 bar
39.90 min	100.00 %	0.00 %	0.00 %	0.00 %	0.400 mL/min	1200.00 bar
40.00 min	100.00 %	0.00 %	0.00 %	0.00 %	0.250 mL/min	1200.00 bar

**Column Compartment G7116B**	
Temperature Control Mode	Temperature Set
Temperature	35.0 °C
Enable Analysis Left Temperature	
Enable Analysis Left Temperature On	Yes
Enable Analysis Left Temperature Value	0.8 °C
Left Temp. Equilibration Time	0.0 min
Right Temperature Control	
Right temperature Control Mode	Combined
Enable Analysis Right Temperature	
Enable Analysis Right Temperature On	Yes
Enable Analysis Right Temperature Value	0.8 °C
Right Temp. Equilibration Time	0.0 min
Enforce column for run	
Enforce column for run enabled	No
Stop Time	
Stoptime Mode	As pump/injector
Post Time	
Posttime Mode	Off
Valve Position	Position 2 (Port 1 -> 2)
Ready when front door open	Yes
Position Switch After Run	Do not switch

**Time (min)**	**Function**	**Parameter**
24.00 min	Change Valve Position	Column at Position 1

39.00 min	Change Valve Position	Column at Position 2

Supplementary Table 5: Metabolite trajectories of CNS-associated metabolites during neuro-inflammation

Table represents membership degree of the specified metabolites to their assigned cluster. A high membership degree represents a strong affiliation to the assigned cluster. SC, Spinal Cord; CSF, cerebrospinal fluid.

SC Cluster 1

Metabolite	Membership Degree
Citrate	0,7590
Methylbutyrylcarnitine	0,7586
Isovalerylcarnitine	0,7574
Isobutyrylcarnitine	0,7542
N-Acetylgalactosamine	0,7440
Nudifloramide	0,7439
Butyrylcarnitine	0,7427
CTP	0,7411
Xanthosine	0,7386
Oleoylcarnitine	0,7365
Hexanoylcarnitine	0,7358
UricAcid	0,7243
Octanoylcarnitine	0,7190
Thymidine	0,7165
L-arnitine	0,7159
O-Acetylcarnitine	0,7128
N-alpha-acetylglutamine	0,7058
ATP	0,7044
alpha-N-Acetyl-Lysine	0,6982
trans-4-Hydroxy-proline	0,6851
Taurine	0,6691
Quinolinic Acid	0,6615
GTP	0,6546
CoA	0,6513
Glutarylcarnitine	0,6500
Itaconic Acid	0,6353
Dimethyltryptamine	0,6306
Malate	0,6305
Deoxyuridine	0,6283
UDP-Glucuronate	0,6266
Dihydrosphingosine	0,6243
Anthranillic Acid	0,6212
Fumarate	0,6030
CDP	0,6006
Decanoylcarnitine	0,5920

CSF Cluster 1

Metabolite	Membership Degree
Isovalerylcarnitine	0,7179
Tryptophan	0,7099
Uric Acid	0,7070
Quinolinic Acid	0,7062
Pyruvate	0,7053
Pyridoxal	0,6996
Allantoin	0,6962
Riboflavin	0,6793
Methylbutyrylcarnitine	0,6766
Histidine	0,6598
Orotic Acid	0,6521
O-Phosphorylethanolamine	0,6185
Citrulline	0,6092
N-Carbamoyl-aspartic acid	0,6087
Pantothenic Acid	0,6057
alpha-Ketoglutaric Acid	0,5966
Hydroxyindoleacetate	0,5930
Threonine	0,5650
Homocitrate	0,5641
Methylnicotinamide	0,5563
N-Methyl-4-Pyridone-3-Carboxamide	0,5498
Nicotinic Acid	0,5299
Mannose-1-phosphate	0,5132
Cytidine	0,5121
Isobutyrylcarnitine	0,5096
Argininosuccinate	0,4977
Glutarylcarnitine	0,4957
Indoxyl-3-sulfate	0,4903
Malate	0,4861
Fructose-6-phosphate	0,4855
Methylcitric Acid	0,4810
Butyrylcarnitine	0,4806
Fumarate	0,4773
Itaconic Acid	0,4629
Thymidine	0,4579

Tyrosine	0,5858
O-Phosphorylethanolamine	0,5844
Propionylcarnitine	0,5820
Palmitoylcarnitine	0,5779
alpha-Ketoglutaric Acid	0,5647
UDP-N-acetylgalactosglucosamine	0,5519
Guanidinobutyric Acid	0,5313
ITP	0,5217
Pantothenic Acid	0,5187
Tryptophan	0,5136
Allantoin	0,5088
Lauroylcarnitine	0,4956
UTP	0,4947
IMP	0,4926
UDP	0,4603
N-Acetyl-glucosamine-6phosphate	0,4508
Isoleucine	0,4416
Methylnicotinamide	0,4360
Indoxyl-3-sulfate	0,4355
UDP-glucose	0,4309
UDP-galactose	0,4301
N-Methyl-4-Pyridone-3Carboxamide	0,4128
Pyridoxic Acid	0,3846
Acetyl-CoA	0,3811
cAMP	0,3765
Nicotinamide Mononucleotide	0,3761
Glutathione Oxidized	0,3754
FAD	0,3630
Pyridoxal	0,3533
ShikimicAcid	0,3451

Xanthosine	0,4502
Serine	0,4480
Phenylalanine	0,4478
Lactate	0,4465
Propionylcarnitine	0,4390
UDP-Glucuronate	0,4385
UDP-N-acetylgalactosglucosamine	0,4290
Glutamine	0,4246
Asparagine	0,4151
Isoleucine	0,4073
Kynurenine	0,4069
N-alpha-acetyl-glutamine	0,4051
N-Acetylglutamate	0,4033
Methionine	0,3955
Citrate	0,3728
Quinic Acid	0,3657

CSF Cluster 2

**Metabolite**	**Membership Degree**
Dihydroxyacetone Phosphate	0,6241
Adenosine	0,6222
Creatine	0,6124
Hyroxykynurenine	0,6089
Methylthioadenosine	0,5933
N-Acetyl-Glycine	0,5898
Adenine	0,5788
Methylmalonate	0,5750
Glutathione Oxidized	0,5629

SC Cluster 2	
**Metabolite**	**Membership Degree**
Histidine	0,6821
S-Adenosyl-Methionine	0,6804
Methylthioadenosine	0,6804
Ophthalmate	0,6788
dCMP	0,6693
Methylcitric Acid	0,6682
Methionine	0,6527
Aminoadipic Acid	0,6526
alpha-Glucose-1-phosphate	0,6504
alpha-N-Acetyl-L-Arginine	0,6462
Fructose-1-6-biphosphate	0,6430
Uracil	0,6404
Cytidine	0,6392
N-Acetyl-L-Glycine	0,6307
Cystathionine	0,6211
Pyruvate	0,6125
Mannose-1-phosphate	0,6028
UMP	0,5993
N-Acetylglutamate	0,5733
dTTP	0,5678
dAMP	0,5604
D-Sedoheptulose-7phosphate	0,5489
Serine	0,5477
PRPP	0,5388
Phenylalanine	0,5387
Creatinine	0,5555
UTP	0,5400
N-Acetyl-valine	0,5357
N-Carbamyl_-glutamic acid	0,5118
UMP	0,5025
NAD	0,4999
Taurine	0,4854
IMP	0,4778
Glutathione Reduced	0,4728
Glutamate	0,4650
D-Maltose	0,4577
Leucine	0,4573
O-Acetylcarnitine	0,4486
L-arnitine	0,4408
alpha-Ribose-5-phosphate	0,4403
S-Adenosyl-Homocysteine	0,4372
AMP	0,4369
Gluconate	0,4266
Anthranillic acid	0,4245
Inosine	0,4189
UDP-glucose	0,4106
cAMP	0,3997
Uracil	0,3981
Phosphoglycerate	0,3976
Xanthine	0,3927
dTDP	0,3834
N-Acetyl-Aspartate	0,3735
Tyrosine	0,3654
Nicotinamide mononucleotide	0,3645

Hydroxyglutaric acid	0,5366
Glucose-6-phosphate	0,5285
CMP	0,5077
Methylmalonic Acid	0,5074
Fructose-6-phosphate	0,5065
Pyridoxal-5-phosphate	0,4969
Xanthine	0,4845
N-Acetyl-alpha-glucosamine1-phosphate	0,4835
Succinate	0,4688
Kynurenine	0,4687
Phosphoglycerate	0,4641
Threonine	0,4593
O-Phospho-Serine	0,4586
Argininosuccinate	0,4488
Orotic Acid	0,4456
Citrulline	0,4446
Ophthalmic Acid	0,4358
Homocitrate	0,4303
Hydroxyindoleacetate	0,4301
Dihydroxyacetone Phosphate	0,3864
Deoxy-glucose-6-phosphate	0,3505

SC Cluster 3	
**Metabolite**	**Membership Degree**
Creatine	0,7599
N-Acetyl-Aspartate	0,7575
N-Carbamoyl-aspartic acid	0,7496
dTDP	0,7455
ADP	0,7423
S-Adenosyl-Homocysteine	0,7083
L-Arabitol-Xylitol	0,6838
Adenosine	0,6754
Mannose	0,6740
Sorbose	0,6550
Guanosine	0,6522
AMP	0,6520
GDP	0,6420
Adenine	0,6224
Asparagine	0,6198
myo-Inositol	0,6144
Lactate	0,6007
Inosine	0,5864

CSF Cluster 3	
**Metabolite**	**Membership Degree**
Xylose	0,6574
L-Arabitol-Xylitol	0,6526
Mannose	0,6486
N-Acetylneuraminic Acid	0,6480
Arginine	0,6412
Sorbose	0,6401
Shikimic Acid	0,6148
S-Adenosyl-Methionine	0,6048
Arabinose	0,5588
myo-Inositol	0,5263
Glyceric Acid	0,5193
Guanosine	0,4641
Cystine	0,4537
N-Acetylglucosamine	0,4508
Nicotinamide-N-oxide	0,4135
Hypoxanthine	0,3936
Nudifloramide	0,3920
Thiamine	0,3876
Uridine	0,3863
Pyridoxal-5-phosphate	0,3785
trans-4-Hydroxy-proline	0,3679
Deoxyuridine	0,3577

Glutamate	0,5725
alpha-Ribose-5-phosphate	0,5589
Creatinine	0,5539
N-Acetylneuraminic Acid	0,5385
Arginine	0,5317
Leucine	0,5314
Glutathione Reduced	0,5313
Nicotinamide	0,5296
NicotinicAcid	0,5285
N-Carbamyl-glutamic acid	0,5143
NAD	0,5094
Guanine	0,5047
Uridine	0,4992
GDP-Mannose	0,4963
Xylulose-5-phosphate	0,4938
Trehalose	0,4793
gamma-Glu-Cys	0,4633
Methylmalonate	0,4569
Hypoxanthine	0,4458
Thiamine	0,4339
Adenylosuccinate	0,4299
IDP	0,4120
Maltose	0,3925
N-Acetylglucosamine	0,3687
Glutamine	0,3590
N-methyl-Nicotinamide	0,3528

Supplementary Table 6. m/z and RT of derivatized polyamines

Compound	Derivatized m/z	Retention Time
ISTD-D8Tryptophan	213.1474	2.5
N1-N8-diacetylspermidine	330.2387	3.2
N-acetylputrescine	231.1703	3.3
Arginine	375.2238	3.4
Gamma-Aminobutyrate	104.0706	3.4
N-acetylcadaverine	245.186	3.5
N1N12 diacetylspermine	487.349	4.3
Ornithine	333.202	4.4
N8-Acetylspermidine	388.2806	4.8
N1-Acetylspermidine	388.2806	4.8
1,3 Diaminopropane	275.1965	5.1
Putrescine	289.2122	5.3
Cadaverine	303.2278	5.7
ISTD-16Diaminohexane	317.2435	6.2
N1-Acetylspermine	545.3909	6.4
N-(3-oxopropyl) acetamide	316.1755	7
Spermidine	446.3225	7.1
Spermine	603.4327	8.4

Supplementary Table 7. MS patient characteristics and controls used in Fig.3

RRMS, relapsing remitting multiple sclerosis; SPMS, secondary progressive multiple sclerosis; AMS, acute MS; PPMS, primary progressive multiple sclerosis

Case ID	patient ID	Sex	Clinical Course	age	Disease Duration (month)	Lesion Localization	preDMTera	LesionType
18-78	1	m	RRMS	65	25x12	supratentorial	yes	inactive
328-78	2	m	SPMS	34	2x12	supratentorial	yes	active
434-79	3	f	PPMS	30	7x12	supratentorial	yes	chronic active
241-84	4	f	RRMS	46	18x12	supratentorial	yes	inactive
73-85	5	m	AMS	28	6	supratentorial	yes	active
215-86	6	f	RRMS	78	26x12	supratentorial	yes	inactive
251-87	7	f	RRMS	59	37x12	supratentorial	yes	chronic active
7-02	8	f	AMS	37	7	infratentorial	no	active
116-04	9	f	AMS	40	6	supratentorial	no	active
19-05	10	f	unknown	44	n.a.	infratentorial	no	active
75-09	11	f	unknown	76	n.a.	supratentorial	no	inactive
187-09	12	f	PPMS	46	n.a.	supratentorial	no	chronic active
217-10	13	f	PMS	69	n.a.	infratentorial	no	inactive
78-11	14	f	unknown	56	n.a.	supratentorial	no	chronic active
82-15	15	f	SPMS	61	n.a.	infratentorial	no	chronic active
101-15	16	m	RRMS	52	24x12	supratentorial	no	chronic active
35-16	17	f	SPMS	50	>10x12	supratentorial	no	chronic active
194-16	18	m	SPMS	73	n.a.	supratentorial	no	inactive

62-04	19	m	Control	51	-	supratentorial	-	-
79-04	20	m	Control	58	-	supratentorial	-	-
175-04	21	m	Control	77	-	supratentorial	-	-
53-06	22	f	Control	37	-	supratentorial	-	-
195-07	23	f	Control	62	-	supratentorial	-	-
160-08	24	f	Control	86	-	supratentorial	-	-
150-09	25	m	Control	72	-	supratentorial	-	-

Supplementary Table 8. Statistical matrix for comparisons of populations between different treatment conditions depicted in **Ext Fig. 8b**

**24 hours**						**48 hours**					
**Arg1-iNOS-**	US	IL4/IL13	LPS/IFNg	LPS/GMCSF	LPS/IFNg/GMCSF	**Arg1-iNOS-**	US	IL4/IL13	LPS/IFNg	LPS/GMCSF	LPS/IFNg/GMCSF
US		<0.0001	<0.0001	<0.0001	<0.0001	US		<0.0001	<0.0001	<0.0001	<0.0001
IL4/IL13			<0.0001	<0.0001	<0.0001	IL4/IL13			<0.0001	ns	<0.0001
LPS/IFNg				<0.0001	<0.0001	LPS/IFNg				<0.0001	0.001
LPS/GMCSF					<0.0001	LPS/GMCSF					<0.0001
LPS/IFNg/GMCSF						LPS/IFNg/GMCSF					
**Arg1-iNOS+**	US	IL4/IL13	LPS/IFNg	LPS/GMCSF	LPS/IFNg/GMCSF	**Arg1-iNOS+**	US	IL4/IL13	LPS/IFNg	LPS/GMCSF	LPS/IFNg/GMCSF
US		0.0002	<0.0001	<0.0001	<0.0001	US		ns	<0.0001	<0.0001	<0.0001
IL4/IL13			<0.0001	<0.0001	<0.0001	IL4/IL13			<0.0001	<0.0001	<0.0001
LPS/IFNg				<0.0001	<0.0001	LPS/IFNg				<0.0001	<0.0001
LPS/GMCSF					<0.0001	LPS/GMCSF					<0.0001
LPS/IFNg/GMCSF						LPS/IFNg/GMCSF					
**Arg1+iNOS-**	US	IL4/IL13	LPS/IFNg	LPS/GMCSF	LPS/IFNg/GMCSF	**Arg1+iNOS-**	US	IL4/IL13	LPS/IFNg	LPS/GMCSF	LPS/IFNg/GMCSF
US		0.0002	ns	<0.0001	ns	US		0.0002	ns	<0.0001	ns

IL4/IL13			<0.0001	<0.0001	<0.0001	IL4/IL13			<0.0001	<0.0001	<0.0001
LPS/IFNg				<0.0001	ns	LPS/IFNg				<0.0001	ns
LPS/GMCSF					<0.0001	LPS/GMCSF					<0.0001
LPS/IFNg/GMCSF						LPS/IFNg/GMCSF					
**Arg1+iNOS+**	US	IL4/IL13	LPS/IFNg	LPS/GMCSF	LPS/IFNg/GMCSF	**Arg1+iNOS+**	US	IL4/IL13	LPS/IFNg	LPS/GMCSF	LPS/IFNg/GMCSF
US		0.0119	ns	<0.0001	ns	US		ns	<0.0001	<0.0001	<0.0001
IL4/IL13			ns	<0.0001	<0.0001	IL4/IL13			<0.0001	<0.0001	<0.0001
LPS/IFNg				<0.0001	<0.0001	LPS/IFNg				<0.0001	<0.0001
LPS/GMCSF					<0.0001	LPS/GMCSF					ns
LPS/IFNg/GMCSF						LPS/IFNg/GMCSF					
