# Supplementary material for: Monocyte infiltration induces CNS arginine catabolism to fuel neuroinflammation
Source: Nat Immunol. 2026 May 18;27(7):1418–32. doi: 10.1038/s41590-026-02516-4 (PMC13310764; doi:10.1038/s41590-026-02516-4)
Supplement: Supplementary file 2 — Reporting Summary [file 41590_2026_2516_MOESM2_ESM.pdf]

nature portfolio

Corresponding author(s): Sharif O. & Schabbauer G.

Last updated by author(s): Apr 8, 2026

Reporting Summary

Nature Portfolio wishes to improve the reproducibility of the work that we publish. This form provides structure for consistency and transparency in reporting. For further information on Nature Portfolio policies, see our Editorial Policies and the Editorial Policy Checklist.

Statistics

For all statistical analyses, confirm that the following items are present in the figure legend, table legend, main text, or Methods section.

n/a	Confirmed	
☐	☒	The exact sample size (n) for each experimental group/condition, given as a discrete number and unit of measurement
☐	☒	A statement on whether measurements were taken from distinct samples or whether the same sample was measured repeatedly
☐	☒	The statistical test(s) used AND whether they are one- or two-sided<br>*Only common tests should be described solely by name; describe more complex techniques in the Methods section.*
☐	☒	A description of all covariates tested
☐	☒	A description of any assumptions or corrections, such as tests of normality and adjustment for multiple comparisons
☐	☒	A full description of the statistical parameters including central tendency (e.g. means) or other basic estimates (e.g. regression coefficient) AND variation (e.g. standard deviation) or associated estimates of uncertainty (e.g. confidence intervals)
☐	☒	For null hypothesis testing, the test statistic (e.g. F, t, r) with confidence intervals, effect sizes, degrees of freedom and P value noted<br>*Give P values as exact values whenever suitable.*
☒	☐	For Bayesian analysis, information on the choice of priors and Markov chain Monte Carlo settings
☒	☐	For hierarchical and complex designs, identification of the appropriate level for tests and full reporting of outcomes
☐	☒	Estimates of effect sizes (e.g. Cohen's d, Pearson's r), indicating how they were calculated

Our web collection on statistics for biologists contains articles on many of the points above.

Software and code

Policy information about availability of computer code

Data collection	OlyVIA Ver.2.9<br>Step-OnePlus Real-Time PCR Software v2.3<br>iBright Analysis Software<br>Gen5<br>CytExpert v2.5<br>BD FACSDiva Software v9.0<br>HiSeq Control Software, HCS, HD 3.4.0.38<br>Real-Time Analysis Software, RTA, 2.7.7<br>Skyline (Version 25.1.0.237).
Data analysis	Microsoft Excel<br>TraceFinder 4.1/5.1 software<br>CytExpert v2.5<br>Prism 9.4.1<br>Adobe Illustrator CS6<br>FlowJo software v10.8.1<br>ImageJ v1.53<br>ImageJ2 v2.16.0<br>GIMP<br>QuPath v0.5.1<br>Picard tools (2.19.2)

Bioconductor (3.11)
GenomicAlignments (1.24.0) package
R v4.2.1/v4.4.2
limma R package v3.52.4/v3.62.2
fgsea R package
Seurat R package v4.3.0
Mfuzz R package v2.56.0
Metaboanalyst v5.0
SCiLS Lab 2023a Pro

For manuscripts utilizing custom algorithms or software that are central to the research but not yet described in published literature, software must be made available to editors and reviewers. We strongly encourage code deposition in a community repository (e.g. GitHub). See the Nature Portfolio guidelines for submitting code & software for further information.

Data

Policy information about availability of data

All manuscripts must include a data availability statement. This statement should provide the following information, where applicable:

- Accession codes, unique identifiers, or web links for publicly available datasets
- A description of any restrictions on data availability
- For clinical datasets or third party data, please ensure that the statement adheres to our policy

Data availability: Bulk RNA-Seq data generated in this study has been deposited in Gene Expression Omnibus (GEO) under accession number GSE231474. The results published here are in part based on analysis of publicly available scRNA-seq data deposited under Gene Expression Ominbus data set GSE130119. Source data are provided with this paper.
Code Availability: All code used in this study is available on GitHub (https://github.com/osharif-lab/EAE-Arg1).

Research involving human participants, their data, or biological material

Policy information about studies with human participants or human data. See also policy information about sex, gender (identity/presentation), and sexual orientation and race, ethnicity and racism.

Reporting on sex and gender	Tissue samples used in this study were acquired from the tissue bank of the Division for Neuropathology and Neurochemistry at the Medical University of Vienna and included five male and 13 female patients.
Reporting on race, ethnicity, or other socially relevant groupings	N/A
Population characteristics	Multiple sclerosis (MS) was diagnosed either throughout the patient's life or via autopsy and neuropathological examination. Mean age was 52±15 years. in five male and 13 female patients. The clinical course of the disease was classified as acute MS (AMS) (three patients), relapsing and remitting MS (RRMS) (five patients), secondary progressive MS (SPMS) (four patients), and primary progressive MS (PPMS) (two patients). In some cases, the definite clinical course was not available; these cases were designated as unknown (four patients). Control cases showed absence of major pathological alterations, however, minor vascular pathology in the form of small vessel disease was frequently detected. Only white matter sections without significant pathology were selected. Mean age was 63±15 years in four male and three female controls. Patient characteristics are indicated in Supplementary Table 7.
Recruitment	All procedures involving human material in this study were post-mortem
Ethics oversight	Ethics approved by the Ethics Committee of Medical University of Vienna (votes: 1636/19 and 1067/2024).

Note that full information on the approval of the study protocol must also be provided in the manuscript.

Field-specific reporting

Please select the one below that is the best fit for your research. If you are not sure, read the appropriate sections before making your selection.

☒ Life sciences ☐ Behavioural & social sciences ☐ Ecological, evolutionary & environmental sciences

For a reference copy of the document with all sections, see nature.com/documents/nr-reporting-summary-flat.pdf

Life sciences study design

All studies must disclose on these points even when the disclosure is negative.

Sample size	No statistical methods were used to predetermine sample size, but our sample sizes were similar to those generally used in EAE studies.
Data exclusions	Animals exhibiting extreme weight loss due to tamoxifen diet (>20% after 2 weeks) or those that did not develop disease (score 0) were excluded from the study.

Replication	All experiments were performed at least two times and represent reproducible findings, except OMICS data and if stated otherwise.
Randomization	Mice were grouped randomly per cage.
Blinding	Data collection and analysis were not performed blind to the conditions of the experiments except targeted metabolomics data collection was blinded as collaboration partners performing data aquisition were not aware of experimental groups. Blinding was not possible for experiments involving arginine free diet due to the color of the food.

Behavioural & social sciences study design

All studies must disclose on these points even when the disclosure is negative.

Study description	*Briefly describe the study type including whether data are quantitative, qualitative, or mixed-methods (e.g. qualitative cross-sectional, quantitative experimental, mixed-methods case study).*
Research sample	*State the research sample (e.g. Harvard university undergraduates, villagers in rural India) and provide relevant demographic information (e.g. age, sex) and indicate whether the sample is representative. Provide a rationale for the study sample chosen. For studies involving existing datasets, please describe the dataset and source.*
Sampling strategy	*Describe the sampling procedure (e.g. random, snowball, stratified, convenience). Describe the statistical methods that were used to predetermine sample size OR if no sample-size calculation was performed, describe how sample sizes were chosen and provide a rationale for why these sample sizes are sufficient. For qualitative data, please indicate whether data saturation was considered, and what criteria were used to decide that no further sampling was needed.*
Data collection	*Provide details about the data collection procedure, including the instruments or devices used to record the data (e.g. pen and paper, computer, eye tracker, video or audio equipment) whether anyone was present besides the participant(s) and the researcher, and whether the researcher was blind to experimental condition and/or the study hypothesis during data collection.*
Timing	*Indicate the start and stop dates of data collection. If there is a gap between collection periods, state the dates for each sample cohort.*
Data exclusions	*If no data were excluded from the analyses, state so OR if data were excluded, provide the exact number of exclusions and the rationale behind them, indicating whether exclusion criteria were pre-established.*
Non-participation	*State how many participants dropped out/declined participation and the reason(s) given OR provide response rate OR state that no participants dropped out/declined participation.*
Randomization	*If participants were not allocated into experimental groups, state so OR describe how participants were allocated to groups, and if allocation was not random, describe how covariates were controlled.*

Ecological, evolutionary & environmental sciences study design

All studies must disclose on these points even when the disclosure is negative.

Study description	*Briefly describe the study. For quantitative data include treatment factors and interactions, design structure (e.g. factorial, nested, hierarchical), nature and number of experimental units and replicates.*
Research sample	*Describe the research sample (e.g. a group of tagged Passer domesticus, all Stenocereus thurberi within Organ Pipe Cactus National Monument), and provide a rationale for the sample choice. When relevant, describe the organism taxa, source, sex, age range and any manipulations. State what population the sample is meant to represent when applicable. For studies involving existing datasets, describe the data and its source.*
Sampling strategy	*Note the sampling procedure. Describe the statistical methods that were used to predetermine sample size OR if no sample-size calculation was performed, describe how sample sizes were chosen and provide a rationale for why these sample sizes are sufficient.*
Data collection	*Describe the data collection procedure, including who recorded the data and how.*
Timing and spatial scale	*Indicate the start and stop dates of data collection, noting the frequency and periodicity of sampling and providing a rationale for these choices. If there is a gap between collection periods, state the dates for each sample cohort. Specify the spatial scale from which the data are taken*
Data exclusions	*If no data were excluded from the analyses, state so OR if data were excluded, describe the exclusions and the rationale behind them, indicating whether exclusion criteria were pre-established.*
Reproducibility	*Describe the measures taken to verify the reproducibility of experimental findings. For each experiment, note whether any attempts to repeat the experiment failed OR state that all attempts to repeat the experiment were successful.*
Randomization	*Describe how samples/organisms/participants were allocated into groups. If allocation was not random, describe how covariates were controlled. If this is not relevant to your study, explain why.*

Blinding

Describe the extent of blinding used during data acquisition and analysis. If blinding was not possible, describe why OR explain why blinding was not relevant to your study.

Did the study involve field work? ☐ Yes ☐ No

Field work, collection and transport

Field conditions

Describe the study conditions for field work, providing relevant parameters (e.g. temperature, rainfall).

Location

State the location of the sampling or experiment, providing relevant parameters (e.g. latitude and longitude, elevation, water depth).

Access & import/export

Describe the efforts you have made to access habitats and to collect and import/export your samples in a responsible manner and in compliance with local, national and international laws, noting any permits that were obtained (give the name of the issuing authority, the date of issue, and any identifying information).

Disturbance

Describe any disturbance caused by the study and how it was minimized.

Reporting for specific materials, systems and methods

We require information from authors about some types of materials, experimental systems and methods used in many studies. Here, indicate whether each material, system or method listed is relevant to your study. If you are not sure if a list item applies to your research, read the appropriate section before selecting a response.

Materials & experimental systems

n/a	Involved in the study	
☐	☒	Antibodies
☒	☐	Eukaryotic cell lines
☒	☐	Palaeontology and archaeology
☐	☒	Animals and other organisms
☒	☐	Clinical data
☒	☐	Dual use research of concern
☒	☐	Plants

Methods

n/a	Involved in the study	
☒	☐	ChIP-seq
☐	☒	Flow cytometry
☒	☐	MRI-based neuroimaging

Antibodies

Antibodies used

For flow cytometry of viable spinal cord cell populations the following antibodies were used: CD45.2-BV650 (clone 104, Biolegend, #109836, 1:100), Ly6G-BV605 (clone 1A8, Biolegend, #127639, 1:100), Ly6G-AF-700 (clone 1A8, Biolegend, #127622, 1:100), CD3-BV605 (clone 145-2C11, Biolegend, #100351, 1:100), Ly6C-BV605 (clone HK14, Biolegend, #128035, 1:100), Ly6C-BV510 (clone HK14, Biolegend, #128033, 1:100), CD11b-BV510 (clone M1/70, Biolegend, #101245, 1:100), CD11b-PB (clone M1/70.15, Invitrogen, # RM2828, 1:100), CD11b-BV605 (clone M1/70, Biolegend, #101237, 1:100), CX3CR1-PeCy7 (clone SA011F11, Biolegend, #149015, 1:100), CCR2-BV421 (clone SA203G11, Biolegend, #150605, 1:100), CCR2-FITC (clone SA203G11, Biolegend, #150607, 1:100), F4/80-BV510 (clone BM8, Biolegend, #123135, 1:100), F4/80-FITC (clone BM8, Biolegend, #123108, 1:100), F4/80-BV605 (clone BM8, Biolegend, #123133, 1:100), CD11c-PE-Cy5.5 (clone N418, Invitrogen, #35-0114-82, 1:100), P2RY12-APC (clone S16007D, Biolegend, #848006, 1:100), P2RY12-AF488 (clone S16007D, Biolegend, #848016, 1:100), MerTK-APC (clone 2B10C42, Biolegend, #151507, 1:100), CD44-FITC (clone IM7, Invitrogen, #11-0441-82, 1:100), CD44-eF506 (clone IM7, Invitrogen, # 69-0441-82, 1:100), CD64-BV605 (clone X54-5/7.1, Biolegend, #139323, 1:100), CD3-AF700 (clone 17A2, Biolegend, #100216, 1:100), CD3-rF710 (clone 17A2, Tonbo, #80-0032-U100, 1:80), CD8-BV510 (clone 53-6.7, Biolegend, #100751, 1:80), CD4-APC (clone REA604, Miltenyi Biotec, #130-116-487, 1:80), CD4-PE (clone REA604, Miltenyi Biotec, #130-116-509, 1:80) and CD25-BV421 (clone PC61, Biolegend, #102033, 1:80), Arginase 1-APC (clone A1exF5, Invitrogen, #17-3697-82, 1:100), Arginase 1-PE (clone A1exF5, Invitrogen, #12-3697-80, 1:50), iNOS-PE-Cy5.5 (clone CXNFT, Invitrogen, #12-5920-80, 1:50), iNOS-PE (clone CXNFT, Invitrogen, #12-5920-82, 1:100), IL-10-APC (clone JES5-16E3, Invitrogen, # 17-7101-82, 1:50), TGF-β1-PE (clone TW7-16B4, Biolegend, # 141403, 1:50), Heme oxygenase-1-AF647 (clone EPR18161-128, Abcam, #ab237268, 1:50), IL-17-PE (clone REA660, Miltenyi Biotec, #130-112-009, 1:50), IFNɣ-FITC (clone REA638, Miltenyi Biotec, #130-117-780, 1:50) and FoxP3-APC (clone 3G3, Tonbo, #20-5773, 1:100) or isotype control rat IgG2a kappa-APC (clone eBR2a, Invitrogen, #17-4321-81, 1:100), rat IgG2a kappa-PE (clone eBR2a, Invitrogen, #12-4321-80, 1:50), rat IgG2a kappa-PE-Cy5.5 (clone eBR2a, Invitrogen, #35-4321-82, 1:50), rat IgG2a kappa-PE (clone eBR2a, Invitrogen, #12-4321-41, 1:100), rat IgG2b kappa-APC (clone eB149/10H5, Invitrogen, #17-4031-81, 1:50), rabbit anti-goat IgG-AF647 (Invitrogen, #A21446, 1:50), mouse IgG1 kappa-PE (clone P3.6.2.8.1, Invitrogen, #12-4714-81, 1:50), REA control antibody-APC (clone REA293, Miltenyi Biotec, #130-113-446, 1:80), REA control antibody-FITC (clone REA293, Miltenyi Biotec, #130-113-449, 1:50), REA control antibody-PE (clone REA293, Miltenyi Biotec, #130-118-347, 1:50) and rat IgG1 kappa-APC (clone P3.6.2.8.1, eBioscience, #17-4714-41, 1:100).
For analysis of tomato expression within Ly6Chigh blood monocytes by flow cytometry the following antibodies were used: Ly6G-AF-700 (clone 1A8, Biolegend, #127622, 1:200), Ly6C-BV605 (clone HK14, Biolegend, #128035, 1:80), CD11b-PE-Cy7 (clone M1/70, eBioscience, #25-0112-82, 1:400), B220-PE-Cy5 (clone RA3-6B2, Tonbo, #55-0452-U100, 1:100) and CD3-PerCP-Cy5.5 (clone 145-2C11, Tonbo, #65-0031-U025, 1:100).

For analysis of BMDMs by flow cytometry, the following antibodies were used: F4/80-BV605 (clone BM8, Biolegend, #123133, 1:80), F4/80-BV421 (clone BM8, Biolegend, #123137, 1:80) and CD11b-BV510 (clone M1/70, Biolegend, #101245, 1:80), Arginase 1-APC (clone A1exF5, Invitrogen, #17-3697-82, 1:100) and iNOS-PE (clone CXNFT, Invitrogen, #12-5920-82, 1:100) or isotype control rat IgG2a kappa-APC (clone eBR2a, Invitrogen, #17-4321-81, 1:100), rat IgG2a kappa-PE (clone eBR2a, Invitrogen, #12-4321-41, 1:100). For analysis of in vitro induced Tregs, the following antibodies were used: CD4-PerCP-Cy5.5 (clone GK1.5, Biolegend, #100433, 1:100) and CD25-BV421 (clone PC61, Biolegend, #102033, 1:100), FoxP3-APC (clone 3G3, Tonbo, #20-5773, 1:100) or isotype control mouse IgG1 kappa-APC (clone P3.6.2.8.1, Invitrogen, #17-4714-82, 1:100).

For western blotting the following antibodies were used: Arg1 (clone D4E3M, Cell Signaling, #93668S, 1:1000), GRB2 (Cell Signaling, #3972, 1:1000), HRP-conjugated anti-rabbit secondary antibody (Cell Signaling, #7074, 1:5000).

For Immunofluorescence staining the following antibodies were used: anti-Arginase 1 primary antibody (polyclonal, Invitrogen, #PA5-85267, 1:100), anti-NOS2 (1:5000, rabbit, PA3-030A, Invitrogen), anti-IBA1 (1:300, rabbit, 019-19741, FUJIFILM Wako), anti-GFAP (1:1000, rabbit, Z0334, Agilent Dako), anti-neurofilament H (clone: SMI32, 1:400, mouse, 801701, Sternberger Monoclonals), Cy3 anti-rabbit (1:1000, goat, 711-485-152, Jackson ImmunoResearch); AF488 anti-mouse (1:800, goat, 115-545-166, Jackson ImmunoResearch), biotinylated secondary antibody (K675, Dako, #SDS391), anti-CD68 antibody (clone KP1, Dako, #M0814, 1:500), Alexa Fluor 488-linked anti-mouse IgG secondary antibody (polyclonal, Jackson ImmunoResearch, #115-545-166, 1:800), CD45 (clone 30-F11, Invitrogen, #14-0451-82, 1:100) and Alexa Fluor 647-goat anti-rat IgG secondary antibody (Invitrogen, #A21247, 1:1000), anti-GFP (Abcam, #ab13970, 1:500) and Alexa Fluor 488-goat anti-chicken IgG secondary antibody (Invitrogen, #A11039, 1:400).

Validation

All antibodies are commercially available and their validation statements are available on the manufacturer's website.

Eukaryotic cell lines

Policy information about cell lines and Sex and Gender in Research

Cell line source(s)

State the source of each cell line used and the sex of all primary cell lines and cells derived from human participants or vertebrate models.

Authentication

Describe the authentication procedures for each cell line used OR declare that none of the cell lines used were authenticated.

Mycoplasma contamination

Confirm that all cell lines tested negative for mycoplasma contamination OR describe the results of the testing for mycoplasma contamination OR declare that the cell lines were not tested for mycoplasma contamination.

Commonly misidentified lines
(See ICLAC register)

Name any commonly misidentified cell lines used in the study and provide a rationale for their use.

Palaeontology and Archaeology

Specimen provenance

Provide provenance information for specimens and describe permits that were obtained for the work (including the name of the issuing authority, the date of issue, and any identifying information). Permits should encompass collection and, where applicable, export.

Specimen deposition

Indicate where the specimens have been deposited to permit free access by other researchers.

Dating methods

If new dates are provided, describe how they were obtained (e.g. collection, storage, sample pretreatment and measurement), where they were obtained (i.e. lab name), the calibration program and the protocol for quality assurance OR state that no new dates are provided.

☐ Tick this box to confirm that the raw and calibrated dates are available in the paper or in Supplementary Information.

Ethics oversight

Identify the organization(s) that approved or provided guidance on the study protocol, OR state that no ethical approval or guidance was required and explain why not.

Note that full information on the approval of the study protocol must also be provided in the manuscript.

Animals and other research organisms

Policy information about studies involving animals; ARRIVE guidelines recommended for reporting animal research, and Sex and Gender in Research

Laboratory animals

In all experiments 8-12 weeks old animals were used. C57BL/6J mice were obtained from the Animal Core Facility of the Medical University of Vienna or purchased from Janvier (#SC-C57J-M). Csf2rbfl/fl Ccr2-creERT2(-mKate2) mice were kindly provided by Burkard Becher and further crossed to mice expressing a CAG-loxP-STOP-loxP-tdTomato cassette in the Rosa26 locus (B6;129S6-Gt(ROSA)26Sortm14(CAG-tdTomato)Hze/J, Jackson Lab stock #007908, R26tdTomato), which have been provided by Christoph Österreicher, to generate R26tdTomato Ccr2-creERT2 fate-mapping animals. R26tdTomato Ms4a3-cre animals have been generated by crossing R26tdTomato animals to Ms4a3-cre expressing animals (C57BL/6J-Ms4a3em2(cre)Fgnx/J, Jackson Lab stock #036382), which were purchased from Jackson Laboratories. Arg1-eYFP reporter mice (B6.129S4-Arg1tm1.1Lky/J, Jackson Lab stock #0015857) were purchased from Jackson Laboratories and crossed with R26tdTomato Ccr2-creERT2 animals to generate R26tdTomato Ccr2-creERT2 Arg1-eYFP animals. Arg1fl/fl animals (C57BL/6-Arg1tm1Pmu/J, Jackson Lab stock #008817) were a kind gift of Peter J.

	Murray and crossed to Cx3cr1-cre mice (B6J.B6N(Cg)-Cx3cr1tm1.1(cre)Jung/J, Jackson Lab stock #025524), obtained from Jackson Laboratories, or Ccr2-creERT2 mice to generate Arg1fl/fl Cx3cr1-cre or Arg1fl/fl Ccr2-creERT2 animals, respectively.
Wild animals	N/A
Reporting on sex	In the vast majority of experiments, only male mice were used as female sex hormones are protective in EAE and thereby distinct female estrous phases influence the development of neuro-inflammation and clinical score inter-mouse variance.
Field-collected samples	N/A
Ethics oversight	All animal experiments were performed in strict accordance with regulations of the relevant animal welfare acts and protocols approved by the respective regulatory bodies (Austrian Ministry of Sciences, project numbers 2022-0.474.463 and 2025-1.040.322).

Note that full information on the approval of the study protocol must also be provided in the manuscript.

Clinical data

Policy information about clinical studies

All manuscripts should comply with the ICMJE guidelines for publication of clinical research and a completed CONSORT checklist must be included with all submissions.

Clinical trial registration	*Provide the trial registration number from ClinicalTrials.gov or an equivalent agency.*
Study protocol	*Note where the full trial protocol can be accessed OR if not available, explain why.*
Data collection	*Describe the settings and locales of data collection, noting the time periods of recruitment and data collection.*
Outcomes	*Describe how you pre-defined primary and secondary outcome measures and how you assessed these measures.*

Dual use research of concern

Policy information about dual use research of concern

Hazards

Could the accidental, deliberate or reckless misuse of agents or technologies generated in the work, or the application of information presented in the manuscript, pose a threat to:

No	Yes	
☐	☐	Public health
☐	☐	National security
☐	☐	Crops and/or livestock
☐	☐	Ecosystems
☐	☐	Any other significant area

Experiments of concern

Does the work involve any of these experiments of concern:

No	Yes	
☐	☐	Demonstrate how to render a vaccine ineffective
☐	☐	Confer resistance to therapeutically useful antibiotics or antiviral agents
☐	☐	Enhance the virulence of a pathogen or render a nonpathogen virulent
☐	☐	Increase transmissibility of a pathogen
☐	☐	Alter the host range of a pathogen
☐	☐	Enable evasion of diagnostic/detection modalities
☐	☐	Enable the weaponization of a biological agent or toxin
☐	☐	Any other potentially harmful combination of experiments and agents

Plants

Seed stocks: N/A

Novel plant genotypes: N/A

Authentication: N/A

ChIP-seq

Data deposition

☐ Confirm that both raw and final processed data have been deposited in a public database such as GEO.

☐ Confirm that you have deposited or provided access to graph files (e.g. BED files) for the called peaks.

Data access links
May remain private before publication.: *For "Initial submission" or "Revised version" documents, provide reviewer access links. For your "Final submission" document, provide a link to the deposited data.*

Files in database submission: *Provide a list of all files available in the database submission.*

Genome browser session
(e.g. UCSC): *Provide a link to an anonymized genome browser session for "Initial submission" and "Revised version" documents only, to enable peer review. Write "no longer applicable" for "Final submission" documents.*

Methodology

Replicates: *Describe the experimental replicates, specifying number, type and replicate agreement.*

Sequencing depth: *Describe the sequencing depth for each experiment, providing the total number of reads, uniquely mapped reads, length of reads and whether they were paired- or single-end.*

Antibodies: *Describe the antibodies used for the ChIP-seq experiments; as applicable, provide supplier name, catalog number, clone name, and lot number.*

Peak calling parameters: *Specify the command line program and parameters used for read mapping and peak calling, including the ChIP, control and index files used.*

Data quality: *Describe the methods used to ensure data quality in full detail, including how many peaks are at FDR 5% and above 5-fold enrichment.*

Software: *Describe the software used to collect and analyze the ChIP-seq data. For custom code that has been deposited into a community repository, provide accession details.*

Flow Cytometry

Plots

Confirm that:

☒ The axis labels state the marker and fluorochrome used (e.g. CD4-FITC).

☒ The axis scales are clearly visible. Include numbers along axes only for bottom left plot of group (a 'group' is an analysis of identical markers).

☒ All plots are contour plots with outliers or pseudocolor plots.

☒ A numerical value for number of cells or percentage (with statistics) is provided.

Methodology

Sample preparation: For spinal cord cell sample preparation: mice were euthanized, spinal cords extracted, cut into small pieces and digested using either a tissue dissociation kit (Miltenyi Biotec, tissue dissociation kit 1 for inflamed neural tissue, #130-110-201) according to the manufacturer's instructions or 3 mL digestion medium (DMEM; 1 mg/ml Papain, Sigma-Aldrich, #P4762; 0.03 mg/ml DNase I, Roche, #11284932001; 0.5 mg/ml Collagenase/Dispase, Sigma-Aldrich, #10269638001). In experiments where digestion medium was used, mechanical and enzymatic dissociation was performed by GentleMACS™ Octo Dissociator with Heaters (Miltenyi Biotec) with a spinal cord specific program (37C_ABDK_01). Digestion was stopped by addition of 5 mL of neutralization medium (DMEM, 10% FBS). Tissue homogenate was filtered through a pre-wet 70µm cell strainer and post

washing, centrifuged at 250 g for 5 min at RT. Supernatant was discarded, and isolated cells were separated from debris/myelin using a 70/37/30% layered Percoll gradient (Sigma-Aldrich, #P1644) with centrifugation at 300 g for 40 min at 18°C without brake. 3 mL of the immune cell containing 70/37% interphase was collected, washed with 9 ml 1x HBSS (Gibco, #14175-05) and pelleted by centrifugation at 500 g for 7 min at 4°C. Debris removal in experiments involving the Miltenyi Biotec tissue dissociation kit was performed with Debris Removal Solution (Miltenyi Biotec, #130-109-398). Post cellular isolation with either protocols, cellular pellets were resuspended in 1 mL Flow buffer (PBS, 1% FCS). Cells were counted and again pelleted by centrifugation at 500 g for 7 min at 4 °C. Finally, cells were resuspended with the appropriate volume of flow buffer and further prepared for flow cytometry or FACS sorting.

For preparation of bone marrow cell suspensions, cells were flushed from murine femur and tibia with sterile PBS (Gibco, #14190-094). Cell suspensions were filtered through 70 µm cell strainers (Falcon, #352350) and two times the volume erythrocyte lysis buffer (0.15M NH4Cl, 10mM KHCO3, 0.1mM Na2EDTA, pH: 7.2 – 7.4) was added for 5 min. Cells were pelleted and resuspended in RPMI-1640 (Gibco, #61870-044) supplemented with 10% FCS (Sigma-Aldrich, #F7524-500ml), 100 U/mL penicillin/streptomycin (Lonza, # LON17-745E and Capricorn, # AAS-B) and 30 ng/mL macrophage colony stimulating factor (M-CSF, R&D Systems, #416-ML-050). Cells were then transferred to 10 cm culture dishes (Sarstedt, 82.1473.001) at a concentration of 0.5 million/mL. After 3 days of cultivation, fresh RPMI-1640 supplemented as described above was added (1/10 of total volume). Another additional 3 days later, cells were washed once with pre-warmed PBS and detached using pre-warmed CellStripper (Corning, #15313661). Cells were re-plated at a concentration of 1 million/mL in 12 or 24 well plates (Costar, #3737) and stimulated with: 100 ng/mL LPS (Invivogen, #tlrl-3pelps), 20 ng/mL IFN-γ (Miltenyi Biotec, #130-105-774), 20 ng/mL GM-CSF (R&D Systems, #415-ML-050), 20 ng/mL IL-4 (Miltenyi Biotec, #130-097-761), 20 ng/mL IL-13 (Miltenyi Biotec, #130-094-070).

Stainings for viablity was performed using fixable viability dye (Invitrogen, #65-0865-14, 1:2000, 10 min at RT) and surface staining was performed for 20 min at room temperature. Intracellular staining was performed using the Fixation and Permeabilization Buffer Set (eBioscience, #88-8824-00), True-Phos Buffer Set (Biolegend,# 425401) or Foxp3/Transcription Factor Staining Buffer Set (Invitrogen, #00-5523-00) according to the manufacturer's instructions.

Instrument: CytoFLEX S Flow Cytometer (Beckman Coulter)

Software: CytExpert (Version 2.5) and FlowJo (Version 10.8.1, LLC) software

Cell population abundance: A minimum of 100,000 viable CD3-Ly6G−CD45+CX3CR1+Arg1+/− cells were sorted and used for bulk RNA seq and validated for the presence or absence of Arg1 expression

Gating strategy: Cells were gated on FSC-A/SSC-A. A detailed gating strategy for myeloid cells and T cells is provided in Extended Data Fig. 1c and 1e respectively. A detailed gating strategy for Arg1 and iNOS within spinal cord Mdcs or tomato expression within monocytes during EAE is provided in Extended Data Fig. 3c and 3f respectively. A gating strategy defining different Mdc/microglia populations using fate-mapper animals is provided in Extended Data Fig. 4a and f. A gating strategy for T cell subsets (Th1, Th17, Treg) is provided in Extended Data Fig. 10h. Positive cell populations were determined using FMO or isotype controls.

☒ Tick this box to confirm that a figure exemplifying the gating strategy is provided in the Supplementary Information.

Magnetic resonance imaging

Experimental design

Design type: *Indicate task or resting state; event-related or block design.*

Design specifications: *Specify the number of blocks, trials or experimental units per session and/or subject, and specify the length of each trial or block (if trials are blocked) and interval between trials.*

Behavioral performance measures: *State number and/or type of variables recorded (e.g. correct button press, response time) and what statistics were used to establish that the subjects were performing the task as expected (e.g. mean, range, and/or standard deviation across subjects).*

Acquisition

Imaging type(s): *Specify: functional, structural, diffusion, perfusion.*

Field strength: *Specify in Tesla*

Sequence & imaging parameters: *Specify the pulse sequence type (gradient echo, spin echo, etc.), imaging type (EPI, spiral, etc.), field of view, matrix size, slice thickness, orientation and TE/TR/flip angle.*

Area of acquisition: *State whether a whole brain scan was used OR define the area of acquisition, describing how the region was determined.*

Diffusion MRI ☐ Used ☐ Not used

Preprocessing

Preprocessing software	*Provide detail on software version and revision number and on specific parameters (model/functions, brain extraction, segmentation, smoothing kernel size, etc.).*
Normalization	*If data were normalized/standardized, describe the approach(es): specify linear or non-linear and define image types used for transformation OR indicate that data were not normalized and explain rationale for lack of normalization.*
Normalization template	*Describe the template used for normalization/transformation, specifying subject space or group standardized space (e.g. original Talairach, MNI305, ICBM152) OR indicate that the data were not normalized.*
Noise and artifact removal	*Describe your procedure(s) for artifact and structured noise removal, specifying motion parameters, tissue signals and physiological signals (heart rate, respiration).*
Volume censoring	*Define your software and/or method and criteria for volume censoring, and state the extent of such censoring.*

Statistical modeling & inference

Model type and settings	*Specify type (mass univariate, multivariate, RSA, predictive, etc.) and describe essential details of the model at the first and second levels (e.g. fixed, random or mixed effects; drift or auto-correlation).*
Effect(s) tested	*Define precise effect in terms of the task or stimulus conditions instead of psychological concepts and indicate whether ANOVA or factorial designs were used.*

Specify type of analysis: ☐ Whole brain ☐ ROI-based ☐ Both

Statistic type for inference (See Eklund et al. 2016)	*Specify voxel-wise or cluster-wise and report all relevant parameters for cluster-wise methods.*
Correction	*Describe the type of correction and how it is obtained for multiple comparisons (e.g. FWE, FDR, permutation or Monte Carlo).*

Models & analysis

n/a	Involved in the study
☐	☐ Functional and/or effective connectivity
☐	☐ Graph analysis
☐	☐ Multivariate modeling or predictive analysis

Functional and/or effective connectivity	*Report the measures of dependence used and the model details (e.g. Pearson correlation, partial correlation, mutual information).*
Graph analysis	*Report the dependent variable and connectivity measure, specifying weighted graph or binarized graph, subject- or group-level, and the global and/or node summaries used (e.g. clustering coefficient, efficiency, etc.).*
Multivariate modeling and predictive analysis	*Specify independent variables, features extraction and dimension reduction, model, training and evaluation metrics.*
