# Supplementary material for: Monocyte infiltration induces CNS arginine catabolism to fuel neuroinflammation
Source: Nat Immunol. 2026 May 18;27(7):1418–32. doi: 10.1038/s41590-026-02516-4 (PMC13310764; doi:10.1038/s41590-026-02516-4)
Supplement: Supplementary file 4 — Supplementary Methods and Supplementary References. [file 41590_2026_2516_MOESM4_ESM.pdf]

**Monocyte infiltration induces CNS arginine catabolism to fuel neuroinflammation**

## Material and Methods (supplementary methods)

### EAE-conditioned medium

For EAE-conditioned media (EAE-CM) spinal cords were harvested from either peak-diseased or healthy C57BL/6J animals and cut into small pieces under sterile conditions. Cut spinal cord pieces were cultivated in 12 well plates with 2 mL RPMI-1640 (Gibco, 61870-044) supplemented with 10% FCS (Sigma-Aldrich, #F7524-500ml) and 100 U/mL penicillin/streptomycin (Lonza, #LON17-745E and Capricorn, #AAS-B) for 24 h at 37°C and 5% $CO_2$. The mixture was then filtered through a 40 μm cell strainer (Falcon, #352350) and centrifuged at 500 g for 5 min to remove both cells and debris. Subsequently, supernatant was collected, aliquoted and stored at -80 °C until further analyses. For *in vitro* EAE-CM stimulations of BMDMs, medium was changed to EAE-CM or healthy-CM diluted at 1:2.5 in RPMI-1640 for 24 h. In conditions where arginine was supplemented, L-arginine (Sigma Aldrich, #A8094-25g) was dissolved in RPMI-1640 to a concentration of 6 mM and pH-adjusted using hydrochloride acid.

### Luxol fast blue (LFB) staining and image processing of murine EAE tissue

Murine spinal cord tissues were fixed in 4% PFA at 4 °C overnight. Tissues were embedded in paraffin using a vacuum infiltration processor (Tissue-Tek VIP 6 AI, Sakura) and transferred to paraffin tissue blocks. Following wax solidification, 7 μm-thick tissue sections were cut using a microtome for the Luxol fast blue (LFB) with periodic acid-Schiff (PAS) staining and put on glass slides. Adherence of the slides was ensured by melting the paraffin at 70 °C for 30 min. To prepare the slides for staining, they were deparaffinized in xylene for 20 min. Subsequently, LFB-PAS staining was performed on non-rehydrated slides using 0.1% Luxol fast blue at 58 °C overnight. Consecutively, slides were washed in 96% ethanol and distilled water, and incubated in 0.1% aqueous litiumcarbonate solution for 5 min at 21 °C. Differentiation was

performed under inspection in 70% ethanol. After washing with distilled water, slides were incubated for 10 min in 0.8% periodic acid, for 6 min in total in a sulfide washing solution (0.35% hydrochloric acid and 0.38% potassium disulfate in distilled water), washed in distilled water and incubated for 20 min in Schiff's reagent (1.09033.0500, Sigma-Aldrich). Following 10 min of incubation in water, slides were counterstained for 1 min in Mayer's hemalum, washed, differentiated, blued, and coverslipped. Images of murine spinal cord samples were scanned using a NanoZoomer S60v2MD Slide scanner system (Hamamatsu). The ndpi-files were analyzed were in QuPath. Demyelinated area detection on LFB-PAS stainings was performed using the build-in pixel thresholder. All quantifications were normalized to the white matter area visible on the slides. Background normalization for the LFB images was performed by setting the white point threshold to 80% of the maximum intensity.

Immunofluorescence staining and image processing of murine EAE tissue

Mice were euthanized and transcardially perfused with PBS. Spinal cord tissue was isolated and fixed in ROTI Histofix (Roth, #P087.1) for 6 h. Tissue was saturated with sucrose by transferring to a 30% sucrose solution for 24-48 h and embedded in OCT cryoprotective (Scigen, #4586). The following stainings were performed on 8-12 μm thick tissue sections: FluoroMyelin (Invitrogen, #F34652 and #F34651, 1:300), Hoechst (Merck, #14533, stock: 10 mg/ml in NaCl, 1:3000), anti-CD45 (clone 30-F11, Invitrogen, #14-0451-82, 1:100) and Alexa Fluor 647-goat anti-rat IgG secondary antibody (Invitrogen, #A21247, 1:1000), anti-GFP (Abcam, #ab13970, 1:500) and Alexa Fluor 488-goat anti-chicken IgG secondary antibody (Invitrogen, #A11039, 1:400). Sections for anti-CD45 stainings were blocked in blocking buffer (10% goat serum, 0.3% Triton X-100 in PBS) for 1 h at 21 °C, followed by incubation with anti-CD45 antibody solution overnight. Tissues from *R26*$^{tdTomato}$ *Ccr2*-creER$^{T2}$ *Arg1*-eYFP animals were permeabilized with ice cold methanol for 10 min at -20 °C, blocked in blocking buffer (5% goat serum, 0.3% Triton X-100 in PBS) for 1 h at 21 °C and incubated with anti-GFP antibody solution overnight at 4 °C. After washing, sections were stained with respective secondary antibody solution for 2 h at 21 °C. FluoroMyelin staining was performed according to the

manufacturer's instructions. Slides were washed, counter-stained with Hoechst for 20 min at RT and mounted with either ProLong Gold antifade reagent (Invitrogen, #P10144 ) or ProLong Diamond antifade reagent (Invitrogen, #P36961). Images of spinal cord tissue sections were acquired using a slide scanner (Olympus/Evident, VS120 Virtual Slide System) or a confocal microscope (Zeiss, LSM 980). Images were cropped and processed (adjusting brightness and contrast) by using both Adobe Illustrator and ImageJ. To quantify co-expression of CCR2-tdTomato and Arg1-YFP in lesions, scans were analyzed in QuPath running the stardist model "dsb2018_heavy_augment.pb"[1] via a plug-in to identify all round-shaped structures in the tissue. To account for exposure and fluorescence inconsistencies between the slides, two random forest models were trained either on all green channel or all red channel cellular intensities via the built-in model tool, normalizing for mean and standard deviation discrepancies between the slides. Then a combination classifier was run on all slides. Only lesioned areas, identified by a white matter infiltrate of CCR2-tdTomato expressing cells were quantified.

Oil red O staining and quantification

Formalin-fixed BMDMs in 48-well tissue culture dishes were washed with 300 μl of 60% isopropanol and dried completely. 300 μl of oil red O (Sigma-Aldrich, #O-0625) working solution (composed of 4 parts water and 6 parts 0.6% oil red O dye in isopropanol) was added for 20 min, after which the wells were washed 6 times with dd$H_2O$. At the last wash, dd$H_2O$ was left in the wells so that microscopy could be performed. Oil red O staining of 40x images was analyzed using Fiji (ImageJ). For total cell counting, the green channel was used for cell segmentation following contrast enhancement, Gaussian smoothing ($\sigma = 2$), and thresholding (Triangle). Binary masks were processed using Fill Holes and Watershed, and cells were counted using Analyze Particles (≥2000 px, circularity 0.1–1.0), excluding edge particles. Oil red O staining was quantified from the red channel after contrast enhancement and thresholding to generate binary masks. To merge the fragmented oil red O signal within cells, binary images were processed using dilation and closing before particle analysis. Oil red O

positive objects were quantified using Analyze Particles, and all thresholds were kept constant within conditions.

Arginase and NOS Activity Assay

Mice were euthanized, spinal cords extracted, weighed, immediately snap frozen in liquid nitrogen and stored at -80 °C until further processing. Arginase and NOS activity of the obtained spinal cord tissue was analyzed using the Arginase activity (Abcam, #ab180877) and NOS activity assay (Abcam, #ab211084) according to the manufacturer's instructions and normalized to spinal cords weights used for the assay.

ELISAs and multiplex cytokine detection

Arginine levels in murine CSF and in cell supernatants were quantified using a commercially available ELISA kit (Immundiagnostik, #KR7733). Cytokine levels in cell supernatants were quantified using commercially available ELISA kits (IL-6: R&D Systems, #DY406-05; IL-1β: R&D Systems, #DY401-05; IL-10: R&D Systems, #DY417-05). Cytokine levels in murine CSF were measured using the bead-based immunoassay Legendplex (Biolegend, mouse inflammation panel, #740446). For MDA and 4-HNE quantification, mice were euthanized, spinal cords extracted, weighed, immediately snap frozen in liquid nitrogen and stored at -80 °C until further processing. MDA and 4-HNE of the obtained spinal cord tissue was analyzed using the MDA (Abcam, #ab118970) and 4-HNE ELISA kits (Abcam, #ab287803), normalized to spinal cords weights used for the assay. All kits were used according to the manufacturer's instructions.

RNA isolation and quantitative PCR

Total RNA from BMDMs and spinal cord cells was extracted using TRIzol Reagent (Ambion, #15596018) and a clean-up kit (Monarch® RNA Cleanup Kit, NewEngland Biolabs, #T2030L). Reverse transcription as well as qPCR reactions were performed using commercially available kits (Applied Biosystems, #4368814 and New England BioLabs, #M3003E) according to the

manufacturer's instructions. qPCR reactions were executed on a Step-OnePlus machine (Applied Biosystems) using Step-OnePlus Real-Time PCR Software (v2.3). Water controls were run simultaneously to ensure absence of contamination and primer dimers. qPCR was performed using the following primers which were syntheized from Microsynth: *Arginase 1*: *5'*-GGAAAGCCAATGAAGAGCTG-*3'* and *5'*-GCTTCCAACTGCCAGACTGT-*3'; Arginase 2: 5'*-CTGTGTCACCATGGGAGGAG-*3'* and *5'*-GCATGAGCATCAACCCAGAT-*3'*; *Nos2*: *5'*-TGAAGAAAACCCCTTGTGCT-*3'* and *5'*-TTCTGTGCTGTCCCAGTGAG-*3'*; *IL-6: 5'*-CAAGTCGGAGGCTTAATTACACATG-*3'* and *5'*-ATTGCCATTGCACAACTCTTTTCT-*3'*; *IL-1$\beta$*: *5'*-GGACAGAATATCAACCAACAAGTGATA-*3'* and *5'*-GTGTGCCGTCTTTCATTACACAG-*3'*; *IL-10*: *5'*-AGCTGAAGACCCTCAGGATG-*3'* and *5'*-TGGCCTTGTAGACACCTTGG-*3'*; *Slc7a2: 5'*-CCCGAGTATCGTGGTGTCTT-*3'* and *5'*-CTCTTGCGACACTGGACGTA *-3'; Csf2rb*: *5'*-CAGCCAGTGTCCTGTGAGC-*3'* and *5'*-CTGGGCAAGTGGCACC-*3'; Hmox1*: *5'*-CAGGTGATGCTGACAGAGGA-*3'* and *5'*-GAGAGTGAGGACCCACTGGA-*3'; Ne2l2: 5'*-AGCGACAGAAGGACTATGAGC-3' and *5'*-ATGTGGGCAACCTGGGAGTA-*3'; Nqo1*: *5'*-CGCCTGAGCCCAGATATTGT-3' and *5'*-GCACTCTCTCAAACCAGCCT-*3'; Txnrd1*: *5'*-AAAGACGATGAACGTGTCG-*3'* and *5'*-CTTAGTCAGCCCACACTTGAG-*3'; Gss: 5'*-GCACTGGGTCGTACCGAAG-*3'* and *5'*-GTAGCCATCCCAACTCGCT-*3'; Gsr*: *5'*-TGGAGCTCCAAGTGGTGACT-*3'* and *5'*-CCACAGCATAGACGCCTTTG-*3'; Hprt*: *5'*-CGCAGTCCCAGCGTCGTG-*3'* and *5'*-CCATCTCCTTCATGACATCTCGAG-*3'*. Sample ΔCt values were normalized to hypoxanthine phosphoribosyltransferase 1 (*Hprt*) and ΔΔCt values were calculated (ΔΔCt = ΔCt experimental group − ΔCt control).

Western Blot

For protein detection by western blot spinal cords were harvested and subsequently homogenized with metal beads in 1x RIPA buffer (Cell Signaling, #9806) containing a protease inhibitor cocktail (Roche, #04693132001) by using a Precellys24 device. Homogenates were further sonicated in an ultrasound bath for 15 min and centrifuged at 14 000 g for 10 min at 4

°C. Protein content in supernatants were quantified by using the PierceTM BCA Protein Assay kit (Thermo Fisher Scientific, #23225) according to manufacturer's instructions. Samples were mixed with 5x Laemmli buffer and separated by SDS-PAGE on a 4-20% polyacrylamide gel (Biorad, #4561094) and blotted onto a Roti®-PVDF membrane (ROTH, #T830.1). Proteins were probed with the following antibodies ARG1 (clone D4E3M, Cell Signaling, #93668S, 1:1000) and GRB2 (Cell Signaling, #3972, 1:1000) followed by HRP-conjugated anti-rabbit secondary antibody (Cell Signaling, #7074, 1:5000). Chemiluminescent detection was performed by using the WesternBright Sirius HRP substrate (Advansta, #K-12043-D20) and images were acquired with the Fusion Fx Vilber Lourmat imaging system.

Quantification of macrophages and microglia in human tissue samples

ARG1 positive myeloid cells were counted. For the quantification, a Nikon Eclipse E400 microscope was used. Regions of interest (ROI) were evaluated using a rectangle measuring 0.4x0.7 mm (0.28 $mm^2$) at 200x magnification. If possible, two ROIs for each lesion zone were counted and a mean was calculated. Only one lesion was not large enough to count two separate ROIs. The quantification was normalized to 1 $mm^2$. Lesion center inactive (LCI) refers to the center of inactive and chronic active lesions; lesion center active (LCA) refers to the lesion center of active lesions; lesion rim (LR) refers to the lesion rim of chronic active lesions.

RNA sequencing

Total RNA was prepared from 200,000 spinal cord FACS sorted $Ly6G^-CD3^-CD45^+CX3CR1^+YFP^+$ or 100,000 $Ly6G^-CD3^-CD45^+CX3CR1^+YFP^-$ cells of EAE suffering *Arg1*-eYFP animals at peak-disease using TRIzol Reagent (Ambion, #15596018) and quantified using the Qubit 2.0 Fluorometric Quantitation system (Thermo Fisher Scientific, Waltham, MA, USA). RNA integrity number (RIN) was determined using the 2100 Bioanalyzer instrument (Agilent, Santa Clara, CA, USA). Isolated RNA was subsequently utilized for RNA-Seq library preparation using the NEBNext® Ultra™ II Directional RNA sample preparation kit (New England Biolabs, Inc., Ipswich, MA, USA). NGS library concentrations were quantified with

Qubit 2.0 Fluorometric Quantitation system (Life Technologies, Carlsbad, CA, USA) and the size distribution was assessed using the 2100 Bioanalyzer instrument (Agilent, Santa Clara, CA, USA). For sequencing, samples were diluted and pooled into multiplex NGS libraries in equimolar amounts. Expression profiling libraries were sequenced on HiSeq 3000/4000 instruments (Illumina, San Diego, CA, USA) following a 50-base-pair, single-end recipe. Raw data acquisition (HiSeq Control Software, HCS, HD 3.4.0.38) and base calling (Real-Time Analysis Software, RTA, 2.7.7) was performed on-instrument, while the subsequent raw data processing off the instruments involved two custom programs (https://github.com/epigen/picard/) based on Picard tools (2.19.2) (https://broadinstitute.github.io/picard/). In a first step, base calls were converted into lane-specific, multiplexed, unaligned BAM files suitable for long-term archival (IlluminaBasecallsToMultiplexSam, 2.19.2-CeMM). In a second step, archive BAM files were demultiplexed into sample-specific, unaligned BAM files (IlluminaSamDemux, 2.19.2-CeMM). NGS reads were mapped to the Genome Reference Consortium GRCm38 assembly via "Spliced Transcripts Alignment to a Reference" (STAR)[2] utilising the "basic" Ensembl transcript annotation from version e100 (April 2020) as reference transcriptome. Since the mm10 assembly flavour of the UCSC Genome Browser was preferred for downstream data processing with Bioconductor packages for entirely technical reasons, Ensembl transcript annotation had to be adjusted to UCSC Genome Browser sequence region names. STAR was run with options recommended by the ENCODE project. NGS read alignments overlapping Ensembl transcript features were counted with the Bioconductor (3.11) GenomicAlignments (1.24.0) package via the summarizeOverlaps function in Union mode, ignoring secondary alignments and alignments not passing vendor quality filtering. Transcript-level counts were aggregated to gene-level counts and gene read counts were subjected to several processing steps for downstream analysis, mainly using R v4.2.1 (http://www.R-project.org/). First, counts per million (CPM) were obtained from read counts for gene filtering purposes. Genes were filtered to retain genes with CPM > 1 in at least 3 replicates and normalized with the limma-trend approach. Briefly, the limma-trend approach obtains normalized expression data through

taking $\log_2$(CPM) of read counts with an adjustment for sample read depth variance. Differentially expressed genes were detected using two-tailed moderated t-tests, implemented in the *limma* R package v3.52.4[3]. Significant differences (false discovery rate (FDR) < 0.05) between up- and downregulated genes in $Ly6G^-CD3^-CD45^+CX3CR1^+YFP^{+/-}$ cells ($|\log_2 FC| > 1$) were depicted using a volcano plot. Enrichment statistics for Gene Ontology (biological process) and REACTOME pathways of differentially expressed genes between samples were computed on the basis of pre-ranked gene-level t-test statistics using fgsea R package (https://doi.org/10.1101/060012). Empirical null distribution was computed from the enrichment score using random gene sets of the same size and p-values determined for normalized enrichment scores and adjusted for multiple hypothesis testing using Benjamini and Hochberg correction (FDR < 0.01).

Polyamine measurements of CSF and spinal cord tissue

Polyamine derivatization and LCMS analysis was conducted as previously described[4,5]. Dried metabolite extract from cerebral spinal fluid and spinal cord tissue were resuspended in 200 μl water spiked with 1 μg/mL of 1'6-Diaminohexane (Toronto Research Chemicals D416170) as a nonendogenous polyamine internal standard (ISTD) for derivatization efficiency. Sample pH was adjusted to 9 using 1 M sodium bicarbonate (VWR BDH9280) solution. Derivatization was performed by adding 20 μl of isobutyl chloroformate (Sigma 177989) followed by an incubation period of 15 min at 37 °C. Derivatized polyamines were collected via phase separation by mixing each sample with 1 mL of diethyl ether (Sigma 309966) and collection of the organic layer. Samples were dried in a nitrogen evaporator and resuspended in 50/50 v/v LCMS grade water/acetonitrile (Fisher Scientific W6-4/Fisher Scientific A955-4). D8-Tryptophan (Cambridge Isotope Libraries DLM-6903) was added to the resuspension solvent at 10 μg/mL as an internal standard to track instrument performance.

Polyamines were analyzed via LC-MS on a Vanquish liquid chromatography system coupled to an Orbitrap ID-X (Thermo Fisher Scientific) using an H-ESI (heated electrospray ionization)

source in positive mode[5]. 2 µL of each standard and/or sample was injected and run through a 15 min reversed-phase chromatography CORTECS T3 column (1.6 µm, 2.1 mm × 150 mm, 186008500, Waters, Eschborn, Germany) combined with a VanGuard pre-column (1.6 µm, 2.1 mm × 5 mm, 186008508, Waters). Mobile phase A consisted of 100% LC/MS grade water (W6, Fisher), 0.1% LC/MS grade formic acid (A117, Fisher Scientific), and Mobile phase B consisted of 99% LC/MS grade acetonitrile (A955, Fisher Scientific), 1% LC/MS grade water, 0.1% LC/MS grade formic acid. Column temperature was kept at 50 °C, flow rate was held at 0.4 mL/min, and the chromatography gradient was as follows: isocratic flow of solvent A from 0-0.5 min, 0.5-6 min from 0% B to 40% B, and 2-10 min from 40% B to 99% B, 10-15 min hold at 99% B.  A 15 min wash gradient was run between every injection (in parallel with an alternating chromatography method) to flush the column and re-equilibrate solvent conditions as follows: 0-11.4 min held at 100% B at 0.1 ml/min, from 11.4-11.5 min flow was transitioned to 0.6 ml/min of 100% A and held until 14.5 min. From 14.5-14.6 min flow rate was decreased to 0.4ml/min.

Mass spectrometer parameters were: source voltage 3500V, sheath gas 70, aux gas 25, sweep gas 1, ion transfer tube temperature 300 °C, and vaporizer temperature 250 °C. Full scan data were collected using the orbitrap with a scan range of 100-1000 m/z at a resolution of 60,000 and RF lens at 35%. A targeted mass list with 17 standard-verified derivatized polyamines was used to monitor for target polyamines of interest. Data dependent MS2 fragmentation was induced in the orbitrap and ion trap using assisted higher-energy collisional dissociation (HCD) collision energies at 20, 35, and 50%. Orbitrap resolution was 15,000 and the isolation window was 1.5 m/z while the ion trap scan rate was set at rapid with an isolation window of 1.5 m/z. Total cycle time was 0.8 sec. Peak picking was performed on analytical standard confirmed retention time, accurate mass, and MS2 (as listed in Supplementary Table 7) using Skyline Software (Version 25.1.0.237).

<u>Targeted metabolomics of *in vitro* samples</u>

Samples have been analyzed by liquid chromatography directly coupled to mass spectrometry (LC-MS/MS), using either reversed phase chromatography (RP) or hydrophilic interaction liquid chromatography (HILIC). Before RP-LC-MS/MS analysis, 100 µl of the extracts have been dried down in a vacuum centrifuge and have been resolved in 100 µl of acidified water (0.1% formic acid). Detection and quantification have been done by LC-MS/MS using an Ultimate 3000 HPLC system (Dionex, Thermo Fisher Scientific), directly coupled via electrospray ionization to a TSQ Altis mass spectrometer (Thermo Fisher Scientific). 1 µl of each sample has been injected onto a Kinetex (Phenomenex) C18 column (100 Å, 150 x 2.1 mm) connected with the respective guard column, and employing a 8-min-long linear gradient from 99%A (1% acetonitrile, 0.1% formic acid in water) to 80%B (0.1% formic acid in acetonitrile) at a flow rate of 80 µl/min. Employing selected reaction monitoring (SRM), the following transitions have been applied in the positive ion mode: *m/z* 61 to *m/z* 44 (urea), *m/z* 133 to *m/z* 70 (ornithine), *m/z* 175 to *m/z* 70 (arginine), *m/z* 176 to *m/z* 159 (citrulline), *m/z* 205 to *m/z* 188 (tryptophan) and *m/z* 241 to *m/z* 74 (cystine). In HILIC, 1 µl of the original sample was directly injected onto a polymeric iHILIC-(P) Classic HPLC column (HILICON, 100 x 2.1 mm; 5 µm) and the respective guard column, operated at a flow rate of 100 µl/min. A linear gradient (A: 95% acetonitrile 5%, 10 mM aqueous ammonium acetate; B: 5 mM aqueous ammonium bicarbonate, both supplemented with 0.1 µg/mL medronic acid) starting with 15% B and ramping up to 60% B in 9 min was used for separation. Using a TSQ Quantiva mass spectrometer (Thermo Fisher Scientific), he following SRM transitions were used for quantitation in the negative ion mode: *m/z* 87 to *m/z* 43 (pyruvate), *m/z* 89 to *m/z* 43 (lactate), *m/z* 239 to *m/z* 179 (glucose), *m/z* 259 to *m/z* 97 (glucose-6phosphate) and oxidized glutathione was measured in the positive ion mode (*m/z* 613 to *m/z* 355). In all cases, authentic standards have been used for determining optimal collision energies and transitions of the SRM transitions and for validating experimental retention times via standard addition to a pooled quality control sample. The data interpretation was performed using TraceFinder (Thermo Fisher Scientific).

Metabolomic and Lipidomic analysis of conditioned media and BMDM treated with EAE-CM

For metabolite extraction, 10 μL of the sample was transferred to an empty Eppendorf tube and mixed with 80 μL of methanol and 10 μL of an isotopically labelled internal standard. Each sample was vortexed for 15 seconds and then centrifuged for 10 min at 10000 RPM. The supernatant was transferred into an HPLC glass vial. Targeted quantitative metabolite analysis was conducted using HILIC-based liquid chromatography and mass spectrometric detection. Metabolites were separated on ACQUITY Premiere BEH Z-HILIC 1.7 µm 2.1 x 100 mm analytical columns (Waters) utilizing a gradient elution method with 0.15% formic acid and 10 mM ammonium formate in water as mobile phase A and a solution of 0.15% formic acid and 10 mM ammonium formate in 85% acetonitrile as mobile phase B with the total analysis time of 18 min. The mobile phase flow rate was set at 0.4 mL/min, the injection volume at 2 µL, and the column temperature at 40 °C. For MS detection, an Orbitrap Exploris 120 mass spectrometer (Thermo Fisher Scientific) was used. The MS analysis was performed in ESI positive and ESI negative modes using full scan detection. The scan range was set from 50 to 600 m/z, and the mass resolution was set to 60,000. The ESI spray voltage was set to 3.5 kV in positive mode and 2.5 kV in negative mode; the gas heater temperature was set to 400 °C; the capillary temperature was set to 350 °C; the auxiliary gas flow rate was set to 12 arbitrary units; and the nebulizing gas flow rate was set to 50 arbitrary units. For quantitative analysis, seven-point calibration curves with internal standardization were used. TraceFinder 5.1 General Quan (Thermo Fisher Scientific) software was used for LC-MS data processing and quantification. Every reported metabolite was identified at level A using an authentic standard compound that had been previously mapped to the analytical system.

For lipid extraction, 20 μL of the sample was transferred into a glass vial, followed by the addition of 10 μL of internal standard solution (SPLASH Lipidomix, Avanti Polar Lipids, Alabaster, AL) and 140 μL of methanol. After vortexing, 500 μl methyl-tert-butyl ether (MTBE) was added, and the mixture was incubated for 10 min at 21 °C on a shaker. Phase separation was induced by adding 130 μl MS-grade water, followed by another 10 min incubation at 21

°C. The samples were then centrifuged at 1000x g for 10 min, and 450 μl of the upper (organic) phase was carefully collected and evaporated to dryness under a stream of nitrogen. The dried lipid extracts were reconstituted in 100 μl methanol and subjected to LC-MS analysis. Chromatographic separation of lipids was carried out on an Accucore C18 column (2.1x 150 mm, Thermo Fisher Scientific) using a gradient elution with mobile phase A consisting of 0.1% formic acid and 10 mM ammonium formate in 50% acetonitrile, and mobile phase B composed of 0.1% formic acid and 10 mM ammonium formate in 10% acetonitrile and 88% isopropanol. The elution gradient was programmed as follows: from 0.0 to 4.0 min, mobile phase A was decreased from 65% to 40% and mobile phase B increased from 35% to 60%; from 4.0 to 12.0 min, A was further reduced to 15% and B increased to 85%; from 12.0 to 21.0 min, A was decreased to 0% and B increased to 100%, which was maintained until 24.0 min. At 24.1 min, the initial conditions (65% A/35% B) were restored and maintained until 28.0 min for column re-equilibration. The column temperature was set to 40 °C, with a flow rate of 0.4 mL/min and an injection volume of 2 μL. Mass spectrometric analysis was performed using an Orbitrap Exploris 120 high-resolution mass spectrometer (Thermo Fisher Scientific), operated in both electrospray ionization (ESI) positive and negative modes. Full scan acquisition was conducted over a mass range of m/z 150–1200 at a resolution of 60,000. The spray voltage was set to 3.5 kV in positive mode and 2.5 kV in negative mode. The capillary temperature was 350 °C, the gas heater temperature was 400 °C, the sheath gas flow rate was 50, and the auxiliary gas flow rate was 12. LC-MS data processing and quantification were performed using TraceFinder 5.1 General Quant software (Thermo Fisher Scientific). Lipid identification was based on accurate mass measurements at the MS1 level. For semiquantitative analysis, a one-point calibration was performed using lipid class-specific internal standards.

Targeted lipidomics data analysis

For data analysis of the individual lipid species, all values below detection threshold were replaced by the lowest detected value across all samples for each lipid species. Lipidomics data was then log2 transformed and differential abundance of individual lipid species was

determined using Empirical Bayes moderated t-test and the Benjamini-Hochberg correction for multiple testing from package limma (version 3.62.2) in R (version 4.4.2). For data analysis of the lipid classes, all individual lipid species values from raw dataset were summed by lipid class (disregarding the values below detection threshold), then log2-transformed. For heatmaps, the z-scores were calculated inside the pheatmap function (package pheatmap, version 1.0.13) by centering and scaling. To determine differentially abundant lipid classes, we used one-way ANOVA followed by Tukey's HSD test from package stats (version 4.4.2). For determining significance, a threshold of adjusted p-value $< 0.05$ was applied.

MALDI-MSI data acquisition and analysis

Spinal cords were embedded as previously described with slight modifications[6]. Briefly, 7.5 g hydroxypropyl methyl cellulose (HPMC) and 2.5 g polyvinylpyrrolidone (PVP) were added to 100 mL of deionized water, stirred for 1h, and kept at 4 °C overnight to ensure both polymers were dissolved completely. 3 mL aliquots were centrifuged for 5 min at 800 g to get rid of trapped air and stored at -20 °C until use.

Embedded mouse spinal cord tissue was cut into 12 μm thick sections (Leica CM1860 UV, Leica Biosystems, Nussloch, Germany) at -18 °C, mounted onto ITO slides (Bruker Daltonik, Bremen, Germany) and stored at -80 °C until use. Just before further processing, slides were brought to 21 °C and dried for 10 min in a vacuum desiccator. Optical images were acquired using a slide scanner (Leica CS2). Matrix solutions of 10 mg/mL 1,5-daminonaphthalene (DAN) were prepared in ACN/$H_2O$ (50:50, v/v) and in ACN/$H_2O$ (70:30, v/v) for hydrophilic metabolite and lipid measurements respectively. Matrix solutions were sonicated for 10 min and deposited onto slides using an M5 Sprayer (HTX Technologies LLC, Chapel Hill, North Carolina, USA). The nozzle temperature was set at 75 °C and 80 °C for the metabolite and lipid measurements respectively and the reagent was sprayed (6 passes) over the tissue sections at a velocity of 1200 mm/min with a track spacing of 2 mm (CC pattern) and at a flow rate of 100 µL/min. The gas pressure of the sprayer was set to 10 psi with a gas flow rate of 2

L/min and the nozzle height was set to 40 mm. The bed temperature for matrix application was set to 40 °C and 30 °C for the hydrophilic metabolite and lipid measurements respectively.

Data acquisition was performed on a MALDI-TIMS-QTOF mass spectrometer (TimsTOF flex, Bruker Daltonik) at 10 μm spatial resolution on replicate slides. Metabolites were measured in the negative ion mode (mass range [*m/z*] 50-650, 200 laser shots, laser frequency 10000 1/s, laser field size 10x10 μm). Transfer parameters for metabolite measurements were; Funnel 1 RF 150 Vpp, Funnel 2 RF 180 Vpp, Multipole RF 180 Vpp. Quadrupole parameters for metabolite measurements were; Collision Energy 7eV, Collision RF 650 Vpp, Low Mass [*m/z*] 100. Focus PreTof parameters for metabolite measurements were; Transfer Time 45 μs, Pre Pulse Storage 5 μs. Lipids were measured in both the negative ion mode (mass range [*m/z*] 200-2000, 250 laser shots) or positive ion mode (mass range [*m/z*] 300-2000, 250 laser shots, laser frequency 10000 1/s, laser field size 10x10 μm). Transfer parameters for lipid measurements in both modes were; Funnel 1 RF 400 Vpp, Funnel 2 RF 500 Vpp, Multipole RF 400 Vpp. Quadrupole parameters for lipid measurements in the negative mode were; Collision Energy 10 eV, Collision RF 1500 Vpp, Low Mass [*m/z*] 350. Focus PreTof parameters for lipid measurements in the negative mode were; Transfer Time 85 μs, Pre Pulse Storage 10 μs. Quadrupole parameters for lipid measurements in the positive mode were; Collision Energy 10 eV, Collision RF 1800 Vpp, Low Mass [*m/z*] 200. Focus PreTof parameters for lipid measurements in the positive mode were; Transfer Time 80 μs, Pre Pulse Storage 10 μs. Single point online calibration was performed using the following signals: Metabolites $[1,5\text{-DAN-H}]^-$ (*m/z* 157.0771); Lipids (negative mode) $[\text{C24:1 sulfatide-H}]^-$ (*m/z* 888.6240); Lipids (positive mode) $[\text{PC(34:1)+H}]^+$ (*m/z* 760.5851).

Post MSI data acquisition, the matrix was washed off by submerging the slides in ice-cold acetone for 5 min. Subsequently, hematoxylin and eosin (H&E) staining was performed using an automated staining apparatus (SunTissuePrep, SunChrom Wissenschaftliche Geräte GmbH, Friedrichsdorf, Germany). Briefly, Hematoxylin was applied to slides for 1 min, after which they were rinsed in tap and then distilled water. Acidic alcohol was applied for 1 min,

slides were washed with distilled water for 45 sec and bluing solution applied for 1 min. Post washing with distilled water (1 min), Eosin was applied to the slides for 2 min, after which they were washed with distilled water (45 sec) and dehydrated with graded ethanol (80% Ethanol 1 min, 90% Ethanol 2 min, 99% Ethanol 2 min) followed by Xylene application for 2 min. Slides were mounted using mounting medium (Eukitt, Sigma-Aldrich) and bright field images were recorded using a slide scanner (Aperio CS2, Leica Biosystems).

MSI data (centroided) was imported into SCiLS Lab 2023a Pro (Bruker Daltonik) and root mean square- normalized. Subsequently, data was exported as an imzML file and uploaded to (www.metaspace2020.eu)[7] and matched against the HMDB database[8] for annotation on the Level 2 according to the Metabolomics Standards initiative[9]. The resulting annotations were filtered to 10% false discovery rate (FDR), exported as csv file and reimported into SCiLS Lab. H&E stained images were also imported into SCiLS Lab and manually co-registered with the corresponding ion images. Lesions in EAE tissue were annotated on the co-registered H&E image, along with matched regions in the white matter of spinal cords from healthy control mice. The mean signal intensities of these annotated regions were extracted and used for relative quantification between metabolites and lipids between the two groups.

## References (Supplementary Methods)


1. Schmidt, U., Weigert, M., Broaddus, C. & Myers, G. Cell Detection with Star-Convex Polygons. *Lect Notes Comput Sc* **11071**, 265-273 (2018).

2. Dobin, A. *et al.* STAR: ultrafast universal RNA-seq aligner. *Bioinformatics* **29**, 15-21 (2013).

3. Ritchie, M.E. *et al.* limma powers differential expression analyses for RNA-sequencing and microarray studies. *Nucleic Acids Res* **43**, e47 (2015).

4. Isaguirre, C., Gendjar, M., Nauta, K.M., Burton, N.O. & Sheldon, R.D. Polyamine quantitation by LC-MS using isobutyl chloroformate derivatives. *Methods Enzymol* **715**, 437-458 (2025).

5. Nauta, K.M. *et al.* A noncanonical polyamine from bacteria antagonizes host mitochondrial function. *Nat Commun* **16**, 11638 (2025).

6. Dannhorn, A. *et al.* Universal Sample Preparation Unlocking Multimodal Molecular Tissue Imaging. *Anal Chem* **92**, 11080-11088 (2020).

7. Palmer, A. *et al.* FDR-controlled metabolite annotation for high-resolution imaging mass spectrometry. *Nat Methods* **14**, 57-60 (2017).

8. Wishart, D.S. *et al.* HMDB 4.0: the human metabolome database for 2018. *Nucleic Acids Res* **46**, D608-D617 (2018).

9. Sumner, L.W. *et al.* Proposed minimum reporting standards for chemical analysis Chemical Analysis Working Group (CAWG) Metabolomics Standards Initiative (MSI). *Metabolomics* **3**, 211-221 (2007).
