# Supplementary material for: Monocyte infiltration induces CNS arginine catabolism to fuel neuroinflammation
Source: Nat Immunol. 2026 May 18;27(7):1418–32. doi: 10.1038/s41590-026-02516-4 (PMC13310764; doi:10.1038/s41590-026-02516-4)
Supplement: Supplementary file 14 — Unprocessed western blots. [file 41590_2026_2516_MOESM14_ESM.pdf]

Supplementary Information

Unprocessed Western Blot (Extended Data Fig. 3b)

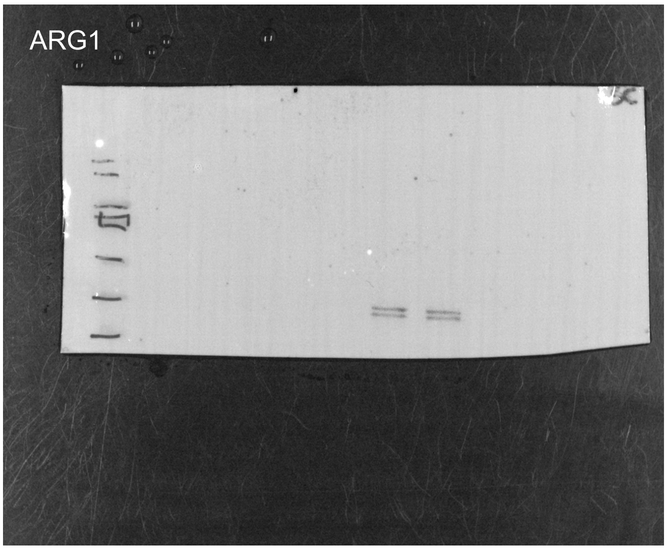

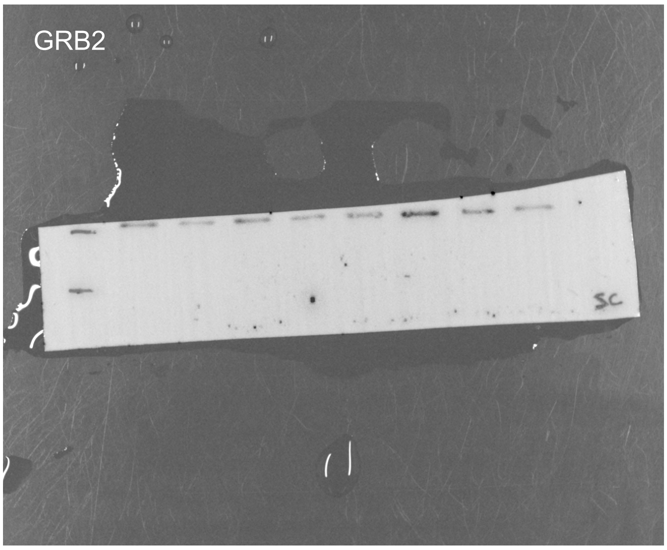

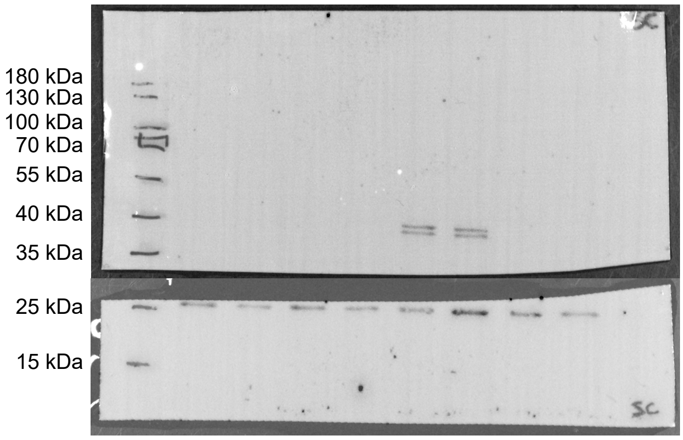

Marker: PageRuler™ Prestained Protein Ladder, 10 to 180 kDa (ThermoFisher, Cat.: #26616)
