# Supplementary material for: Monocyte infiltration induces CNS arginine catabolism to fuel neuroinflammation
Source: Nat Immunol. 2026 May 18;27(7):1418–32. doi: 10.1038/s41590-026-02516-4 (PMC13310764; doi:10.1038/s41590-026-02516-4)
Supplement: Supplementary file 3 — Peer Review file [file 41590_2026_2516_MOESM3_ESM.pdf]

nature portfolio

Peer Review File

Infiltration of monocyte-derived cells perturbs CNS metabolism licensing arginine catabolism augmenting neuroinflammation

Corresponding Author: Dr Omar Sharif

This file contains all editorial decision letters in order by version, followed by all author rebuttals in order by version.

Decision Letter:

16th Jul 2024

Dear Omar & Gernot,

Thank you for providing a point-by-point response to the referees' comments on your manuscript entitled, "Infiltration of monocyte-derived cells perturbs the CNS metabolic landscape, licensing arginine catabolism and augments neuro-inflammation". As noted previously, while they find your work of considerable potential interest, they have raised quite substantial concerns that must be addressed. In light of these comments, we cannot accept the current manuscript for publication, but would be very interested in considering a revised version that addresses these serious concerns along the lines proposed in your point-by-point rebuttal.

We invite you to submit a substantially revised manuscript, however please bear in mind that we will be reluctant to approach the referees again in the absence of major revisions.

Specifically, the revision should include new experiments to address:

(1) Using the alternative Ms4a3cre x R26TdTomato genetic model to track infiltrating monocytes into the CNS, using the Ms4a3-Cre driver to delete Arg1^f/f and subsequent CNS disease course/metabolomic analysis in the MOG immunization model

(2) Likewise, use the inducible Ccr2-cre^ERT2 x Csf2rb^f/f mouse model to interrogate the role of GM-CSF in CNS arginine metabolism during the EAE disease course

(3) Measure additional arginine metabolites, including urea, ornithine and polyamines during the EAE disease course

(4) Examine GCN2, Nrf2, and ATF4 protein expression by flow cytometry to support role for arginase in regulating iNOS and lipid peroxidation in EAE

(5) Examine lipidomics of unstimulated WT and Arg1-deficient BMDMs and those stimulated with EAE-induced culture media

(6) Reanalysis of human MS expression datasets to determine role for Arg1 and iNOS in disease stage

(7) Examine iNOS activity at different stages of EAE disease

Please include the additional textual clarifications as indicated in your response letter.

When you revise your manuscript, please take into account all reviewer and editor comments, please highlight all changes in the manuscript text file in Microsoft Word format.

We are committed to providing a fair and constructive peer-review process. Do not hesitate to contact us if there are specific requests from the reviewers that you believe are technically impossible or unlikely to yield a meaningful outcome.

When revising your manuscript:

* Include a “Response to referees” document detailing, point-by-point, how you addressed each referee comment. If no action was taken to address a point, you must provide a compelling argument. This response will be sent back to the referees along with the revised manuscript.

* If you have not done so already please begin to revise your manuscript so that it conforms to our Article format instructions at http://www.nature.com/ni/authors/index.html. Refer also to any guidelines provided in this letter.

* Include a revised version of any required reporting checklist. It will be available to referees (and, potentially, statisticians) to aid in their evaluation if the manuscript goes back for peer review. A revised checklist is essential for re-review of the paper.

The Reporting Summary can be found here:
https://www.nature.com/documents/nr-reporting-summary.pdf

When submitting the revised version of your manuscript, please pay close attention to our href="https://www.nature.com/nature-portfolio/editorial-policies/image-integrity">Digital Image Integrity Guidelines. and to the following points below:

-- that unprocessed scans are clearly labelled and match the gels and western blots presented in figures.
-- that control panels for gels and western blots are appropriately described as loading on sample processing controls
-- all images in the paper are checked for duplication of panels and for splicing of gel lanes.

Extended Data figures and tables are online-only (appearing in the online PDF and full-text HTML version of the paper), peer-reviewed display items that provide essential background to the Article but are not included in the printed version of the paper due to space constraints or being of interest only to a few specialists. A maximum of ten Extended Data display items (figures and tables) is typically permitted. When re-submitting your manuscript, please ensure that any supplementary figures and tables that are more critical to the manuscript’s conclusions are converted to Extended data to increase these data’s visibility.

Finally, please ensure that you retain unprocessed data and metadata files after publication, ideally archiving data in perpetuity, as these may be requested during the peer review and production process or after publication if any issues arise.

You may use the link below to submit your revised manuscript and related files:

Link Redacted

Note: This URL links to your confidential home page and associated information about manuscripts you may have submitted, or that you are reviewing for us. If you wish to forward this email to co-authors, please delete the link to your homepage.

If you wish to submit a suitably revised manuscript we would hope to receive it within 6 months. If you cannot send it within this time, please let us know. We will be happy to consider your revision so long as nothing similar has been accepted for publication at Nature Immunology or published elsewhere.

Nature Immunology is committed to improving transparency in authorship. As part of our efforts in this direction, we are now requesting that all authors identified as ‘corresponding author’ on published papers create and link their Open Researcher and Contributor Identifier (ORCID) with their account on the Manuscript Tracking System (MTS), prior to acceptance. ORCID helps the scientific community achieve unambiguous attribution of all scholarly contributions. You can create and link your ORCID from the home page of the MTS by clicking on ‘Modify my Springer Nature account’. For more information please visit please visit www.springernature.com/orcid.

Please do not hesitate to contact me if you have any questions or would like to discuss the required revisions further.

Thank you for the opportunity to review your work.

Kind regards,

Laurie

Laurie A. Dempsey, Ph.D.
Senior Editor
Nature Immunology
l.dempsey@us.nature.com
ORCID: 0000-0002-3304-796X

Version 0:

Reviewers' Comments:

Reviewer #1:
Remarks to the Author:
Review of "Infiltration of monocyte-derived cells perturbs the CNS metabolic landscape, licensing arginine catabolism and augments neuro-inflammation"
In this work, Kerndl and colleagues explored metabolic changes in spinal cord and CSF in an EAE mouse model, with an emphasis on arginine metabolism. The catabolism of arginine was driven by infiltrating monocyte-derived cells (Mdcs), which were highly positive for Arg1 and showed Slc7a2 expression. Arginine scarcity in the tissue was associated with increased oxidative damage and lipid accumulation and an overall worsened disease outcome. These findings were corroborated in a model employing an arginine-deficient diet, while on the other hand, blocking Arg1 in Mdcs led to better clinical scores, with higher IL-10 and TGFβ production in Mdcs and Treg expansion.
Overall, the findings in this work are interesting and of translational potential. Monocyte influx into the CNS has been characterized as unfavorable in different neuropathological contexts. The detailed metabolomics data in different EAE stages are state of art. Thus, this work has value by describing the consequences of monocyte influx on CNS tissue metabolism.
On the other hand a substantial part of the provided immunological evidence has been reported before (such as Arg-1 expression specifically by infiltrating myeloid cells EAE). Finally, the clarity of the information presented is partially insufficient and overstatements are repeatedly made ("strong tendency").
Major points:
The paper emphasizes the role of Mdcs in arginine consumption in comparison to other myeloid populations in the CNS. That Arg-1 is specifically expressed in infiltrating myeloid cells in EAE has been shown several years ago, yet whereas the paper by the Becher group is cited twice (see below), the older paper by Greenhalgh et al. is not cited (PMID: 27126514)
Moreover, the methodology used to delineate different myeloid populations in EAE lacks precision. In particular, CNS-associated macrophages (CAMs) are mentioned in the introduction, yet their contribution is not considered in further analyses.
1) The authors heavily rely on CX3CR1 and CCR2 expression when defining CNS myeloid populations. The authors should emphasize more the limitations of the models (e.g. when using Cx3cr1cre mice).
2) Fig. 2i: This analysis needs quantification (relative proximity to lesions).
3) MS samples: How do the authors come to the conclusion that Arg1 expression is linked to Mdc infiltration?
4) Fig.3a: authors sort CX3CR1+YFP+ and YFP- cells from Arg1tm1Lky animals and see significant upregulation of Mrc1 in YFP+ population. Yet this could very well be due to the contribution of CAMs.
5) Microglia was defined as CD11b+CCR2neg,CX3CR1pos, CD45mid, P2RY12pos. Yet, gating strategy in extended Fig.3d shows microglia in CX3CR1neg gate, and gated from CD45mid but without the discrimination based on CD11b.
6) Extended.Fig.4: It is mentioned that activated microglia upregulates CD45. However, it seems essential to consider that P2RY12 is downregulated in EAE microglia (Jordão et al, Science 2109). In Ex.Fig.4c, the expression of Arg1-YFP is shown in CD45high activated microglia. The authors should show what kind of gating strategy was employed for the identification of this population. In Ex.Fig. 4a-b the frequency of moDCs and moMACs appears to be much higher than the frequency of microglia, differing from the quantitative analysis shown in Fig.1. This should be explained.
7) The definition and separation of moMACs and moDCs should be better. There is no gating discrimination in Ex.Fig.4a regarding CD44, although authors call moMACs CD44hi and moDCs CD44+. Usage of other markers (e.g., CD64, MHC-II, CD16/32, MerTK) might be useful for clearer differentiation between these two populations.
8) Ex. Fig. 6 and 7: The statement that Slc72ahigh cells express both chains of the CSF2 receptor has to be backed up by proper quantitative analysis.
9) Extended Fig. 7f: Important controls are missing, e.g. LPS alone, GM-CSF alone.
10) Ex. Fig.7: The importance of GM-CSF for Arg1-mediated arginine catabolism in Mdcs is interesting and should be analyzed in vivo, e.g., by using Ccr2creERT2;Csf2rbfl/fl mice. Furthermore, the authors could utilize the publicly available scSeq dataset from Csf2rb-/- chimeric mice in EAE model (Amorim et al), which already suggests that Arg1+ mononuclear phagocytes found in the CNS of peak EAE mice rely on GM-CSF signaling (Figure 6a in Amorim et al).
11) Fig. 5b and line 434: This wording here is misleading. Correct: The authors did not see a significant clinical effect of the arginine-free diet, there is nothing like a "strong tendency". In the same line: instead of changing between "ns" and "p=0.0547", either p-values have to be provided for each comparison, or only if below 0.05 (see for example the "strong tendency" in Fig. 5 e). Similar concerns exist the description of Fig. 5j.
Minor points:
- The language appears partially inflated (e.g. overuse of terms like "landscape", "rewiring", "orchestrate", and in particular "striking"). The language should be toned down. Terms like "intriguingly" should be avoided.
- It seems important to better define what the authors mean by meningeal macs: leptomeningeal? Dural? Both?
- Fig 1b: The colors of different experimental groups are in part very similar and not easy to distinguish, especially between peak and recovery groups. The names of the metabolites are not visible. It would be sufficient to indicate values of 5 - 10 metabolites (more relevant to the paper) on the bar plots. Y-axis labeling is also hardly visible.
- Fig 1d: Are CX3CR1+ cells also CCR2+? Here, the same labeling of populations as in Ex.Fig.1c,d should be employed. Please add the frequencies of same populations from recovered mice.
- Fig.1e,f: The metabolites that are increased or decreased in both spinal cord and CSF should be somehow indicated (bold letters, arrows, etc.)
- Ex.Fig.1d: Could authors show additionally Ly6C expression as a dotplot?
- Ex.Fig.1e: In T-cell gating, please add FMO for CD3 staining.
- 73-75: Macrophages in the dura are also replaced by monocytes (Van Hove, Nat Neuroscience 2019.)
- 84: Authors state that Mdcs do not contribute to the microglial pool, however, this is disease specific, e.g., monocytes can

engraft in cases of bacterial meningitis (Djukic et al, Brain 2006.).
- 145: There is no Ly6C expression shown in Ex.Fig.1c, Ex.Fig.1d should be shown as dotplots as well.
- l275: "Orchestrated" should be replaced by "associated with"
- Fig 2a: Plotting spinal cord and CSF FDR of MSEA together on the same plot would make data clearer.
- Fig 2b, c: Why is there no data on recovered animals?
- Fig 2e: The scheme of urea cycle should come before Fig 2b.
- Ex.Fig.3a: Could authors confirm that Arginase2 expression between heathy and peak/recovery group is indeed insignificant?
- Ex.Fig3e: It should be elaborated on the chosen timeline, e.g. on the reason for a break in tamoxifen provision before EAE induction. The labeling of CCR2+ progeny seems to be transient, e.g., blood monocytes drop their Tomato expression to 10.6% after a week of discontinued tamoxifen. Using reporter mice that allow for a long-term monocyte labeling would resolve possible off-target effects of long-term tamoxifen usage. Considering it was previously shown that all moMACs and moDCs stem from GMP (Amorim et al), Ms4a3cre;R26TdTomato mice would be appropriate alternative.
- The authors should comment on the discrepancies in percentages between Ex.Fig.3c and g?
- Fig 2i: Please use colorblind-friendly colors, e.g., green+magenta? The addition of the single channel pictures is needed. IF staining using IBA-1 as a microglial marker to confirm less Arg1 expression in comparison to Mdcs is advised.
- 303: There are 6 myeloid clusters, not 5.
- 304-306: This is an overstatement, Fig 3e doesn't confirm Arg1+ Mdcs differentiate into Arg1+ moMACs.
- Fig 3f: What are the identifying genes of cluster 18? If it represents monocytes/macrophages, shouldn't it be Ly6c2 positive as well? Could you show representative tSNE for other markers used throughout the paper, e.g. Ccr2, Cx3cr1, Adgre1?
- 330: Csf2ra and Csf2rb seem to be expressed heterogeneously across multiple clusters and not just on moMACs, could authors comment on it? More objective approach would be using violin plots in order to compare Csf2ra and Csf2rb expression across populations.
- 335: The overusage of abbreviations is confusing and should be avoided (LG, LI etc.), i.e. replaced by the established abbreviations of the stimulants.
- 372: "strong tendencies" do not exist in the scientific context (s. a.).
- Fig. 5: "Undesireable" should be omitted from the title, it is not s scientific term. In the same direction, the title of fig. 4 seems inappropriate ("detrimentally rewiring…)
- Ex.Fig. 7a: Based on previously published data, one would expect higher GM-CSF levels during peak disease. On the presented heatmap the levels do not seem to be significantly increased. The authors should comment and provide an additional bar plot.
- Ex.Fig 7d: Is there a significant difference between % of Arg+iNOS+ cells in LIG vs LG groups? Why is LI group used as a baseline to compare other conditions to?
- Ex.Fig.7f: Pleaae show arginine levels in IL-4/IL-13 condition?
- Ex.Fig.7g: Pleasse show CellRox for other conditions, e.g., LPS alone.
- Ex.Fig.7c,d: The unstimulated condition should be provided as in f.
- 345-348: The conclusion lacks the GM-CSF-related findings.
- Ex.Fig 7h: Arg1 expression should be analyzed in CX3CR1+ cells.
- 367-368: Strong statement based on in vitro data. In vivo confirmation would be necessary (check major points).
- Ex.Fig.8a: Arg1 depletion is not complete in Cx3cr1Cre/+;Arg1fl/fl animals. This should be commented on.
- Ex.Fig.8a,d: There seems to be a significant difference between Arg1 expression from Argfl/fl mice in two different models: Cx3cr1cre and CCR2creERT2. This should be explained. Also, it seems that Arg1fl/flCcr2creERT2 mice have an overall lower clinical score than Arg1fl/flCx3cr1cre/+ mice, and the littermate controls seem to be significantly different as well (Ex.Fig.8f and Fig.4a). This should be discussed.
- Ex.Fig.8: The plots for IL-10 and TGFβ in both genotypes should be shown.
- 444-445: Although it is mentioned here, the Mdcs frequency is not shown. In Fig5e: What is the percentage of CX3CR1+CCR2+ cells in control vs. arginine free-diet?
- 467 – 473: This paragraph should be worded more modestly and inserted into the discussion section.
- 575-576: Was CD11b used for gating on myeloid population?
Statistics:
- Overall authors use appropriate statistics. In Ex. Fig. 3c and g, error bars should be added to the bar plots. In certain figure legends the numbers of experimental repetitions are missing.
References:
- Amorim et al. appears twice (#9 and #36)
- In lines 151-154, authors claim that quinolinic acid and α-aminoadipic acid are produced by microglia and CAMs. For quinolinic acid, citation of the original data is missing (Heyes et al, Biochem 1996, Espey et al, Neuroreport 1997.). The citation showing CAMs and microglia are the producers of α-aminoadipic acid is missing.
-489-490: Citation is missing.
- A previously published report showed a decrease in arginine in rat CSF in EAE, which should be cited (Noga et al, Metabolomics 2012).
Spelling and editing:
- 78-79: Where they can differentiate into dendritic cells (moDCs) or macrophages (moMACs), with CCR2 being important for…
- 148: Having established that our experimental…
- 153: …lipid peroxidation, while oligodendrocyte and neuronal death…
-154: This, along…
- 158: …energy metabolism imbalances may be compensated…
- 314: demonstrated that, versus…
- 342: Comma missing after "notably"

- There are inconsistencies in how mice lines are written throughout the text and in the figures (e.g., Ex.Fig.8e and f).
- 502: If "akin to others" implies that similar Arg1 expression in microglia is reported in other papers, then citation is missing. If this sentence has other meaning, rephrasing is necessary for clarity.
- 534-535 and 543-544 is somewhat repetitive, both mention arginine scarcity affecting IL-10, TGFβ and Treg abundance.

Reviewer #2:
Remarks to the Author:
In this manuscript, Kerndl et al. examine the role of arginine metabolism in the pathophysiology of EAE using several elegant animal models of arginine perturbation and dietary experiments. While the data is somewhat interesting, the lack of strong human data, unusual choice of models, and unclear mechanism without strong supporting evidence give the findings a very correlative quality with an underwhelming mechanistic interpretation. Addressing the comments below will help strengthen this manuscript's observations:
• No cellular metabolism measurements are performed on Mdcs/MG of these mice. Using 13C-tracing to definitively show the urea cycle versus creatine/polyamines is crucial to support this work. It is important to show the fate of arginine in macrophages with Arg1 KO.
• Healthy composition in Figure 1d would be useful.
• Figure 2C, what does recovery look like for arginine? Related, if the authors are trying to make an argument about arginine availability to tissues why does arginine depletion not come up in the initial metabolomics of CSF?
• Urea measurements are missing from this work; as the end product of the urea cycle, this data is needed.
• What is the status of polyamines, which are also downstream of Arg1 activation?
• Regarding "Together, these observations suggest that at peak disease, subsets of Arg1+ Mdcs within demyelinating lesions deprive the local microenvironment of arginine," you need to show that arginine levels are maintained with Arg1 KO in the CSF.
• You have Tregs in Figure 4 but not in Figure 1. What do Tregs look like at recovery?
• The MOG-EAE model is supposed to be acute, not relapsing-remitting. Why use this model to study the "recovery" phase? This is a significant limitation of the work.
• Lipid peroxidation probes are insufficient for measurement in Mdcs, especially as a mechanistic insight. How is arginine perturbing lipid peroxidation, this is essential to the mechanism of this study, and provide something beyond correlative measurements.
• Please show flow gates for IL10 and TGFB staining in Figure 4.
• Panel 5G needs quantification.
• The differences in Panel 5K are marginal and not convincing.
• In Figure 5, the change in clinical score is non-significant, and even if it was, its biological relevance is doubtful.
o Does arginine free chow attenuate the clinical benefits of the Arg1 conditional knockout mice? This would strongly support the hypothesis
• Figures 4F and 5I are both at peak disease, yet the percentages are not even close to one another. What does your Foxp3 staining look like? Are you using the FJK16S clone?
o The work surrounding the Treg data is underdeveloped without any mechanistic insight.
• There's a lot of correlative data, without any direct examination of the role of arginine in EAE. The only mechanism proposed is oxidative damage, and that is underdeveloped.

Reviewer #3:
Remarks to the Author:
Summary of Key Results
Kerndl et al. (2024) investigated the metabolic changes that occur in the central nervous system (CNS) during inflammation using the MOG EAE mouse model of multiple sclerosis (MS). The authors analysed different phases of disease and found reduced arginine levels in the spinal cord and an increase of downstream metabolites, suggesting catabolism, especially in the acute inflammatory phase of EAE. This finding was associated with the accumulation of arginase 1 (Arg1) expression by monocytes/macrophages. Further genetic deletion of Arg1 in myeloid cells/monocytes reduced arginine catabolism and improved disease outcomes, suggesting a key role of arginine catabolism in neuroinflammation. Accordingly, the authors found that arginine deficiency worsened the disease, highlighting the importance of maintaining adequate arginine availability in neuroinflammation.

Originality and Significance
The role of arginine in immune regulation has been previously investigated, and other studies have shown that during EAE, CNS-infiltrated monocytes differentiate into an Arg1-expressing immune modulatory state, which may facilitate tissue repair (PMID: 29283442 and PMID: 30127427). Herein, the authors provide a somewhat different/complementary interpretation of the functional role of this population, which is strengthened by the use of cell specific genetic knockout animals. Similarly, while Arg1 expression has been shown in patients with MS, specifically PBMCs (PMID: 30128151), an extensive characterisation of its expression in MS lesions was lacking and is presented in this article. This is very important, given that previous data suggest that specific metabolic pathways (including Arginase metabolism) are different in mice vs humans during CNS inflammation (PMID: 25339954).

Data & Methodology
The authors used a combination of ex vivo targeted metabolomics, MALDI-MS, gene expression (sequencing and re-

analysis of publicly available snRNAseq datasets) to investigate the metabolic changes in the CNS during neuroinflammation. This multi-omics approach provided a complementary view of the metabolic landscape and allowed for the identification of key metabolic pathways, even though many of the observed findings (especially those in Figure 1 to 3) seem to be mostly correlative. The use of transgenic tools partially overcomes this limitation, but there are several and important drawbacks that still need to be addressed (see below).

Appropriate Use of Statistics and Treatment of Uncertainties
The authors used appropriate statistical tests to analyze their data and reported the results with standard errors of the mean (SEM). They also acknowledged the limitations of their study, such as the use of a mouse model and the small sample size for some experiments.

Conclusions
The conclusions drawn by the authors are most of the times supported by their data. However, the manuscript also includes sentences that are not supported by the authors' experimental results. For example, lines 155-175 include a very speculative description of the significance of metabolic changes without any experimental data to support "impaired oligodendrocyte mitochondrial ATP production and survival". Also, the interpretation of other metabolic changes, e.g. decreased reduced glutathione, is biased towards the peak-disease while exactly the same changes are present in the remission phase of disease as well (thus suggesting that oxidative stress is also present in chronic EAE tissues). These are two key examples of overstatements and interpretation of data, which should be corrected through the manuscript.

Suggested Improvements
Potentially being a very important and significant step towards the understanding of the contribute of cell metabolism in modulating monocyte/macrophage function, this paper falls short in some areas that require further major experiments to fully assess the validity of the proposed findings. These include:
1. Arg1 metabolism in the spinal cord and CSF. Data for known metabolites that play a role in arginine metabolism (ornithine, urea) are missing from the heatmaps of Fig 1 and Extended data Fig 2. If the problem is that these are not significant, the authors should provide these data nonetheless (including a power analysis to prove that their experiments were not underpowered). These are important to understand the preferential use of the Arginine substrate towards a certain pathway. Please provide raw data, normalised data, and statistical significance for all the metabolites tested in an excel file.
2. Arg1/iNOS activity. The authors need to provide additional evidence to show how the expression of genes regulating both Arg1, Arg2, and iNOS and their enzymatic activity changes in the EAE tissues and in ex vivo isolated cells at different phases of disease.
3. Fig 2i does not show clearly the colocalization of markers. Separation of single stainings in different panels and quantification of single/double labelled cells in EAE peak and remission is needed.
4. Human pathology. The authors need to show if there are any differences in Arg1 expression in the different MS subtypes. Also, they need double immunolabelling for a myeloid cell marker (besides CD68, in Fig 2l, which is also not convincing without proper z stack) vs astrocytic and neuronal markers, to show staining specificity. iNOS and Arg2 stainings for all these conditions should be also provided.
5. SnRNAseq human datasets. These are needed to confirm the pathology data and provide an unbiased assessment of Arg1/2/inos expression in different cell subtypes in MS and controls. A reanalysis of available datasets should be sufficient.
6. In situ metabolomics. MALDI-MS data are partial and do not assess many of the metabolites detected in Fig1. Even given differences in methodologies, this should be addressed, as there is a lack of correspondence between the two datasets that makes most interpretations correlative at most. There is also a need to provide better images without artifacts (e.g., see ganglioside levels found increased in the tear of the tissue of the peak EAE mouse of Extended Data Fig 5) with better/higher magnification images (showing at least markers for myeloid cells in the interested areas). Finally, the authors should provide raw data, normalised data, and quantification with statistical significance for all the metabolites tested in an excel file comparing different spinal cord regions.
7. Reanalysis of the Wheeler dataset. This approach is instrumental to the authors' paper, but data need to be stratified in the different phases of EAE (priming, peak, remission) instead of being presented altogether. Also, the authors should use these data to exclude that the increased arginase activity is related to other cell types beyond myeloid cells. Please note that cluster 1 in Fig 3i is microglia/macrophages and not only macrophages. Also, the sub-clustering of Slc7a2high and Slc7a2low cells in in Extended Data Fig 6c seems to be including way more cells than those reported to express this gene in Extended Data Fig 6b. Finally, to see what genes are varying with the Arg1 cluster, an analysis of covariance (e.g. MOFA) is needed.
6. In vitro experiments. Data from unstimulated controls is missing from Extended Data Fig 7d, as well as IL-4/13 stimulated controls are missing from Extended Data Fig 7e. Please keep the experimental groups consisting throughout Extended Data Fig 7. Moreover, what is the metabolic and proteomic composition of the EAE CM experiments? This seems rather a crude experimental set up with many potential confounding players. Finally, what is the link/mechanism regulating arginine metabolism and the observed alterations of lipid metabolism? Metabolic flux data are needed to understand how arginine catabolism affects the intracellular and extracellular metabolome/lipidome of myeloid cells.
8. Mechanistic studies to prove toxicity. These are needed. They should include direct neurotoxicity and oligotoxicity of Arg1+ vs Arg1- BMDM in vitro, as well as neuropathological data (immune cell subtypes, Bielschowsky, and LFB) from all EAE studies.
9. Transgenic mice. Please note that all the experiments (in vitro and in vivo) using the Arg1fl/fl Cx3cr1cre/+ mice need an additional control. That is Arg1wt/wt Cx3cr1cre/+ mice. The reason being that it is known that the expression of the cre under the cx3cr1 promoter generates a partial loss of function for Cx3cr1, which could affect the results. Moreover, all the experiments with KO animals need to be accompanied by quality controls showing the efficient knockout of the target gene and protein over time (this is particularly important for the CCR2 promoter, given that the paradigm of tamoxifen treatment was likely chosen to optimise this). Finally, MALDI-MS data for these in vivo transgenic experiments are likely needed to tie

this paper together.
10. Arginine deficiency or supplementation. Arginine, being an essential amino acid, is only partially regulated by diet and can be synthetised locally. Therefore, the authors need to prove how their arginine free diet (and L-arginine supplementation) impact the dynamics and levels of circulating arginine, at different time points, and in the CNS.

References
The authors appropriately cited previous work and acknowledged the contributions of other researchers in the field. However, they should provide a more comprehensive list of references when describing the metabolic changes seen in the CSF/CNS using mouse models of MS (e.g., PMID: 22448154, 29478844, 36210459) and correlate them with their own findings. Also, the potential controversy around the differences of arginine metabolism in monocytes from mice and humans needs to be discussed (PMID: 30467554).

Clarity and Context
The abstract, introduction, and conclusions of the article are quite clear. The main problem (besides the issues with some of the conclusions, as written above) is that at least in two occasions, figures/panels are not cited in order of appearance in the text, making it unnecessarily hard to follow the results. For example, the authors reference Extended Data 1a, b, in line 123, which is followed by Extended Data 2a, b, in line 125. But then the authors go back to Extended Data 1c in line 142, instead of continuing their numbering with Extended Data 2. Same thing happens for the panels i-l of Figure 3. Finally, the choice of referring to disease-associated metabolites as DAMs is highly questionable, given that the scientific community preferentially uses “DAM” to refer to disease associated microglia instead (PMID: 29775591).

Decision Letter:

Our ref: NI-A38081A

6th Mar 2026

Dear Omar & Gernot,

Thank you for submitting your revised manuscript "Infiltration of monocyte-derived cells perturbs the CNS metabolic landscape, licensing arginine catabolism and augments neuroinflammation" (NI-A38081A). It has now been seen by the original referees and their comments are below. The reviewers find that the paper has improved in revision, and therefore we'll be happy in principle to publish it in Nature Immunology, pending minor revisions to satisfy the referees' final requests and to comply with our editorial and formatting guidelines.

We will now perform detailed checks on your paper and will send you a checklist detailing our editorial and formatting requirements in about two weeks. Please do not upload the final materials and make any revisions until you receive this additional information from us.

If you had not uploaded a Word file for the current version of the manuscript, we will need one before beginning the editing process; please email that to immunology@us.nature.com at your earliest convenience.

Thank you again for your interest in Nature Immunology Please do not hesitate to contact me if you have any questions.

Kind regards,

Laurie

Laurie A. Dempsey, Ph.D.
Senior Editor
Nature Immunology
l.dempsey@us.nature.com
ORCID: 0000-0002-3304-796X

Version 1:

Reviewer comments:

Reviewer #1

(Remarks to the Author)
The authors have strengthened their findings by introducing new experiments and employing several new mouse lines. Overall, they have substantially improved the manuscript and appropriately addressed the major concerns raised previously. However, several minor points should be addressed:
-Extended figure 4d and 4i: The authors claim that moMACs display higher CD44, CD64, and MerTK expression, but only histograms are provided. A graph showing MFI comparisons would be necessary to support this claim.
-Extended Fig 4f: The x-axis in labeled “CCR2-Tomato” instead of “Ms4a3-Tomato”
-Extended Fig. 8c, g: It would be helpful to include the full gating strategy.

-73-76: Rewording is needed. Cited papers haven't shown that macrophage populations in choroid plexus and dura are dependent on monocyte trafficking, but rather that they are replenished by monocytes.
-154: Add comma after "This".
-210: Add comma after "This".
-344: Missing word: flow cytometry demonstrated that…
-445: Missing word: illustrate that Arg1-expressing…
-392-394: This sentence should be split in two for readability.

Reviewer #2

(Remarks to the Author)
I am not persuaded by the RRR or technical feasibility argument as justification for not performing carbon tracing. Such experiments could reasonably be conducted ex vivo using isolated myeloid populations (e.g., Gr1-based enrichment) to directly assess arginine fate.

That being said, this represents only one outstanding point among many, and the authors have substantially strengthened and clarified the manuscript in most other areas.

Reviewer #3

(Remarks to the Author)
The revised submission from Kerndl et al. is substantially strengthened compared to the original version, and the authors have been broadly responsive to many of the major issues I raised—particularly around overstatement, inclusion of key arginine-pathway metabolites, improved figure clarity/quantification, expanded data availability, and strengthened computational and human-tissue support. Overall, the revision improves the mechanistic coherence of the central model (Arg1-associated arginine catabolism in CNS inflammation contributing to disease severity) and increases transparency. That said, I think a few additional points should be highlighted to appropriately calibrate the strength of the conclusions and the degree of "mechanistic closure".
My remaining concerns are therefore:

1. MALDI-MSI triangulation vs bulk metabolomics. The authors did not expand MALDI-MSI to cover a broader set of metabolites detected by bulk metabolomics, so MALDI-MSI remains supportive rather than a strong cross-validation layer. The manuscript should avoid implying stronger concordance between modalities than is demonstrated and ensure the spatial data are interpreted conservatively.

2. EAE-conditioned media model complexity and mechanistic link to lipid metabolism. Despite the new targeted metabolite characterisation of EAE-CM (arginine↓ with ornithine/urea↑) and expanded lipidomics/perturbation data (including an effect of arginine addition on lipid loading), the mechanistic link between arginine catabolism and lipid remodelling remains incompletely resolved (as the authors appropriately acknowledge). Conclusions here should remain framed as associative/perturbational rather than definitive pathway mechanisms. Reviewer #1 also notes that the GM-CSF dependence of this axis is interesting but remains largely supported by in vitro evidence; in vivo genetic or dataset-level substantiation would strengthen causality (or otherwise needs careful wording).

3. Dietary arginine manipulation kinetics. The authors show reduced blood and CSF arginine levels under an arginine-free diet, supporting that the manipulation affects relevant compartments. However, longitudinal/phase-resolved kinetics during the EAE time course are not provided. This is not necessarily fatal, but it limits claims about temporal coupling between diet, systemic/CNS arginine availability, and disease trajectory.

4. Transgenic control concerns (Cx3cr1-Cre haploinsufficiency; longitudinal KO validation). The main methodological caveat that remains is the absence of Cre-only controls (Arg1wt/wt Cx3cr1cre/+), given the known biology of Cx3cr1 haploinsufficiency. The authors argue that the directionality of published Cx3cr1 phenotypes makes confounding unlikely and acknowledge the limitation in the Discussion; nevertheless, the lack of this control leaves residual interpretational uncertainty. Similarly, while attenuation of Arg1 protein at peak disease is shown, longitudinal recombination/KO efficiency across the disease course is not demonstrated.

5. Precision of myeloid population definitions / potential CAM contribution. Some conclusions rely on delineation of CNS myeloid subsets, and (as also noted by another referee) the manuscript should ensure that definitions/gating and terminology around microglia versus infiltrating monocyte-derived cells versus CNS-associated macrophages are precise, or otherwise explicitly acknowledge this as a limitation where it bears on specific interpretations.

6. Statistical/language overreach ("trend" wording; reporting consistency). Remaining "trend/strong tendency" language and any inconsistencies in p-value reporting should be avoided to prevent over-interpretation of non-significant effects, and statistical reporting should be consistent across the manuscript.
In my view, the revision has substantially improved the manuscript and addresses many technical/presentation concerns. On balance, I consider the manuscript potentially publishable in Nature Immunology provided the authors substantially constrain causal/mechanistic claims (especially around arginine depletion being driven by Arg1+ lesion myeloid cells and the lipid peroxidation mechanism) and clearly acknowledge limitations of model choice and missing flux/urea-in vivo validation. If the journal/editorial standard for this paper requires direct demonstration of metabolic flux, additional

experimentation may still be warranted.

Open Access This Peer Review File is licensed under a Creative Commons Attribution 4.0 International License, which permits use, sharing, adaptation, distribution and reproduction in any medium or format, as long as you give appropriate credit to the original author(s) and the source, provide a link to the Creative Commons license, and indicate if changes were made.
In cases where reviewers are anonymous, credit should be given to 'Anonymous Referee' and the source.
The images or other third party material in this Peer Review File are included in the article's Creative Commons license, unless indicated otherwise in a credit line to the material. If material is not included in the article's Creative Commons license and your intended use is not permitted by statutory regulation or exceeds the permitted use, you will need to obtain permission directly from the copyright holder.
To view a copy of this license, visit https://creativecommons.org/licenses/by/4.0/

Reviewer #1

Review of "Infiltration of monocyte-derived cells perturbs the CNS metabolic landscape, licensing arginine catabolism and augments neuroinflammation"
In this work, Kerndl and colleagues explored metabolic changes in spinal cord and CSF in an EAE mouse model, with an emphasis on arginine metabolism. The catabolism of arginine was driven by infiltrating monocyte-derived cells (Mdcs), which were highly positive for Arg1 and showed Slc7a2 expression. Arginine scarcity in the tissue was associated with increased oxidative damage and lipid accumulation and an overall worsened disease outcome. These findings were corroborated in a model employing an arginine-deficient diet, while on the other hand, blocking Arg1 in Mdcs led to better clinical scores, with higher IL-10 and TGFβ production in Mdcs and Treg expansion.
Overall, the findings in this work are interesting and of translational potential. Monocyte influx into the CNS has been characterized as unfavorable in different neuropathological contexts. The detailed metabolomics data in different EAE stages are state of art. Thus, this work has value by describing the consequences of monocyte influx on CNS tissue metabolism.
On the other hand a substantial part of the provided immunological evidence has been reported before (such as Arg-1 expression specifically by infiltrating myeloid cells EAE). Finally, the clarity of the information presented is partially insufficient and overstatements are repeatedly made ("strong tendency").

Response: We thank reviewer #1 for the deep review, stating the manuscript is interesting, includes "state-of-art metabolomics data" and the shared views on the scope of the work. Please find our point-by-point response to the comments raised below, including differences and similarities between our work and that previously reported. Further, we have removed terms like "strong tendencies" and significantly toned down on language.

<u>*Major points:*</u>
The paper emphasizes the role of Mdcs in arginine consumption in comparison to

other myeloid populations in the CNS. That Arg-1 is specifically expressed in infiltrating myeloid cells in EAE has been shown several years ago, yet whereas the paper by the Becher group is cited twice (see below), the older paper by Greenhalgh et al. is not cited (PMID: 27126514) Moreover, the methodology used to delineate different myeloid populations in EAE lacks precision. In particular, CNS-associated macrophages (CAMs) are mentioned in the introduction, yet their contribution is not considered in further analyses.

Response: While several different manuscripts have indeed demonstrated Arg1 is expressed by infiltrating Mdcs, there appears be no consensus on whether infiltrating Mdcs exclusively express Arg1 or co-express iNOS. The paper from the Becher group mentioned by the reviewer[1] gates for Mdcs as lin^{-}Cd11b^{+}CD88^{+}Ly6C^{+} (excluding CD45^{lo}CD11b^{+}CD88^{-} microglia) and then demonstrates the kinetics of Arg1 and iNOS within all CNS infiltrating CD11b^{+} cells in EAE. This analysis reveals presence of both populations at peak EAE (Extended Data Fig. 1d, 1f of that manuscript). Other single cell RNA sequencing studies of Mdcs gated as CD11b^{+}Ly6G^{-} (excluding CX3CR1^{hi}CD44^{lo} microglia) have shown the presence of many different Mdc subsets including three distinct “Arg1^{+} macrophage populations”, two of which do not co-express *Nos2*, and a *Nos2* expressing cluster co-expressing Arg1[2]. Still other work utilizing the *Arg1*-eYFP animals (which we also used) and crossing them to iNOS-tdTomato-Cre animals demonstrates almost exclusive expression of iNOS within IBA1^{+} cells at peak-disease while at EAE recovery, Arg1^{+}IBA1^{+} cells dominate[3]. This is different to what is observed in the Greenhalgh manuscript mentioned by the reviewer (we have now cited it), which using LysM^{-}EGFP animals demonstrates almost exclusive Arg1 co-localization with Iba1^{+}/EGFP^{-} cells (which they designate Mdcs)[4]. In our manuscript, using various flow cytometry panels coupled to fate mapping, we provide evidence that the majority cells at peak disease are Arg1^{+}iNOS^{-}. Using mouse models deficient in Arg1, we then suggest that likely their influx instigates arginine catabolism and detrimental effects in neuroinflammation.

Although using CCR2 and CX3CR1 to define myeloid cells populations has limitations, as mentioned above, by coupling these markers to fate mapping using *R26*^{tdTomato} *Ccr2*-creER^{T2} *Arg1*-eYFP animals and flow panels that employ P2RY12, CD44, F4/80, we are more precisely able to delineate that indeed the majority of Arg1^{+}iNOS^{-} cells at peak disease are infiltrating Mdcs. Thanks to the nice suggestion of the reviewer below

in minor point 16, we have now employed *R26*^{tdTomato} Ms4a3-cre animals, together with these flow cytometry markers. Herein, we identify a minor population of CD11b^{+}CD45^{+}Ms4a3-tdTomato^{-}CX3CR1^{+}P2RY12^{-} cells in spinal cords of animals at peak-disease that are mostly Arg1^{+}iNOS^{-} (approx. 18%). Given their marker repertoire, we have designed these cells as CAMs/cDC2s (Fig. 2i, m, Extended Data Fig. 4f-i). However, as these cells represent such a small amount in the inflamed CNS (please see population 2 in Extended Data Fig. 4a and 4f), clearly infiltrating Mdcs are the most prominent Arg1 source.

1) The authors heavily rely on CX3CR1 and CCR2 expression when defining CNS myeloid populations. The authors should emphasize more the limitations of the models (e.g. when using Cx3cr1cre mice).

Response: Please see our response above regarding use of CX3CR1 and CCR2. We agree it is important to emphasize the limitations of the models, particularly the *Arg1*$^{fl/fl}$ *Cx3cr1*-cre animals. We have now included this in the discussion on **pages 24-25, lines 577-584** and write, "Nonetheless, we acknowledge the limitations of our experimental setup utilizing *Arg1*$^{fl/fl}$ *Cx3cr1*-cre animals which aside from broad targeting activity also include confounding effects of CX3CR1 haploinsufficency, that in turn can influence myeloid cell activation and worsen EAE severity. Although we cannot conclusively rule this out, *Arg1*$^{fl/fl}$ *Cx3cr1*-cre animals exhibited better clinical outcome and Arg1 expressing CAMs represented a minor population in the inflamed CNS, suggesting their phenotype is unlikely due to off target cre-activity or partial CX3CR1 loss"

2) Fig. 2i: This analysis needs quantification (relative proximity to lesions).

Response: We have now included a quantification of Arg1-expressing cells in *R26*^{tdTomato} *Ccr2*-creER^{T2} *Arg1*-eYFP animals (old Fig. 2i, new Fig. 3a, quantification is in new Fig. 3b).

3) MS samples: How do the authors come to the conclusion that Arg1 expression is linked to Mdc infiltration?

Response: We agree that CD68 staining cannot distinguish between Mdcs and inflamed microglia/macrophages in human MS lesions. Thus, we have removed this overstatement from the revised version of the manuscript. Based on the suggestion of reviewer #3, we now employed double immune-labelling of Arg1 with IBA-1, additionally including astrocyte and neuronal makers. This provides corroborating evidence that Arg1 is indeed found in myeloid cells within MS lesions, supporting the CD68 double immuno-fluorescence data (new Fig. 3e)

4) Fig.3a: authors sort CX3CR1+YFP+ and YFP- cells from Arg1tm1Lky animals and see significant upregulation of Mrc1 in YFP+ population. Yet this could very well be due to the contribution of CAMs.

Response: We agree that we cannot exclude a contribution of CAMs in this experimental setup and have now acknowledged this in **lines 310-311**. As mentioned by the reviewer below in minor point 19, we have now included data on the identity of cluster 18 in the publicly available single cell sequencing data-set employed in our study[5]. Although cluster 18 is rich for CAM related genes such as *Mrc1* and *Cd163*[6,7,8,] this cluster does not express Arg1 in this data set (New Extended Data Fig. 7c).

As mentioned in the text (**lines 312-313**), except for *Mrc1* (CD206), we did not see an upregulation in genes associated with M2 responses. This was interesting because it suggested contrary to the well-established role of Arg1 in M2 responses and tissue repair, this population was perhaps doing something at peak-inflammation different to that previously proposed[9,3], possibly via its effects on arginine availability within the CNS. Interestingly, in other organs (e.g. adipose tissue, tumour-associated macrophages), *Mrc1* has been associated with lipid-associated macrophages[10,11].

5) Microglia was defined as CD11b+CCR2negCX3CR1pos, CD45mid, P2RY12pos. Yet, gating strategy in extended Fig.3d shows microglia in CX3CR1neg gate, and gated from CD45mid but without the discrimination based on CD11b.

Response: We are very grateful to the reviewer for noticing this mistake! In old Extended Fig. 3d (new Extended Data Fig. 3c), although we did gate on CD11b, it is indeed puzzling why $CD45^{mid}$ microglia were CX3CR1 negative. We re-examined all of our flow cytometry data and realized there was a problem with the FMO of CX3CR1

(we now show this FMO in Extended Fig. 1c). We have now rectified this mistake and consequently corrected all data. Importantly, this does not change the main message or conclusions of our manuscript.

6) Extended.Fig.4: It is mentioned that activated microglia upregulates CD45. However, it seems essential to consider that P2RY12 is downregulated in EAE microglia (Jordão et al, Science 2109). In Ex.Fig.4c, the expression of Arg1-YFP is shown in CD45high activated microglia. The authors should show what kind of gating strategy was employed for the identification of this population. In Ex.Fig. 4a-b the frequency of moDCs and moMACs appears to be much higher than the frequency of microglia, differing from the quantitative analysis shown in Fig.1. This should be explained.

Response: We agree that the term activated microglia is confusing. Although P2RY12 is downregulated during EAE it is not completely lost[12,6]. We now use the term microglia (defined as $CD45^{mid}CX3CR1^{+}CCR2\text{-}tomato^{-}P2RY12^{+}$) and show P2YR12 downregulation in mice suffering from peak-disease versus health (New Extended Fig. 4c). We agree, the frequency of Mdcs that comprise moDCs and moMACs (population 1) is much higher than population 2 in Extended Fig. 4a compared to the quantitative analysis shown in Fig 1d. We also see a similar effect when looking at these populations in Extended Fig. 3c. This can be explained as data in Extended Fig. 4a comprises fate mapping mice, i.e. $tdTomato^{+}$ Mdcs comprise both $CCR2^{+}$ Mdcs as well as Mdcs that have already downregulated CCR2 versus the setup's employed in Fig. 1 and Extended Fig. 3c.

7) The definition and separation of moMACs and moDCs should be better. There is no gating discrimination in Ex.Fig.4a regarding CD44, although authors call moMACs CD44hi and moDCs CD44+. Usage of other markers (e.g., CD64, MHC-II, CD16/32, MerTK) might be useful for clearer differentiation between these two populations.

Response: Although it is difficult to define moMACs and moDCs in the CNS, CD44 has been previously used[2], with data from the Becher group also suggesting it is a good marker to distinguish infiltrating moDCs from moMACs[1]. In line, we find CD44 expression is higher in $F4/80^{hi}$ moMACs than $F4/80^{-}$ moDCs (Extended Data Fig. 4b).

As mentioned earlier, based on the reviewer's suggestion below in minor point 16, we have now included other markers in the *R26*^{tdTomato} *Ms4a3*-cre experimental setup. This demonstrates that aside from CD44, CD64 and MERTK are useful for discriminating moMACs from moDCs (new Extended Data Fig. 4i).

8) Ex. Fig. 6 and 7: The statement that Slc72ahigh cells express both chains of the CSF2 receptor has to be backed up by proper quantitative analysis.

Response: We have removed this data from the revised version of the manuscript.

9) Extended Fig. 7f: Important controls are missing, e.g. LPS alone, GM-CSF alone.

Response: Old Extended Data Fig. 7f (new Extended Data. Fig. 8d) was an arginine ELISA from supernatants of these conditions, where we did not include these controls. Metabolomics of supernatants of these control conditions indicates that relative to GM-CSF or LPS alone, a combination of the treatments leads to most decreases in extracellular arginine. Please find the data below.

Figure 1

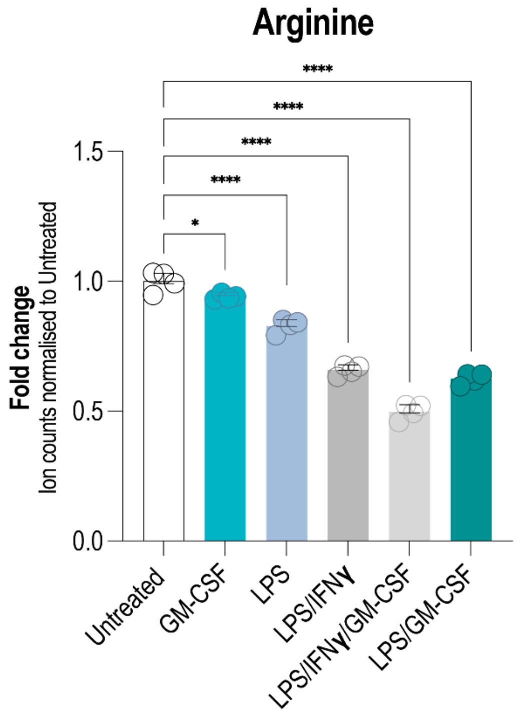

Figure 1. Arginine consumption of treated BMDMs
Differences in extracellular metabolite composition between SN of BMDMs from C57BL/6J animals treated as indicated for 48h. n = 4 samples per condition. Results are mean ± SEM (*P = 0.0259, ****P < 0.0001; one-way ANOVA followed by Sidak's multiple-comparisons post-test).

10) Ex. Fig.7: The importance of GM-CSF for Arg1-mediated arginine catabolism in Mdcs is interesting and should be analyzed in vivo, e.g., by using Ccr2creERT2;Csf2rbfl/fl mice. Furthermore, the authors could utilize the publicly available scSeq dataset from Csf2rb-/- chimeric mice in EAE model (Amorim et al), which already suggests that Arg1+ mononuclear phagocytes found in the CNS of peak EAE mice rely on GM-CSF signaling (Figure 6a in Amorim et al).

Response: Thanks for this nice suggestion! We have now utilized *Csf2rb*$^{fl/fl}$ *Ccr2*-cre^{ERT2} animals, administering tamoxifen at disease onset, as its deletion prior to disease induction on Mdcs renders animals EAE resistant[13]. This new data supports that GM-CSF does indeed partially contribute to Arg1 induction in Mdcs in the context of EAE (new Fig. 4j, Extended Data Fig. 8k). This is in line with findings in other systems such as the tumor microenvironment, where tumour derived GM-CSF promotes myeloid Arg1 expression[14]. Furthermore, BMDMs isolated from *Csf2rb*$^{fl/fl}$

Ccr2-creER^{T2} animals and administered 4-Hydroxytamoxifen (4-OHT) displayed attenuated Arg1^{+} signatures post LPS/GM-CSF relative to controls (new Fig. 4k).

Although the single cell RNA seq data mentioned by the reviewer[1] also supports that Arg1^{+} Mdcs rely on GM-CSF signaling in EAE, here, the investigators performed mixed bone marrow chimeras of mice transplanted with *Csf2rb*$^{+/+}$ and *Csf2rb*$^{-/-}$ bone marrow (1:1), subsequently sorting Mdcs (CD11b^{+}Ly6G^{-}Nk1.1^{-}CX3CR1^{+}CD44^{hi} cells) and performing scRNAseq. This demonstrates that aside from the absence of the Arg1 cluster, several other clusters are missing and that a lack of GM-CSF signaling in *Csf2rb*$^{-/-}$ monocytes leads to the emergence different clusters of "atypical Mdcs" (Fig. 6a/b of *Amorim et al*). Overall, we thought including this scRNAseq data set would unnecessarily complicate our already very extensive manuscript and instead opted to focus on how GM-CSF promotes Arg1 induction at the protein level.

11) Fig. 5b and line 434: This wording here is misleading. Correct: The authors did not see a significant clinical effect of the arginine-free diet, there is nothing like a "strong tendency". In the same line: instead of changing between "ns" and "p=0.0547", either p-values have to be provided for each comparison, or only if below 0.05 (see for example the "strong tendency" in Fig. 5 e). Similar concerns exist the description of Fig. 5j.

Response: We apologize for this overstatement. Although in old Fig. 5b (EAE clinical course in context of arginine sufficient or deficient diet), there was a strong tendency for arginine free diet fed animals to do worse, we agree the p value was insignificant. We increased the n and the data is now significant (New Fig. 6b). Despite this, we acknowledge this effect is modest. We now write on **page 20, lines 485-487** "Consistent with hypothesized beneficial effects of arginine, these animals exhibited mildly worsened clinical outcome". Based on the comments of reviewers #2 and #3, in this experimental setup, at clinical end-point, we have now performed 4-HNE, MDA ELISAs and Luxol blue staining (New Fig. 6c, f). This new data supports the clinical outcome data by demonstrating that arginine free diet is associated with more demyelination and lipid peroxidation, strengthening that L-arginine scarcity has negative effects in neuroinflammation.

Regarding the data in old Fig. 5j, which demonstrated a strong tendency for more CD25$^+$FoxP3$^+$ Tregs in EAE in the context of arginine supplementation, given below (minor point 36) the reviewer recommends to remove the in vitro iTreg data (old Fig. 5k) to the discussion, we have now removed the data from old Fig. 5j from the revised manuscript.

Minor points:

1) The language appears partially inflated (e.g. overuse of terms like "landscape", "rewiring", "orchestrate", and in particular "striking"). The language should be toned down. Terms like "intriguingly" should be avoided.

Response: We have now significantly toned down on the language.

2) It seems important to better define what the authors mean by meningeal macs: leptomeningeal? Dural? Both?

Response: We have now changed the intro to be more broad and do no distinguish between these macrophage types, instead writing "CAMs express a different transcriptional signature to resident microglia" (**lines 87**).

3) Fig 1b: The colors of different experimental groups are in part very similar and not easy to distinguish, especially between peak and recovery groups. The names of the metabolites are not visible. It would be sufficient to indicate values of 5 - 10 metabolites (more relevant to the paper) on the bar plots. Y-axis labeling is also hardly visible.

Response: Thanks, we increased the font of Y-axis labelling so the reader is able to interpret the bar plots, but would prefer to keep the soft color scheme. The main point of the bar plots is to depict the values that were used to generate the density kernel plots above them. Therein, the Z-scores for the individual metabolites are overwhelmingly on the positive side (above 0) for peak-disease, negative for healthy and more balanced or close to zero for recovery. Thus, we prefer to show the overall picture over picking just 5-10 metabolites. If the reader is interested in individual metabolites, more detailed information can be found in Fig. 1e, f or could zoom in on a pdf version of the plot.

4) Fig 1d: Are CX3CR1+ cells also CCR2+? Here, the same labeling of populations as in Ex.Fig.1c,d should be employed. Please add the frequencies of same populations from recovered mice.

Response: Yes, CX3CR1^{+} cells are also CCR2^{+}. We have now gated on the same populations in Fig. 1d as in Extended Fig. 1c.

5) Fig.1e,f: The metabolites that are increased or decreased in both spinal cord and CSF should be somehow indicated (bold letters, arrows, etc.)

Response: We have now highlighted them in red (Fig. 1e, f)**.**

6) Ex.Fig.1d: Could authors show additionally Ly6C expression as a dotplot?

Response: We now have provided the dot-plot in Ext Fig. 1d.

7) Ex.Fig.1e: In T-cell gating, please add FMO for CD3 staining.

Response: We now provided this.

8) 73-75: Macrophages in the dura are also replaced by monocytes (Van Hove, Nat Neuroscience 2019.)

Response: Thanks, we have now cited this paper **(lines 74-76).**

9) - 84: Authors state that Mdcs do not contribute to the microglial pool, however, this is disease specific, e.g., monocytes can engraft in cases of bacterial meningitis (Djukic et al, Brain 2006.).

Response: We are now more specific with regards to this statement and write on **lines 81-84 "**CAMs express a different transcriptional signature to resident microglia and although during neuroinflammation several distinct monocytes, moMACs, and DC populations transiently emerge through the blood-brain barrier, triggering <u>autoimmune</u>

neuroinflammation, these monocyte-derived cells (Mdcs) do not contribute to the microglial pool"

10) - 145: There is no Ly6C expression shown in Ex.Fig.1c, Ex.Fig.1d should be shown as dotplots as well.

Response: In this panel no Ly6C was included, however as mentioned above, we have included it for Ext Fig. 1d.

11) l275: "Orchestrated" should be replaced by "associated with"

Response: We have removed the word orchestrated from the manuscript

12) Fig 2a: Plotting spinal cord and CSF FDR of MSEA together on the same plot would make data clearer.

Response: We kindly disagree. We measured 155 and 111 metabolites in the spinal cord and CSF respectively (Fig. 1a). As we always separate these two tissues, we think combining the MSEA would not make the data clearer.

13) Fig 2b, c: Why is there no data on recovered animals?

Response: We classified arginine as a disease-linked metabolite (Fig. 1e) as it was significantly changed between health and peak-disease as well as health and recovery. There is no significant difference in arginine levels between peak-disease and recovery (otherwise we would have classified it as a recovery-linked metabolite).

14) Fig 2e: The scheme of urea cycle should come before Fig 2b.

Response: We think it would be clearer to keep the order as it is. The current data in Fig. 2b, represent the arginine biosynthesis metabolites from the MSEA analysis. Subsequently, we verify arginine levels and zoom in on the arginine pathway.

15) Ex.Fig.3a: Could authors confirm that Arginase2 expression between heathy and peak/recovery group is indeed insignificant?

Response: We agree the statistical comparison employed was wrong and thank the reviewer for alerting us to this point. We now use a two-way ANOVA which indeed shows *Arg2* is not significantly changed between EAE phases in relation to *Arg1* and *iNos*. The conclusion related to this figure has not changed and we still write in **line 202** “Robust Arg1 versus Arg2 induction is consistent with previous studies”.

16) Ex.Fig3e: It should be elaborated on the chosen timeline, e.g. on the reason for a break in tamoxifen provision before EAE induction. The labeling of CCR2+ progeny seems to be transient, e.g., blood monocytes drop their Tomato expression to 10.6% after a week of discontinued tamoxifen. Using reporter mice that allow for a long-term monocyte labeling would resolve possible off-target effects of long-term tamoxifen usage. Considering it was previously shown that all moMACs and moDCs stem from GMP (Amorim et al), Ms4a3cre;R26TdTomato mice would be appropriate alternative.

Response: We did the break to allow the mice to rest from the tamoxifen diet (reducing long-term off target effects) and additionally to demonstrate that labelling efficiency is indeed transient. Thanks for the nice suggestion to use $R26^{\mathrm{tdTomato}}$ Ms4a3-cre animals to alleviate long term tamoxifen use. Data from these animals confirms that $CD11b^{+}CD45^{+}Ms4a3\text{-}tdTomato^{+}CX3CR1^{+}$ Mdcs were mainly $Arg1^{+}INOS^{-}$ at peak-disease with similar percentages obtained to $R26^{\mathrm{tdTomato}}$ *Ccr2*-$creER^{T2}$ animals (New Fig. 2l, New Extended Data Fig. 4f,g, Extended Data Fig. 3g), hence demonstrating comparable labelling efficiency. As mentioned earlier, data from these animals also ratifies the fidelity of the moMAC and moDC populations (New Extended Data Fig. 4i).

17) The authors should comment on the discrepancies in percentages between Ex.Fig.3c and g?

Response: As mentioned above in our response to major comment point 5, there was a mistake with the CX3CR1 gating. As we rectified this, we do not observe $CCR2^{+}CX3CR1^{-}$ cells that caused the mentioned discrepancies. Once again, we are very grateful to the reviewer for noticing this mistake!

- Fig 2i: Please use colorblind-friendly colors, e.g., green+magenta? The addition of the single channel pictures is needed. IF staining using IBA-1 as a microglial marker to confirm less Arg1 expression in comparison to Mdcs is advised.

Response: We have now provided color blind friendly colors for the data in old Fig 2i (new Fig. 3a). Single images have been included. Given we provide flow cytometry data demonstrating that murine microglia at peak-EAE barely express Arg1, we believe the suggested IBA-1 stain may not be necessary.

Correct this

18) 304-306: This is an overstatement, Fig 3e doesn't confirm Arg1+ Mdcs differentiate into Arg1+ moMACs.

Response: We agree and have removed this overstatement. The text now reads "This revealed Arg1 was predominant and specifically expressed in a cluster (cluster 2) rich for typical monocyte and macrophage markers including *Ly6i*, *Ly6c2* and *Lyz2*" (**lines 331-333**).

Change

19) Fig 3f: What are the identifying genes of cluster 18? If it represents monocytes/macrophages, shouldn't it be Ly6c2 positive as well? Could you show representative tSNE for other markers used throughout the paper, e.g. Ccr2, Cx3cr1, Adgre1?

Response: As mentioned in major point 4, we have now added dot plots for the identifying genes of the myeloid cell clusters from the Wheeler data set (GSE130119). This revealed cluster 18, is rich for typical CAM markers including *Mrc1* and *Cd163* (new Extended Data Fig. 7c). For the reviewer's reference, please find below tSNE plots for *Ccr2*, *Cx3cr1*, *Adgre1* and *Cd44*.

Figure 2

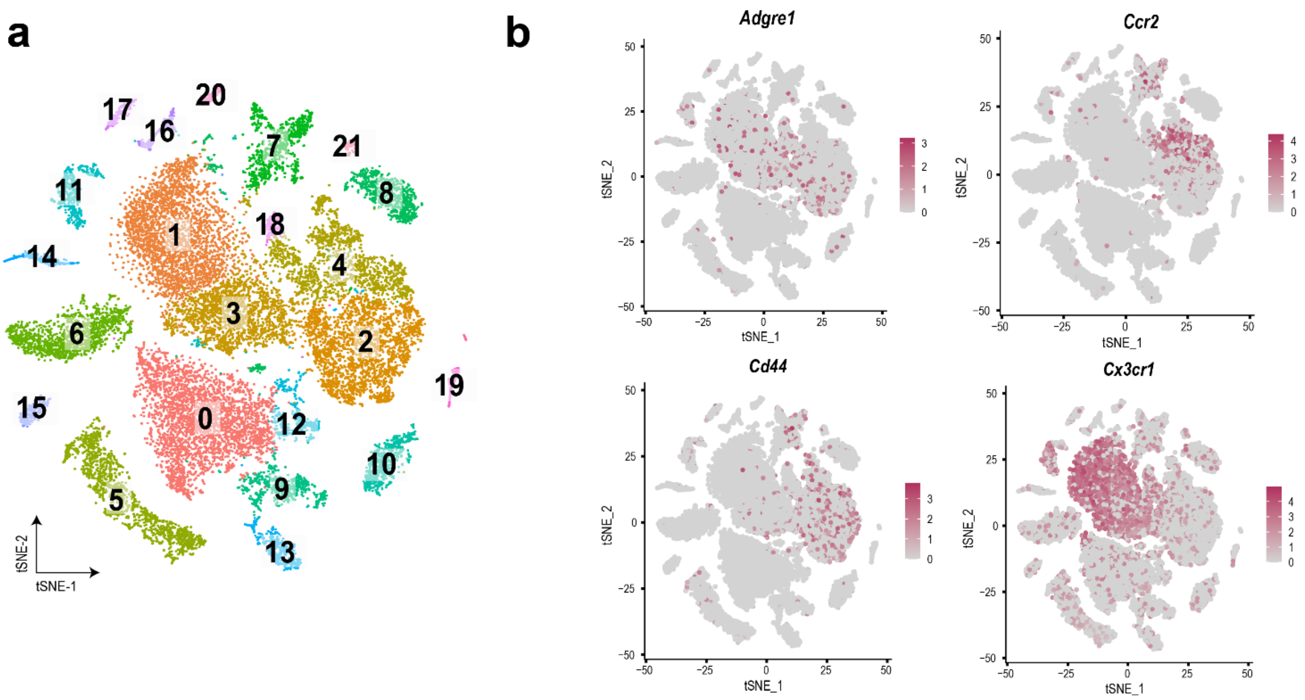

Figure 2. t-SNE plots of disease associated populations in EAE
a) Unsupervised clustering t-distributed stochastic neighbor embedding (t-SNE) plot showing spinal cord cellular composition during EAE among all CNS cells subjected to scRNA-seq (24,275 cells) from data set GSE130119. Coordinates, metadata and annotations were kept the same as the original publication. 0, MG/MΦ; 1, MG/MΦ; 2, moMACs; 3, MG/MΦ; 4, Dendritic cells; 5, Ependymal cells; 6, Oligodendrocytes; 7, T cells; 8, Astrocytes; 9, Pericytes; 10, Astrocytes; 11, Neutrophils; 12, Endothelial cells; 13, Sm. Muscle cells; 14, Astrocytes; 15, Endothelial cells; 16, Neurons; 17, Epithelial cells; 18, Monocytes/MΦ; 19, Epithelial cells; 20, Stromal cells; 21, Neurons. MG, microglia; MΦ, macrophages. b) t-SNE plot showing *Adgre1*, *Ccr2, Cd44* and *Cx3cr1* expression in clusters shown in (a).

20) 330: Csf2ra and Csf2rb seem to be expressed heterogeneously across multiple clusters and not just on moMACs, could authors comment on it? More objective approach would be using violin plots in order to compare Csf2ra and Csf2rb expression across populations.

Response: In the revised version, we have removed this data (old Extended Data Fig. 6d).

21) 335: The overusage of abbreviations is confusing and should be avoided (LG, LI etc.), i.e. replaced by the established abbreviations of the stimulants.

Response: We have removed these abbreviations and now write out established ones.

22) 372: "strong tendencies" do not exist in the scientific context (s. a.).

Response: As mentioned in major point 11, we have removed or replaced insignificant data and as strong tendencies are an overstatement we do not use this term anymore.

23) Fig. 5: "Undesireable" should be omitted from the title, it is not s scientific term. In the same direction, the title of fig. 4 seems inappropriate ("detrimentally rewiring…)

Response: We have removed the term "undesirable" from the legend of old Fig. 5 (new Fig. 6) and replaced it with "negative". Similarly, we have removed the term "detrimentally rewiring" from the legend of old Fig 4 (new Fig. 5). Based on the reviewer's suggestions, we have toned down on the language.

24) Ex.Fig. 7a: Based on previously published data, one would expect higher GM-CSF levels during peak disease. On the presented heatmap the levels do not seem to be significantly increased. The authors should comment and provide an additional bar plot.

Response: Old Extended Data Fig 7a, has now been shifted to Fig. 2f. We have now included a bar plot showing GM-CSF levels are indeed elevated at peak-disease versus health and recovery (new Fig. 2g).

25) Ex.Fig 7d: Is there a significant difference between % of Arg+iNOS+ cells in LIG vs LG groups? Why is LI group used as a baseline to compare other conditions to?

Response: Based on the reviewer's comments below to include untreated controls as well as IFN-γ, GM-CSF, LPS alone, we have significantly modified old Extended Data Fig. 7d to include these other conditions (new Extended Data Fig. Fig 8b). There is a significant difference in % $Arg1^{+}iNOS^{+}$ cells between LPS/IFN-γ/GM-CSF versus LPS/GM-CSF at 24h, but not at 48h (please see statistical matrix in Supplementary Table 8). However, the $Arg^{+i}NOS^{-}$ cells are significant at both time points. We now

refer to this data on **page 16, lines 364-368** “While LPS/ IFN-γ or IL-4/IL-13 led to either iNOS or Arg1 production, and GM-CSF or IFN-γ alone had no impact, simultaneous LPS/IFN-γ/GM-CSF stimulation resulted in a shift from $iNOS^+Arg1^-$ to $iNOS^+Arg1^+$ expression. This effect was prominent in IFN-ɣ absence with LPS/GM-CSF treated cells exhibiting a clear temporal shift from an $iNOS^+Arg1^+$ to an $iNOS^-Arg1^+$ signature (Extended Data Fig. 8b, c)”.

26) - Ex.Fig.7f: Please show arginine levels in IL-4/IL-13 condition?

Response: We have now included the arginine levels for the IL4/IL-13 condition (new Extended Fig. 8d).

27) - Ex.Fig.7g: Please show CellRox for other conditions, e.g., LPS alone.

Response: We have now included the LPS alone condition (new Extended Fig. 8f).

28) - Ex.Fig.7c,d: The unstimulated condition should be provided as in f.

Response: We have now included the unstimulated condition (new Extended Fig. 8b,c).

29) - 345-348: The conclusion lacks the GM-CSF-related findings.

Response: As we have now used *Csf2rb*$^{fl/fl}$ *Ccr2*-creER^{T2} animals to strengthen the role of GM-CSF to Arg1 induction, the text has significantly changed and in the revised version this conclusion is stated after these new findings.

30) - Ex.Fig 7h: Arg1 expression should be analyzed in CX3CR1+ cells.

Response: We have now gated for Arg1 expression in $CX3CR1^+$ cells (new Extended Data Fig. 8g, h)

31) - 367-368: Strong statement based on in vitro data. In vivo confirmation would be necessary (check major points).

Response: As mentioned above, we have now included data on *Csf2rb*$^{fl/fl}$ *Ccr2*-creER^{T2} animals, which strengthens the role of GM-CSF to Arg1 induction.

32) Ex.Fig.8a: Arg1 depletion is not complete in Cx3cr1Cre/+;Arg1fl/fl animals. This should be commented on.

Response: We acknowledge that Arg1 depletion is not complete. Although we do not exactly know why this is, a closer examination of the literature utilizing *Cx3cr1*^{cre}*R26-YFP* animals demonstrates roughly 40% of Ly6C^{+} blood monocytes are targeted by the Cre[15], which may explain the moderate two-fold reduction in Arg1 expressing Mdcs in this experimental setup (Extended Data Fig. 10a, b). Despite this, we clearly observe biological effects in this experimental system.

To strengthen the role of Arg1 in Mdcs to worsened EAE outcome, and based on the reviewer's excellent suggestion to include *R26*^{tdTomato} Ms4a3-cre animals (which demonstrates Arg1 expressing Mdcs have a Ms4a3 lineage), we generated *Arg1*$^{fl/fl}$*Ms4a3*$^{cre/+}$ animals to provide an alternative experimental approach to study Arg1 function specifically in Mdcs, excluding potential limitations of the CX3CR1 Cre. Unfortunately, in this experimental setup we could not observe any Arg1 deletion within Mdcs and consequently no impact on EAE. Further, in a sterile peritonitis model although we could observe robust tdTomato and Arg1 expression within infiltrating Mdcs, we also did not observe a deletion in this experimental setup. This is likely due to limited and transient Cre availability possibly related to chromatin accessibility at the GMP stage in the bone marrow. For the reviewer's reference we have included the data below.

Figure 3

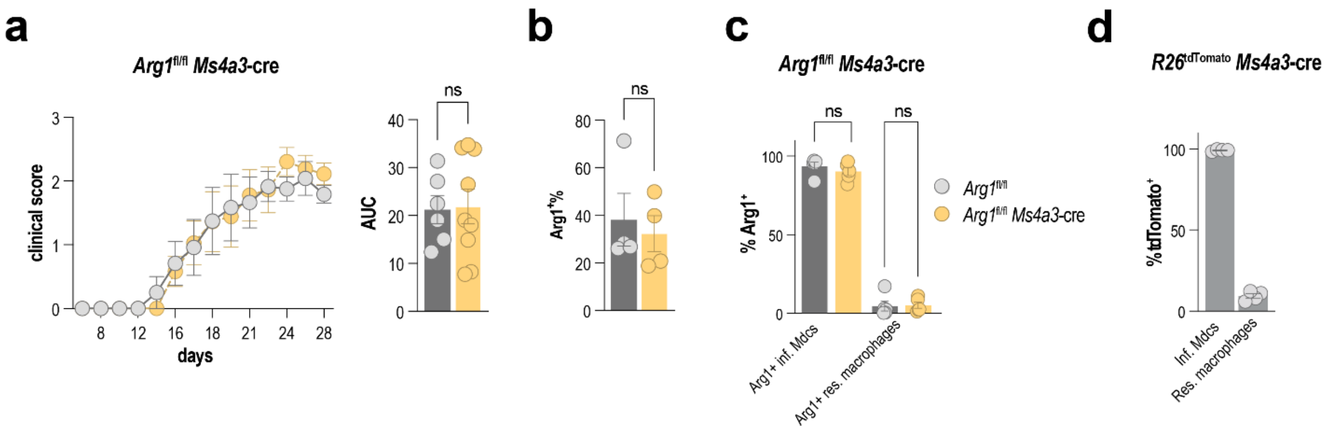

Figure 3. Clinical disease course of *Arg1*$^{fl/fl}$ *Ms4a3*-cre animals and deletion efficiency during EAE and sterile peritonitis

a) Clinical course of *Arg1*$^{fl/fl}$ and *Arg1*$^{fl/fl}$ *Ms4a3*-cre animals subjected to EAE. n = 6 (*Arg1*$^{fl/fl}$), 9 (*Arg1*$^{fl/fl}$ *Ms4a3*-cre) animals, pooled from two independent experiments. Results are mean ± SEM. Statistical analysis was performed using unpaired t-test or for the clinical course P values were determined using area under the curve (AUC) followed by t-test. b) Percentages of Arg1+ cells within CX3CR1$^+$CCR2$^+$ cells (Mdcs) from spinal cords of *Arg1*$^{fl/fl}$ and *Arg1*$^{fl/fl}$ *Ms4a3*-cre animals at peak EAE. n = 4 (*Arg1*$^{fl/fl}$), n = 4 (*Arg1*$^{fl/fl}$ *Ms4a3*-cre) animals. Results are mean ± SEM (unpaired t-test). Cells pre-gated on single, viable, Ly6G$^-$CD3$^-$CD45^{hi}CX3CR1$^+$CCR2$^+$. c) Percentages of Arg1$^+$ cells within infiltrating Mdcs (CCR2$^+$Ly6C$^+$) and resident peritoneal macrophages (CCR2$^-$Ly6C$^-$) from the peritoneal lavage of *Arg1*$^{fl/fl}$ and *Arg1*$^{fl/fl}$ *Ms4a3*-cre animals 48 hours after induction of a sterile peritonitis with thioglycolate. n = 5 (*Arg1*$^{fl/fl}$), n = 5 (*Arg1*$^{fl/fl}$ *Ms4a3*-cre) animals. Results are mean ± SEM (unpaired t-test). Cells pre-gated on single, viable, CD45$^+$Ly6G$^-$CD3$^-$CD11b$^+$F4/80$^+$. d) Percentages of tdTomato+ cells within infiltrating Mdcs (CCR2$^+$Ly6C$^+$) and resident peritoneal macrophages (CCR2$^-$Ly6C$^-$) from the peritoneal lavage of *R26*^{tdTomato} *Ms4a3*-cre animals 48 hours after induction of a sterile peritonitis with thioglycolate. n = 4 animals. Results are mean ± SEM (unpaired t-test). Cells pre-gated on single, viable, CD45$^+$Ly6G$^-$CD3$^-$CD11b$^+$F4/80$^+$.

33) - Ex.Fig.8a,d: There seems to be a significant difference between Arg1 expression from Argfl/fl mice in two different models: Cx3cr1cre and CCR2creERT2. This should be explained. Also, it seems that Arg1fl/flCcr2creERT2 mice have an overall lower clinical score than Arg1fl/flCx3cr1cre/+ mice, and the littermate controls seem to be significantly different as well (Ex.Fig.8f and Fig.4a). This should be discussed.

Response: The differences in Arg1 expression as well as clinical score in old Extended Data Fig 8a,d (new Extended Data Fig. 10a, d) might be explained by different experimental setups. One setup uses tamoxifen, while the other doesn't. We now refer to this in the discussion on **page 24, lines 571-573** and write, "Noteworthy,

tamoxifen itself ameliorates EAE development[16], which could explain differences in clinical score between *Arg1*$^{fl/fl}$ *Cx3cr1*-cre and *Arg1*$^{fl/fl}$ *Ccr2*-creER^{T2} animals”.

34) Ex.Fig.8: The plots for IL-10 and TGFβ in both genotypes should be shown.

Response: This is now included in new Extended Data 10i.

35) 444-445: Although it is mentioned here, the Mdcs frequency is not shown. In Fig5e: What is the percentage of CX3CR1+CCR2+ cells in control vs. arginine free-diet?

Response: We apologize for this mistake and have now included the data in new Fig 6i.

36) 467 – 473: This paragraph should be worded more modestly and inserted into the discussion section.

Response: As mentioned above in major point 11 we have 1) Removed old Fig. 5j, which demonstrated a strong albeit insignificant tendency for more CD25^{+}FoxP3^{+} Tregs in EAE in the context of arginine supplementation. 2) Removed data demonstrating that a 6-fold molar excess of L-arginine, moderately increased TGF-β mediated iTreg generation (old Fig. 5k). Based on the reviewer’s recommendation, to word this more modestly and include potential effects of arginine on Tregs in the discussion, we now write on **page 25, lines 593-599** “Highlighting the crucial role for arginine catabolism downstream of Arg1 in promoting neuroinflammation, we disclose that EAE suffering animals fed arginine-free diet display lower IL-10, TGF-β and Treg levels. Although arginine is well described to influence survival and proliferation of T cells[17,18], in neuroinflammation, arginine exerted unremarkable influences on T cell frequencies. Instead, arginine scarcity dampens Mdc-driven IL-10 and TGF-β production, setting the stage for reduced Treg abundance, thereby conferring worsened EAE outcome[19], <u>although we cannot rule out direct effects of arginine on Treg abundance</u>[20]”

37) 575-576: Was CD11b used for gating on myeloid population?

Response: In Fig. 1d, we did not use CD11b for gating on myeloid populations, however we exclude T cells, neutrophils, subsequently gating infiltrating myeloid cell populations on $CD45^{hi}$ and microglia on $CD45^{mid}$.

Statistics:

- Overall authors use appropriate statistics. In Ex. Fig. 3c and g, error bars should be added to the bar plots. In certain figure legends the numbers of experimental repetitions are missing.

Response: We thank the reviewer for stating that we use appropriate statistics. Bar plots for data comprising old Extended Data Fig 3c (new Extended Data Fig. 3d) as well as Extended Data Fig. 3g now includes errors bars. Legends have been updated to indicate repetitions.

References:

- Amorim et al. appears twice (#9 and #36)

- In lines 151-154, authors claim that quinolinic acid and α-aminoadipic acid are produced by microglia and CAMs. For quinolinic acid, citation of the original data is missing (Heyes et al, Biochem 1996, Espey et al, Neuroreport 1997.). The citation showing CAMs and microglia are the producers of α-aminoadipic acid is missing.

-489-490: Citation is missing.

- A previously published report showed a decrease in arginine in rat CSF in EAE, which should be cited (Noga et al, Metabolomics 2012).

Response: The citations have been added and duplications in the Amorim reference fixed.

Spelling and editing:

- 78-79: Where they can differentiate into dendritic cells (moDCs) or macrophages (moMACs), with CCR2 being important for…

- 148: Having established that our experimental…

- 153: …lipid peroxidation, while oligodendrocyte and neuronal death…

-154: This, along…

- 158: …energy metabolism imbalances may be compensated…

- 314: demonstrated that, versus…

- 342: Comma missing after "notably"

- There are inconsistencies in how mice lines are written throughout the text and in the figures (e.g., Ex.Fig.8e and f).

- 502: If "akin to others" implies that similar Arg1 expression in microglia is reported in other papers, then citation is missing. If this sentence has other meaning, rephrasing is necessary for clarity.

- 534-535 and 543-544 is somewhat repetitive, both mention arginine scarcity affecting IL-10, TGFβ and Treg abundance.

Response: All of these spelling mistakes have been rectified. Further, mouse lines are now consistent throughout the manuscript.

References

1. Amorim, A. *et al.* IFNgamma and GM-CSF control complementary differentiation programs in the monocyte-to-phagocyte transition during neuroinflammation. *Nat Immunol* **23**, 217-228 (2022).

2. Giladi, A. *et al.* Cxcl10(+) monocytes define a pathogenic subset in the central nervous system during autoimmune neuroinflammation. *Nat Immunol* **21**, 525-534 (2020).

3. Locatelli, G. *et al.* Mononuclear phagocytes locally specify and adapt their phenotype in a multiple sclerosis model. *Nat Neurosci* **21**, 1196-1208 (2018).

4. Greenhalgh, A.D. *et al.* Arginase-1 is expressed exclusively by infiltrating myeloid cells in CNS injury and disease. *Brain Behav Immun* **56**, 61-67 (2016).

5. Wheeler, M.A. *et al.* MAFG-driven astrocytes promote CNS inflammation. *Nature* **578**, 593-599 (2020).

6. Jordao, M.J.C. *et al.* Single-cell profiling identifies myeloid cell subsets with distinct fates during neuroinflammation. *Science* **363** (2019).

7. Masuda, T. *et al.* Novel Hexb-based tools for studying microglia in the CNS. *Nat Immunol* **21**, 802-815 (2020).

8. Goldmann, T. *et al.* Origin, fate and dynamics of macrophages at central nervous system interfaces. *Nat Immunol* **17**, 797-805 (2016).

9. Giles, D.A. *et al.* Myeloid cell plasticity in the evolution of central nervous system autoimmunity. *Ann Neurol* **83**, 131-141 (2018).

10. Brunner, J.S. *et al.* The PI3K pathway preserves metabolic health through MARCO-dependent lipid uptake by adipose tissue macrophages. *Nat Metab* **2**, 1427-1442 (2020).

11. Masetti, M. *et al.* Lipid-loaded tumor-associated macrophages sustain tumor growth and invasiveness in prostate cancer. *J Exp Med* **219** (2022).

12. Beaino, W. *et al.* Purinergic receptors P2Y12R and P2X7R: potential targets for PET imaging of microglia phenotypes in multiple sclerosis. *J Neuroinflammation* **14**, 259 (2017).

13. Croxford, A.L. *et al.* The Cytokine GM-CSF Drives the Inflammatory Signature of CCR2+ Monocytes and Licenses Autoimmunity. *Immunity* **43**, 502-514 (2015).

14. Su, X. *et al.* Breast cancer-derived GM-CSF regulates arginase 1 in myeloid cells to promote an immunosuppressive microenvironment. *J Clin Invest* **131** (2021).

15. Yona, S. *et al.* Fate mapping reveals origins and dynamics of monocytes and tissue macrophages under homeostasis. *Immunity* **38**, 79-91 (2013).

16. Bebo, B.F., Jr. *et al.* Treatment with selective estrogen receptor modulators regulates myelin specific T-cells and suppresses experimental autoimmune encephalomyelitis. *Glia* **57**, 777-790 (2009).

17. Geiger, R. *et al.* L-Arginine Modulates T Cell Metabolism and Enhances Survival and Anti-tumor Activity. *Cell* **167**, 829-842 e813 (2016).

18. Rodriguez, P.C., Quiceno, D.G. & Ochoa, A.C. L-arginine availability regulates T-lymphocyte cell-cycle progression. *Blood* **109**, 1568-1573 (2007).

19. Kohm, A.P., Carpentier, P.A., Anger, H.A. & Miller, S.D. Cutting edge: CD4+CD25+ regulatory T cells suppress antigen-specific autoreactive immune responses and central nervous system inflammation during active experimental autoimmune encephalomyelitis. *J Immunol* **169**, 4712-4716 (2002).

20. Shi, H. *et al.* Amino Acids License Kinase mTORC1 Activity and Treg Cell Function via Small G Proteins Rag and Rheb. *Immunity* **51**, 1012-1027 e1017 (2019).

Reviewer #2

(Remarks to the Author)

In this manuscript, Kerndl et al. examine the role of arginine metabolism in the pathophysiology of EAE using several elegant animal models of arginine perturbation and dietary experiments. While the data is somewhat interesting, the lack of strong human data, unusual choice of models, and unclear mechanism without strong supporting evidence give the findings a very correlative quality with an underwhelming mechanistic interpretation. Addressing the comments below will help strengthen this manuscript's observations:

Response: We thank reviewer #2 for stating that we elegantly perturb arginine using different experimental approaches. We kindly disagree our model/experimental approaches are unusual (please see our response to point 3 below) and that we have an unclear mechanism. Using multiple fate mapping approaches, we demonstrate that likely $Arg1^{+}iNOS^{-}$ Mdcs at peak-disease instigate arginine catabolism. Subsequently, using different conditional Arg1 deficient animals and dietary arginine deficiency, we identify arginine scarcity is associated with negative effects on neuroinflammation. In the revised version, using double immunofluorescence, we have further strengthened the human data by providing data that myeloid cells and not astrocytes or neurons are $Arg1^{+}$ in human MS lesions. Thanks to the reviewer's nice suggestion below to further strengthen the mechanisms by which arginine deprivation instigates lipid peroxidation, we now provide data demonstrating lack of extracellular arginine availability negatively impacts Nrf2 and HO-1 expression. Please find below our point by point response to the comments raised.

1) No cellular metabolism measurements are performed on Mdcs/MG of these mice. Using 13C-tracing to definitively show the urea cycle versus creatine/polyamines is crucial to support this work. It is important to show the fate of arginine in macrophages with Arg1 KO.

Response: We think an *in vivo* tracing is simply not feasible nor ethical in our experimental setup as we have no ethical permission to continuously infuse (via a

catheter) $13C_6$ labelled arginine into animals and then induce EAE. Importantly, EAE already has a high severity grade according to the Austrian Ministry of Sciences. Continuous infusion would likely be needed as a single bolus results in lower overall 13C enrichment in target metabolites[1].

We don't think performing cellular metabolic experiments on microglia is useful as our manuscript is about Mdcs. Further we show that Arg1 is minimally expressing on microglia (Fig. 2k-m). We always aim to adhere to the 3Rs of animal experimentation (reduction, refinement and replacement) and thus try to reduce on mouse numbers wherever possible. Metabolomics of *ex vivo* isolated spinal cord Mdcs is not plausible for several reasons. First, flow sorting is known to have dramatic effects on the metabolome[2] due to cellular stress responses, nutrient deprivation, and metabolite exchange during sorting. This alters the metabolome in unknown ways from the in-vivo state. Moreover, our yield of $CCR2^+CX3CR1^+P2RY12^-$ Mdcs is ~150,000 cells per peak-disease mouse, and we need >1 million cells for adequate metabolite coverage. This low-yield is exacerbated by the loss of intracellular metabolites during flow sorting. Thus, poor signal-to-noise and strong metabolic perturbation made metabolomics analysis of these cells not feasible, and further we would have to sacrifice a lot of animals. As mentioned metabolites are also changed during the isolation process versus immediate freezing of spinal cord and CSF, which preserves metabolic integrity[3,4]. Regarding the fate of arginine in *Arg*1$^{fl/fl}$ *Cx3cr1*-cre macrophages versus controls, we already include data demonstrating that post LPS/GM-CSF, which leads to arginine catabolism via Arg1, arginine levels are elevated and this is associated with decreased orthinine and urea (Extended Data Fig. 8e and 8i).

2) Healthy composition in Figure 1d would be useful.

Response: There are very few $CD45^+$ cells in the spinal cord of healthy animals (Extended data Fig 1a, b).

3) Figure 2C, what does recovery look like for arginine? Related, if the authors are trying to make an argument about arginine availability to tissues why does arginine depletion not come up in the initial metabolomics of CSF?

Response: We classified arginine as a disease-linked metabolite (Fig. 1e) as it was significantly changed between health and peak-disease as well as health and recovery. There is no significant difference in arginine levels between peak-disease and recovery (otherwise we would have classified it as a recovery-linked metabolite). Further, arginine is less in the CSF (Fig. 2b, Extended Data Fig. 2d). It just didn't meet our statistical significance testing for classification as a homeostasis, disease or recovery metabolite.

4) Urea measurements are missing from this work; as the end product of the urea cycle, this data is needed.

Response: Analysis of urea requires a separate procedure for analysis, including sample collection to prevent exchange with media components, extraction, and chemical derivatization to increase molecular weight and improve chromatography. Alternatively, commercial kits are available but require fresh samples and are fully consumptive of the material. Moreover, rapid exchange of urea between the cell and the extracellular environment (both in vivo and during processing) further complicates this analysis. In our case, given the material limitation of our model, we elected to perform polar metabolomics and polyamine analysis, which are incompatible with urea. While we agree with the reviewer that it would be nice to have urea measurements, given the polyamines are downstream of Arg1 (and we have now measured these), we hope the reviewer will agree with us that the lack of urea measurements in spinal cord and CSF does not affect the overall impact of our paper.

Further, we have examined urea in the EAE-conditioned media (EAE-CM) experimental setup. Supporting that arginine catabolism is mediated by $Arg1^{+}$ Mdcs at peak-disease, we could demonstrate that analogous to whole spinal cord of peak-EAE suffering animals, versus healthy-conditioned media (healthy-CM), downregulations in arginine are associated with upregulations of orthinine and urea (Extended Data Fig. 9b).

5) What is the status of polyamines, which are also downstream of Arg1 activation?

Response: Thanks for this important suggestion. We have now measured the polyamines in the identical samples to those employed in Fig.1 and 2. This revealed that at peak-disease ornithine, putrescine and spermidine were elevated in spinal cord and putrescine was elevated in CSF (Fig. 2j, Supplementary Table 6).

6) Regarding "Together, these observations suggest that at peak disease, subsets of Arg1+ Mdcs within demyelinating lesions deprive the local microenvironment of arginine," you need to show that arginine levels are maintained with Arg1 KO in the CSF.

Response: The sentence the reviewer is referring to has been shifted in the revised version. This conclusion comes now after data demonstrating that extracellular arginine catabolism via recombinant arginase-1 (recArg1), leads to more oxidative stress within LPS/GM-CSF treated macrophages (Extended Data Fig. 8m). Based on the reviewer's suggestions to provide more mechanism in point 9 below, we demonstrate this is associated with decreases in Nrf2 and HO-1 and more lipid peroxidation (new Fig. 4n-p). Regarding demonstrating arginine levels are elevated in the CSF of *Arg*1$^{fl/fl}$ *Cx3cr1*-cre animals, we did provide this in old Extended Data Fig. 8c (new extended Data Fig. 10c).

7) You have Tregs in Figure 4 but not in Figure 1. What do Tregs look like at recovery?

Response: In Fig. 1, we only examined myeloid cell and basic T-cell subsets. We have now examined Tregs at recovery and observed they are elevated. Please find the data below.

Figure 1

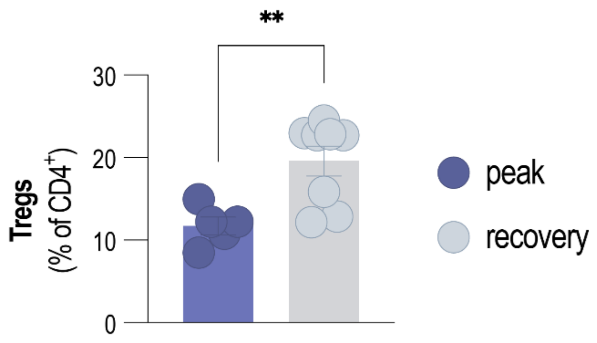

Figure 1. Treg frequency at EAE phases
Percentages of Tregs (FoxP3$^+$ CD25$^+$) at peak-disease and recovery. n = 5 (peak,) and n = 8 (recovery) animals. Results are mean ± SEM (**P = 0.0085; unpaired t-test). Cells were gated on viable CD45$^+$ CD3$^+$ CD4$^+$.

8) The MOG-EAE model is supposed to be acute, not relapsing-remitting. Why use this model to study the "recovery" phase? This is a significant limitation of the work.

Response: We strongly disagree that is a significant limitation and think the reviewer may not fully understand our reasoning for using the term "recovery". The MOG-EAE model in *C57BL/6* animals is a very well established neuroinflammation model, characterized by mono-phasic EAE with an onset phase, acute peak phase, followed by a slow and partial symptom recovery over the next 10-20 days[5]. However, animals never fully recover, still maintaining some disease activity. In our manuscript, we define peak disease as animals having a clinical score of 3.5-3.75. Recovered animals are those whose clinical score is 1.5-1.75 (with a previous score ≥ 3).

The recovery terminology we have used has been used by others in the field, e.g. please see Figure 1 of Korn T et al., *Nature Medicine* (2007) or Fig. 1 of Rothammer V et al., *Nature* (2018) and others[6,7,8]. Alternatively, some call this phase "remission"[9]. Indeed, "recovery" or "remission" are often used interchangeably.

Immunization with myelin proteolipid protein peptide of the SJL/J mouse leads to a relapse-remitting disease course[10,11]). This model does not exhibit a monophasic clinical score and is characterized by repeated phases of "remission" and "relapse". We chose to use the term recovery and not remission to avoid confusion with relapsing-remitting mouse models of MS.

9) Lipid peroxidation probes are insufficient for measurement in Mdcs, especially as a mechanistic insight. How is arginine perturbing lipid peroxidation, this is essential to the mechanism of this study, and provide something beyond correlative measurements.

Response: Despite C-11 BODIPY being is very well-established method for assessing lipid peroxidation within cells[12,13,14], according to the reviewer's suggestion, we have now examined lipid peroxidation levels using 4-hydroxynonenal (4-HNE) and malondialdehyde (MDA) ELISAs in the arginine sufficient/deficient diet experimental setup. This new data demonstrates that elevated lipid peroxidation within Mdcs during arginine scarcity is accompanied by enhanced 4-HNE and MDA spinal cord adducts (new Fig. 6f).

As mentioned in point 6 above, we now provide evidence that arginine is perturbing lipid peroxidation via Nrf2 and HO-1. Extracellular arginine catabolism via recombinant arginase-1 (recArg1), leads to more oxidative stress, lipid peroxidation and less Nrf2 and HO-1 within macrophages in the context of LPS/GM-CSF (new Fig. 4n-p). Further, HO-1 levels are lower in Mdcs in the arginine free diet experimental setup (new Fig. 6g).

We have amended the results section accordingly and write on **page 18, lines 411-423,** "As L-arginine levels in supernatants were only decreased by one third post LPS/GM-CSF-induced arginase catabolism (Extended Data Fig. 8d), we next depleted extracellular arginine using recombinant arginase 1 (recArg1), subsequently examining oxidative stress. Extracellular arginine was robustly depleted and LPS/GM-CSF mediated oxidative stress further elevated (Extended Data. Fig. 8l,m). To understand the mechanisms therein, we measured several anti-oxidant genes including those associated with the glutathione pathway (*Gss, Gsr*), heme oxygenase 1 (HO-1, encoded by *Hmox1*) and the well-established central anti-oxidant regular Nrf2 (encoded by *Ne2l2*). Extracellular arginine depletion in the context of LPS/GM-CSF led to significantly reduced *Ne2l2* and *Hmox1*, with attenuated HO-1 levels verified using flow cytometry and was associated with elevated lipid peroxidation (Fig. 4n-p, Extended Data Fig. 8n). Together, these observations suggest at peak-disease lipid-laden $Arg1^+$ Mdcs within demyelinating lesions deprive the local microenvironment of

arginine, thereby instigating oxidative stress and lipid peroxidation." Further, we now write on **pages 20-21, lines 488-492** "Supporting the premise that extracellular arginine scarcity contributes to oxidative damage and Mdc dysfunction, Mdcs of mice fed an arginine-free diet exhibited heightened lipid peroxidation (Fig. 6e). Lipid peroxidation within Mdcs was accompanied with enhanced 4-hydroxynonenal (4-HNE) and malondialdehyde (MDA) spinal cord adducts and simultaneously associated with decreased HO-1 within Mdcs (Fig, 6f, g)".

We have now discussed these important data in the context of the literature and write on **page 23-24, lines 541-550** "Arg1-deficient Mdcs of *Arg1*$^{fl/fl}$ *Cx3cr1*-cre animals at peak-disease exhibited lower lipid load and peroxidation, while conversely arginine scarcity augmented macrophage lipid load, peroxidation and simultaneously decreased Nrf2 and HO-1 expression. Macrophages in active MS lesions and oligodendrocytes at lesion edges stain positive for nuclear Nrf2. Similarly, CD68$^+$HO-1$^+$ myeloid cells are elevated in murine EAE lesions. This may reflect a cellular defense mechanism against the profound oxidative damage and mitochondrial injury characteristic of MS and EAE lesions, which gives rise to a state of hypoxia-associated with energy deficiency. Accordingly, Nrf2 or HO-1 deficient animals exhibit worsened EAE severity"

10) Please show flow gates for IL10 and TGFB staining in Figure 4.

Response: This is now included in new Extended Data Fig. 10i.

11) Panel 5G needs quantification.

Response: We have now quantified the oil red staining in old Fig 5g (new Fig. 6k). Old Fig. 5g contained the rec-Arg1 condition and as quantification demonstrated no differences in this experimental setup, we removed the data from the revised version of the manuscript. Thus, the quantification consists of the untreated, EAE-CM and EAE-CM/L-arg conditions (new Fig. 6l).

12) The differences in Panel 5K are marginal and not convincing.

Response: We agree that the differences depicted in the data in old Fig 5K, demonstrating that a 6-fold molar excess of L-arginine, moderately increased TGF-β mediated iTreg generation was marginal. Based on this reviewer's comment as well as that of reviewer #1 who suggested to relegate the Treg data to the discussion, we have removed this data from the revised manuscript and word it more modestly in the discussion, where we now write on **page 25, lines 593-599** "Highlighting the crucial role for arginine catabolism downstream of Arg1 in promoting neuroinflammation, we disclose that EAE suffering animals fed arginine-free diet display lower IL-10, TGF-β and Treg levels. Although arginine is well described to influence survival and proliferation of T cells, in neuroinflammation, arginine exerted unremarkable influences on T cell frequencies. Instead, arginine scarcity dampens Mdc-driven IL-10 and TGF-β production, setting the stage for reduced Treg abundance, thereby conferring worsened EAE outcome, although we cannot rule out direct effects of arginine on Treg abundance"

13) In Figure 5, the change in clinical score is non-significant, and even if it was, its biological relevance is doubtful.

Response: We apologize for this overstatement and this point was also raised by reviewer #1. Although in old Fig. 5b (EAE clinical course in context of arginine sufficient or deficient diet), there was a strong tendency for arginine free diet fed animals to do worse, we agree the p value was insignificant. We increased the n and the data is now significant (New Fig. 6b). Despite this, we acknowledge this effect is modest. We now write on **page 20, lines 485-487** "Consistent with hypothesized beneficial effects of arginine, these animals exhibited mildly worsened clinical outcome". As suggested by the reviewer above in point 9, we have provided evidence that arginine free diet is associated with enhanced lipid peroxidation within the spinal cord (4-HNE, MDA). Further, we observe more demyelination in this experimental setup (New Fig. 6c, f). These data support the clinical outcome data, strengthening that L-arginine scarcity has negative effects in neuroinflammation.

14) Does arginine free chow attenuate the clinical benefits of the Arg1 conditional knockout mice? This would strongly support the hypothesis

Response: As highlighted in Fig. 5c, many of the DLMs found in Fig. 1e, exhibited a trajectory reversal in EAE suffering *Arg1*$^{fl/fl}$*Cx3cr1*$^{cre/+}$ animals. Although some of the effects at peak EAE might be reliant on extracellular arginine, given that there are so many players important for a clinical outcome read-out, we think this experimental setup will likely not yield a meaningful outcome. Further, as we always try to reduce on mouse numbers wherever possible, we hope the reviewer agrees that this experiment (which will require a very large number of animals for all conditions -both genotypes +/- arginine sufficient or deficient diet) is likely not warranted.

15) Figures 4F and 5I are both at peak disease, yet the percentages are not even close to one another. What does your Foxp3 staining look like? Are you using the FJK16S clone?

Response: Old Fig 4f (new Fig. 5f) and old Fig. 5l (new Fig. 6o), depict $CD25^{+}FoxP3^{+}$ T-reg percentages within the $CD4^{+}$ T cell pool in the *Arg1*$^{fl/fl}$ *Cx3cr1*-cre versus the arginine free diet experimental setup. These differences could possibly be explained by experimental setups i.e. control arginine sufficient diet (Fig. 5i) versus normal chow diet (Fig. 5f). As indicated in the methods (**line 1296**), we use clone 3G3, Tonbo. Gating strategy and specificity with isotype control is indicated in Extended Data Fig. 10h.

16) The work surrounding the Treg data is underdeveloped without any mechanistic insight.

Response: Originally we provided alternative hypothesis for why Tregs are changed via extracellular arginine availability during EAE suggesting this could either be due to indirect effects (mediated via Mdc produced IL-10 and TGF-β) or due to direct effects of arginine (e.g. as suggested by the in vitro iTreg assay or arginine supplementation in the context of EAE). Given the comments of the reviewer on the modest effect in the iTreg assay (point 12) and that another reviewer suggested to relegate the Treg related aspect to the discussion, we have now removed the data on Tregs in the context of arginine supplementation (old Fig. 5j) as well as the iTreg assay (old Fig. 5k).

Indeed, given the pleiotropic beneficial effects of arginine in neuroinflammation, we think it is really difficult in the context of EAE to prove a direct effect of arginine on Tregs. However, we would like to point out arginine has been reported to affect T-cell proliferation and survival[15,16]. Suggesting an importance of context therein, surprisingly, within CNS infiltrating T-cells, although we observe decreased Tregs in the context of arginine free diet, there no differences in T-cell frequencies (new Fig. 6o). As mentioned in point 12 above, we now word these findings more modestly and include them in the discussion.

17) There's a lot of correlative data, without any direct examination of the role of arginine in EAE. The only mechanism proposed is oxidative damage, and that is underdeveloped.

Response: We kindly disagree that that we provide no direct examination of how arginine influences EAE triggered neuroinflammation. In fact, we provide multiple lines of evidence (genetic and dietary models) showing effects of arginine on Mdc anti-inflammation and lipid load/peroxidation. The catabolism of arginine by $Arg1^{+}iNOS^{-}$ cells and the detrimental effects therein provides a new and important view on Arg1, which is well associated with M2 responses and tissue repair. Thanks to the suggestions of the reviewer we have further developed how arginine influences oxidative damage.

References

1. Faubert, B., Tasdogan, A., Morrison, S.J., Mathews, T.P. & DeBerardinis, R.J. Stable isotope tracing to assess tumor metabolism in vivo. *Nat Protoc* **16**, 5123-5145 (2021).

2. Binek, A. *et al.* Flow Cytometry Has a Significant Impact on the Cellular Metabolome. *J Proteome Res* **18**, 169-181 (2019).

3. Llufrio, E.M., Wang, L., Naser, F.J. & Patti, G.J. Sorting cells alters their redox state and cellular metabolome. *Redox Biol* **16**, 381-387 (2018).

4. Rauckhorst, A.J. *et al.* Mouse tissue harvest-induced hypoxia rapidly alters the in vivo metabolome, between-genotype metabolite level differences, and (13)C-tracing enrichments. *Mol Metab* **66**, 101596 (2022).

5. Bittner, S., Afzali, A.M., Wiendl, H. & Meuth, S.G. Myelin oligodendrocyte glycoprotein (MOG35-55) induced experimental autoimmune encephalomyelitis (EAE) in C57BL/6 mice. *J Vis Exp* (2014).

6. Rothhammer, V. *et al.* Microglial control of astrocytes in response to microbial metabolites. *Nature* **557**, 724-728 (2018).

7. Korn, T. *et al.* Myelin-specific regulatory T cells accumulate in the CNS but fail to control autoimmune inflammation. *Nat Med* **13**, 423-431 (2007).

8. McGeachy, M.J., Stephens, L.A. & Anderton, S.M. Natural recovery and protection from autoimmune encephalomyelitis: contribution of CD4+CD25+ regulatory cells within the central nervous system. *J Immunol* **175**, 3025-3032 (2005).

9. Wheeler, M.A. *et al.* MAFG-driven astrocytes promote CNS inflammation. *Nature* **578**, 593-599 (2020).

10. Miller, S.D. & Karpus, W.J. Experimental autoimmune encephalomyelitis in the mouse. *Curr Protoc Immunol* **Chapter 15**, 15 11 11-15 11 18 (2007).

11. McMahon, E.J., Bailey, S.L., Castenada, C.V., Waldner, H. & Miller, S.D. Epitope spreading initiates in the CNS in two mouse models of multiple sclerosis. *Nat Med* **11**, 335-339 (2005).

12. Luoqian, J. *et al.* Ferroptosis promotes T-cell activation-induced neurodegeneration in multiple sclerosis. *Cell Mol Immunol* **19**, 913-924 (2022).

13. Sousa, J.A. *et al.* GPx1 deficiency confers increased susceptibility to ferroptosis in macrophages from individuals with active Crohn's disease. *Cell Death Dis* **15**, 903 (2024).

14. Martinez, A.M., Kim, A. & Yang, W.S. Detection of Ferroptosis by BODIPY 581/591 C11. *Methods Mol Biol* **2108**, 125-130 (2020).

15. Geiger, R. *et al.* L-Arginine Modulates T Cell Metabolism and Enhances Survival and Anti-tumor Activity. *Cell* **167**, 829-842 e813 (2016).

16. Rodriguez, P.C., Quiceno, D.G. & Ochoa, A.C. L-arginine availability regulates T-lymphocyte cell-cycle progression. *Blood* **109**, 1568-1573 (2007).

Reviewer #3

(Remarks to the Author)

Summary of Key Results

Kerndl et al. (2024) investigated the metabolic changes that occur in the central nervous system (CNS) during inflammation using the MOG EAE mouse model of multiple sclerosis (MS). The authors analysed different phases of disease and found reduced arginine levels in the spinal cord and an increase of downstream metabolites, suggesting catabolism, especially in the acute inflammatory phase of EAE. This finding was associated with the accumulation of arginase 1 (Arg1) expression by monocytes/macrophages. Further genetic deletion of Arg1 in myeloid cells/monocytes reduced arginine catabolism and improved disease outcomes, suggesting a key role of arginine catabolism in neuroinflammation. Accordingly, the authors found that arginine deficiency worsened the disease, highlighting the importance of maintaining adequate arginine availability in neuroinflammation.

Originality and Significance

The role of arginine in immune regulation has been previously investigated, and other studies have shown that during EAE, CNS-infiltrated monocytes differentiate into an Arg1-expressing immune modulatory state, which may facilitate tissue repair (PMID: 29283442 and PMID: 30127427). Herein, the authors provide a somewhat different/complementary interpretation of the functional role of this population, which is strengthened by the use of cell specific genetic knockout animals. Similarly, while Arg1 expression has been shown in patients with MS, specifically PBMCs (PMID: 30128151), an extensive characterisation of its expression in MS lesions was lacking and is presented in this article. This is very important, given that previous data suggest that specific metabolic pathways (including Arginase metabolism) are different in mice vs humans during CNS inflammation (PMID: 25339954).

Response: We thank the reviewer for the overall positive critique, for the deep review (including a balanced view on the similarities/differences between our work and already existing literature) and for acknowledging the importance of our work. Please see our point-by-point response below.

Data & Methodology

The authors used a combination of ex vivo targeted metabolomics, MALDI-MS, gene expression (sequencing and re-analysis of publicly available snRNAseq datasets) to investigate the metabolic changes in the CNS during neuroinflammation. This multi-omics approach provided a complementary view of the metabolic landscape and allowed for the identification of key metabolic pathways, even though many of the observed findings (especially those in Figure 1 to 3) seem to be mostly correlative. The use of transgenic tools partially overcomes this limitation, but there are several and important drawbacks that still need to be addressed (see below).

Response: We don't think our findings in Fig. 1-3 are mostly correlative. We start by defining different metabolite trajectories in distinct disease phases (Fig.1). We find arginine perturbations are most significantly enriched in the CNS (Fig. 2a,b), subsequently providing evidence (microscopy, flow cytometry, MALDI-MSI) that likely Arg1^{+}iNOS^{-} Mdcs consume arginine within EAE lesions (Fig. 2, Fig. 3). Figure 4 provides links between Arg1^{+} Mdcs, lipids and ROS.

Although the use of the transgenic animals naturally allows us to test the experimental hypothesis that have been developed as a direct consequence of the experimental work in Fig. 1-3, these earlier experiments laid the foundations for this.

Appropriate Use of Statistics and Treatment of Uncertainties

The authors used appropriate statistical tests to analyze their data and reported the results with standard errors of the mean (SEM). They also acknowledged the limitations of their study, such as the use of a mouse model and the small sample size for some experiments.

Response: We thank the reviewer for stating the statistical analysis is appropriate.

Conclusions

The conclusions drawn by the authors are most of the times supported by their data. However, the manuscript also includes sentences that are not supported by the authors' experimental results. For example, lines 155-175 include a very speculative description of the significance of metabolic changes without any experimental data to support "impaired oligodendrocyte mitochondrial ATP production and survival". Also,

the interpretation of other metabolic changes, e.g. decreased reduced glutathione, is biased towards the peak-disease while exactly the same changes are present in the remission phase of disease as well (thus suggesting that oxidative stress is also present in chronic EAE tissues). These are two key examples of overstatements and interpretation of data, which should be corrected through the manuscript.

Response: We have significantly toned down on overstatements in the revised manuscript. Regarding the mentioned metabolic changes we removed the wording related to oligodendrocytes and write on **page 8, lines 156-158** "Suggesting impaired energy metabolism and mitochondrial dysfunction in diseased spinal cord, we observed decreased adenosine, adenosine monophosphate and neuroprotective creatine." Regarding the interpretation of reduced glutathione, we now make it clear, this particular metabolic change is also observed at recovery and write on **pages 8-9 lines 166-169** "Although the DLM reduced glutathione was unchanged between peak-disease and recovery, indicative of an importance in resolving oxidative stress during EAE, metabolites protective against oxidative stress (nicotinamide, nicotinic acid) that were decreased at peak-disease were elevated to homeostatic healthy levels at recovery (Fig. 1e)."

Suggested Improvements
Potentially being a very important and significant step towards the understanding of the contribute of cell metabolism in modulating monocyte/macrophage function, this paper falls short in some areas that require further major experiments to fully assess the validity of the proposed findings. These include:

1) Arg1 metabolism in the spinal cord and CSF. Data for known metabolites that play a role in arginine metabolism (ornithine, urea) are missing from the heatmaps of Fig 1 and Extended data Fig 2. If the problem is that these are not significant, the authors should provide these data nonetheless (including a power analysis to prove that their experiments were not underpowered). These are important to understand the preferential use of the Arginine substrate towards a certain pathway. Please provide raw data, normalised data, and statistical significance for all the metabolites tested in an excel file.

Response: Thanks for this comment. We did not include data on ornithine or urea because they were not in our original panels. Please see supplementary Table 1 for the list of metabolites included in the original submission. We have now measured orthinine and several polyamines and did so in identical animals to the data corresponding to Fig. 1e, f and Extended Data Fig. 2. This new data demonstrates that ornithine, putrescine and spermidine were elevated in spinal cord and putrescine was elevated in CSF (new Fig. 2j, Supplementary Table 6).

Analysis of urea requires a separate procedure for analysis, including sample collection to prevent exchange with media components, extraction, and chemical derivatization to increase molecular weight and improve chromatography. Alternatively, commercial kits are available but require fresh samples and are fully consumptive of the material. Moreover, rapid exchange of urea between the cell and the extracellular environment (both in vivo and during processing) further complicates this analysis. In our case, given the material limitation of our model, we elected to perform polar metabolomics and polyamine analysis, which are incompatible with urea. While we agree with the reviewer that it would be nice to have urea measurements, given the polyamines are downstream of Arg1 (and we have now measured these), we hope the reviewer will agree with us that the lack of urea measurements in spinal cord and CSF does not affect the overall impact of our paper.

Nonetheless, based on point 8 of the reviewer, we have examined urea in the EAE-conditioned media (EAE-CM) experimental setup. Further indicating arginine catabolism is mediated by $Arg1^+$ Mdcs at peak-disease, we could demonstrate that analogous to whole spinal cord of peak-EAE suffering animals, versus healthy-conditioned media (healthy-CM), downregulations in arginine are associated with upregulations of ornithine and urea (Extended Data Fig. 9b).

We have provided an excel file for metabolomics source data corresponding to Figs 1e, f, Extended Data Fig. 2 (raw data, normalized data and statistical significance) for the reviewer. We indicate this in the data availability section and are happy to provide any other source data. Should the manuscript be acceptable for publication we will include all source data.

2. Arg1/iNOS activity. The authors need to provide additional evidence to show how

the expression of genes regulating both Arg1, Arg2, and iNOS and their enzymatic activity changes in the EAE tissues and in ex vivo isolated cells at different phases of disease.

Response: Although we think an exhaustive examination of the expression of genes regulating both *Arg1*, *Arg2*, and *iNOS* and their enzymatic activities therein is out-with the scope of our already very extensive manuscript, we agree as we examine the relative contribution of *iNOS* versus *Arg1*-expressing Mdcs to arginine catabolism, it would be important to measure NOS activity. This new data demonstrates that versus health, NOS activity is elevated and reduced at recovery (New Fig. 2i). We now write on **page 10, lines 208-210** "Further, Arg1 and NOS activity were elevated in spinal cord at peak-disease and although post recovery activity of both decreased, Arg1 activity remained modestly increased compared to health (Fig. 2h,i)."

We always aim to adhere to the 3Rs of animal experimentation (reduction, refinement and replacement) and thus try to reduce on mouse numbers wherever possible. Given that 1) enzymatic activity assays of *ex vivo* isolated spinal cord would require a lot of animals (via cell sorting we obtain around 150,000 freshly infiltrating $CCR2^+CX3CR1^+P2RY12^-$ Mdcs from one peak EAE suffering animal), 2) Enzymatic/metabolic activity might also be changed during the isolation process versus immediate freezing of spinal cord and CSF, which preserves metabolic integrity [1,2] and 3) such data would likely not yield anything new versus the current enzymatic activity assays from whole spinal cords employed, we feel this is not necessary and hope the reviewer agrees.

However, to more clearly demonstrate that cytokines within the CSF are associated with Mdc influx and effector function and hence Arg1 and NOS2 activity, we have shifted the CSF cytokine data from old Extended Data Fig. 7a to Fig. 2f. We write on **page 10, lines 205-208** "*Arg1* and *Nos2* expression patterns during EAE were mirrored by CSF levels of cytokines well associated with monocyte influx and pro-inflammation including MCP-1 and TNF-α, as well as the Mdc effector cytokines IFN-γ and GM-CSF (Fig. 2f, g)."

3. Fig 2i does not show clearly the colocalization of markers. Separation of single

stainings in different panels and quantification of single/double labelled cells in EAE peak and remission is needed.

Response: We have now provided single images for old Fig 2i and included a quantification (new Fig. 3a,b). As the reviewer requested we separate the Wheeler data set into the different phases and this demonstrates that $Arg1^+$ Mdcs persist at remission (new Extended Data Fig. 7d,e) and arginine levels are not different between peak and recovery in both the spinal cord and CSF (Fig. 1e, Extended Data Fig. 2e), we believe performing the requested staining at recovery would unlikely provide additional insight and hope the reviewer agrees.

4. Human pathology. The authors need to show if there are any differences in Arg1 expression in the different MS subtypes. Also, they need double immunolabelling for a myeloid cell marker (besides CD68, in Fig 2l, which is also not convincing without proper z stack) vs astrocytic and neuronal markers, to show staining specificity. iNOS and Arg2 stainings for all these conditions should be also provided.

Response: Thanks for this important suggestion! We have now provided immunofluorescence and z-stack data showing that $Arg1^+IBA\text{-}1^+$ myeloid cells are found in active lesions centres of MS patients. Consistent with literature[3,4], we observed strong cytoplasmic Arg1 localization. Double immunofluorescence with astrocytic and neuronal markers demonstrated staining specificity (new Fig. 3e,f). Given our manuscript focuses on Arg1, we don't think performing an Arg2 staining is necessary, but we did perform the iNOS staining. Although only a limited number of MS patients were tested (n = 3), this data suggests that similar to murine EAE, the majority of myeloid cells in human MS are $Arg1^+iNOS^-$ (new Fig. 3e,f). We now refer to this important data on **page 13, lines 291-296** and write: "Immunofluorescence of Arg1 and iNOS together with the myeloid cell marker IBA-1 demonstrated that analogous to murine EAE, most myeloid cells in human MS lesions were $Arg1^+iNOS^-$, with z-stacks revealing pronounced cytoplasmic Arg1 localization. Further, although as expected glial fibrillary acidic protein (GFAP) positive astrocytes were found in LCA, they did not express Arg1, nor did SMI32 positive neurons[5] in the grey matter (Fig. 3e,f)."

Regarding differences in Arg1 expression in the different MS subtypes employed in our study (Supplementary Table 7), we have split up Fig.3c into the different subtypes. Although our patient cohort comprises a small number of MS patients comprising different subtypes, rendering definitive conclusions therein difficult, in both primary progressive and relapsing-remitting MS (PPMS and RRMS respectively), versus the periplaque white matter (PPWM), Arg1 expression appears increased in the rim of chronic active lesions (RIM CA), while this was not observed in patients with secondary progressive MS (SPMS). Further, most Arg1 was found in the active lesions centres (LCA) of acute MS patients. Please find the data below.

Figure 1

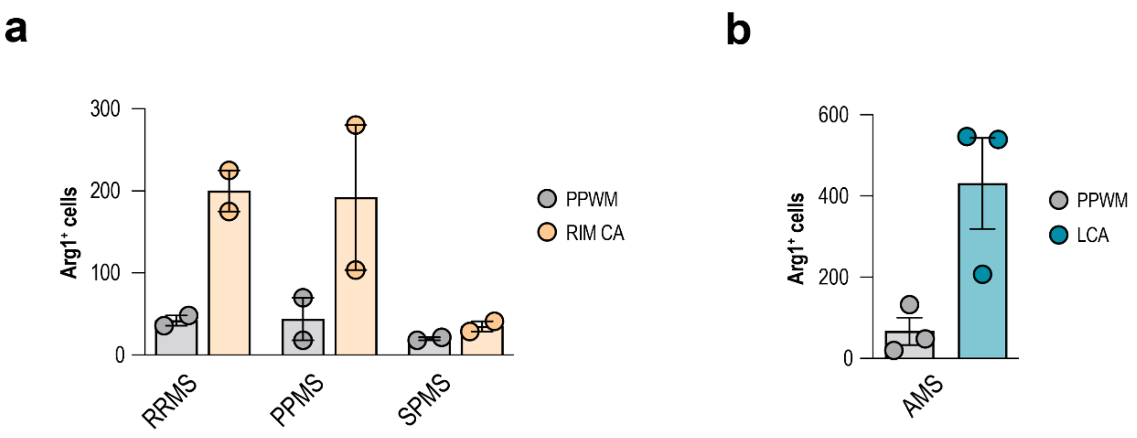

Figure 1. Arg1 expression in human MS lesions
Quantification of immuno-histochemical Arg1 staining of human MS brain sections. PPWM, periplaque white matter; RIM CA, rim of the active center; LCA, lesion center active; RRMS, Relapsing Remitting MS; PPMS, Primary Progressive MS; SPMS, Secondary Progressive MS; AMS, Active MS.

5. SnRNAseq human datasets. These are needed to confirm the pathology data and provide an unbiased assessment of Arg1/2/inos expression in different cell subtypes in MS and controls. A reanalysis of available datasets should be sufficient.

Response: We have now mined several different publicly available human SnRNAseq data-sets and provide the reviewer below the data from two of these[6,7]. In the data-sets we mined, despite detecting *P2ry12* and *Cd14* positive microglia and

macrophages, with *P2ry12* being downregulated in MS patients versus controls, we could not detect *Arg1*, *Nos2*, or *Arg2*. Please find below the data.

Figure 2

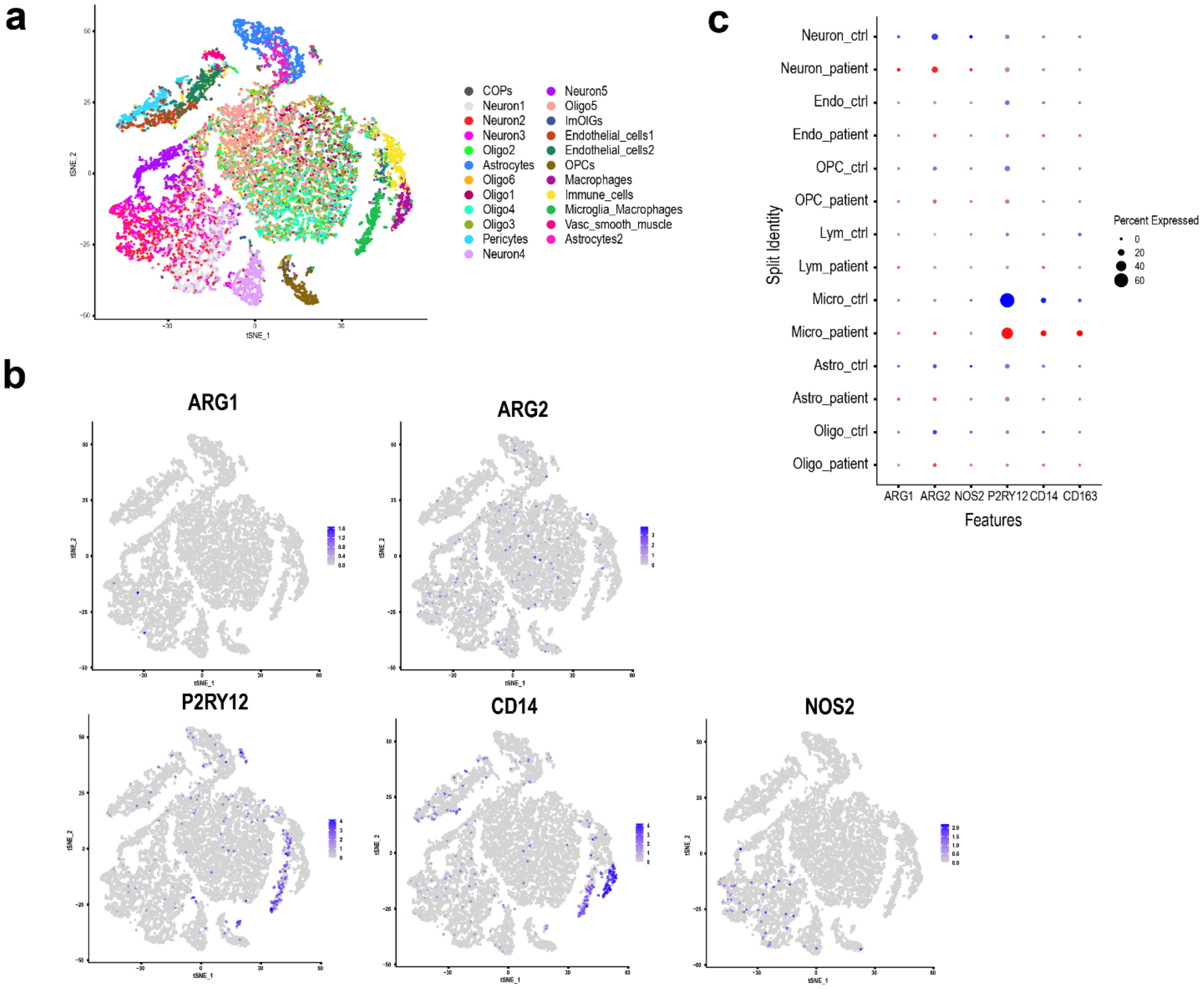

Figure 2. snRNAseq Human MS
a) tSNE projection of all cell types from snRNAseq dataset GSE118257. Number of nuclei is n = 17,799 combined from five control and four MS patients. Immune cells, n = 423, Macrophages = 368, Microglia_Macrophages = 428 nuclei. b) tSNE projections of *Arg1, Arg2, Nos2, P2ry12* and *Cd14* from (a). c) Dot plot depicting expression of the indicated genes in GSE301908. Number of nuclei is n = 293,970 of which microglia n = 25,036 from which n = 5423 nuclei (control) and n = 19,613 nuclei (patient). Data comprises 3 healthy controls and 14 patients with MS.

Although there are a limited number of EAE snRNA seq data-sets, we utilized one that identified multiple cell types at EAE onset including microglia and infiltrating Mdcs[8]. Indicating that SnRNA seq technology does not sufficiently detect *Arg1* or *Nos2*, we

could not detect either genes in *Ccr2* and *Lyz2* expressing Mdcs. Please find below the data.

Figure 3

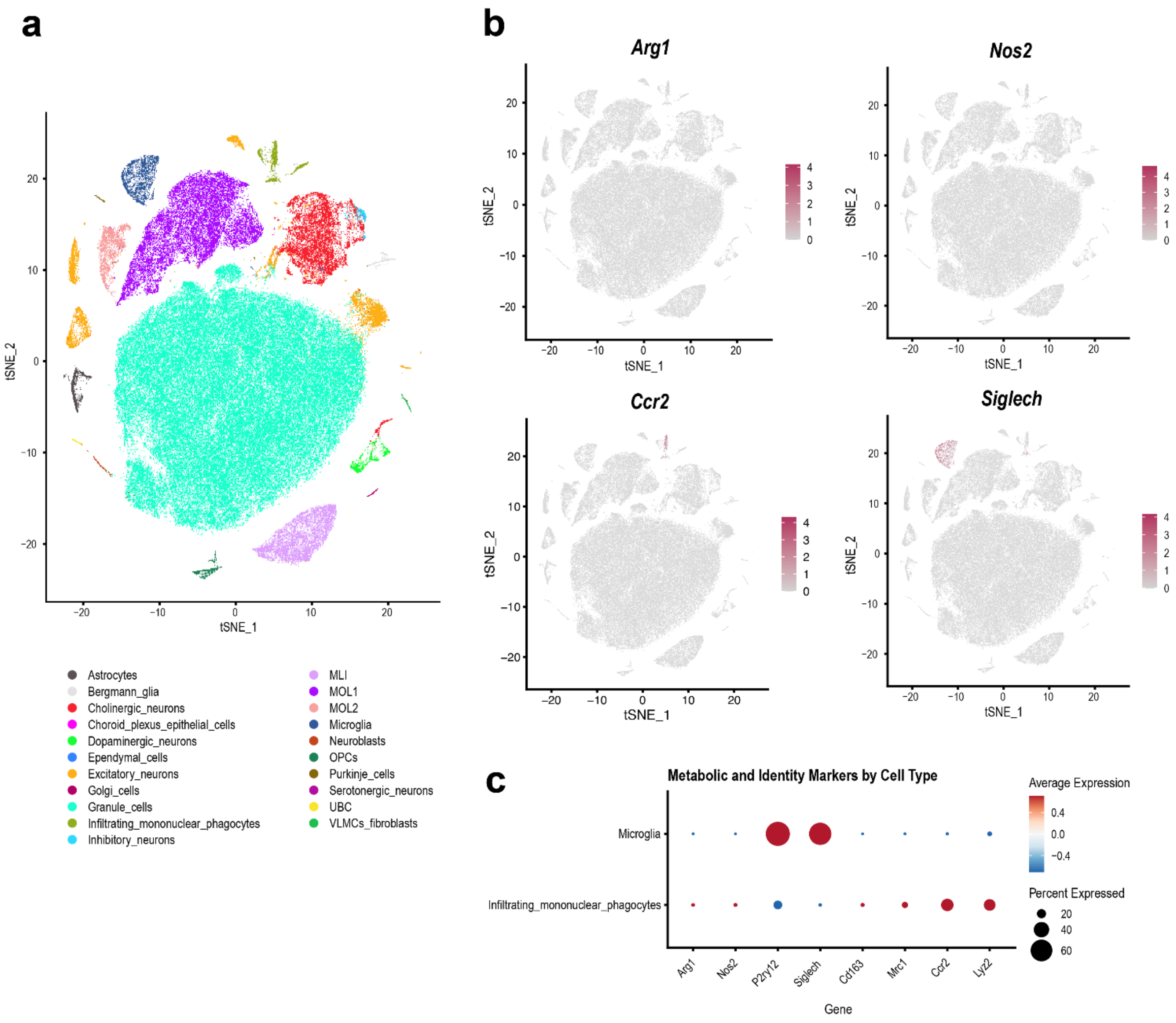

Figure 3. snRNAseq Murine EAE
a) tSNE projection of all cell types from snRNAseq dataset GSE236540. Number of nuclei is n = 133,151 nuclei combined from (n = 4 animals per genotype) at EAE onset (10 d.p.i.). Number of microglia nuclei is n = 1964 and infiltrating mononuclear phagocytes n = 1359. b) tSNE projections of *Arg1, Nos2, Ccr2* and *Siglech* from a). c) Dot plot showing expression of the indicated genes from microglia and infiltrating mononuclear phagocytes in (a).

Since *Arg1* is found in infiltrating Mdcs during spinal cord injury[9], we examined *Arg1* expression in a publicly available SnRNA seq spinal cord injury atlas that utilized 67,903 nuclei[10]. https://seqseek.ninds.nih.gov/spinalcordinjury. We could not robustly detect it in this data set either.

There are well established differences between ScRNAseq and SnRNAseq technologies. ScRNA-seq detects full-length and cytoplasmic mRNAs, while SnRNA-seq is biased toward towards nascent or incompletely spliced transcripts. It likely to miss lowly expressed transcripts or those that are rapidly exported to the cytoplasm[11,12]. Further, it has been reported that snRNA seq is not suitable for the detection of genes related to microglial activation[13]. Overall, we conclude snRNA seq technology is not suitable for detecting *Arg1*.

6. In situ metabolomics. MALDI-MS data are partial and do not assess many of the metabolites detected in Fig1. Even given differences in methodologies, this should be addressed, as there is a lack of correspondence between the two datasets that makes most interpretations correlative at most. There is also a need to provide better images without artifacts (e.g., see ganglioside levels found increased in the tear of the tissue of the peak EAE mouse of Extended Data Fig 5) with better/higher magnification images (showing at least markers for myeloid cells in the interested areas). Finally, the authors should provide raw data, normalised data, and quantification with statistical significance for all the metabolites tested in an excel file comparing different spinal cord regions.

Response: We have MALDI-MSI data on many other metabolites (several are found in different locations in the spinal cord) but believe including this data in our manuscript is not necessary as the particular metabolites we did include are focused on the arginine pathway. We have now provided better/zoomed images (Fig. 3g, Extended Data Fig. 6b) and have quantified the depicted metabolites (Fig. 3h, Extended Data Fig. 6c). Given myeloid cells are predominantly found in EAE lesions, we think including a co-localization with the metabolites is not warranted. As requested by the reviewer, we have provided a source excel file containing raw data, normalized data and statistical significance as indicated in the data availability section.

7. Reanalysis of the Wheeler dataset. This approach is instrumental to the authors' paper, but data need to be stratified in the different phases of EAE (priming, peak, remission) instead of being presented altogether. Also, the authors should use these data to exclude that the increased arginase activity is related to other cell types beyond myeloid cells. Please note that cluster 1 in Fig 3i is microglia/macrophages and not only macrophages. Also, the sub-clustering of Slc7a2high and Slc7a2low cells in in Extended Data Fig 6c seems to be including way more cells than those reported to express this gene in Extended Data Fig 6b. Finally, to see what genes are varying with the Arg1 cluster, an analysis of covariance (e.g. MOFA) is needed.

Response: As mentioned in point 3, we have now stratified the Wheeler dataset (GSE130119) into the different EAE phases, which demonstrates that although $Arg1^{+}$ Mdcs are found in all disease phases, most are found at peak-disease (Extended Data Fig. 7d, e). An analysis of data at peak-disease revealed that no other cell types beyond myeloid cells in cluster 2 robustly express Arg1 (Extended Data Fig. 7b). Thanks for pointing us out to the mistake in the labelling of cluster 1, which we have now corrected (Fig. 4f).

Regarding the sub-clustering of *Slc7a2*^{high} and *Slc7a2*^{low} cells in old Extended Data Fig 6c (now Extended Data Fig. 7f), due to the specifics of scRNAseq technology (i.e. high incidence of false negatives), the two clusters are identified by overall transcription profile similarity, as is usual in the field (using Seurat functions FindNeighbors and FindClusters). *Slc7a2* is an excellent marker gene for these two transcriptionally different sub-clusters of cluster 2. Hence, the "*Slc7a2*^{high}" subcluster also contains cells without a single *Slc7a2* read. For the reviewer's reference, please find below violin plots for *Slc7a2* in the high vs low sub-cluster below.

Figure 4

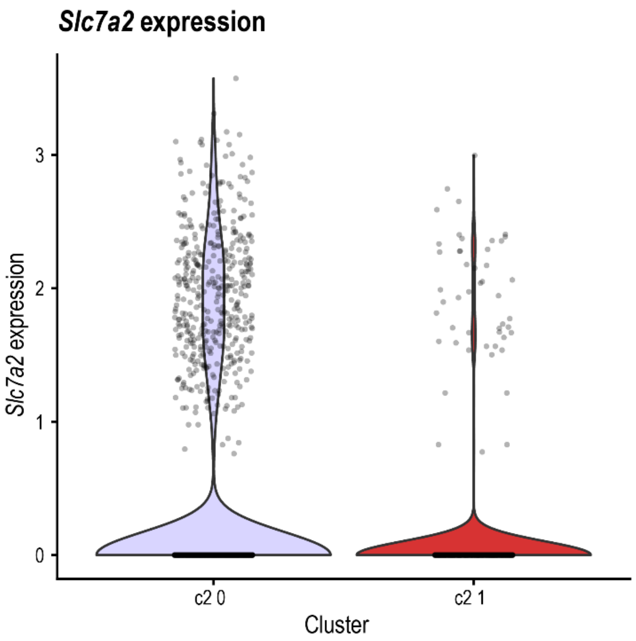

Figure 4. *Slc7a2* expression
Slc7a2 expression in *Slc7a2*^{hi} and *Slc7a2*^{lo} sub-clusters of cluster 2.

We performed the requested MOFA analysis on cluster 2, observing that there many varying genes inside this cluster. For a summary please see Fig 5 below, showing top 10 genes from the first seven factors. Among them there are genes related to lipid-ladenness and lysosomal function/myelin breakdown (*Lgals3, Fabp5, Spp1, Ccl6, Ctsb, Psap*) associated with Arg1, as well as genes related to antigen presentation/dendritic cell function (*H2-Aa, H2-Ab1, H2-Eb1, Cd74*).

Figure 5

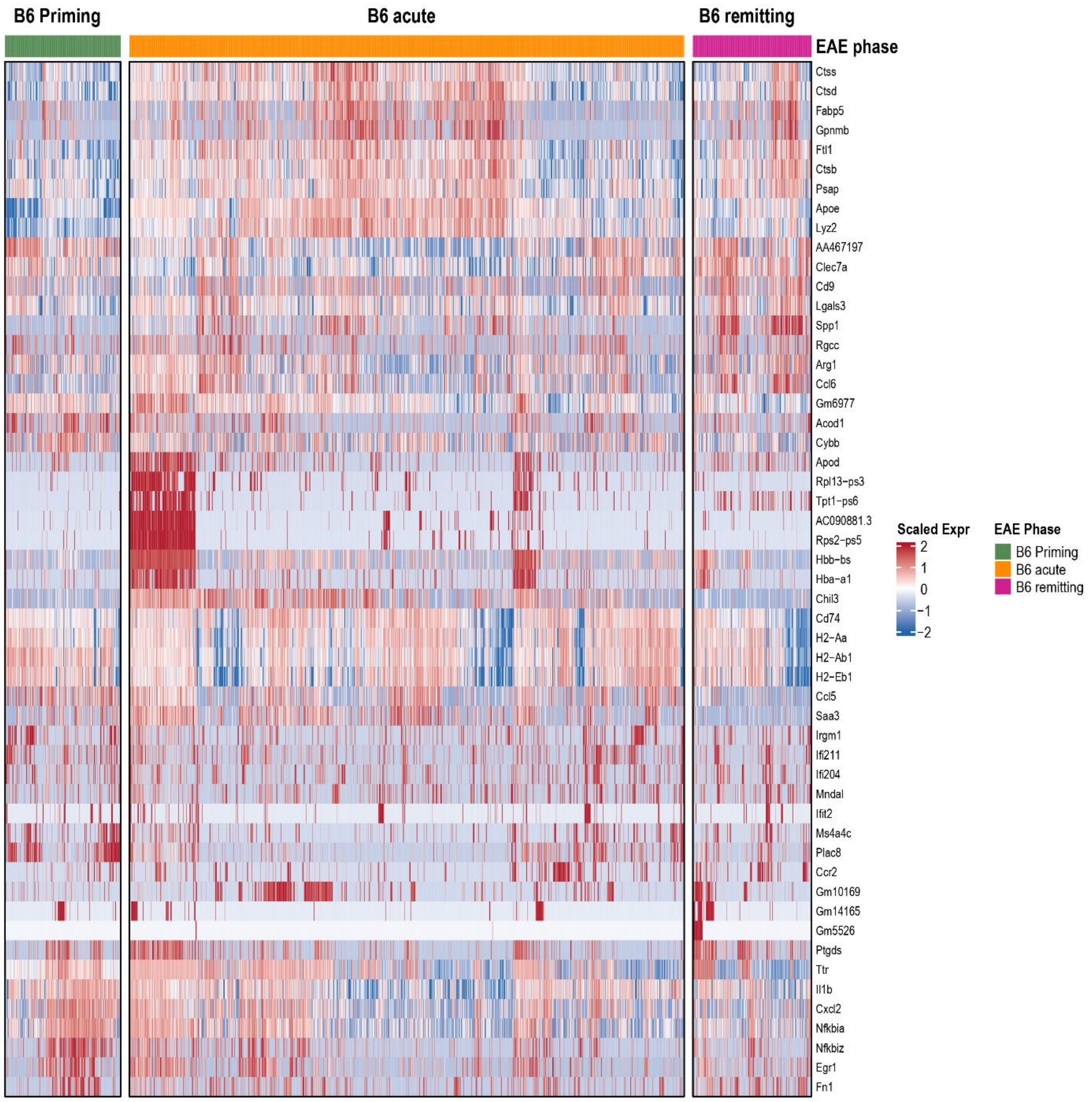

Figure 5. Genes varying in cluster 2
Heatmap showing top 10 genes from the first seven factors from MOFA performed on cluster 2 (53 genes in total), with cells split according to EAE phase and clustered by similarity.

Therein, we identified that factor 4 (which we designate as a "lipid remodelling factor") from the MOFA analysis captured gene expression programs involving lipid-ladenness/remodelling, possibly indicate Arg1^{+} cells represent a gradient of lipid remodelling states. Supportive, we observed a strong gradient of Arg1 with factor 4 on a UMAP and positive MOFA weights with the aforementioned lipid remodelling

genes (Fig 6, below). Interestingly, *Slc7a2* expression within cluster 2 strongly associates with factor 4 (p value = 0.0016), suggesting import of arginine is associated with lipid metabolism.

Figure 6

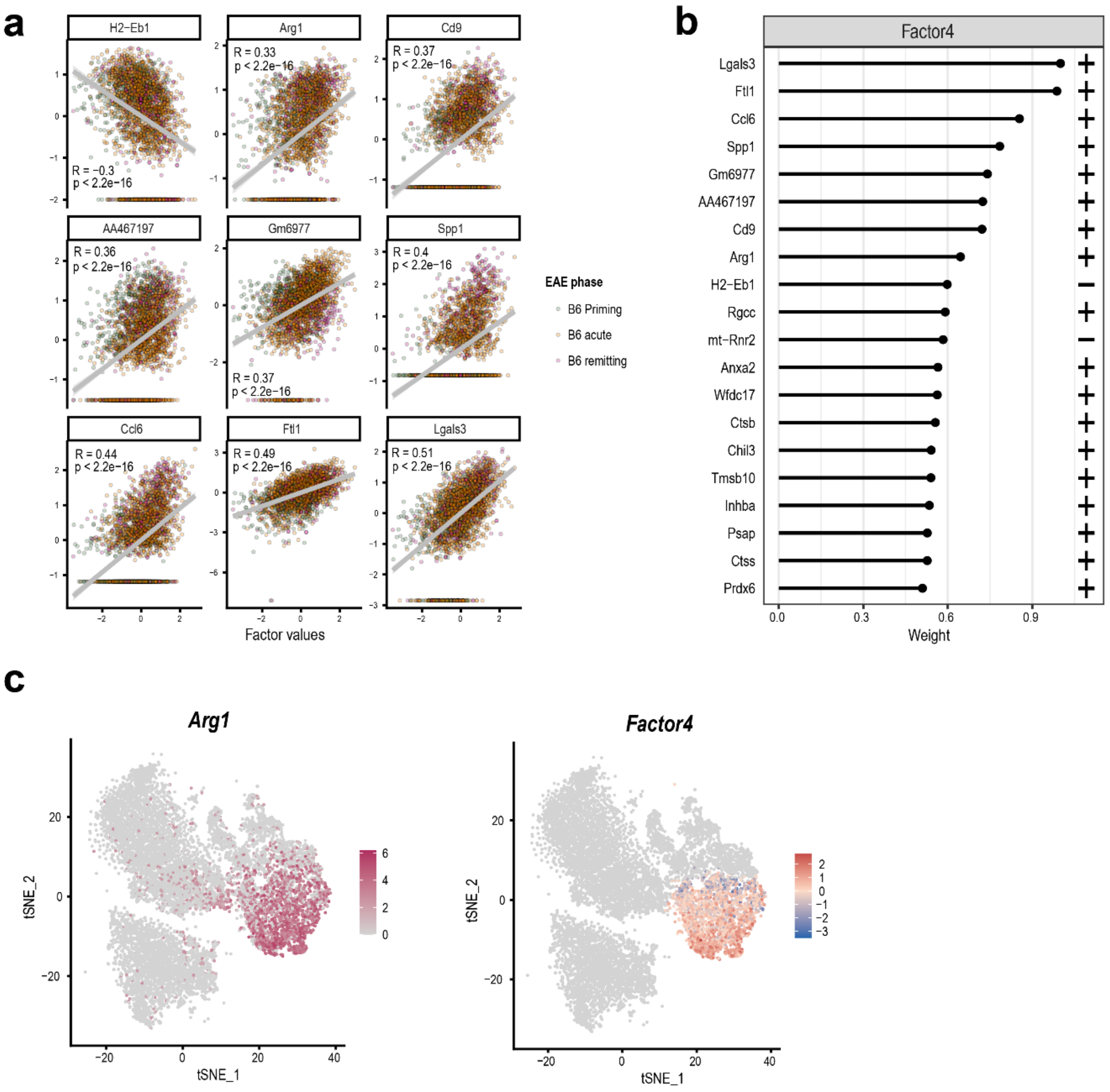

Figure 6. Analysis of factor 4 from MOFA analysis performed on cells from cluster 2
a) Association of top 9 most associated genes with factor 4, coloured by EAE phase. b) Weights of top 20 most associated genes with factor 4. The sign on the right depicts whether the association is positive (+) or negative (-). c) The expression of Arg1 across all cells and values of factor 4 across cells (only calculated for cluster 2).

We thank the reviewer for this suggestion, which further strengthens our work. However, due to space restrictions, we have decided not to put in the paper.

8. In vitro experiments. Data from unstimulated controls is missing from Extended Data Fig 7d, as well as IL-4/13 stimulated controls are missing from Extended Data Fig 7e. Please keep the experimental groups consisting throughout Extended Data Fig 7. Moreover, what is the metabolic and proteomic composition of the EAE CM experiments? This seems rather a crude experimental setup with many potential confounding players. Finally, what is the link/mechanism regulating arginine metabolism and the observed alterations of lipid metabolism? Metabolic flux data are needed to understand how arginine catabolism affects the intracellular and extracellular metabolome/lipidome of myeloid cells.

Response: We have added untreated controls to old Extended Data Fig. 7d (new Extended Data Fig. 8b). As reviewer #1 asked us to also examine Arg1/iNOS expression under LPS, IFN-γ and GM-CSF conditions alone, this figure has significantly changed. Our main question in this figure is how GM-CSF alters Arg1/iNOS expression in the context of LPS, which we ask because subsets of $Arg1^{+}$ Mdcs resemble LDAMs that are characterized by lipid droplet formation, high ROS levels and pro-inflammation triggered by LPS[14]. We included the IL4/IL-13 control because this is a very well established Arg1 inducer that is associated with arginine catabolism, which we have now added supporting ELISA data for (new Extended Fig. 8d). Hence, we reason performing metabolomics on this condition is unnecessary and hope the reviewer agrees. We have tried to keep the experimental groups consistent throughout Extended Fig. 8. However, as this figure primarily focuses on how LPS/GM-CSF promotes an $iNOS^{-}Arg1^{+}$ signature instigating arginine catabolism and promoting ROS production, data from this condition is naturally more emphasized.

Although the EAE-CM setup might seem crude, others have used similar approaches[15]. We think a proteomic characterization of EAE-CM is out-with the scope of our study. However, we have performed the suggested metabolomics characterization, focusing specifically on arginine-related metabolites. This new data demonstrates that versus healthy-conditioned media (healthy-CM), arginine

downregulation in EAE-CM is associated with orthinine and urea upregulation (Extended Data Fig. 9b).

We think performing metabolic flux experiments, i.e. specifically tracing arginine into lipids would not yield a meaningful outcome as lipids are generated from glycerol-3-phosphate or mitochondrial derived acetyl-CoA[16]. However, to further support the impact of arginine on macrophage lipid-ladeness/metabolism we have utilized the EAE-CM setup in conjuction with lipidomics. EAE-CM that contains more GM-CSF makes macrophages more lipid-laden and induces Arg1 (Fig. 4l-m). Lipidomics revealed that compared to healthy-CM, EAE-CM contains several different lipid species (new Extended Data Fig. 9a) and that addition of L-arginine decreases EAE-CM induced macrophage lipid load (new Fig. 6m). Together our findings suggest that EAE-CM induces Arg1 via GM-CSF instigating arginine catabolism and that this in turn further augments lipid ladenness. Nonetheless, as mentioned in the discussion (**page 26, lines 606-607**) whether arginine availability influences lipid uptake, synthesis, catabolism or efflux remains to be elucidated.

9. Mechanistic studies to prove toxicity. These are needed. They should include direct neurotoxicity and oligotoxicity of Arg1+ vs Arg1- BMDM in vitro, as well as neuropathological data (immune cell subtypes, Bielschowsky, and LFB) from all EAE studies.

Response: We now provide LFB data in the arginine free diet setup (new Fig. 6c,d). As in this experimental setup we now provide further supporting data indicating that arginine scarcity is associated with oxidative damage measuring MDA-adducts and 4-HNE in spinal cords (new Fig. 6f), we don't think the suggested Bielschowsky staining is essential to support the key findings. Further, we already assessed several immune cell types in our EAE models using flow cytometry including T cell subsets and Mdcs. We're not sure what the reviewer is referring to by suggesting to assess neurotoxicity and oligotoxicity of Arg1+ vs Arg1- BMDM, but believe a co-culture of oligodendrocytes and neurons with Arg1 deficient versus -sufficient BMDMs is out-with the scope of our study. In this regard, previous work has demonstrated that L-arginine deprivation is associated with decreased neuronal viability[17].

10. Transgenic mice. Please note that all the experiments (in vitro and in vivo) using the Arg1fl/fl Cx3cr1cre/+ mice need an additional control. That is Arg1wt/wt Cx3cr1cre/+ mice. The reason being that it is known that the expression of the cre under the cx3cr1 promoter generates a partial loss of function for Cx3cr1, which could affect the results. Moreover, all the experiments with KO animals need to be accompanied by quality controls showing the efficient knockout of the target gene and protein over time (this is particularly important for the CCR2 promoter, given that the paradigm of tamoxifen treatment was likely chosen to optimise this). Finally, MALDI-MS data for these in vivo transgenic experiments are likely needed to tie this paper together.

Response: We acknowledge the limitations of our experimental setup utilizing Arg1$^{fl/fl}$ Cx3cr1-cre animals, which aside from broad targeting activity of the Cre also include potential confounding effects of Cx3cr1 haploinsufficency. However, unlike EAE suffering *Arg1*$^{fl/fl}$ *Cx3cr1*-cre animals which exhibited better outcome compared to *Arg1*$^{fl/fl}$ littermate controls, previous work demonstrated that CX3CR1 deficient and heterozygous animals exhibit worsened outcome during EAE, making it unlikely that CX3CR1 haploinsufficency comprises our phenotype. We have now included the limitations of this experimental setup in the discussion (**pages 24-25, lines 577-584**).

We kindly disagree on the necessity of showing our knockout over the course of EAE We already provide flow cytometry proof that at peak-disease (i.e. when Arg1$^+$ Mdcs are prominent in spinal cord), Arg1 protein levels are attenuated in both mouse strains (Extended Data Fig. 10a,b,d,e). Given the extensive scope of our manuscript, we believe inclusion of MALDI-MSI in these transgenic experimental setups is not essential.

11. Arginine deficiency or supplementation. Arginine, being an essential amino acid, is only partially regulated by diet and can be synthetised locally. Therefore, the authors need to prove how their arginine free diet (and L-arginine supplementation) impact the dynamics and levels of circulating arginine, at different time points, and in the CNS.

Response: We clearly show arginine levels are attenuated in the blood and CSF of animals fed the arginine free diet prior to EAE (Fig. 6a). As mentioned earlier, we

always try to reduce on mouse numbers wherever possible. Given we provide multiple lines of evidence that arginine scarcity has pleiotropic negative effects in neuroinflammation and that a high number of animals would be required for such an analysis, we think that an examination of circulating and CSF arginine levels during the EAE time course in the arginine-sufficient and -deficient diet setup is not strictly necessary and hope the reviewer agrees. At the suggestion of another reviewer, we have removed the Treg arginine supplementation data set (old Fig. 5k) from the revised version.

References

The authors appropriately cited previous work and acknowledged the contributions of other researchers in the field. However, they should provide a more comprehensive list of references when describing the metabolic changes seen in the CSF/CNS using mouse models of MS (e.g., PMID: 22448154, 29478844, 36210459) and correlate them with their own findings. Also, the potential controversy around the differences of arginine metabolism in monocytes from mice and humans needs to be discussed (PMID: 30467554).

Response: We have now cited several of the mentioned references and included a section on the potential controversy about arginine catabolizing enzymes in humans in the discussion and write on **page 25, lines 589-592**, "Although there is controversy regarding the Arg1 and iNOS expression in human myeloid cells, others have demonstrated that Arg1 is present in PBMCs of MS patients. Despite the small number of MS patients analyzed, we now expand on these findings by revealing that Arg1-expressing IBA1$^+$ and CD68$^+$ myeloid cells are found in human MS lesions."

Clarity and Context

The abstract, introduction, and conclusions of the article are quite clear. The main problem (besides the issues with some of the conclusions, as written above) is that at least in two occasions, figures/panels are not cited in order of appearance in the text, making it unnecessarily hard to follow the results. For example, the authors reference Extended Data 1a, b, in line 123, which is followed by Extended Data 2a, b, in line 125. But then the authors go back to Extended Data 1c in line 142, instead of

continuing their numbering with Extended Data 2. Same thing happens for the panels i-l of Figure 3. Finally, the choice of referring to disease-associated metabolites as DAMs is highly questionable, given that the scientific community preferentially uses "DAM" to refer to disease associated microglia instead (PMID: 29775591).

Response: Although we agree there might be a bit of jumping around between the Extended Data Figs, we have tried hard to keep these figures consistent with their content/message. Thanks for the point regarding the DAM abbreviation which we agree is confusing. We have now changed this to DLM (Disease-linked metabolite).

References

1. Llufrio, E.M., Wang, L., Naser, F.J. & Patti, G.J. Sorting cells alters their redox state and cellular metabolome. *Redox Biol* **16**, 381-387 (2018).

2. Rauckhorst, A.J. *et al.* Mouse tissue harvest-induced hypoxia rapidly alters the in vivo metabolome, between-genotype metabolite level differences, and (13)C-tracing enrichments. *Mol Metab* **66**, 101596 (2022).

3. Caldwell, R.W., Rodriguez, P.C., Toque, H.A., Narayanan, S.P. & Caldwell, R.B. Arginase: A Multifaceted Enzyme Important in Health and Disease. *Physiol Rev* **98**, 641-665 (2018).

4. Asrani, K.H., Cheng, L., Cheng, C.J. & Subramanian, R.R. Arginase I mRNA therapy - a novel approach to rescue arginase 1 enzyme deficiency. *RNA Biol* **15**, 914-922 (2018).

5. Watkins, L.M. *et al.* Complement is activated in progressive multiple sclerosis cortical grey matter lesions. *J Neuroinflammation* **13**, 161 (2016).

6. Jakel, S. *et al.* Altered human oligodendrocyte heterogeneity in multiple sclerosis. *Nature* **566**, 543-547 (2019).

7. Feng, R. *et al.* Single-cell spatial transcriptomic profiling defines a pathogenic inflammatory niche in chronic active multiple sclerosis lesions. *Immunity* **58**, 2989-3005 e2910 (2025).

8. Andreadou, M. *et al.* IL-12 sensing in neurons induces neuroprotective CNS tissue adaptation and attenuates neuroinflammation in mice. *Nat Neurosci* **26**, 1701-1712 (2023).

9. Greenhalgh, A.D. *et al.* Arginase-1 is expressed exclusively by infiltrating myeloid cells in CNS injury and disease. *Brain Behav Immun* **56**, 61-67 (2016).

10. Matson, K.J.E. *et al.* Single cell atlas of spinal cord injury in mice reveals a pro-regenerative signature in spinocerebellar neurons. *Nat Commun* **13**, 5628 (2022).

11. Andrews, T.S. *et al.* Single-Cell, Single-Nucleus, and Spatial RNA Sequencing of the Human Liver Identifies Cholangiocyte and Mesenchymal Heterogeneity. *Hepatol Commun* **6**, 821-840 (2022).

12. Kim, N., Kang, H., Jo, A., Yoo, S.A. & Lee, H.O. Perspectives on single-nucleus RNA sequencing in different cell types and tissues. *J Pathol Transl Med* **57**, 52-59 (2023).

13. Thrupp, N. *et al.* Single-Nucleus RNA-Seq Is Not Suitable for Detection of Microglial Activation Genes in Humans. *Cell Rep* **32**, 108189 (2020).

14. Marschallinger, J. *et al.* Lipid-droplet-accumulating microglia represent a dysfunctional and proinflammatory state in the aging brain. *Nat Neurosci* **23**, 194-208 (2020).

15. Haney, M.S. *et al.* APOE4/4 is linked to damaging lipid droplets in Alzheimer's disease microglia. *Nature* **628**, 154-161 (2024).

16. Chandel, N.S. Lipid Metabolism. *Cold Spring Harb Perspect Biol* **13** (2021).

17. Lee, J., Ryu, H. & Kowall, N.W. Motor neuronal protection by L-arginine prolongs survival of mutant SOD1 (G93A) ALS mice. *Biochem Biophys Res Commun* **384**, 524-529 (2009).
